# Synthesis and pharmacological evaluation of newly detected synthetic cannabinoid receptor agonists AB-4CN-BUTICA, MMB-4CN-BUTINACA, MDMB-4F-BUTICA, MDMB-4F-BUTINACA and their analogs

**DOI:** 10.3389/fpsyt.2022.1010501

**Published:** 2022-09-28

**Authors:** Eric Sparkes, Rochelle Boyd, Shuli Chen, Jack W. Markham, Jia Lin Luo, Tahira Foyzun, Humayra Zaman, Charlotte Fletcher, Ross Ellison, Iain S. McGregor, Marina J. Santiago, Felcia Lai, Roy R. Gerona, Mark Connor, David E. Hibbs, Elizabeth A. Cairns, Michelle Glass, Adam Ametovski, Samuel D. Banister

**Affiliations:** ^1^The Lambert Initiative for Cannabinoid Therapeutics, Brain and Mind Centre, The University of Sydney, Sydney, NSW, Australia; ^2^Faculty of Science, School of Chemistry, The University of Sydney, Sydney, NSW, Australia; ^3^Faculty of Science, School of Psychology, The University of Sydney, Sydney, NSW, Australia; ^4^Department of Pharmacology and Toxicology, University of Otago, Dunedin, New Zealand; ^5^Faculty of Medicine and Health, School of Pharmacy, The University of Sydney, Sydney, NSW, Australia; ^6^Macquarie Medical School, Macquarie University, Sydney, NSW, Australia; ^7^Clinical Toxicology and Environmental Biomonitoring Laboratory, University of California, San Francisco, San Francisco, CA, United States

**Keywords:** synthetic cannabinoid, cannabinoid receptor 1 agonists, pharmacology, cannabinoids, SCRAs, docking, *in vitro* evaluation, synthesis

## Abstract

Synthetic cannabinoid receptor agonists (SCRAs) continue to make up a significant portion new psychoactive substances (NPS) detected and seized worldwide. Due to their often potent activation of central cannabinoid receptors *in vivo*, use of SCRAs can result in severe intoxication, in addition to other adverse health effects. Recent detections of AB-4CN-BUTICA, MMB-4CN-BUTINACA, MDMB-4F-BUTICA and MDMB-4F-BUTINACA mark a continuation in the appearance of SCRAs bearing novel tail substituents. The proactive characterization campaign described here has facilitated the detection of several new SCRAs in toxicological case work. Here we detail the synthesis, characterization, and pharmacological evaluation of recently detected SCRAs, as well as a systematic library of 32 compounds bearing head, tail, and core group combinations likely to appear in future. *In vitro* radioligand binding assays revealed most compounds showed moderate to high affinity at both CB_1_ (p*K*_i_ = < 5 to 8.89 ± 0.09 M) and CB_2_ (p*K*_i_ = 5.49 ± 0.03 to 9.92 ± 0.09 M) receptors. *In vitro* functional evaluation using a fluorescence-based membrane potential assay showed that most compounds were sub-micromolar to sub-nanomolar agonists at CB_1_ (pEC_50_ = < 5 to 9.48 ± 0.14 M) and CB_2_ (pEC_50_ = 5.92 ± 0.16 to 8.64 ± 0.15 M) receptors. An *in silico* receptor-ligand docking approach was utilized to rationalize binding trends for CB_2_ with respect to the tail substituent, and indicated that rigidity in this region (i.e., 4-cyanobutyl) was detrimental to affinity.

## Introduction

**Figure 1:**
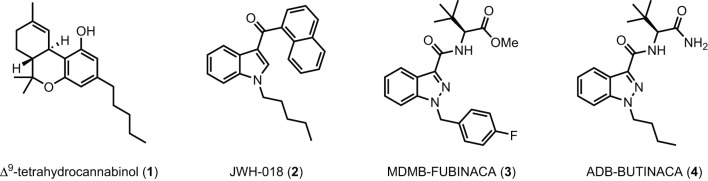
Δ^9^-THC **(1)**, JWH-018 **(2)**, and selected SCRAs MDMB-FUBINACA **(3)** and ADB-BUTINACA **(4)**.

Synthetic cannabinoid receptor agonists (SCRAs) represent a large portion of detected new psychoactive substances (NPS) globally, accounting for 29% of the 1,047 NPS identified between 2009 and 2019 (1). Commonly sold as herbal blends, such as “Spice,” “K2,” and “Black Mamba” which are consumed by smoking, SCRAs are part of a conscious effort by manufacturers and retailers to mimic the effect of Δ^9^-tetrahydrocannabinol (THC, **1**, Figure 1), the primary intoxicating compound in cannabis (2–6). Several mass intoxication events have been observed over the past decade, with clinically significant impact on patients including psychosis, seizure, respiratory failure, encephalopathy, necrotizing pancreatitis, acute kidney injury, and death (3, 7–31).

SCRAs primarily target the endocannabinoid system, specifically as agonists of centrally expressed cannabinoid 1 receptors (CB_1_), to provide users with a high analogous to cannabis, although these compounds generally display equal or better affinity and potency at CB_1_ compared with THC. However, unlike THC, SCRAs such as JWH-018 **(2)**, MDMB-FUBINACA **(3)**, and ADB-BUTINACA **(4)** generally act as high efficacy agonists at CB_1_ resulting in significantly greater intoxication of users, and display differing profiles of tolerance, dependence, and withdrawal (32).

Modern SCRAs typically consist of amino acid-derived indole- and indazole-3-carboxamide type scaffolds, similar to compounds disclosed by Pfizer in a series of patents in 2009 (33–38). Appearance of NPS containing modification of these scaffolds has seemingly been a result of incoming legislation bringing detected compounds under national and international control, while attempting to retain CB_1_ activity. Recent approaches observed include scaffold hopping, fluorination, nitrogen walking, alkyl chain contraction, and homologation, as well as the coupling of different amino acid residues to the substituted heterocyclic cores. These modifications have led to a significant structural evolution of contemporary SCRAs from the late 2000s, with equivalent or increased potency at CB_1_ (39–41).

**Figure 2:**
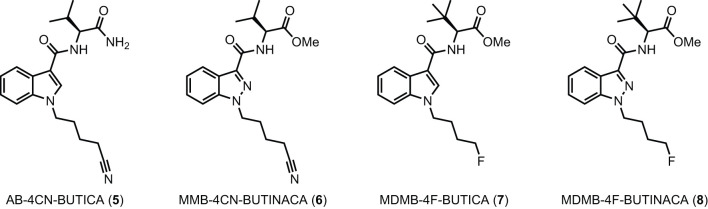
Detected SCRAs AB-4CN-BUTICA **(5)**, MMB-4CN-BUTINACA **(6)**, MDMB-4F-BUTICA **(7)**, and MDMB-4F-BUTINACA **(8)**.

**Figure 3:**
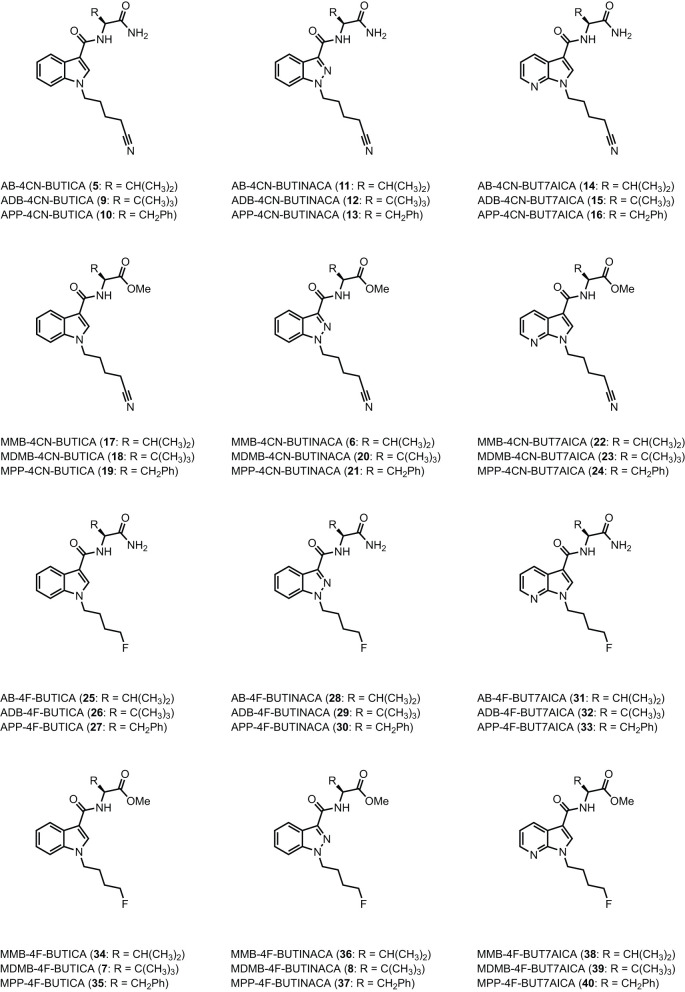
Structures of amino acid-derived 4-cyanobutyl and 4-fluorobutyl-substitued indole, indazole and 7-azaindole SCRAs explored in this study. AB, [(*S*)-2-amino-3-methylbutanamide]; ADB, [(*S*)-2-amino-3,3-dimethylbutanamide]; APP, [(*S*)-2-amino-3-phenylpropanamide]; MMB, [methyl (S)-2-amino-3-methylbutanoate]; MDMB, [methyl (S)-2-amino-3,3-dimethylbutanoate]; MPP, [methyl ((*S*)-2-amino-3-phenylpropanoate]; 4-CN-BUT, 4-cyanobutyl; 4F-BUT, 4-fluorobutyl; ICA, indole-3-carboxamide; INACA, indazole-3-carboxamide; 7AICA, 7-aza-indole-3-carboxamide.

The utility of proactive synthesis programs to detect emerging NPS has recently facilitated the detection of two new SCRAs bearing the 4-cyanobutyl tail moiety. The indole and indazole derivatives AB-4CN-BUTICA **(5)** and MMB-4CN-BUTINACA (**6**, a.k.a. AMB-4CN-BUTINACA), respectively, were identified in Alabama, facilitated by proactive reference standard generation and toxicological screening by our laboratories (Figure 2) (42, 43). The 4-fluorobutyl compounds MDMB-4F-BUTICA **(7)** and MDMB-4F-BUTINACA **(8)** were also recently detected, with the latter involved in a suicide, as well as multiple fatal intoxications (7, 31, 44–49). With the appearance of these compounds, it is feasible that NPS manufacturers may introduce the 4-fluorobutyl or 4-cyanobutyl subunits to other amino acid-derived indole-, indazole- and 7-azaindole-3-carboxamide SCRAs in future. As such, this paper describes the synthesis, chemical analysis, and pharmacological characterization of the newly detected SCRAs, as well as a series of 32 systematic analogs (5–40, Figure 3). Due to the combinatorial design of the library, direct comparison of the contributions of subunits to the pharmacological profile of the compounds enabled identification of key structure-activity relationships (SARs). *In vitro* binding and functional data were obtained at both CB_1_ and CB_2_, and an *in silico* docking approach was utilized to explore observed SARs, especially concerning differences in binding between 4-cyanobutyl and 4-fluorobutyl tail moieties. Most compounds displayed high affinity, potency, and efficacy at both CB_1_ and CB_2_, suggesting these compounds should be included in NPS monitoring programs. Given the similarity in structure and pharmacological profile of the library evaluated in this work compared to existing SCRAs, these data provide important insights into the potential effect of these compounds in humans, pending evaluation *in vivo*.

## Experimental

### General chemical synthesis details

All reactions were performed under an atmosphere of nitrogen unless otherwise specified. Methyl 1*H*-indazole-3-carboxylate **(42)** and methyl 1*H*-pyrrolo[2,3-*b*]pyridine-3-carboxylate **(43)** were purchased from Fluorochem LTD (Derbyshire, UK) and used as received. 1-Ethyl-3-(3-dimethylaminopropyl)carbodiimide hydrochloride (EDC•HCl) was purchased from Oakwood Chemical (Estill, SC, USA). Deuterated solvents (CD_3_OD, CDCl_3_, and DMSO-*d*_6_) were purchased from Cambridge Isotope Laboratories (Tewksbury, MA, USA). Unless otherwise stated, all other reagents and solvents used for this manuscript were obtained from Sigma-Aldrich/Merck (Castle Hill, NSW, Australia) and used as purchased. Analytical thin-layer chromatography was performed using Merck aluminum-backed silica gel 60 F254 (0.2 mm) plates (Merck, Darmstadt, Germany), which were visualized using shortwave (254 nm) UV fluorescence. Flash chromatography was performed using a Biotage Isolera Spektra One and Biotage SNAP KP-Sil silica cartridges (Uppsala, Sweden), with gradient elution terminating at the solvent combination indicated for each compound (*vide infra*). Melting point ranges (m.p.) were measured in open capillaries using a Stuart SMP50 Automated melting point apparatus (Cole-Palmer, Staffordshire, UK) and are uncorrected. Nuclear magnetic resonance (NMR) spectra were recorded at 298 K using an Agilent 400-MHz spectrometer (Santa Clara, CA, USA), or a Bruker NEO 300 MHz NMR spectrometer (Billerica, MA, USA). The data are reported as chemical shift (δ ppm) relative to the residual protonated solvent resonance, relative integral, multiplicity (s = singlet, brs = broad singlet, d = doublet, t = triplet, q = quartet, quin = quintet, m = multiplet), coupling constants (*J* Hz), and assignment. High resolution mass spectrometry (HRMS) data were collected using an Agilent LC 1260-QTOF/MS 6550 (Santa Clara, CA, US) or a Bruker Solarix 2xR 7T Fourier Transform Ion Cyclotron Resonance Mass Spectrometer. A methanolic extract of each pure standard was run using an electrospray ionization (ESI) source in automated MS/MS (information dependent acquisition) mode. Accurate mass for the parent ion and its corresponding mass error expressed in parts per million (ppm) are reported. LC-MS/UV data were acquired using a Shimadzu (Kyoto, Japan) Nexera LC-30AD UHPLC system coupled to a Shimadzu SPD-20AV photo diode array detector and a Shimadzu LCMS-8040 triple-quadrupole mass spectrometer, equipped with an electrospray ionization (ESI) source. Compounds were dissolved in a 90:10 mixture of 0.1% formic acid in water and methanol, and placed in the UHPLC autosampler that was maintained at 8°C. Elution was performed in gradient mode with an Agilent (CA, USA) Zorbax XDB-C18 column (2.1 × 50 mm, 3.5 μm particle size), held at 50°C, with a 10 μL injection volume. The mobile phases were 0.1% formic acid in water (A) and methanol (B). Mobile phase composition was held at 10% B until 0.5 min, then steadily increasing to 100% B at 2.5 min, holding until 3 min, and then re-equilibrating at 10% B for a total run time of 4 min. UV absorbance was measured from 190 to 800 nm. UV data from blank injections were subtracted to account for mobile phase absorbance and background noise. Single stage mass spectra (MS1) were collected in positive ESI mode using a single quadrupole (Q3) scanning from *m/z* 100–600. All data were processed using Shimadzu LabSolutions (v 5.89) software. Fourier-transform infrared spectroscopy (FTIR) spectra were collected using a Bruker Alpha II ATR FTIR spectrophotometer. Precursors **44–51**, and compounds **6, 7, 11, 17**, and **22** were synthesized and used as is, as previously described (50, 51). Synthetic procedures and characterization data (^1^H and ^13^C NMR, melting point, R_*f*_, FTIR, HRMS, UV, and LCMS) can be found in the supporting information.

### *In vitro* binding evaluation

Human embryonic kidney (HEK) cells expressing either human CB_1_ receptors N-terminally tagged with pplss (preprolactin signal sequence) and 3HA (3x haemagglutinin) epitopes or human CB_2_ receptor N-terminally tagged with 3HA were harvested in 5 mM EDTA in PBS, and “P2” membranes were prepared in sucrose buffer as previously described (52, 53). Protein content was estimated using a BioRad (Hercules, CA) DC protein assay (modified Lowry assay). For binding assays, radioligand ([^3^H]-CP55,940, PerkinElmer, Waltham, MA, USA), non-radiolabeled drugs, and P2 membrane preparations were diluted in binding buffer (50 mM HEPES pH 7.4, 1 mM MgCl_2_, 1 mM CaCl_2_, 2 mg/mL NZ-origin BSA, MP Biomedicals, Santa Ana, CA, USA) and dispensed into 96-well, polypropylene V-well plates (Hangzhou Gene Era Biotech Co Ltd, Zhejiang, China) in a final reaction volume of 200 μL (membranes were dispensed last). Final radioligand concentration was 1 nM for all assays. Protein content was 3 μg/point for pplss-3HA-hCB_1_ HEK membranes, and 2 μg/point for 3HA-hCB_2_ HEK membranes for the assays using type A harvest plate (PerkinElmer, GF/C filters, 1.2 μm pores), these plates were discontinued during the course of this study, and replaced with type B harvest plates (Merk Millipore, GF/C filters, 1.2 μm pores). As these plates appear to retain a smaller proportion of the protein, membrane content was increased to 8 μg/point for CB_1_ membranes, and 3 μg/point for CB_2_ membranes. When all components had been dispensed, the V well plate was sealed and incubated for 1 h at 30°C. During the incubation, the 96 well harvest plate was treated with 0.1% w/v branched polyethyleneimine (PEI; Sigma Aldrich) in H_2_O. Immediately prior to washing, PEI was washed through the filters using a vacuum manifold (Pall Corporation, Port Washington, NY) and all wells were washed once with ice cold wash buffer (50 mM HEPES pH 7.4, 500 mM NaCl, 1 mg/mL BSA). Equilibrated binding mixture was then transferred to the harvest plate under vacuum, and samples washed through. For the assays using type A harvest plate, binding wells were rinsed once with wash buffer and transferred to the harvest plate, and then wells were washed three more times with 200 μL of wash buffer. The plate was then removed, and filters allowed to dry overnight. The next day, the plate bottom was sealed, and 50 μL of Ultima Gold XR scintillation fluid (PerkinElmer) was dispensed to each well and the plate loaded into the 96 well “rigid” cassette. For the assays using type B harvest plate, samples were rapidly washed 4 times with 100 μL of wash buffer. The bottom of the plate was then removed, and filters allowed to dry overnight. The next day, the plate was inserted into a 96 well “rigid” cassette. The base of the cassette was sealed and 50 μL of scintillation fluid was added to each well. The top of the plates were sealed prior to loading into a Wallac MicroBeta2^®^ TriLux Liquid Scintillation Counter (PerkinElmer). Scintillation was detected after a 30 min delay, for 2 min per well. Counts were corrected for detector efficiency. Data were then exported and analyzed in GraphPad Prism v9 (GraphPad Software Inc., La Jolla, CA, USA). K_i_ was determined through fit of “Competition binding- One site fit K_i_” in GraphPad prism using a K_d_ of 3.50 nM for the radioligand for the assays using type A harvest plate; while for the assays using harvest plate B, K_d_ was 3.58 nM for binding at CB_1_ and 1.162 nM for binding at CB_2_ (K_d_ was determined empirically using homologous competition assay under matching conditions). Log K_i_ were determined for at least three independent experiments (maximum 6) and combined to determine mean pK_i_ ± SEM reported in Tables 1, 2. Assays for compounds **5, 6, 10–12, 14, 15, 17, 18, 20–22, 24, 29, 34** used type A harvest plate. Assays for compounds **7–9, 13, 16, 19, 23, 25–28, 30–33, 35–40** used type B harvest plates.

**Table 1:** Affinities and functional activities of 4-cyanobutyl derived SCRAs at hCB_1_ and hCB_2_.

**Compound**	**hCB**_**1**_			**hCB**_**2**_		
	**p***K*_i_ ±**SEM (***K*_i_**, nM)**	**pEC**_50_ ±**SEM (EC**_50_**, nM)**	**E**_max_ ±**SEM (% CP55,940)**	**p***K*_i_ ±**SEM (***K*_i_**, nM)**	**pEC**_50_ ±**SEM (EC**_50_**, nM)**	**E**_max_ ±**SEM (% CP55,940)**
AB-4CN-BUTICA (**5**)	6.26 ± 0.09 (550)	6.40 ± 0.08 (400)	111 ± 5	6.22 ± 0.13 (**603**)	7.12 ± 0.05 (75.9)	100 ± 2
ADB-4CN-BUTICA (**9**)	8.17 ± 0.12 (6.8)	7.99± 0.16 (10.2)	115 ± 5	8.26 ± 0.06 (5.5)	7.98 ± 0.21 (10.4)	106 ± 6
APP-4CN-BUTICA (**10**)	5.48 ± 0.11 (3310)	DNC^b^	85 ± 3^d^	6.11 ± 0.06 (**776**)	7.01 ± 0.08 (98.4)	95 ± 3
AB-4CN-BUTINACA (**11**)	7.36 ± 0.12 (43.7)	7.58 ± 0.14 (26.3)	111 ± 5	7.62 ± 0.07 (24.0)	8.30 ± 0.05 (4.97)	103 ± 1
ADB-4CN-BUTINACA (**12**)	8.09 ± 0.03 (8.13)	9.48 ± 0.14 (0.33)	109 ± 2	8.59 ± 0.05 (2.57)	8.75 ± 0.03 (1.76)	96 ± 2
APP-4CN-BUTINACA (**13**)	5.97 ± 0.09 (1100)	DNC^b^	104 ± 3^d^	6.78 ± 0.03 (170)	7.13 ± 0.25 (74.1)	107 ± 11
AB-4CN-BUT7AICA (**14**)	5.59 ± 0.12 (2570)	DNC^b^	80 ± 3^d^	5.49 ± 0.03 (3240)	6.41 ± 0.10 (390)	87 ± 5
ADB-4CN-BUT7AICA (**15**)	6.81 ± 0.09 (155)	7.85 ± 0.1 (14.0)	102 ± 4	6.84 ± 0.10 (145)	8.22 ± 0.03 (6.09)	96 ± 1
APP-4CN-BUT7AICA (**16**)	< 5	ND^c^	25 ± 3^d^	5.73 ± 0.08 (1860)	6.64 ± 0.09 (232)	72 ± 3
MMB-4CN-BUTICA (**17**)	6.80 ± 0.14 (159)	7.29 ± 0.06 (50.8)	105 ± 3	7.07 ± 0.12 (85.1)	7.87 ± 0.08 (13.5)	95 ± 3
MDMB-4CN-BUTICA (**18**)	8.18 ± 0.11 (6.61)	8.57 ± 0.13 (2.68)	111 ± 3	8.69 ± 0.05 (2.04)	8.39 ± 0.14 (4.04)	101 ± 4
MPP-4CN-BUTICA (**19**)	7.07 ± 0.09 (85.1)	7.45 ± 0.07 (35.5)	109 ± 3	8.06 ± 0.02 (8.71)	8.07 ± 0.08 (8.44)	105 ± 3
MMB-4CN-BUTINACA (**6**)	7.76 ± 0.06 (17.4)	8.44 ± 0.19 (3.61)	117 ± 6	8.19 ± 0.14 (6.46)	8.67 ± 0.17 (2.14)	101 ± 4
MDMB-4CN-BUTINACA (**20**)	8.89 ± 0.09 (1.29)	9.14 ± 0.14 (0.72)	113 ± 2	9.53 ± 0.07 (0.295)	8.45 ± 0.07 (3.56)	101 ± 2
MPP-4CN-BUTINACA (**21**)	7.68 ± 0.13 (20.9)	8.06 ± 0.07 (8.72)	115 ± 3	8.42 ± 0.10 (3.80)	8.49 ± 0.06 (3.24)	100 ± 2
MMB-4CN-BUT7AICA (**22**)	5.82 ± 0.08 (1510)	6.37 ± 0.1 (430)	114 ± 7	6.50 ± 0.07 (316)	7.17 ± 0.08 (68.3)	95 ± 4
MDMB-4CN-BUT7AICA (**23**)	8.03 ± 0.13 (9.2)	7.99 ± 0.16 (10.3)	114 ± 5	8.61 ± 0.08 (2.4)	7.90 ± 0.16 (12.6)	106± 5
MPP-4CN-BUT7AICA (**24**)	6.42 ± 0.07 (380)	6.73 ± 0.08 (189)	110 ± 5	7.26 ± 0.06 (55.0)	7.84 ± 0.05 (14.3)	104 ± 2

^a^Data represent mean values ± standard error of the mean (SEM) from 3 to 6 independent experiments;

b Did not converge (DNC);

c Not determined (ND): < 50% change in fluorescence at 10 μM;

d Maximal response at 10 μM. AB, [(S)-2-amino-3-methylbutanamide]; ADB, [(S)-2-amino-3,3-dimethylbutanamide]; APP, [(S)-2-amino-3-phenylpropanamide]; MMB, [methyl (S)-2-amino-3-methylbutanoate]; MDMB, [methyl (S)-2-amino-3,3-dimethylbutanoate]; MPP, methyl [(S)-2-amino-3-phenylpropanoate]; 4-CN-BUT, 4-cyanobutyl; 4F-BUT, 4-fluorobutyl; ICA, indole-3-carboxamide; INACA, indazole-3-carboxamide; 7AICA, 7-aza-indole-3-carboxamide.

**Table 2:** Affinities and functional activities of 4-fluorobutyl derived SCRAs at hCB_1_ and hCB_2_.

**Compound**	**hCB**_**1**_			**hCB**_**2**_		
	**p***K*_i_ ±**SEM (***K*_i_**, nM)**	**pEC**_50_ ±**SEM (EC**_50_**, nM)**	**E**_max_ ±**SEM (% CP55,940)**	**p***K*_i_ ±**SEM (***K*_i_**, nM)**	**pEC**_50_ ±**SEM (EC**_50_**, nM)**	**E**_max_ ±**SEM (% CP55,940)**
AB-4F-BUTICA (**25**)	6.00 ± 0.11 (1,000)	7.02 ± 0.05 (96.5)	108 ± 3	7.09 ± 0.08 (82)	7.63 ± 0.05 (23.5)	97 ± 2
ADB-4F-BUTICA (**26**)	7.46 ± 0.08 (35)	7.39 ± 0.09 (40.4)	110 ± 4	8.10 ± 0.11 (8.0)	7.36 ± 0.12 (44.1)	105 ± 4
APP-4F-BUTICA (**27**)	5.50 ± 0.10 (3,200)	DNC^b^	82 ± 4^d^	6.76 ± 0.01 (170)	6.51 ± 0.16 (306)	108 ± 8
AB-4F-BUTINACA (**28**)	6.88 ± 0.13 (130)	8.22 ± 0.06 (5.98)	107 ± 2	8.07 ± 0.07 (8.5)	8.31 ± 0.04 (4.85)	96 ± 1
ADB-4F-BUTINACA (**29**)	8.39 ± 0.08 (4.07)	8.79 ± 0.06 (1.61)	116± 2	9.16 ± 0.04 (0.69)	8.28 ± 0.07 (5.28)	103 ± 2
APP-4F-BUTINACA (**30**)	5.86 ± 0.13 (1,380)	6.30 ± 0.05 (504)	103 ± 3	7.55 ± 0.15 (28.2)	8.08 ± 0.13 (8.35)	101 ± 12
AB-4F-BUT7AICA (**31**)	< 5	DNC^b^	82 ± 4^d^	5.86 ± 0.07 (1,400)	6.86 ± 0.05 (137)	98 ± 4
ADB-4F-BUT7AICA (**32**)	5.90 ± 0.06 (1,300)	7.34 ± 0.13 (46.0)	112 ± 6	7.34 ± 0.06 (46)	8.30 ± 0.04 (4.99)	108 ± 2
APP-4F-BUT7AICA (**33**)	< 5	ND^c^	25 ± 14^d^	5.62 ± 0.04 (2,400)	5.93 ± 0.16 (1,170)	99 ± 12
MMB-4F-BUTICA (**34**)	7.01 ± 0.10 (97.7)	7.11 ± 0.04 (76.9)	116 ± 3	7.57 ± 0.05 (26.9)	7.95 ± 0.05 (11.4)	102 ± 2
MDMB-4F-BUTICA (**7**)	7.88 ± 0.12 (13.2)	8.47 ± 0.08 (3.43)	114 ± 3	9.30 ± 0.12 (0.35)	8.54 ± 0.05 (2.87)	110 ± 3
MPP-4F-BUTICA (**35**)	6.57 ± 0.10 (270)	7.44 ± 0.06 (36.6)	105 ± 2	8.22 ± 0.06 (6.1)	8.00 ± 0.05 (10.1)	100 ± 2
MMB-4F-BUTINACA (**36**)	7.50 ± 0.15 (32)	8.41 ± 0.08 (3.93)	103 ± 2	8.81 ± 0.09 (1.6)	8.42 ± 0.08 (3.76)	100 ± 2
MDMB-4F-BUTINACA (**8**)	8.21 ± 0.13 (6.2)	9.39 ± 0.17 (0.41)	106 ± 3	9.92 ± 0.09 (0.10)	8.48 ± 0.14 (3.28)	103 ± 5
MPP-4F-BUTINACA (**37**)	7.47 ± 0.13 (34)	8.25 ± 0.12 (5.62)	106 ± 3	9.34 ± 0.05 (0.50)	8.33 ± 0.10 (4.65)	101 ± 3
MMB-4F-BUT7AICA (**38**)	6.65 ± 0.05 (220)	5.86 ± 0.18 (1,390)	115 ± 16	7.22 ± 0.02 (61)	7.30 ± 0.13 (49.6)	114 ± 6
MDMB-4F-BUT7AICA (**39**)	6.97 ± 0.08 (107)	7.65 ± 0.08 (22.2)	109 ± 3	8.67 ± 0.07 (2.14)	8.41 ± 0.08 (3.88)	95 ± 2
MPP-4F-BUT7AICA (**40**)	6.06 ± 0.13 (880)	6.20 ± 0.14 (637)	122 ± 10	7.54 ± 0.08 (29)	7.57 ± 0. 17 (26.9)	110 ± 6

^a^Data represent mean values ± standard error of the mean (SEM) from 3 to 6 independent experiments;

b Did not converge (DNC);

c Not determined (ND): < 50% change in fluorescence at 10 μM;

d Maximal response at 10 μM. AB, [(S)-2-amino-3-methylbutanamide]; ADB, [(S)-2-amino-3,3-dimethylbutanamide]; APP, [(S)-2-amino-3-phenylpropanamide]; MMB, [methyl (S)-2-amino-3-methylbutanoate]; MDMB, [methyl (S)-2-amino-3,3-dimethylbutanoate]; MPP, methyl [(S)-2-amino-3-phenylpropanoate]; 4-CN-BUT, 4-cyanobutyl; 4F-BUT, 4-fluorobutyl; ICA, indole-3-carboxamide; INACA, indazole-3-carboxamide; 7AICA, 7-aza-indole-3-carboxamide.

### *In vitro* functional evaluation

Mouse AtT20 FlpIn adenocarcinoma cells stably transfected with human CB_1_ or CB_2_ were cultured in DMEM containing 10% FBS, 100 U penicillin/streptomycin, and 80 μg/mL of hygromycin, as previously described (54). Cells were passaged at 80% confluency, cells for assays were grown in 75 cm^2^ flasks and used at 90% confluence. The day before the assay cells were detached from the flask with trypsin/EDTA (Sigma Aldrich) and resuspended in 10 mL of Leibovitz's L-15 media (L-15) supplemented with 1% FBS, 100 U penicillin/streptomycin, and 15 mM glucose. The cells were plated in volume of 90 μL in black walled, clear bottomed 96-well microplates (Corning, Corning, NY, USA) and incubated overnight at 37°C in ambient CO_2_. Membrane potential was measured using a Membrane Potential Assay Kit (blue) from Molecular Devices (San Jose, CA, USA), as described previously (55, 56). The dye was reconstituted with assay buffer [145 mM NaCl, 22 mM HEPES, 0.338 mM Na_2_HPO_4_, 4.17 mM NaHCO_3_, 0.441 mM KH_2_PO_4_, 0.407 mM MgSO_4_, 0.493 mM MgCl_2_, 1.26 mM CaCl_2_, 5.56 mM glucose (pH 7.4)]. Prior to the assay, cells were loaded with 90 μL/well of the dye solution without removal of the L-15. Plates were then incubated at 37°C in ambient CO_2_ for 60 min. Fluorescence was measured using a FlexStation 3 (Molecular Devices) microplate reader with cells excited at a wavelength of 530 nm and emission measured at 565 nm. Baseline readings were taken every 2 s for at least 2 min, at which time either drug or vehicle was added in a volume of 20 μL. The background fluorescence of cells without dye or dye without cells was negligible. Changes in fluorescence were expressed as a percentage of baseline fluorescence after subtraction of the changes produced by vehicle (DMSO, final concentration was no more than 0.1%) addition. Concentration-response data were analyzed using GraphPad Prism 9 (GraphPad Software, San Diego, CA, USA), using a four-parameter non-linear regression to fit concentration-response curves on data from 3 to 6 independent experiments.

### Molecular modeling

#### Protein preparation

The cryo-EM structures of the CB_1_ receptor (PDB: 6N4B) and the CB_2_ receptor (PDB: 6KPF) were retrieved from RCSB PDB (57–59). The structures were prepared with Maestro's Protein Preparation Wizard as follows (60). The G proteins and cholesterol were removed, leaving only the CB_1_ and CB_2_ receptor and their cognate ligands. The preparation process consisted of assigning bond orders, adding hydrogens, generation of disulfide bonds, generation of missing side chains using Prime, generating het states using Epik at pH 7.4 ± 1.0, and deleting water molecules beyond 3 Å from het groups (61, 62). The hydrogen bonding network was optimized, the p*K*_a_ values of the protein were predicted using PROPKA, and target pH value was set at 7.4 (63). Lastly, the protein structure was minimized using the OPLS4 force field where RMSD of the atom displacement for terminating the minimization was set as 0.3 Å (64).

#### Ligand preparation

Ligands were prepared using LigPrep to generate energy minimized 3D structures (65). OPLS4 force field was used for minimization. Epik was used to generate all possible ionized states at pH 7.4 ± 1.0.

#### Ligand docking

A receptor grid was generated using Glide, with a van der Waals radius scaling factor of 1.0 and a partial charge cutoff at 0.25 (66). The binding site was defined by the centroid of the cognate ligand for each structure. The Van der Waals scaling factor for the ligands was set to 0.80 with a partial charge cut-off at 0.15. The precision was set to Extra Precision (XP) with flexible ligand sampling. Nitrogen inversions and ring conformations were sampled and Epik state penalties were added to the docking scores. The ligand cores were restricted to the position of the cognate ligand by their maximum common substructure and post docking minimization was performed. Poses that had the lowest RMSD between the 4-fluorobutyl and 4-cyanobutyl analogs were taken for direct comparison.

#### Strain calculations

The internal strain of ligands in their bound and unbound states was calculated using MacroModel (65). The 4RDDD solvation forcefield was used, a constant 4.00 kcal/mol energy offset selected, a penalty scale factor of 0.25 and Cartesian restraints with a bound state half-width of 0.3 Å and a 120 kcal/mol/Å^2^ force constant.

#### Binding site mapping

The binding site regions for both the CB_1_ and CB_2_ structures were evaluated using SiteMap (67). SiteMap was run in evaluate mode using the structures cognate ligand and a 5 Å buffer. A minimum of 15 site points per binding site was set, using the “more restrictive” definition of hydrophobicity and a standard grid. Site maps were cropped at 4 Å from the nearest site point.

## Results and discussion

### Synthesis

**Scheme 1:**
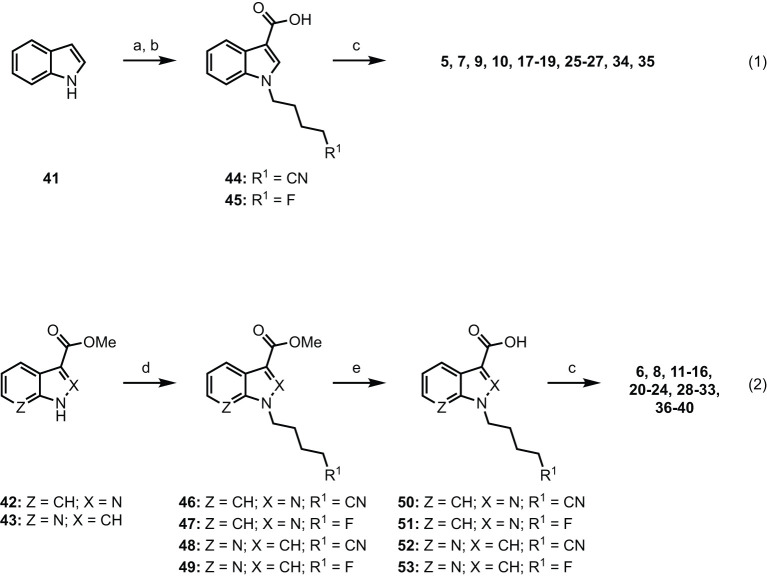
Reagents and conditions: **(a)** (i) NaH, alkylbromide, DMF, 0 °C–rt, 1 h; (ii) (CF_3_CO)_2_O, 0 °C–rt, 2 h; **(b)** KOH, MeOH, PhMe, reflux, 2 h; **(c)** EDC•HCl, HOBt•H_2_O, amino acid•HCl, Et_3_N, DMF, rt, 18 h, 29–99%. **(d)** NaH, alkylbromide, DMF, 0°C–rt, 24 h, 81%; **(e)** 1 M aq. NaOH, MeOH, rt, 48 h, 98%.

Indole-based SCRAs were derived from indole **(41)**, with a convenient one-pot alkylation and trifluoracetylation procedure employed to give intermediate *N*-alkyl-5-(3-(2,2,2-trifluoroacetyl)-1*H*-indoles before subsequent hydrolysis gave *N*-alkyl-1*H*-indole-3-carboxylic acids **44** and **45** in good yield (Scheme 1, eq. 1). This was then subjected to 1-ethyl-3-(3-dimethylaminopropyl)carbodiimide (EDC)/1-hydroxybenzotriazole (HOBt)-mediated amide coupling with the appropriate amino acid derivatives to furnish the final indole derived compounds (**5, 7, 9, 10, 17–19, 25–27, 34, 35**). The indazole and 7-azaindole derivatives were synthesized using a similar sequence from methyl indazole- or methyl-7-azaindole-3 carboxylates **42** and **43** (Scheme 1, eq. 2). Thus, alkylation with the appropriate alkylbromide and sodium hydride provided methyl *N*-alkyl-indazole- or methyl *N*-alkyl-7-azaindole-3 carboxylates **46–49** before hydrolysis to give the corresponding carboxylic acids 50–53. In the same manner as described previously, EDC/HOBt-mediated amide coupling furnished *N*-alkyl-indazole- or -alkyl-7-azaindole-3-carboxylamides **6, 8, 11–16, 20–24, 28–33, 36–40** (6, 50, 51, 54, 68, 69). Synthetic procedures and characterization data (^1^H and ^13^C NMR, melting point, R_*f*_, FTIR, HRMS, and LCMS) for all novel compounds can be found in the supporting information.

### *In vitro* binding affinity trends for 5–40

Following our previous efforts in the characterization of existing SCRAs, *in vitro* binding data were obtained *via* a competitive radio binding assay in HEK293 cells stably transfected with human cannabinoid receptor 1 (hCB_1_) or human cannabinoid receptor 2 (hCB_2_) (6, 50, 54, 68, 69). Ligand affinity (p*K*_i_) was determined based on extent to which compounds displaced the tritiated standard [^3^H]CP55,940 (Tables 1, 2). Except for terminal amide bearing 7-azaindoles SCRAs **38, 33** and **16**, all compounds displayed micromolar to sub-nanomolar affinity for CB_1_ receptors (p*K*_i_ = 8.89 ± 0.09–5.48 ± 0.11). Similar or increased CB_2_ affinity was observed in almost all cases (p*K*_i_ = 9.92 ± 0.09–5.49 ± 0.03), with several examples exhibiting sub-nanomolar affinities for this receptor subtype (**20**, p*K*_i_ = 9.53 ± 0.07; **29**, p*K*_i_ = 9.16 ± 0.04; 7, p*K*_i_ = 9.30 ± 0.12; **8**, 9.92 ± 0.09; **37**, p*K*_i_ = 9.34 ± 0.05), which represent the highest affinities in the series. Across the 4-cyanobutyl and 4-fluorobutyl tail substituents, no clear trend was apparent for CB_1_ except within methyl *tert-*leucinate (**18, 20, 23** > **7, 8, 39**) and methyl phenylalaninate (**35, 37, 40** **>** **19, 21, 24**) subgroups, whereby 4-cyanobutyl and 4-fluorobutyl substitution, respectively, conveyed increased affinity, albeit slightly. Only 4-cyanobutyl bearing **15** (CB_1_ p*K*_i_ = 6.81 ± 0.09) showed affinity an order of magnitude greater than its 4-fluorobutyl analog **32** (CB_1_ p*K*_i_ = 5.90 ± 0.06). Conversely, 4-fluorobutyl derivatives were in general equal or better ligands at CB_2_. Variation of the headgroup within the indazole series (AB: **28** **>** **11; ADB: 29** **>** **12; APP: 30** **>** **13; MMB: 36** **>** **4; MDMB: 8** **≈20; MMP: 37** **≈21**) highlights this trend. A clear structure-affinity trend within the core scaffold was observed, with indazoles (CB_1_, p*K*_i_ = 8.89 ± 0.09–5.48 ± 0.11; CB_2_, p*K*_i_ = 9.53 ± 0.07–6.78 ± 0.03) providing the best affinity to both CB_1_ and CB_2_, followed closely by indoles (CB_1_, p*K*_i_ = 8.18 ± 0.11–5.50 ± 0.10; CB_2_, p*K*_i_ = 9.30 ± 0.12–6.22 ± 0.13). Finally, 7-azaindoles (CB_1_, p*K*_i_ = 8.03 ± 0.13– < 5; CB_2_, p*K*_i_ = 8.67 ± 0.07–5.49 ± 0.03) exhibited reduced affinity compared with the other groups, often by an order of magnitude. This is in keeping with previous studies detailing related indole, indazole and 7-azaindole carboxamides (36, 51, 54). Notably, the indazole core was required to achieve sub-nanomolar affinity for CB_2_ (i.e., **20, 29, 8, 37**) except in the case of 7, whereby high affinity was maintained with combination of an indole core, and importantly, the methyl *tert*-leucinate head group. In each core and tail sub-group, the *tert-*leucine derivatives (MDMB > ADB) were consistently the best ligands for both CB_1_ and CB_2_, followed by phenylalanine methyl esters (MPP), which were better than both valine derivatives (MMB > AB), and finally, phenylalaninamides (APP). For example, within the indole set, a *tert*-leucinate conferred the highest CB_1_ affinity (**18**, p*K*_i_ = 8.18 ± 0.11), which was reduced by an order of magnitude for the corresponding phenylalaninate (**19**, p*K*_i_ = 7.07 ± 0.09), and further halved for valine derivatives (**17**, p*K*_i_ = 6.80 ± 0.14). An alternate order was observed for the corresponding terminal amides of *tert-*leucine (**9**, p*K*_i_ = 8.17 ± 0.12), valine (**5**, p*K*_i_ = 6.26 ± 0.09) and phenylalanine (**10**, p*K*_i_ = 5.48 ± 0.11), which conferred one of the lowest affinities in the set. Affinities for CB_2_ followed the same general trend, although phenylalaninamides exhibited affinities up to an order of magnitude greater compared to CB_1_ (e.g., **13**, CB_1_ p*K*_i_ = 5.48 ± 0.11; CB_2_ p*K*_i_ = 6.78 ± 0.03). Indeed, APP derivatives were not well-tolerated at CB_1_ and thus modest selectivity for CB_2_ (4- to 50-fold) was observed in this class (i.e., **10, 33, 16, 27, 30**).

### *In vitro* CB_1_ and CB_2_ functional characterization of 5–40

**Figure 4:**
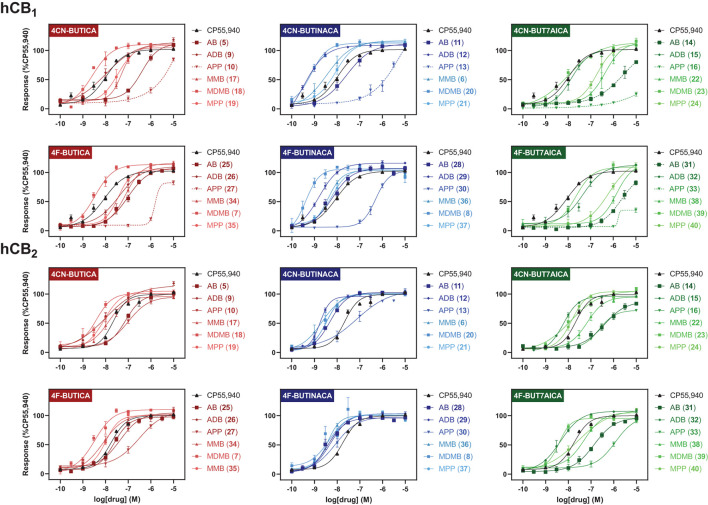
Functional activities of compounds **5–40** at hCB_1_ and hCB_2_, relative to CP55,940. Efficacies are normalised to maximal response of 1 μM CP55,940. Vertical sets indicate the same heterocyclic core (left = indole; centre = indazole; right = 7-azaindole). Graphs are grouped by tail, with rows indicating alkyl tails, either 4-cyanobutyl (rows 1 and 3), or 4-fluorobutyl (rows 2 and 4). Dashed lines indicate where curve could not be fit. Data is expressed as means ± SEM from 3 to 6 independent experiments.

SARs for compounds **5–40** were also investigated *via* a fluorescence-based functional assay using AtT20 cells transfected with hCB_1_ and hCB_2_. This method measures change in membrane potential resulting from G_βγ_*-*coupled activation of inwardly rectifying potassium channels (GIRKs). The full concentration-response curves of **5–40** are shown in Figure 4, with potencies and efficacies referenced to 1 μM CP55,940 in Tables 1, 2. Most compounds, except for selected phenylalaninamide and/or 7-azaindole derivatives (**10, 13, 16, 27, 33**, and **38**), had maximal efficacy (E_max_ = 102–122%), and exhibited nanomolar to sub-nanomolar potencies (pEC_50_ = 9.48 ± 0.14–6.30 ± 0.05) at CB_1_. In general, this group activated CB_2_ with equal or increased potency (pEC_50_ = 8.67 ± 0.17–6.41 ± 0.10) and efficacy, albeit, with no ligands active at sub-nanomolar levels. As with other SCRAs, these compounds are generally similar or greater potency, but more efficacious than THC (pEC_50_ = 6.76 ± 0.09 and E_max_ = 58% ± 3% at CB_1_, and 32% ± 1% at 30 μM at CB_2_) (36). Potency compared with THC is also similar (pK_i_ = 8.09 ± 0.02 and 7.50 ± 0.07 at CB_1_ and CB_2_, respectively) (70).

### Effect of 4-cyanobutyl and 4-fluorobutyl substituents on CB_1_ and CB_2_ activation

Of the 3 chemical modifications investigated in the present study, variation of the tail substituent had the least significant impact on potency and efficacy. The 4-cyanobutyl and 4-fluorobutyl derivatives commonly displayed similar potency at both CB_1_ and CB_2_, for example in the *tert*-leucinate subgroup (**18, 20, 23** **≈7, 8, 39**, respectively), or conversely, exhibited differing potency with no discernable preference for either tail group. Indeed, both tail substituents produced active ligands with sub-nanomolar potency for CB_1_ (**20**: pEC_50_ = 9.14 ± 0.14; **8**: pEC_50_ = 9.39 ± 0.17) and low nanomolar potency for CB_2_ (20: pEC_50_ = 8.45 ± 0.07; **8**: pEC_50_ = 8.48 ± 0.14). These data are consistent with the corresponding structure-affinity trends; however, binding is not predictive of relative potency in some cases. For example, MDMB-4F-BUTINACA **(8)** exhibits activation (pEC_50_ = 9.39 ± 0.17) an order of magnitude greater than its affinity (p*K*_i_ = 8.21 ± 0.13) for CB_1_, whilst for CB_2_, the opposite is true (pEC_50_ = 8.48 ± 0.14; p*K*_i_ = 9.92 ± 0.09).

### Effect of indole, indazole and 7-azaindole core on CB_1_ and CB_2_ activation

In the present study, the nature of the heterocyclic core greatly impacts potency, giving rise to the same trends as previously documented (51, 54, 71). Rank order potency of activity at CB_1_ closely mirrored the observed binding affinities, whereby indazoles (pEC_50_ = 9.39 ± 0.17–6.31 ± 0.05) demonstrated equal or greater potency to the corresponding indoles (pEC_50_ = 8.57 ± 0.13– < 5), which were, again, more potent than the corresponding 7-azaindoles (pEC_50_ = 8.05 ± 0.21– < 5). In all cases, the indazole core was required to produce sub-nanomolar potency (pEC_50_ = 9.39 ± 0.17, 9.48 ± 0.14, 9.14 ± 0.14) observed for compounds **8, 12**, and **20**, respectively. While the same general trend is realized for activation of CB_2_ (indazoles: pEC_50_ = 8.75 ± 0.03–7.00 ± 0.36; indoles: pEC_50_ = 8.54 ± 0.05–6.49 ± 0.2; 7-azaindoles: pEC_50_ = 8.41 ± 0.08–5.92 ± 0.16), differentiation of heterocyclic cores is less pronounced for this receptor, with the MPP subgroup (**21** **>** **19** **>** **24**) highlighting this effect. Notably, this is not apparent for MDMB derivatives (**7** **≈8** **≈39**), whereby all core motifs conferred similar potency.

### Effect of amino acid derived head groups on CB_1_ and CB_2_ activation

As described previously, *tert-*leucine derived compounds (MDMB/ADB) are commonly more potent than the corresponding valine (ADB/AB) species at CB_1_, despite differing only by a single methyl group (36, 51, 54, 69, 71, 72). In line with this is the greater potencies of MDMB/ADB vs. MMB/AB analogs for indole (**18, 9** **>** **5, 17**), indazole (**20, 12** **>** **6, 11**) and 7-azaindole (**23, 15** **>** **22, 14**) sub-groups observed here. Further, MDMB- and ADB-4F-BUTINACA (**8** and **29**) represent two of only three substrates with sub-nanomolar potency, and indeed the highest potencies observed for CB_1_. While the methyl esters of phenylalanine (MPP) were commonly equipotent or greater than valine (AB and MMB) analogs, unlike with *tert*-leucine derivatives, switching to the terminal amide (APP) was detrimental to potency at CB_1_. Specifically, when combined with an indole (**10** and **27**) or 7-azaindole core (**16** and **33**), the APP substituent produced submaximal agonist activity at the highest concentration tested (10 μM), whereas full efficacy was maintained with the indazole core (i.e., **13**: E_max_ = 104%). In terms of CB_2_, structure-activity trends align closely with CB_1_; however, with all phenylalaninamides (APP) except APP-4CN-BUT7AICA (**16**: E_max_ = 73%, pEC_50_ = 6.63 ± 0.11) having full agonist effect with moderate potency. As with binding, these findings indicate a CB_2_ receptor subtype selectivity for the phenylalaninamide group. In general, the SARs described here corroborate the observed structure-affinity trends for the compounds in the present study, as well as those previously found (54).

### *In silico* molecular docking study

To rationalize the novel SARs observed *in vitro* at CB_1_ and CB_2_, we performed docking for each of the screened ligands using the cryo-EM structures of the CB_1_ (PDB ID: 6N4B) and the CB_2_ (PDB ID: 6PT0) receptors retrieved from the Research Collaboratory for Structural Bioinformatics Protein Databank (RCSB PDB) (57–59). These agonist-bound structures were chosen for the similarity between our compounds and the cognate ligands MDMB-FUBINACA (6N4B) and WIN 55,212-2 (6PT0).

When docking at CB_1_ there were π-π interactions between each of the ligands and residues PHE200 and PHE268. The flexible 4-fluorobutyl and 4-cyanobutyl tail groups occupied a hydrophobic pocket comprised of MET363, LEU276, TYR275, LEU193, and ILE271. There is also a common polar interaction between the amide linker carbonyl and SER383, as well as the terminal carbonyl and HIE178.

**Figure 5:**
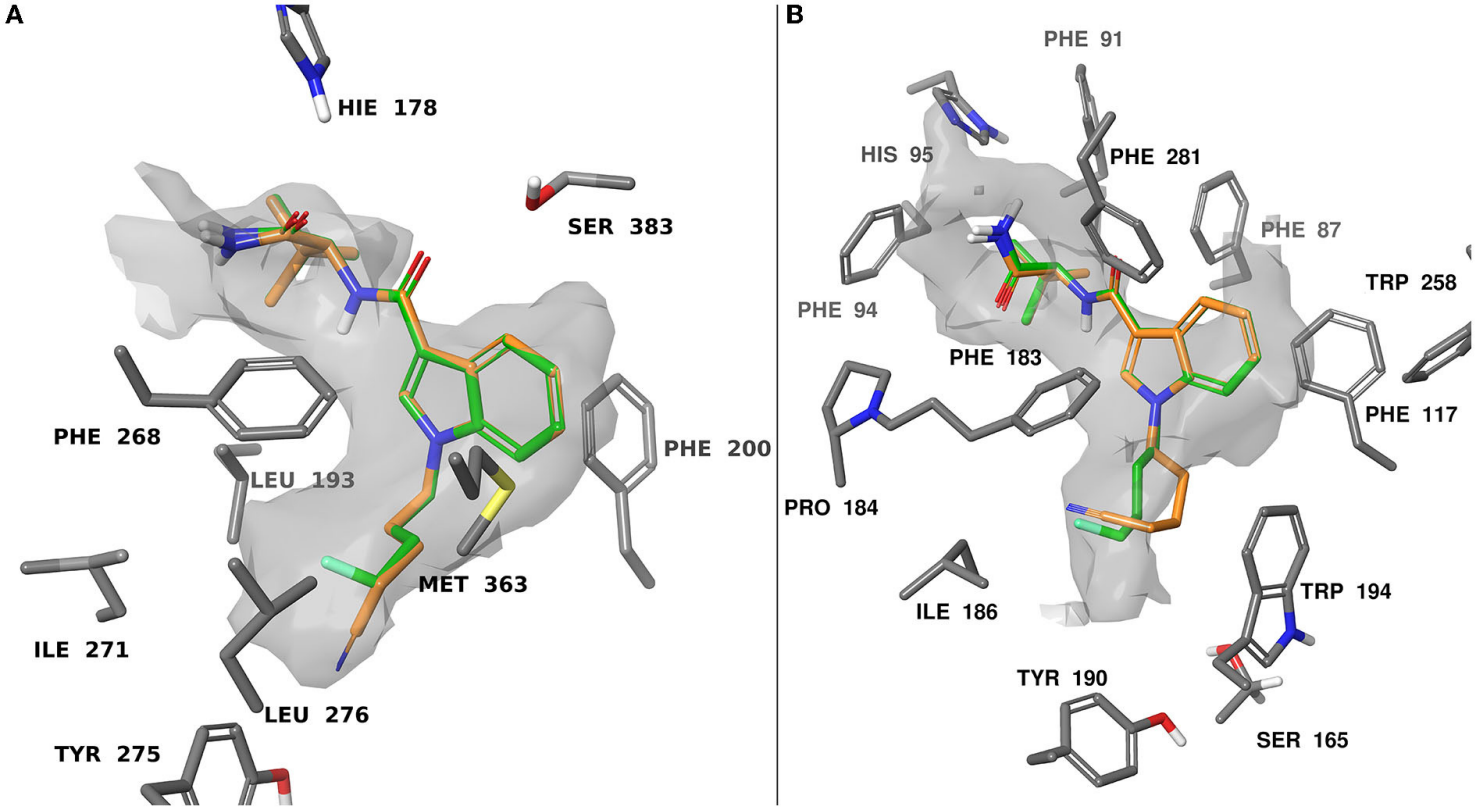
Computationally predicted binding modes of **9** (orange) and **26** (green) overlayed at CB_1_ **(A)**, PDB:6N4B and CB_2_ **(B)**, PDB:6PT0 (57, 58). The hydrophobic regions of the binding site (grey) were evaluated with SiteMap (67). Glide docking was performed using the Schrodinger computational chemistry suite (66).

Docking at CB_2_ was dictated by hydrophobic interactions between the core and PHE117, and PHE183 residues. The flexible tail occupied a pocket comprised of TRP194, ILE186, TYR190, and SER165. The amide and ester head groups occupied a pocket comprised of PHE91, PHE94, HIS95, and PRO184. Ligands bearing a terminal amide had the potential for H-bonding with the backbone carbonyl of PHE183. Pairs of docking poses for ligands that differed by tail substitution were selected for strain rescoring (see SI, figures S105–116 for all calculated docking poses). This allowed us to investigate the SAR described in the *in vitro* section, wherein many 4-fluorobutyl ligands showed higher affinity for the CB_2_ receptor than their 4-cyanobutyl counterparts. Given the lack of formal intermolecular interactions possible for this substituent, the effect is likely due to the size-constraints of the binding pocket for CB_2_ as compared with CB_1_. Figure 5 shows an overlay of compounds **9** and **26** at both CB_1_ and CB_2_, as well as the computed hydrophobic regions of the binding site. Relative to CB_1_, the CB_2_ binding site region occupied by the 4-fluorobutyl or 4-cyanobutyl tail is significantly constrained. This was observed by Sitemap calculation on the active site of CB_1_ and CB_2_ receptors as illustrated in Figure 5. This may force the longer and more rigid 4-cyanobutyl tail into a higher strain conformation to adopt the predicted binding pose. The strain energy of the ligands in their bound state relative to their unbound state was calculated, and the values compared for analogous ligands (see SI Table T1). The mean difference and standard error between the docked 4-cyanobutyl and 4-fluorobutyl ligands were 0.220 ± 0.400 kcal/mol at CB_1_ and 2.552 ± 0.468 kcal/mol at CB_2_.

## Conclusions

The detected SCRAs AB-4CN-BUTICA, MMB-4CN-BUTINACA, MDMB-4F-BUTICA, MDMB-4F-BUTINACA, as well as a series of 32 analogs, were synthesized, characterized, and evaluated *in vitro* using a radioligand binding assay and a functional membrane potential assay at both human CB_1_ and CB_2_. These data confirm that AB-4CN-BUTICA, MMB-4CN-BUTINACA, MDMB-4F-BUTICA, MDMB-4F-BUTINACA are potent and efficacious cannabinoid receptor ligands. Most analogs in the present study, barring the APP (phenylalaninamide) derivatives, were also potent and efficacious cannabinoid ligands. SAR trends observed here were consistent with those previously described for with respect to head group and core group contributions to ligand activity (30, 36, 50, 54, 69, 72). The increased affinity for 4-fluorobutyl derivatives at CB_2_ is likely to arise from increased strain of the 4-cyanobutyl tail in the hydrophobic tail pocket. Given these pharmacological data, availability of precursory chemical building blocks, ease of synthesis, and structural similarity to previous SCRAs, the compounds evaluated in this study should be monitored as potential emerging NPS in the marketplace.

## Data availability statement

The original contributions presented in the study are included in the article/Supplementary material, further inquiries can be directed to the corresponding author/s.

## Author contributions

ES and JL synthesized and characterized compounds under the supervision of AA and SB. *In vitro* binding data were obtained by SC under the supervision of MG. *In vitro* functional characterization was performed by RB and CF under the supervision of EC and by TF and HZ under the supervision of MS and MC. *In silico* docking experiments were performed by JM under the supervision of FL and DH. HRMS data were obtained by RE under the supervision of RG. ES, JM, IM, EC, MC, MG, AA, and SB conceived the experiments. ES, JM, EC, AA, and SB wrote the manuscript. All authors reviewed drafts of the manuscript and approved the final version.

## Funding

Parts of this work were supported NHMRC Project Grant 1161571 and Brain and Mind Centre Research Development Grant awarded to SB. ES, RB, JM, CF, IM, EC, AA, and SB are supported by the Lambert Initiative for Cannabinoid Therapeutics, a philanthropic research program based at the University of Sydney. ES, JM, and TF are supported by an Australian Government Research Training Scholarships through the University of Sydney and Macquarie University respectively.

## Conflict of interest

The authors declare that the research was conducted in the absence of any commercial or financial relationships that could be construed as a potential conflict of interest.

## Publisher's note

All claims expressed in this article are solely those of the authors and do not necessarily represent those of their affiliated organizations, or those of the publisher, the editors and the reviewers. Any product that may be evaluated in this article, or claim that may be made by its manufacturer, is not guaranteed or endorsed by the publisher.

## Glossary

[^3^H]CP55,940, Tritiated 2-((1*R*,2*R*,5*R*)-5-hydroxy-2-(3-hydroxypropyl)cyclohexyl)-5-(2-methyloctan-2-yl)phenol

AB-4CN-BUTICA, (*S*)-*N*-(1-amino-3-methyl-1-oxobutan-2-yl)-1-(4-cyanobutyl)-1*H*-indole-3-carboxamide

ADB-BUTINACA, (*S*)-*N*-(1-amino-3,3-dimethyl-1-oxobutan-2-yl)-1-butyl-1*H*-indazole-3-carboxamide

AtT20, Mouse AtT20 FlpIn adenocarcinoma cells

BSA, Bovine serum albumin

CB_1_, Cannabinoid receptor 1

CB_2_, Cannabinoid receptor 2

CD_3_OD, Deuterated methanol (*d*_4_)

CDCl_3_, Deuterated chloroform

CH_2_Cl_2_, dichloromethane

CP55,940, 2-((1*R*,2*R*,5*R*)-5-hydroxy-2-(3-hydroxypropyl)cyclohexyl)-5-(2-methyloctan-2-yl)phenol

cryo-EM, Cryogenic electron microscopy

DMEM, Dulbecco's modified eagle medium

DMF, Dimethylformamide

DMSO, Dimethyl sulfoxide

DMSO-*d*_6_, Deuterated dimethyl sulfoxide (*d*_6_)

DNC, Did not converge

EC_50_, Half maximal effective concentration

EDC, 1-ethyl-3-(3-dimethylaminopropyl)carbodiimide

EDTA, Ethylenediaminetetraacetic acid

E_max_, Maximum effect

Et_3_N, Triethylamine

EtOAc, Ethyl acetate

FBS, Fetal bovine serum

FTIR, Fourier-transform infrared spectroscopy

G_βγ_, G beta−gamma subunit

GIRK, G protein−coupled inwardly rectifying potassium channel HA, Haemagglutin in

hCB_1_, Human cannabinoid receptor 1

hCB_2_, Human cannabinoid receptor 2

HEK, Human embryonic kidney cells

HEPES, 2-[4-(2-hydroxyethyl)piperazin-1-yl]ethane-1-sulfonic acid

HOBt, 1-hydroxybenzotriazole

HRMS, High resolution mass spectrometry

JWH-018, Naphthalen-1-yl(1-pentyl-1*H*-indol-3-yl)methanone

K_d_, Equilibrium dissociation constant

K_i_, Inhibition constant

LC-UV, Liquid chromatography- Ultraviolet

m.p., Melting point

MDMB-4F-BUTICA, Methyl (*S*)-2-(1-(4-fluorobutyl)-1*H*-indole-3-carboxamido)-3,3-dimethylbutanoate

MDMB-4F-BUTINACA, Methyl (*S*)-2-(1-(4-fluorobutyl)-1*H*-indazole-3-carboxamido)-3,3-dimethylbutanoate

MDMB-FUBINACA, Methyl (*S*)-2-(1-(4-fluorobenzyl)-1*H*-indazole-3-carboxamido)-3,3-dimethylbutanoate

MeOH, Methanol

MgSO_4_, Magnesium sulfate

MMB-4CN-BUTINACA, a.k.a. AMB-4CN-BUTINACA, methyl (1-(4-cyanobutyl)-1*H*-indazole-3-carbonyl)-L-valinate

MS, Mass spectrometry

Na_2_SO_4_, Sodium sulfate

NaH, Sodium hydride

ND, Not determined

NMR, Nuclear magnetic resonance

NPS, New psychoactive substance

PBS, Phosphate buffered saline

PEI, Branched polyethyleneimine

pplss, Preprolactin signal sequence

RCSB PDB, Research Collaboratory for Structural Bioinformatics Protein Databank

RMSD, Root-mean-square deviation

SARs, Structure activity relationships

SCRA, Synthetic cannabinoid receptor agonist

SEM, Standard error of the mean

THC, Δ^9^-tetrahydrocannabinol

XP, Extra Precision
