# Supplementary material for: Synthesis and pharmacological evaluation of newly detected synthetic cannabinoid receptor agonists AB-4CN-BUTICA, MMB-4CN-BUTINACA, MDMB-4F-BUTICA, MDMB-4F-BUTINACA and their analogs
Source: Front Psychiatry. 2022 Sep 28;13:1010501. doi: 10.3389/fpsyt.2022.1010501 (PMC9558907; doi:10.3389/fpsyt.2022.1010501)
Supplement: Supplementary file 1 [file Data_Sheet_1.docx]

**Supporting Information for**

**Synthesis and pharmacological evaluation of newly detected synthetic cannabinoid receptor agonists AB-4CN-BUTICA, MMB-4CN-BUTINACA, MDMB-4F-BUTICA, MDMB-4F-BUTINACA and their analogues**

Eric Sparkes,^1,2^ Rochelle Boyd,^1,3^ Shuli Chen,^4^ Jack W. Markham,^1,2,5^ Jia Lin Luo,^1,3^ Tahira Foyzun,^6^ Humayra Zaman,^6^ Charlotte Fletcher,^1,3^ Ross Ellison,^7^ Iain S. McGregor,^1,3^ Marina J. Santiago,^6^ Felcia Lai,^5^ Roy R. Gerona,^7^ Mark Connor,^6^ David E. Hibbs,^5^ Elizabeth A. Cairns,^1,3^ Michelle Glass,^4^ Adam Ametovski,^1,2†^ Samuel D. Banister^1,2†^

^1^The Lambert Initiative for Cannabinoid Therapeutics, Brain and Mind Centre, The University of Sydney, NSW 2050, Australia;

^2^School of Chemistry, Faculty of Science, The University of Sydney, NSW 2006, Australia;

^3^School of Psychology, Faculty of Science, The University of Sydney, NSW 2006, Australia;

^4^Department of Pharmacology and Toxicology, University of Otago, Dunedin 9016, New Zealand

^5^School of Pharmacy, Faculty of Medicine and Health, The University of Sydney, NSW 2006, Australia;

^6^Macquarie Medical School, Macquarie University, Sydney, NSW 2109, Australia;

^7^Clinical Toxicology and Environmental Biomonitoring Laboratory, University of California, San Francisco, CA 94143, USA;

Contents

[**Synthesis and characterization for newly described compounds** 1](#_Toc112063810)

[**General procedure A: amidation of 1-alkylindole-3-carboxyic acids, 1-alkyl-1*H*-indazole-3-carboxylic acids and 1-alkyl-1*H*-pyrrolo[2,3-*b*]pyridine-3-carboxylic acid.** 1](#_Toc112063811)

[(*S*)-*N*-(1-amino-3-methyl-1-oxobutan-2-yl)-1-(4-cyanobutyl)-1*H*-indole-3-carboxamide (5, AB-4CN-BUTICA). 1](#_Toc112063812)

[(*S*)-*N*-(1-amino-3,3-dimethyl-1-oxobutan-2-yl)-1-(4-cyanobutyl)-1*H*-indole-3-carboxamide (9, ADB-4CN-BUTICA). 2](#_Toc112063813)

[(*S*)-*N*-(1-amino-1-oxo-3-phenylpropan-2-yl)-1-(4-cyanobutyl)-1*H*-indole-3-carboxamide (10, APP-4CN-BUTICA). 2](#_Toc112063814)

[(*S*)-*N*-(1-amino-3,3-dimethyl-1-oxobutan-2-yl)-1-(4-cyanobutyl)-1*H*-indazole-3-carboxamide (12, ADB-4CN-BUTINACA). 3](#_Toc112063815)

[(*S*)-*N*-(1-amino-1-oxo-3-phenylpropan-2-yl)-1-(4-cyanobutyl)-1*H*-indazole-3-carboxamide (13, APP-4CN-BUTINACA). 3](#_Toc112063816)

[(*S*)-*N*-(1-amino-3-methyl-1-oxobutan-2-yl)-1-(4-cyanobutyl)-1*H*-pyrrolo[2,3-*b*]pyridine-3-carboxamide (14, AB-4CN-BUT7AICA). 4](#_Toc112063817)

[(*S*)-*N*-(1-amino-3,3-dimethyl-1-oxobutan-2-yl)-1-(4-cyanobutyl)-1*H*-pyrrolo[2,3-*b*]pyridine-3-carboxamide (15, ADB-4CN-BUT7AICA). 4](#_Toc112063818)

[(*S*)-*N*-(1-amino-1-oxo-3-phenylpropan-2-yl)-1-(4-cyanobutyl)-1*H*-pyrrolo[2,3-*b*]pyridine-3-carboxamide (16, APP-4CN-BUT7AICA). 5](#_Toc112063819)

[methyl (*S*)-2-(1-(4-cyanobutyl)-1*H*-indole-3-carboxamido)-3,3-dimethylbutanoate (18, MDMB-4CN-BUTICA). 5](#_Toc112063820)

[methyl (1-(4-cyanobutyl)-1*H*-indole-3-carbonyl)-*L*-phenylalaninate (19, MPP-4CN-BUTICA). 6](#_Toc112063821)

[methyl (*S*)-2-(1-(4-cyanobutyl)-1*H*-indazole-3-carboxamido)-3,3-dimethylbutanoate (20, MDMB-4CN-BUTINACA). 7](#_Toc112063822)

[methyl (1-(4-cyanobutyl)-1*H*-indazole-3-carbonyl)-*L*-phenylalaninate (21, MPP-4CN-BUTINACA). 7](#_Toc112063823)

[methyl (*S*)-2-(1-(4-cyanobutyl)-1*H*-pyrrolo[2,3-*b*]pyridine-3-carboxamido)-3,3-dimethylbutanoate (23, MDMB-4CN-BUT7AICA). 8](#_Toc112063824)

[methyl (1-(4-cyanobutyl)-1*H*-pyrrolo[2,3-*b*]pyridine-3-carbonyl)-*L*-phenylalaninate (24, MPP-4CN-BUT7AICA). 8](#_Toc112063825)

[(*S*)-*N*-(1-amino-3-methyl-1-oxobutan-2-yl)-1-(4-fluorobutyl)-1*H*-indole-3-carboxamide (25, AB-4F-BUTICA). 9](#_Toc112063826)

[(*S*)-*N*-(1-amino-3,3-dimethyl-1-oxobutan-2-yl)-1-(4-fluorobutyl)-1*H*-indole-3-carboxamide (26, ADB-4F-BUTICA). 9](#_Toc112063827)

[(*S*)-*N*-(1-amino-1-oxo-3-phenylpropan-2-yl)-1-(4-fluorobutyl)-1*H*-indole-3-carboxamide (27, APP-4F-BUTICA). 10](#_Toc112063828)

[(*S*)-*N*-(1-amino-3-methyl-1-oxobutan-2-yl)-1-(4-fluorobutyl)-1*H*-indazole-3-carboxamide (28, AB-4F-BUTINACA). 10](#_Toc112063829)

[(*S*)-*N*-(1-amino-3,3-dimethyl-1-oxobutan-2-yl)-1-(4-fluorobutyl)-1*H*-indazole-3-carboxamide (29, ADB-4F-BUTINACA). 11](#_Toc112063830)

[(*S*)-*N*-(1-amino-1-oxo-3-phenylpropan-2-yl)-1-(4-fluorobutyl)-1*H*-indazole-3-carboxamide (30, APP-4F-BUTINACA). 12](#_Toc112063831)

[(*S*)-*N*-(1-amino-3-methyl-1-oxobutan-2-yl)-1-(4-fluorobutyl)-1*H*-pyrrolo[2,3-*b*]pyridine-3-carboxamide (31, AB-4F-BUT7AICA). 12](#_Toc112063832)

[(*S*)-*N*-(1-amino-3,3-dimethyl-1-oxobutan-2-yl)-1-(4-fluorobutyl)-1*H*-pyrrolo[2,3-*b*]pyridine-3-carboxamide (32, ADB-4F-BUT7AICA). 13](#_Toc112063833)

[(*S*)-*N*-(1-amino-1-oxo-3-phenylpropan-2-yl)-1-(4-fluorobutyl)-1H-pyrrolo[2,3-*b*]pyridine-3-carboxamide (33, APP-4F-BUT7AICA). 13](#_Toc112063834)

[methyl (1-(4-fluorobutyl)-1*H*-indole-3-carbonyl)-*L*-valinate (34, MMB-4F-BUTICA). 14](#_Toc112063835)

[methyl (1-(4-fluorobutyl)-1*H*-indole-3-carbonyl)-*L*-phenylalaninate (35, MPP-4F-BUTICA). 14](#_Toc112063836)

[methyl (1-(4-fluorobutyl)-1*H*-indazole-3-carbonyl)-*L*-valinate (36, MMB-4F-BUTINACA). 15](#_Toc112063837)

[methyl (1-(4-fluorobutyl)-1*H*-indazole-3-carbonyl)-*L*-phenylalaninate (37, MPP-4F-BUTINACA). 16](#_Toc112063838)

[methyl (1-(4-fluorobutyl)-1*H*-pyrrolo[2,3-*b*]pyridine-3-carbonyl)-*L*-valinate (38, MMB-4F-BUT7AICA). 16](#_Toc112063839)

[methyl (*S*)-2-(1-(4-fluorobutyl)-1*H*-pyrrolo[2,3-*b*]pyridine-3-carboxamido)-3,3-dimethylbutanoate (39, MDMB-4F-BUT7AICA). 17](#_Toc112063840)

[methyl (1-(4-fluorobutyl)-1*H*-pyrrolo[2,3-*b*]pyridine-3-carbonyl)-*L*-phenylalaninate (40, MPP-4F-BUT7AICA). 17](#_Toc112063841)

[methyl 1-(4-fluorobutyl)-1*H*-pyrrolo[2,3-*b*]pyridine-3-carboxylate (49, 4F-BUT7AICA COOMe). 18](#_Toc112063842)

[1-(4-fluorobutyl)-1*H*-pyrrolo[2,3-*b*]pyridine-3-carboxylic acid (53, 4F-BUT7AICA COOH). 19](#_Toc112063843)

[**^1^H NMR, ^13^C NMR, LC-UV and UV spectra for newly described compounds. 20**](#_Toc112063844)

[**Figure S1.** ^1^H (400 MHz, DMSO-*d*_6_) and ^13^C (101 MHz, DMSO-*d*_6_) NMR spectra for (*S*)-*N*-(1-amino-3-methyl-1-oxobutan-2-yl)-1-(4-cyanobutyl)-1*H*-indole-3-carboxamide (AB-4CN-BUTICA, **5**). 20](#_Toc112063845)

[**Figure S2.** ^1^H (400 MHz, DMSO-*d*_6_) and ^13^C (101 MHz, DMSO-*d*_6_) NMR spectra for (*S*)-*N*-(1-amino-3,3-dimethyl-1-oxobutan-2-yl)-1-(4-cyanobutyl)-1*H*-indole-3-carboxamide (ADB-4CN-BUTICA, **9**). 21](#_Toc112063846)

[**Figure S3.** ^1^H (400 MHz, DMSO-*d*_6_) and ^13^C (101 MHz, DMSO-*d*_6_) NMR spectra for (*S*)-*N*-(1-amino-1-oxo-3-phenylpropan-2-yl)-1-(4-cyanobutyl)-1*H*-indole-3-carboxamide (APP-4CN-BUTICA, **10**). 22](#_Toc112063847)

[**Figure S4.** ^1^H (400 MHz, CD_3_OD) and ^13^C (101 MHz, CD_3_OD) NMR spectra for (*S*)-*N*-(1-amino-3-methyl-1-oxobutan-2-yl)-1-(4-cyanobutyl)-1*H*-indazole-3-carboxamide (AB-4CN-BUTINACA, **11**). 23](#_Toc112063848)

[**Figure S5.** ^1^H (400 MHz, CD_3_OD) and ^13^C (101 MHz, CD_3_OD) NMR spectra for (*S*)-*N*-(1-amino-3,3-dimethyl-1-oxobutan-2-yl)-1-(4-cyanobutyl)-1*H*-indazole-3-carboxamide (ADB-4CN-BUTINACA, **12**). 24](#_Toc112063849)

[**Figure S6.** ^1^H (400 MHz, CDCl_3_) and ^13^C (101 MHz, DMSO-*d*_6_) NMR spectra for (*S*)-*N*-(1-amino-1-oxo-3-phenylpropan-2-yl)-1-(4-cyanobutyl)-1*H*-indazole-3-carboxamide (APP-4CN-BUTINACA, **13**). 25](#_Toc112063850)

[**Figure S7.** ^1^H (400 MHz, DMSO-*d*_6_) and ^13^C (101 MHz, DMSO-*d*_6_) NMR spectra for (*S*)-*N*-(1-amino-3-methyl-1-oxobutan-2-yl)-1-(4-cyanobutyl)-1*H*-pyrrolo[2,3-*b*]pyridine-3-carboxamide (AB-4CN-BUT7AICA, **14**). 26](#_Toc112063851)

[**Figure S8.** ^1^H (400 MHz, DMSO-*d*_6_) and ^13^C (101 MHz, DMSO-*d*_6_) NMR spectra for (*S*)-*N*-(1-amino-3,3-dimethyl-1-oxobutan-2-yl)-1-(4-cyanobutyl)-1*H*-pyrrolo[2,3-*b*]pyridine-3-carboxamide (ADB-4CN-BUT7AICA, **15**). 27](#_Toc112063852)

[**Figure S9.** ^1^H (400 MHz, CD_3_OD) and ^13^C (101 MHz, DMSO-*d*_6_) NMR spectra for (*S*)-*N*-(1-amino-1-oxo-3-phenylpropan-2-yl)-1-(4-cyanobutyl)-1*H*-pyrrolo[2,3-*b*]pyridine-3-carboxamide (APP-4CN-BUT7AICA, **16**). 28](#_Toc112063853)

[**Figure S10.** ^1^H (400 MHz, CDCl_3_) and ^13^C (101 MHz, CD_3_OD) NMR spectra for methyl (1-(4-cyanobutyl)-1*H*-indole-3-carbonyl)-*L*-valinate (MMB-4CN-BUTICA, **17**). 29](#_Toc112063854)

[**Figure S11.** ^1^H (400 MHz, DMSO-*d*_6_) and ^13^C (101 MHz, CD_3_OD) NMR spectra for methyl (*S*)-2-(1-(4-cyanobutyl)-1*H*-indole-3-carboxamido)-3,3-dimethylbutanoate (MDMB-4CN-BUTICA, **18**). 30](#_Toc112063855)

[**Figure S12.** ^1^H (400 MHz, CDCl_3_) and ^13^C (101 MHz, DMSO-*d*_6_) NMR spectra for methyl (1-(4-cyanobutyl)-1*H*-indole-3-carbonyl)-*L*-phenylalaninate (MPP-4CN-BUTICA, **19**). 31](#_Toc112063856)

[**Figure S13.** ^1^H (400 MHz, DMSO-*d*_6_) and ^13^C (101 MHz, CD_3_OD) NMR spectra for methyl (1-(4-cyanobutyl)-1*H*-indazole-3-carbonyl)-*L*-valinate (MMB-4CN-BUTINACA, **6**). 32](#_Toc112063857)

[**Figure S14.** ^1^H (400 MHz, DMSO-*d*_6_) and ^13^C (101 MHz, DMSO-*d*_6_) NMR spectra for methyl (*S*)-2-(1-(4-cyanobutyl)-1*H*-indazole-3-carboxamido)-3,3-dimethylbutanoate (MDMB-4CN-BUTINACA, **20**). 33](#_Toc112063858)

[**Figure S15.** ^1^H (400 MHz, CDCl_3_) and ^13^C (101 MHz, CD_3_OD) NMR spectra for methyl (1-(4-cyanobutyl)-1*H*-indazole-3-carbonyl)-*L*-phenylalaninate (MPP-4CN-BUTINACA, **21**). 34](#_Toc112063859)

[**Figure S16.** ^1^H (400 MHz, CDCl_3_) and ^13^C (101 MHz, DMSO-*d*_6_) NMR spectra for methyl (1-(4-cyanobutyl)-1*H*-pyrrolo[2,3-*b*]pyridine-3-carbonyl)-*L*-valinate (MMB-4CN-BUT7AICA, **22**). 35](#_Toc112063860)

[**Figure S17.** ^1^H (400 MHz, CD_3_OD) and ^13^C (101 MHz, CD_3_OD) NMR spectra for methyl (*S*)-2-(1-(4-cyanobutyl)-1*H*-pyrrolo[2,3-*b*]pyridine-3-carboxamido)-3,3-dimethylbutanoate (MDMB-4CN-BUT7AICA, **23**). 36](#_Toc112063861)

[**Figure S18.** ^1^H (400 MHz, CDCl_3_) and ^13^C (101 MHz, CD_3_OD) NMR spectra for methyl (1-(4-cyanobutyl)-1*H*-pyrrolo[2,3-*b*]pyridine-3-carbonyl)-*L*-phenylalaninate (MPP-4CN-BUT7AICA, **24**). 37](#_Toc112063862)

[**Figure S19.** ^1^H (400 MHz, DMSO-*d*_6_) and ^13^C (101 MHz, DMSO-*d*_6_) NMR spectra for (*S*)-*N*-(1-amino-3-methyl-1-oxobutan-2-yl)-1-(4-fluorobutyl)-1*H*-indole-3-carboxamide (AB-4F-BUTICA, **25**). 38](#_Toc112063863)

[**Figure S20.** ^1^H (400 MHz, DMSO-*d*_6_) and ^13^C (101 MHz, CDCl_3_) NMR spectra for (*S*)-*N*-(1-amino-3,3-dimethyl-1-oxobutan-2-yl)-1-(4-fluorobutyl)-1*H*-indole-3-carboxamide (ADB-4F-BUTICA, **26**). 39](#_Toc112063864)

[**Figure S21.** ^1^H (400 MHz, CDCl_3_) and ^13^C (101 MHz, DMSO-*d*_6_) NMR spectra for (*S*)-*N*-(1-amino-1-oxo-3-phenylpropan-2-yl)-1-(4-fluorobutyl)-1*H*-indole-3-carboxamide (APP-4F-BUTICA, **27**). 40](#_Toc112063865)

[**Figure S22.** ^1^H (400 MHz, CD_3_OD) and ^13^C (101 MHz, CD_3_OD) NMR spectra for (*S*)-*N*-(1-amino-3-methyl-1-oxobutan-2-yl)-1-(4-fluorobutyl)-1*H*-indazole-3-carboxamide (AB-4F-BUTINACA, **28**). 41](#_Toc112063866)

[**Figure S23.** ^1^H (400 MHz, CD_3_OD) and ^13^C (101 MHz, CDCl_3_) NMR spectra for (*S*)-*N*-(1-amino-3,3-dimethyl-1-oxobutan-2-yl)-1-(4-fluorobutyl)-1*H*-indazole-3-carboxamide (ADB-4F-BUTINACA, **29**). 42](#_Toc112063867)

[**Figure S24.** ^1^H (400 MHz, DMSO-*d*_6_) and ^13^C (101 MHz, DMSO-*d*_6_) NMR spectra for (*S*)-*N*-(1-amino-1-oxo-3-phenylpropan-2-yl)-1-(4-fluorobutyl)-1*H*-indazole-3-carboxamide (APP-4F-BUTINACA, **30**). 43](#_Toc112063868)

[**Figure S25.** ^1^H (400 MHz, CD_3_OD) and ^13^C (101 MHz, CD_3_OD) NMR spectra for (*S*)-*N*-(1-amino-3-methyl-1-oxobutan-2-yl)-1-(4-fluorobutyl)-1*H*-pyrrolo[2,3-*b*]pyridine-3-carboxamide (AB-4F-BUT7AICA, **31**). 44](#_Toc112063869)

[**Figure S26.** ^1^H (400 MHz, CDCl_3_) and ^13^C (101 MHz, CD_3_OD) NMR spectra for (*S*)-*N*-(1-amino-3,3-dimethyl-1-oxobutan-2-yl)-1-(4-fluorobutyl)-1*H*-pyrrolo[2,3-*b*]pyridine-3-carboxamide (ADB-4F-BUT7AICA, **32**). 45](#_Toc112063870)

[**Figure S27.** ^1^H (400 MHz, DMSO-*d*_6_) and ^13^C (101 MHz, DMSO-*d*_6_) NMR spectra for (*S*)-*N*-(1-amino-1-oxo-3-phenylpropan-2-yl)-1-(4-fluorobutyl)-1*H*-pyrrolo[2,3-*b*]pyridine-3-carboxamide (APP-4F-BUT7AICA, **33**). 46](#_Toc112063871)

[**Figure S28.** ^1^H (400 MHz, CDCl_3_) and ^13^C (101 MHz, CD_3_OD) NMR spectra for methyl (1-(4-fluorobutyl)-1*H*-indole-3-carbonyl)-*L*-valinate (MMB-4F-BUTICA, **34**). 47](#_Toc112063872)

[**Figure S29.** ^1^H (400 MHz, DMSO-*d*_6_) and ^13^C (101 MHz, DMSO-*d*_6_) NMR spectra for methyl (*S*)-2-(1-(4-fluorobutyl)-1*H*-indole-3-carboxamido)-3,3-dimethylbutanoate (MDMB-4F-BUTICA, **7**). 48](#_Toc112063873)

[**Figure S30.** ^1^H (400 MHz, CDCl_3_) and ^13^C (101 MHz, CD_3_OD) NMR spectra for methyl (1-(4-fluorobutyl)-1*H*-indole-3-carbonyl)-*L*-phenylalaninate (MPP-4F-BUTICA, **35**). 49](#_Toc112063874)

[**Figure S31.** ^1^H (400 MHz, DMSO-*d*_6_) and ^13^C (101 MHz, CD_3_OD) NMR spectra for methyl (1-(4-fluorobutyl)-1*H*-indazole-3-carbonyl)-*L*-valinate (MMB-4F-BUTINACA, **36**). 50](#_Toc112063875)

[**Figure S32.** ^1^H (400 MHz, DMSO-*d*_6_) and ^13^C (101 MHz, DMSO-*d*_6_) NMR spectra for methyl (*S*)-2-(1-(4-fluorobutyl)-1*H*-indazole-3-carboxamido)-3,3-dimethylbutanoate (MDMB-4F-BUTINACA, **8**). 51](#_Toc112063876)

[**Figure S33.** ^1^H (400 MHz, CDCl_3_) and ^13^C (101 MHz, CD_3_OD) NMR spectra for methyl (1-(4-fluorobutyl)-1*H*-indazole-3-carbonyl)-*L*-phenylalaninate (MPP-4F-BUTINACA, **37**). 52](#_Toc112063877)

[**Figure S34.** ^1^H (400 MHz, DMSO-*d*_6_) and ^13^C (101 MHz, CD_3_OD) NMR spectra for methyl (1-(4-fluorobutyl)-1*H*-pyrrolo[2,3-*b*]pyridine-3-carbonyl)-*L*-valinate (MMB-4F-BUT7AICA¸**38**). 53](#_Toc112063878)

[**Figure S35.** ^1^H (400 MHz, DMSO-*d*_6_) and ^13^C (101 MHz, CD_3_OD) NMR spectra for methyl (*S*)-2-(1-(4-fluorobutyl)-1*H*-pyrrolo[2,3-*b*]pyridine-3-carboxamido)-3,3-dimethylbutanoate (MDMB-4F-BUT7AICA, **39**). 54](#_Toc112063879)

[**Figure S36.** ^1^H (400 MHz, CDCl_3_) and ^13^C (101 MHz, CD_3_OD) NMR spectra for methyl (1-(4-fluorobutyl)-1*H*-pyrrolo[2,3-*b*]pyridine-3-carbonyl)-*L*-phenylalaninate (MPP-4F-BUT7AICA, **40**). 55](#_Toc112063880)

[**Figure S37.** ^1^H (400 MHz, CDCl_3_) and ^13^C (101 MHz, CDCl_3_) NMR spectra for methyl 1-(4-fluorobutyl)-1*H*-pyrrolo[2,3-*b*]pyridine-3-carboxylate (4F-BUT7AICA COOMe, **49**). 56](#_Toc112063881)

[**Figure S38.** ^1^H (400 MHz, DMSO-*d*_6_) and ^13^C (75 MHz, DMSO-*d*_6_) NMR spectra for 1-(4-fluorobutyl)-1*H*-pyrrolo[2,3-*b*]pyridine-3-carboxylic acid (4F-BUT7AICA COOH, **53**). 57](#_Toc112063882)

[**Figure S39.** LC-UV chromatogram for (*S*)-*N*-(1-amino-3-methyl-1-oxobutan-2-yl)-1-(4-cyanobutyl)-1*H*-indole-3-carboxamide (AB-4CN-BUTICA, **5**). 58](#_Toc112063883)

[**Figure S40.** LC-UV chromatogram for (*S*)-*N*-(1-amino-3,3-dimethyl-1-oxobutan-2-yl)-1-(4-cyanobutyl)-1*H*-indole-3-carboxamide (ADB-4CN-BUTICA, **9**). 58](#_Toc112063884)

[**Figure S41.** LC-UV chromatogram for (*S*)-*N*-(1-amino-1-oxo-3-phenylpropan-2-yl)-1-(4-cyanobutyl)-1*H*-indole-3-carboxamide (APP-4CN-BUTICA, **10**). 59](#_Toc112063885)

[**Figure S42.** LC-UV chromatogram for (*S*)-*N*-(1-amino-3-methyl-1-oxobutan-2-yl)-1-(4-cyanobutyl)-1*H*-indazole-3-carboxamide (AB-4CN-BUTINACA, **11**). 59](#_Toc112063886)

[**Figure S43.** LC-UV chromatogram for (*S*)-*N*-(1-amino-3,3-dimethyl-1-oxobutan-2-yl)-1-(4-cyanobutyl)-1*H*-indazole-3-carboxamide (ADB-4CN-BUTINACA, **12**). 60](#_Toc112063887)

[**Figure S44.** LC-UV chromatogram for (*S*)-*N*-(1-amino-1-oxo-3-phenylpropan-2-yl)-1-(4-cyanobutyl)-1*H*-indazole-3-carboxamide (APP-4CN-BUTINACA, **13**). 60](#_Toc112063888)

[**Figure S45.** LC-UV chromatogram for (*S*)-*N*-(1-amino-3-methyl-1-oxobutan-2-yl)-1-(4-cyanobutyl)-1*H*-pyrrolo[2,3-*b*]pyridine-3-carboxamide (AB-4CN-BUT7AICA, **14**). 61](#_Toc112063889)

[**Figure S46.** LC-UV chromatogram for (*S*)-*N*-(1-amino-3,3-dimethyl-1-oxobutan-2-yl)-1-(4-cyanobutyl)-1*H*-pyrrolo[2,3-*b*]pyridine-3-carboxamide (ADB-4CN-BUT7AICA, **15**). 61](#_Toc112063890)

[**Figure S47.** LC-UV chromatogram for (*S*)-*N*-(1-amino-1-oxo-3-phenylpropan-2-yl)-1-(4-cyanobutyl)-1*H*-pyrrolo[2,3-*b*]pyridine-3-carboxamide (APP-4CN-BUT7AICA, **16**). 62](#_Toc112063891)

[**Figure S48.** LC-UV chromatogram for methyl (1-(4-cyanobutyl)-1*H*-indole-3-carbonyl)-*L*-valinate (MMB-4CN-BUTICA, **17**). 62](#_Toc112063892)

[**Figure S49.** LC-UV chromatogram for methyl (*S*)-2-(1-(4-cyanobutyl)-1*H*-indole-3-carboxamido)-3,3-dimethylbutanoate (MDMB-4CN-BUTICA, **18**). 63](#_Toc112063893)

[**Figure S50.** LC-UV chromatogram for methyl (1-(4-cyanobutyl)-1*H*-indole-3-carbonyl)-*L*-phenylalaninate (MPP-4CN-BUTICA, **19**). 63](#_Toc112063894)

[**Figure S51.** LC-UV chromatogram for methyl (1-(4-cyanobutyl)-1*H*-indazole-3-carbonyl)-*L*-valinate (MMB-4CN-BUTINACA, **6**). 64](#_Toc112063895)

[**Figure S52.** LC-UV chromatogram for methyl (*S*)-2-(1-(4-cyanobutyl)-1*H*-indazole-3-carboxamido)-3,3-dimethylbutanoate (MDMB-4CN-BUTINACA, **20**). 64](#_Toc112063896)

[**Figure S53.** LC-UV chromatogram for methyl (1-(4-cyanobutyl)-1*H*-indazole-3-carbonyl)-*L*-phenylalaninate (MPP-4CN-BUTINACA, **21**). 65](#_Toc112063897)

[**Figure S54.** LC-UV chromatogram for methyl (1-(4-cyanobutyl)-1*H*-pyrrolo[2,3-*b*]pyridine-3-carbonyl)-*L*-valinate (MMB-4CN-BUT7AICA, **22**). 65](#_Toc112063898)

[**Figure S55.** LC-UV chromatogram for methyl (*S*)-2-(1-(4-cyanobutyl)-1*H*-pyrrolo[2,3-*b*]pyridine-3-carboxamido)-3,3-dimethylbutanoate (MDMB-4CN-BUT7AICA, **23**). 66](#_Toc112063899)

[**Figure S56.** LC-UV chromatogram for methyl (1-(4-cyanobutyl)-1*H*-pyrrolo[2,3-*b*]pyridine-3-carbonyl)-*L*-phenylalaninate (MPP-4CN-BUT7AICA, **24**). 66](#_Toc112063900)

[**Figure S57.** LC-UV chromatogram for (*S*)-*N*-(1-amino-3-methyl-1-oxobutan-2-yl)-1-(4-fluorobutyl)-1*H*-indole-3-carboxamide (AB-4F-BUTICA, **25**). 67](#_Toc112063901)

[**Figure S58.** LC-UV chromatogram for (*S*)-*N*-(1-amino-3,3-dimethyl-1-oxobutan-2-yl)-1-(4-fluorobutyl)-1*H*-indole-3-carboxamide (ADB-4F-BUTICA, **26**). 67](#_Toc112063902)

[**Figure S59.** LC-UV chromatogram for (*S*)-*N*-(1-amino-1-oxo-3-phenylpropan-2-yl)-1-(4-fluorobutyl)-1*H*-indole-3-carboxamide (APP-4F-BUTICA, **27**). 68](#_Toc112063903)

[**Figure S60.** LC-UV chromatogram for (*S*)-*N*-(1-amino-3-methyl-1-oxobutan-2-yl)-1-(4-fluorobutyl)-1*H*-indazole-3-carboxamide (AB-4F-BUTINACA, **28**). 68](#_Toc112063904)

[**Figure S61.** LC-UV chromatogram for (*S*)-*N*-(1-amino-3,3-dimethyl-1-oxobutan-2-yl)-1-(4-fluorobutyl)-1*H*-indazole-3-carboxamide (ADB-4F-BUTINACA, **29**). 69](#_Toc112063905)

[**Figure S62.** LC-UV chromatogram for (*S*)-*N*-(1-amino-1-oxo-3-phenylpropan-2-yl)-1-(4-fluorobutyl)-1*H*-indazole-3-carboxamide (APP-4F-BUTINACA, **30**). 69](#_Toc112063906)

[**Figure S63.** LC-UV chromatogram for (*S*)-*N*-(1-amino-3-methyl-1-oxobutan-2-yl)-1-(4-fluorobutyl)-1*H*-pyrrolo[2,3-*b*]pyridine-3-carboxamide (AB-4F-BUT7AICA, **31**). 70](#_Toc112063907)

[**Figure S64.** LC-UV chromatogram for (*S*)-*N*-(1-amino-3,3-dimethyl-1-oxobutan-2-yl)-1-(4-fluorobutyl)-1*H*-pyrrolo[2,3-*b*]pyridine-3-carboxamide (ADB-4F-BUT7AICA, **32**). 70](#_Toc112063908)

[**Figure S65.** LC-UV chromatogram for (*S*)-*N*-(1-amino-1-oxo-3-phenylpropan-2-yl)-1-(4-fluorobutyl)-1*H*-pyrrolo[2,3-*b*]pyridine-3-carboxamide (APP-4F-BUT7AICA, **33**). 71](#_Toc112063909)

[**Figure S66.** LC-UV chromatogram for methyl (1-(4-fluorobutyl)-1*H*-indole-3-carbonyl)-*L*-valinate (MMB-4F-BUTICA, **34**). 71](#_Toc112063910)

[**Figure S67.** LC-UV chromatogram for methyl (*S*)-2-(1-(4-fluorobutyl)-1*H*-indole-3-carboxamido)-3,3-dimethylbutanoate (MDMB-4F-BUTICA, **7**). 72](#_Toc112063911)

[**Figure S68.** LC-UV chromatogram for methyl (1-(4-fluorobutyl)-1*H*-indole-3-carbonyl)-*L*-phenylalaninate (MPP-4F-BUTICA, **35**). 72](#_Toc112063912)

[**Figure S69.** LC-UV chromatogram for methyl (1-(4-fluorobutyl)-1*H*-indazole-3-carbonyl)-*L*-valinate (MMB-4F-BUTINACA, **36**). 73](#_Toc112063913)

[**Figure S70.** LC-UV chromatogram for methyl (*S*)-2-(1-(4-fluorobutyl)-1*H*-indazole-3-carboxamido)-3,3-dimethylbutanoate (MDMB-4F-BUTINACA, **8**). 73](#_Toc112063914)

[**Figure S71.** LC-UV chromatogram for methyl (1-(4-fluorobutyl)-1*H*-indazole-3-carbonyl)-*L*-phenylalaninate (MPP-4F-BUTINACA, **37**). 74](#_Toc112063915)

[**Figure S72.** LC-UV chromatogram for methyl (1-(4-fluorobutyl)-1*H*-pyrrolo[2,3-*b*]pyridine-3-carbonyl)-*L*-valinate (MMB-4F-BUT7AICA, **38**). 74](#_Toc112063916)

[**Figure S73.** LC-UV chromatogram for methyl (*S*)-2-(1-(4-fluorobutyl)-1*H*-pyrrolo[2,3-*b*]pyridine-3-carboxamido)-3,3-dimethylbutanoate (MDMB-4F-BUT7AICA, **39**). 75](#_Toc112063917)

[**Figure S74.** LC-UV chromatogram for methyl (1-(4-fluorobutyl)-1*H*-pyrrolo[2,3-*b*]pyridine-3-carbonyl)-*L*-phenylalaninate (MPP-4F-BUT7AICA, **40**). 75](#_Toc112063918)

[**Figure S75.** UV spectrum for (*S*)-*N*-(1-amino-3-methyl-1-oxobutan-2-yl)-1-(4-cyanobutyl)-1*H*-indole-3-carboxamide (AB-4CN-BUTICA, **5**). 76](#_Toc112063919)

[**Figure S76.** UV spectrum for (*S*)-*N*-(1-amino-3,3-dimethyl-1-oxobutan-2-yl)-1-(4-cyanobutyl)-1*H*-indole-3-carboxamide (ADB-4CN-BUTICA, **9**). 76](#_Toc112063920)

[**Figure S77.** UV spectrum for (*S*)-*N*-(1-amino-1-oxo-3-phenylpropan-2-yl)-1-(4-cyanobutyl)-1*H*-indole-3-carboxamide (APP-4CN-BUTICA, **10**). 76](#_Toc112063921)

[**Figure S78.** UV spectrum for (*S*)-*N*-(1-amino-3,3-dimethyl-1-oxobutan-2-yl)-1-(4-cyanobutyl)-1*H*-indazole-3-carboxamide (ADB-4CN-BUTINACA, **12**). 77](#_Toc112063922)

[**Figure S79.** UV spectrum for (*S*)-*N*-(1-amino-1-oxo-3-phenylpropan-2-yl)-1-(4-cyanobutyl)-1*H*-indazole-3-carboxamide (APP-4CN-BUTINACA, **13**). 77](#_Toc112063923)

[**Figure S80.** UV spectrum for (*S*)-*N*-(1-amino-3-methyl-1-oxobutan-2-yl)-1-(4-cyanobutyl)-1*H*-pyrrolo[2,3-*b*]pyridine-3-carboxamide (AB-4CN-BUT7AICA, **14**). 77](#_Toc112063924)

[**Figure S81.** UV spectrum for (*S*)-*N*-(1-amino-3,3-dimethyl-1-oxobutan-2-yl)-1-(4-cyanobutyl)-1*H*-pyrrolo[2,3-*b*]pyridine-3-carboxamide (ADB-4CN-BUT7AICA, **15**). 78](#_Toc112063925)

[**Figure S82.** UV spectrum for (*S*)-*N*-(1-amino-1-oxo-3-phenylpropan-2-yl)-1-(4-cyanobutyl)-1*H*-pyrrolo[2,3-*b*]pyridine-3-carboxamide (APP-4CN-BUT7AICA, **16**). 78](#_Toc112063926)

[**Figure S83.** UV spectrum for methyl (*S*)-2-(1-(4-cyanobutyl)-1*H*-indole-3-carboxamido)-3,3-dimethylbutanoate (MDMB-4CN-BUTICA, **18**). 78](#_Toc112063927)

[**Figure S84.** UV spectrum for methyl (1-(4-cyanobutyl)-1*H*-indole-3-carbonyl)-*L*-phenylalaninate (MPP-4CN-BUTICA, **19**). 79](#_Toc112063928)

[**Figure S85.** UV spectrum for methyl (*S*)-2-(1-(4-cyanobutyl)-1*H*-indazole-3-carboxamido)-3,3-dimethylbutanoate (MDMB-4CN-BUTINACA, **20**). 79](#_Toc112063929)

[**Figure S86.** UV spectrum for methyl (1-(4-cyanobutyl)-1*H*-indazole-3-carbonyl)-*L*-phenylalaninate (MPP-4CN-BUTINACA, **21**). 79](#_Toc112063930)

[**Figure S87.** UV spectrum for methyl (*S*)-2-(1-(4-cyanobutyl)-1*H*-pyrrolo[2,3-*b*]pyridine-3-carboxamido)-3,3-dimethylbutanoate (MDMB-4CN-BUT7AICA, **23**). 80](#_Toc112063931)

[**Figure S88.** UV spectrum for methyl (1-(4-cyanobutyl)-1*H*-pyrrolo[2,3-*b*]pyridine-3-carbonyl)-*L*-phenylalaninate (MPP-4CN-BUT7AICA, **24**). 80](#_Toc112063932)

[**Figure S89.** UV spectrum for (*S*)-*N*-(1-amino-3-methyl-1-oxobutan-2-yl)-1-(4-fluorobutyl)-1*H*-indole-3-carboxamide (AB-4F-BUTICA, **25**). 80](#_Toc112063933)

[**Figure S90.** UV spectrum for (*S*)-*N*-(1-amino-3,3-dimethyl-1-oxobutan-2-yl)-1-(4-fluorobutyl)-1*H*-indole-3-carboxamide (ADB-4F-BUTICA, **26**). 81](#_Toc112063934)

[**Figure S91.** UV spectrum for (*S*)-*N*-(1-amino-1-oxo-3-phenylpropan-2-yl)-1-(4-fluorobutyl)-1*H*-indole-3-carboxamide (APP-4F-BUTICA, **27**). 81](#_Toc112063935)

[**Figure S92.** UV spectrum for (*S*)-*N*-(1-amino-3-methyl-1-oxobutan-2-yl)-1-(4-fluorobutyl)-1*H*-indazole-3-carboxamide (AB-4F-BUTINACA, **28**). 81](#_Toc112063936)

[**Figure S93.** UV spectrum for (*S*)-*N*-(1-amino-3,3-dimethyl-1-oxobutan-2-yl)-1-(4-fluorobutyl)-1*H*-indazole-3-carboxamide (ADB-4F-BUTINACA, **29**). 82](#_Toc112063937)

[**Figure S94.** UV spectrum for (*S*)-*N*-(1-amino-1-oxo-3-phenylpropan-2-yl)-1-(4-fluorobutyl)-1*H*-indazole-3-carboxamide (APP-4F-BUTINACA, **30**). 82](#_Toc112063938)

[**Figure S95.** UV spectrum for (*S*)-*N*-(1-amino-3-methyl-1-oxobutan-2-yl)-1-(4-fluorobutyl)-1*H*-pyrrolo[2,3-*b*]pyridine-3-carboxamide (AB-4F-BUT7AICA, **31**). 82](#_Toc112063939)

[**Figure S96.** UV spectrum for (*S*)-*N*-(1-amino-3,3-dimethyl-1-oxobutan-2-yl)-1-(4-fluorobutyl)-1*H*-pyrrolo[2,3-*b*]pyridine-3-carboxamide (ADB-4F-BUT7AICA, **32**). 83](#_Toc112063940)

[**Figure S97.** UV spectrum for (*S*)-*N*-(1-amino-1-oxo-3-phenylpropan-2-yl)-1-(4-fluorobutyl)-1*H*-pyrrolo[2,3-*b*]pyridine-3-carboxamide (APP-4F-BUT7AICA, **33**). 83](#_Toc112063941)

[**Figure S98.** UV spectrum for methyl (1-(4-fluorobutyl)-1*H*-indole-3-carbonyl)-*L*-valinate (MMB-4F-BUTICA, **34**). 83](#_Toc112063942)

[**Figure S99.** UV spectrum for methyl (1-(4-fluorobutyl)-1*H*-indole-3-carbonyl)-*L*-phenylalaninate (MPP-4F-BUTICA, **35**). 84](#_Toc112063943)

[**Figure S100.** UV spectrum for methyl (1-(4-fluorobutyl)-1*H*-indazole-3-carbonyl)-*L*-valinate (MMB-4F-BUTINACA, **36**). 84](#_Toc112063944)

[**Figure S101.** UV spectrum for methyl (1-(4-fluorobutyl)-1*H*-indazole-3-carbonyl)-*L*-phenylalaninate (MPP-4F-BUTINACA, **37**). 84](#_Toc112063945)

[**Figure S102.** UV spectrum for methyl (1-(4-fluorobutyl)-1*H*-pyrrolo[2,3-*b*]pyridine-3-carbonyl)-*L*-valinate (MMB-4F-BUT7AICA, **38**). 85](#_Toc112063946)

[**Figure S103.** UV spectrum for methyl (*S*)-2-(1-(4-fluorobutyl)-1*H*-pyrrolo[2,3-*b*]pyridine-3-carboxamido)-3,3-dimethylbutanoate (MDMB-4F-BUT7AICA, **39**). 85](#_Toc112063947)

[**Figure S104.** UV spectrum for methyl (1-(4-fluorobutyl)-1*H*-pyrrolo[2,3-*b*]pyridine-3-carbonyl)-*L*-phenylalaninate (MPP-4F-BUT7AICA, **40**). 85](#_Toc112063948)

[Table T1. Strain energy calculations of compounds 5-40 87](#_Toc112063949)

[Calculated binding poses 89](#_Toc112063950)

[**Figure S105.** Computationally predicted binding modes of **11** (orange) and **28** (green) overlayed at the CB_1_ (PDB:6N4B) receptor. 89](#_Toc112063951)

[**Figure S106.** Computationally predicted binding modes of **13** (orange) and **30** (green) overlayed at the CB_1_ (PDB:6N4B) receptor. 90](#_Toc112063952)

[**Figure S107**. Computationally predicted binding modes of **9** (orange) and **26** (green) overlayed at the CB_1_ (PDB:6N4B) receptor. 91](#_Toc112063953)

[**Figure S108.** Computationally predicted binding modes of **15** (orange) and **32** (green) overlayed at the CB_1_ (PDB:6N4B) receptor. 92](#_Toc112063954)

[**Figure S109.** Computationally predicted binding modes of **23** (orange) and **39** (green) overlayed at the CB_1_ (PDB:6N4B) receptor. 93](#_Toc112063955)

[**Figure S110.** Computationally predicted binding modes of **18** (orange) and **7** (green) overlayed at the CB_1_ (PDB:6N4B) receptor. 94](#_Toc112063956)

[**Figure S111.** Computationally predicted binding modes of **11** (orange) and **28** (green) overlayed at the CB_2_ (PDB:6PT0) receptor. 95](#_Toc112063957)

[**Figure S112.** Computationally predicted binding modes of **13** (orange) and **30** (green) overlayed at the CB_2_ (PDB:6PT0) receptor. 96](#_Toc112063958)

[**Figure S113.** Computationally predicted binding modes of **9** (orange) and **26** (green) overlayed at the CB_2_ (PDB:6PT0) receptor. 97](#_Toc112063959)

[**Figure S114.** Computationally predicted binding modes of **15** (orange) and **32** (green) overlayed at the CB_2_ (PDB:6PT0) receptor. 98](#_Toc112063960)

[**Figure S115.** Computationally predicted binding modes of **23** (orange) and **39** (green) overlayed at the CB_2_ (PDB:6PT0) receptor. 99](#_Toc112063961)

[**Figure S116.** Computationally predicted binding modes of **18** (orange) and **7** (green) overlayed at the CB_2_ (PDB:6PT0) receptor. 100](#_Toc112063962)

# Synthesis and characterization for newly described compounds

## **General procedure A: amidation of 1-alkylindole-3-carboxyic acids, 1-alkyl-1*H*-indazole-3-carboxylic acids and 1-alkyl-1*H*-pyrrolo[2,3-*b*]pyridine-3-carboxylic acid.**

To a solution of the appropriate 1-alkylindole-3-carboxyic acid (**44** or **45**, 0.50 mmol), 1-alkyl-1*H*-indazole-3-carboxylic acid (**50** or **51**, 0.50 mmol) or 1-alkyl-1*H*-pyrrolo[2,3-*b*]pyridine-3-carboxylic acid (**52** or **53**, 0.50 mmol), the suitable amine reactant (0.55 mmol, 1.1 equiv.), HOBt·H_2_O (0.55 mmol, 1.1 equiv.) and EDC·HCl (0.75 mmol, 1.5 equiv.) in DMF (3 mL) was added. Et_3_N (1.65 mmol, 3.3 equiv.) was added dropwise and the mixture was stirred for 18 h. The mixture was poured onto H_2_O (80 mL), extracted with EtOAc (3 × 20 mL), and the combined organic layers were washed with H_2_O (2 × 40 mL), brine (40 mL), dried over Na_2_SO_4_, and the solvent evaporated under reduced pressure. The products were obtained following purification by flash chromatography.

## **(*S*)-*N*-(1-amino-3-methyl-1-oxobutan-2-yl)-1-(4-cyanobutyl)-1*H*-indole-3-carboxamide (5, AB-4CN-BUTICA).**

Subjecting 1-(4-cyanobutyl)-1*H*-indole-3-carboxylic acid (**48**, 97 mg, 0.40 mmol) and (*S*)-2-amino-3-methylbutanamide hydrochloride (66 mg, 0.43 mmol) to general procedure A furnished, following purification by flash chromatography (CH_2_Cl_2_:MeOH 100:0 to 80:20), **9** as a white powder (72 mg, 53%). R*_f_* 0.03 (hexane:EtOAc 1:1); m.p. 177–180 °C; ^1^H NMR (400 MHz, DMSO-*d*_6_) *δ* 8.25 (s, 1H), 8.13 – 8.08 (m, 1H), 7.57 (d, *J* = 8.2 Hz, 1H), 7.49 (d, *J* = 9.1 Hz, 1H), 7.47 (brs, 1H), 7.25 – 7.12 (m, 2H), 7.06 (brs, 1H), 4.35 (dd, *J* = 8.9, 7.0 Hz, 1H), 4.26 (td, *J* = 6.9, 2.0 Hz, 2H), 2.55 (t, *J* = 7.1 Hz, 2H), 2.08 (sext., *J* = 6.8 Hz, 1H), 1.95 – 1.83 (m, 2H), 1.63 – 1.51 (m, 2H), 0.93 (dd, *J* = 6.8, 3.0 Hz, 6H); ^13^C NMR (101 MHz, DMSO-*d*_6_) *δ* 174.0, 164.5, 136.5, 131.7, 126.9, 122.4, 121.5, 121.1, 120.9, 110.8, 110.0, 57.8, 45.5, 30.9, 29.2, 22.7, 20.0, 18.9, 16.3; *ѵ*_max_ (cm^-1^): 3293, 1620, 1514, 1466, 1394, 1269, 1231, 1163, 802, 741, 663, 617, 562; HRMS (ESI, +ive) C_19_H_24_N_4_O_2_ theoretical mass 340.1899, experimental exact mass 340.1906 (mass error 2.03 ppm).

## **(*S*)-*N*-(1-amino-3,3-dimethyl-1-oxobutan-2-yl)-1-(4-cyanobutyl)-1*H*-indole-3-carboxamide (9, ADB-4CN-BUTICA).**

Subjecting 1-(4-cyanobutyl)-1*H*-indole-3-carboxylic acid (**44**, 97 mg, 0.40 mmol) and (*S*)-2-amino-3,3-dimethylbutanamide hydrochloride (72 mg, 0.45 mmol) to general procedure A furnished, following purification by flash chromatography (CH_2_Cl_2_:MeOH 100:0 to 90:10), **9** as a white solid (97 mg, 69%). R*_f_* 0.54 (hexane:EtOAc 1:4); m.p. 101–103 °C; ^1^H NMR (400 MHz, DMSO-*d*_6_) *δ* 8.30 (s, 1H), 8.07 (d, *J* = 7.8 Hz, 1H), 7.58 (d, *J* = 8.1 Hz, 1H), 7.56 (s, 1H), 7.26 – 7.13 (m, 3H), 7.11 (s, 1H), 4.47 (d, *J* = 9.5 Hz, 1H), 4.29 – 4.23 (m, 2H), 2.55 (t, *J* = 7.1 Hz, 2H), 1.95 – 1.84 (m, 2H), 1.56 (m, 2H), 1.01 (s, 9H); ^13^C NMR (101 MHz, DMSO-*d*_6_) *δ* 172.5, 163.7, 136.1, 131.5, 126.3, 122.0, 120.8, 120.8, 120.5, 110.5, 109.5, 59.1, 45.1, 34.1, 28.8, 26.9, 22.3, 15.8; *ѵ*_max_ (cm^-1^): 3192, 2955, 1613, 1535, 1464, 1394, 1227, 1166, 746; HRMS (ESI, +ive) C_20_H_26_N_4_O_2_ theoretical mass 354.2056, experimental exact mass 354.2049 (mass error 1.7 ppm).

## **(*S*)-*N*-(1-amino-1-oxo-3-phenylpropan-2-yl)-1-(4-cyanobutyl)-1*H*-indole-3-carboxamide (10, APP-4CN-BUTICA).**

Subjecting 1-(4-cyanobutyl)-1*H*-indole-3-carboxylic acid (**44**, 97 mg, 0.40 mmol) and (*S*)-2-amino-3-phenylpropanamide hydrochloride (69 mg, 0.34 mmol) to general procedure A furnished, following purification by flash chromatography (CH_2_Cl_2_:MeOH 100:0 to 85:15), **10** as a white solid (91 mg, 59%). R*_f_* 0.03 (hexane:EtOAc 1:1); m.p. 84–85 °C; ^1^H NMR (400 MHz, DMSO-*d*_6_) *δ* 8.12 (s, 1H), 8.02 (d, *J* = 8.1 Hz, 1H), 7.87 (d, *J* = 8.5 Hz, 1H), 7.54 (d, *J* = 8.2 Hz, 1H), 7.51 (brs, 1H), 7.33 (dd, *J* = 8.1, 1.4 Hz, 2H), 7.24 (t, *J* = 7.6 Hz, 2H), 7.21 – 7.10 (m, 3H), 7.08 (brs, 1H), 4.71 – 4.62 (m, 1H), 4.25 (t, *J* = 6.9 Hz, 2H), 3.11 (dd, *J* = 13.7, 4.6 Hz, 1H), 2.95 (dd, *J* = 13.7, 9.9 Hz, 1H), 2.55 (t, *J* = 7.1 Hz, 2H), 1.92 – 1.82 (m, 2H), 1.61 – 1.51 (m, 2H); ^13^C NMR (101 MHz, DMSO-*d*_6_) *δ* 173.8, 163.9, 138.6, 136.0, 131.1, 129.2, 128.0, 126.4, 126.1, 122.0, 121.1, 120.6, 120.5, 110.3, 109.5, 53.8, 45.1, 37.6, 28.8, 22.3, 15.8; *ѵ*_max_ (cm^-1^): 3311, 1671, 1614, 1536, 1463, 1394, 1272, 1228, 1166, 746, 699, 509; HRMS (ESI, +ive) C_23_H_24_N_4_O_2_ theoretical mass 388.1899, experimental exact mass 388.1906 (mass error 1.79 ppm).

## **(*S*)-*N*-(1-amino-3,3-dimethyl-1-oxobutan-2-yl)-1-(4-cyanobutyl)-1*H*-indazole-3-carboxamide (12, ADB-4CN-BUTINACA).**

Subjecting 1-(4-cyanobutyl)-1*H*-indazole-3-carboxylic acid (**50**, 297 mg, 1.22 mmol) and (*S*)-2-amino-3,3-dimethylbutanamide hydrochloride (223 mg, 1.34 mmol) to general procedure A furnished, following purification by flash chromatography (CH_2_Cl_2_:MeOH 100:0 to 93:7), **12** as a white solid (383 mg, 89%). R*_f_* 0.43 (hexane:EtOAc 2:8); m.p. 82–86 °C; ^1^H NMR (400 MHz, CD_3_OD) *δ* 8.22 (dd, *J* = 8.3, 1.4 Hz, 1H), 7.68 (d, *J* = 8.5 Hz, 1H), 7.48 (dd, *J* = 8.4, 6.9 Hz, 1H), 7.30 (dd, *J* = 8.2, 6.8 Hz, 1H), 4.57 (t, *J* = 6.9 Hz, 2H), 4.54 (s, 1H), 2.53 – 2.46 (m, 2H), 2.11 (quin, *J* = 7.1 Hz, 2H), 1.67 (quin, *J* = 7.3 Hz, 2H), 1.10 (s, 9H); ^13^C NMR (101 MHz, CD_3_OD) *δ* 175.1, 164.0, 142.5, 137.8, 128.2, 124.0, 123.9, 123.0, 120.8, 111.0, 61.0, 35.6, 29.7, 27.2, 23.9, 16.9; *ѵ*_max_ (cm^-1^): 3327, 3199, 2956, 2248, 1650, 1525, 1491, 1397, 1367, 1314, 1208, 1169, 1133, 1005, 931, 826, 776, 750, 664, 561, 548, 527, 510; HRMS (ESI, +ive) C_19_H_25_N_5_O_2_ theoretical mass 355.2008, experimental exact mass 355.2015 (mass error 2.02 ppm).

## **(*S*)-*N*-(1-amino-1-oxo-3-phenylpropan-2-yl)-1-(4-cyanobutyl)-1*H*-indazole-3-carboxamide (13, APP-4CN-BUTINACA).**

Subjecting 1-(4-cyanobutyl)-1*H*-indazole-3-carboxylic acid (**50**, 80 mg, 0.33 mmol) and (*S*)-2-amino-3-phenylpropanamide hydrochloride (70 mg, 0.35 mmol) to general procedure A furnished, following purification by flash chromatography (CH_2_Cl_2_:MeOH 100:0 to 90:10), **13** as a colorless oil (117 mg, 91%). R*_f_* 0.10 (hexane:EtOAc 1:1); ^1^H NMR (400 MHz, CDCl_3_) *δ* 8.30 (dt, *J* = 8.2, 1.0 Hz, 1H), 7.49 (d, *J* = 8.0 Hz, 1H), 7.45 – 7.37 (m, 2H), 7.34 – 7.23 (m, 6H), 5.97 (brs, 1H), 5.37 (brs, 1H), 4.96 – 4.87 (m, 1H), 4.42 (t, *J* = 6.7 Hz, 2H), 3.30 (dd, *J* = 13.9, 6.5 Hz, 1H), 3.30 (dd, *J* = 13.9, 6.5 Hz, 1H), 2.35 (t, *J* = 7.0 Hz, 2H), 2.17 – 2.07 (m, 2H), 1.68 – 1.58 (m, 2H); ^13^C NMR (101 MHz, DMSO-*d*_6_) *δ* 172.6, 161.2, 140.5, 137.6, 136.6, 129.3, 128.0, 126.7, 126.3, 122.5, 122.0, 121.7, 120.5, 110.3, 53.2, 47.8, 37.7, 28.5, 22.2, 15.8; *ѵ*_max_ (cm^-1^): 3307, 3195, 2928, 2247, 1645, 1574, 1525, 1490, 1454, 1402, 1307, 1214, 1168, 1133, 1030, 1005, 912, 841, 775, 749, 700, 564, 519; HRMS (ESI, +ive) C_22_H_23_N_5_O_2_ theoretical mass 389.1852, experimental exact mass 389.1846 (mass error 1.5 ppm).

## **(*S*)-*N*-(1-amino-3-methyl-1-oxobutan-2-yl)-1-(4-cyanobutyl)-1*H*-pyrrolo[2,3-*b*]pyridine-3-carboxamide (14, AB-4CN-BUT7AICA).**

Subjecting 1-(4-cyanobutyl)-1*H*-pyrrolo[2,3-*b*]pyridine-3-carboxylic acid (**52**, 98 mg, 0.40 mmol) and (*S*)-2-amino-3-methylbutanamide hydrochloride (65 mg, 0.43 mmol) to general procedure A furnished, following purification by flash chromatography (CH_2_Cl_2_:MeOH 100:0 to 90:10), **14** as a white solid (68 mg, 50%). R *_f_* 0.27 (hexane:EtOAc 1:4); m.p. 181–182 °C; ^1^H NMR (400 MHz, DMSO-*d*_6_) *δ* 8.47 (s, 1H), 8.42 (dd, *J* = 7.9, 1.6 Hz, 1H), 8.32 (dd, *J* = 4.7, 1.6 Hz, 1H), 7.70 (d, *J* = 8.9 Hz, 1H), 7.48 (brs, 1H), 7.22 (dd, *J* = 7.9, 4.6 Hz, 1H), 7.07 (brs, 1H), 4.42 – 4.25 (m, 3H), 2.56 (t, *J* = 7.1 Hz, 2H), 2.09 (h, *J* = 6.8 Hz, 1H), 1.99 – 1.88 (m, 2H), 1.61 – 1.51 (m, 2H), 0.94 (dd, *J* = 6.8, 1.8 Hz, 6H); ^13^C NMR (101 MHz, DMSO-*d*_6_) *δ* 173.4, 163.5, 147.1, 143.3, 131.2, 129.5, 120.5, 118.9, 117.2, 108.1, 57.5, 43.3, 30.4, 28.8, 22.3, 19.5, 18.5, 15.8; *ѵ*_max_ (cm^-1^): 3363, 3196, 2955, 1634, 1571, 1534, 1513, 1452, 1426, 1352, 1293, 1265, 1212, 1166, 1136, 871, 799, 773, 747, 655, 574; HRMS (ESI, +ive) C_18_H_23_N_5_O_2_ theoretical mass 341.1852, experimental exact mass 341.1857 (mass error 1.51 ppm).

## **(*S*)-*N*-(1-amino-3,3-dimethyl-1-oxobutan-2-yl)-1-(4-cyanobutyl)-1*H*-pyrrolo[2,3-*b*]pyridine-3-carboxamide (15, ADB-4CN-BUT7AICA).**

Subjecting 1-(4-cyanobutyl)-1*H*-pyrrolo[2,3-*b*]pyridine-3-carboxylic acid (**52**, 99 mg, 0.41 mmol) and (*S*)-2-amino-3,3-dimethylbutanamide hydrochloride (71 mg, 0.45 mmol) to general procedure A furnished, following purification by flash chromatography (CH_2_Cl_2_:MeOH 100:0 to 90:10), **15** as a white solid (95 mg, 65%). R *_f_* 0.42 (hexane:EtOAc 1:4); m.p. 93–94 °C; ^1^H NMR (400 MHz, DMSO-*d*_6_) *δ* 8.54 (s, 1H), 8.40 (dd, *J* = 7.9, 1.6 Hz, 1H), 8.32 (dd, *J* = 4.7, 1.6 Hz, 1H), 7.55 (s, 1H), 7.40 (d, *J* = 9.5 Hz, 1H), 7.23 (dd, *J* = 7.9, 4.7 Hz, 1H), 7.10 (s, 1H), 4.47 (d, *J* = 9.5 Hz, 1H), 4.41 – 4.25 (m, 2H), 2.56 (t, *J* = 7.1 Hz, 2H), 2.00 – 1.88 (m, 2H), 1.60 – 1.49 (m, 2H), 1.01 (s, 9H); ^13^C NMR (101 MHz, DMSO-*d*_6_) *δ* 172.3, 163.3, 147.2, 143.3, 131.5, 129.3, 120.5, 118.8, 117.2, 108.0, 59.3, 43.4, 34.1, 28.7, 26.9, 22.3, 15.8; *ѵ*_max_ (cm^-1^): 3324, 2956, 1673, 1616, 1535, 1426, 1398, 1259, 1164, 1131, 800, 775, 749, 543, 507; HRMS (ESI, +ive) C_19_H_25_N_5_O_2_ theoretical mass 355.2008, experimental exact mass 355.2012 (mass error 1.02 ppm).

## **(*S*)-*N*-(1-amino-1-oxo-3-phenylpropan-2-yl)-1-(4-cyanobutyl)-1*H*-pyrrolo[2,3-*b*]pyridine-3-carboxamide (16, APP-4CN-BUT7AICA).**

Subjecting 1-(4-cyanobutyl)-1*H*-pyrrolo[2,3-*b*]pyridine-3-carboxylic acid (**52**, 100 mg, 0.41 mmol) and (*S*)-2-amino-3-phenylpropanamide hydrochloride (69 mg, 0.35 mmol) to general procedure A furnished, following purification by flash chromatography (CH_2_Cl_2_:MeOH 100:0 to 90:10), **16** as a white solid (67 mg, 42%). R*_f_* 0.31 (hexane:EtOAc 1:4); m.p. 182–184 °C; ^1^H NMR (400 MHz, CD_3_OD) *δ* 8.33 – 8.28 (m, 2H), 8.06 (s, 1H), 7.35 – 7.16 (m, 6H), 4.90 – 4.86 (m, 1H), 4.38 (t, *J* = 7.0 Hz, 2H), 3.29 – 3.26 (m, 1H), 3.07 (dd, *J* = 13.9, 8.9 Hz, 2H), 2.50 (t, *J* = 7.1 Hz, 2H), 2.07 – 1.98 (m, 2H), 1.68 – 1.59 (m, 2H); ^13^C NMR (101 MHz, DMSO-*d*_6_) *δ* 173.6, 163.4, 147.0, 143.3, 138.5, 131.1, 129.5, 129.2, 128.0, 126.2, 120.5, 118.7, 117.2, 108.2, 53.8, 43.4, 37.6, 28.8, 22.3, 15.8; *ѵ*_max_ (cm^-1^): 3439, 3311, 3200, 2928, 2236, 1672, 1621, 1608, 1571, 1528, 1507, 1450, 1425, 1398, 1357, 1336, 1309, 1282, 1247, 1208, 1166, 1127, 1043, 1029, 863, 799, 774, 742, 724, 704, 633, 614, 565, 525; HRMS (ESI, +ive) C_22_H_23_N_5_O_2_ theoretical mass 389.1852, experimental exact mass 389.1846 (mass error 1.5 ppm).

## **methyl (*S*)-2-(1-(4-cyanobutyl)-1*H*-indole-3-carboxamido)-3,3-dimethylbutanoate (18, MDMB-4CN-BUTICA).**

Subjecting 1-(4-cyanobutyl)-1*H*-indole-3-carboxylic acid (**44**, 242 mg, 1.00 mmol) and methyl (*S*)-2-amino-3,3-dimethylbutanoate hydrochloride (200 mg, 1.10 mmol) to general procedure A furnished, following purification by flash chromatography (hexane:EtOAc 90:10 to 60:40), **18** as a white solid (154 mg, 42%). R*_f_* 0.30 (hexane:EtOAc 1:1); m.p. 124–127 °C; ^1^H NMR (400 MHz, DMSO-*d*_6_) *δ* 8.39 (s, 1H), 8.12 – 8.06 (m, 1H), 7.63 (d, *J* = 8.8 Hz, 1H), 7.57 (d, *J* = 8.2 Hz, 1H), 7.25 – 7.17 (m, 1H), 7.16 – 7.11 (m, 1H), 4.49 (d, *J* = 8.7 Hz, 1H), 4.26 (t, *J* = 7.1 Hz, 2H), 3.66 (s, 3H), 2.56 (t, *J* = 7.1 Hz, 2H), 1.90 (quin., *J* = 7.1 Hz, 2H), 1.58 (quin., *J* = 7.2 Hz, 2H), 1.03 (s, 9H); ^13^C NMR (101 MHz, CD_3_OD) *δ* 174.3, 167.0, 148.7, 144.7, 132.5, 131.4, 120.9, 120.9, 118.7, 109.6, 59.5, 52.5, 45.1, 31.9, 30.3, 23.8, 19.6, 19.1, 16.9; *ѵ*_max_ (cm^-1^): 3439, 2957, 2245, 1964, 1729, 1638, 1529, 1501, 1467, 1428, 1395, 1378, 1341, 1312, 1255, 1239, 1217, 1191, 1164, 1129, 1116, 1012, 997, 942, 920, 862, 829, 772, 752, 690, 626, 607, 565, 533, 520, 511; HRMS (ESI, +ive) C_21_H_27_N_3_O_3_ theoretical mass 369.2052, experimental exact mass 369.2060 (mass error 1.95 ppm).

## **methyl (1-(4-cyanobutyl)-1*H*-indole-3-carbonyl)-*L*-phenylalaninate (19, MPP-4CN-BUTICA).**

Subjecting 1-(4-cyanobutyl)-1*H*-indole-3-carboxylic acid (**44**, 242 mg, 1.00 mmol) and methyl (*S*)-2-amino-3-phenylpropanoate hydrochloride (237 mg, 1.10 mmol) to general procedure A furnished, following purification by flash chromatography (hexane:EtOAc 80:20 to 40:60), **19** as a white solid (337 mg, 84%). R*_f_* 0.19 (hexane:EtOAc 1:1); m.p. 144–146 °C; ^1^H NMR (400 MHz, CDCl_3_) *δ* 7.79 – 7.74 (m, 1H), 7.67 (s, 1H), 7.36 (m, 1H), 7.32 – 7.25 (m, 4H), 7.24 – 7.21 (m, 1H), 7.18 (m, 2H), 6.42 (d, *J* = 7.5 Hz, 1H), 5.17 (dt, *J* = 7.5, 5.6 Hz, 1H), 4.20 (t, *J* = 6.8 Hz, 2H), 3.78 (s, 3H), 3.34 (dd, *J* = 13.8, 5.8 Hz, 1H), 3.26 (dd, *J* = 13.8, 5.4 Hz, 1H), 2.33 (t, *J* = 7.0 Hz, 2H), 2.08 – 1.99 (m, 2H), 1.69 – 1.61 (m, 2H); ^13^C NMR (75 MHz, DMSO-*d*_6_) *δ* 172.7, 164.2, 137.8, 136.0, 131.1, 129.1, 128.2, 126.5, 122.1, 121.2, 120.7, 120.5, 110.3, 109.1, 53.6, 51.8, 45.1, 36.6, 28.8, 22.3, 15.8; *ѵ*_max_: (cm^-1^): 3322, 2950, 2245, 1734, 1620, 1546, 1524, 1466, 1388, 1340, 1283, 1223, 1168, 1132, 1118, 1083, 1034, 1013, 988, 920, 872, 830, 779, 751, 703, 668, 606, 577, 534, 516; HRMS (ESI, +ive) C_24_H_25_N_3_O_3_ theoretical mass 403.1896, experimental exact mass 403.1890 (mass error 1.5 ppm).

## **methyl (*S*)-2-(1-(4-cyanobutyl)-1*H*-indazole-3-carboxamido)-3,3-dimethylbutanoate (20, MDMB-4CN-BUTINACA).**

Subjecting 1-(4-cyanobutyl)-1*H*-indazole-3-carboxylic acid (**50**, 124 mg, 0.50 mmol) and methyl (*S*)-2-amino-3,3-dimethylbutanoate hydrochloride (96 mg, 0.52 mmol) to general procedure A furnished, following purification by flash chromatography (hexane:EtOAc 88:12 to 75:25), **20** as a colorless oil (118 mg, 64%). R*_f_* 0.56 (hexane:EtOAc 1:1); ^1^H NMR (400 MHz, DMSO-*d*_6_) *δ* 8.13 (d, *J* = 8.2 Hz, 1H), 7.86 – 7.80 (m, 1H), 7.64 (d, *J* = 9.2 Hz, 1H), 7.49 (ddd, *J* = 8.4, 6.9, 1.1 Hz, 1H), 7.30 (ddd, *J* = 7.8, 6.9, 0.8 Hz, 1H), 4.57 (t, *J* = 7.0 Hz, 2H), 4.49 (d, *J* = 9.3 Hz, 1H), 3.70 (s, 3H), 2.56 (t, *J* = 7.2 Hz, 2H), 2.02 – 1.93 (m, 2H), 1.65 – 1.50 (m, 2H), 1.02 (s, 9H).^13^C NMR (101 MHz, DMSO-*d*_6_) *δ* 171.3, 161.5, 140.7, 136.1, 126.9, 122.8, 121.9, 121.5, 120.5, 110.5, 59.4, 51.8, 47.9, 34.2, 28.4, 26.4, 22.2, 15.7; *ѵ*_max_ (cm^-1^): 2955, 1734, 1666, 1522, 1490, 1331, 1213, 1164, 1134, 1038, 1004, 925, 835, 775, 750, 563; HRMS (ESI, +ive) C_20_H_26_N_4_O_3_ theoretical mass 370.2005, experimental exact mass 370.2012 (mass error 1.84 ppm).

## **methyl (1-(4-cyanobutyl)-1*H*-indazole-3-carbonyl)-*L*-phenylalaninate (21, MPP-4CN-BUTINACA).**

Subjecting 1-(4-cyanobutyl)-1*H*-indazole-3-carboxylic acid (**50**, 122 mg, 0.50 mmol) and methyl (*S*)-2-amino-3-phenylpropanoate hydrochloride (129 mg, 0.60 mmol) to general procedure A furnished, following purification by flash chromatography (hexane:EtOAc 90:10 to 40:60), **21** as a white solid (173 mg, 86%). R*_f_* 0.39 (hexane:EtOAc 1:1); m.p. 82.5–84.5 °C; ^1^H NMR (400 MHz, CDCl_3_) *δ* 8.44 – 8.24 (m, 1H), 7.49 – 7.37 (m, 3H), 7.32 – 7.18 (m, 6H), 5.13 (dt, *J* = 8.1, 6.0 Hz, 1H), 4.44 (t, *J* = 6.7 Hz, 2H), 3.75 (s, 3H), 3.33 – 3.22 (m, 2H), 2.37 (t, *J* = 7.0 Hz, 2H), 2.18 – 2.07 (m, 2H), 1.73 – 1.59 (m, 2H); ^13^C NMR (101 MHz, CD_3_OD) *δ* 173.7, 167.6, 138.0, 132.7, 127.8, 123.8, 122.5, 121.9, 120.9, 111.4, 110.6, 61.8, 52.3, 46.8, 35.5, 30.2, 27.2, 23.9, 17.0; *ѵ*_max_: (cm^-1^): 3362, 2950, 2243, 1750, 1737, 1656, 1642, 1524, 1492, 1456, 1438, 1411, 1353, 1318, 1282, 1234, 1206, 1171, 1134, 1066, 1039, 1000, 983, 913, 861, 843, 780, 757, 739, 699, 653, 620, 570, 523; HRMS (ESI, +ive) C_23_H_24_N_4_O_3_ theoretical mass 404.1848, experimental exact mass 404.1856 (mass error 1.82 ppm).

## **methyl (*S*)-2-(1-(4-cyanobutyl)-1*H*-pyrrolo[2,3-*b*]pyridine-3-carboxamido)-3,3-dimethylbutanoate (23, MDMB-4CN-BUT7AICA).**

Subjecting 1-(4-cyanobutyl)-1*H*-pyrrolo[2,3-*b*]pyridine-3-carboxylic acid (**52**, 243 mg, 1.00 mmol) and methyl (*S*)-2-amino-3,3-dimethylbutanoate hydrochloride (200 mg, 1.10 mmol) to general procedure A furnished, following purification by flash chromatography (hexane:EtOAc 60:40 to 50:50), **23** as a colorless oil (106 mg, 29%). R*_f_* 0.50 (hexane:EtOAc 1:4); ^1^H NMR (300 MHz, CD_3_OD) δ 8.45 (dd, *J* = 8.0, 1.6 Hz, 1H), 8.33 (dd, *J* = 4.8, 1.6 Hz, 1H), 8.29 (s, 1H), 7.25 (dd, *J* = 8.0, 4.8 Hz, 1H), 4.63 (s, 1H), 4.49 – 4.35 (m, 2H), 3.76 (s, 3H), 2.51 (t, *J* = 7.1 Hz, 2H), 2.12 – 1.99 (m, 2H), 1.73 – 1.58 (m, 2H), 1.10 (s, 9H); ^13^C NMR (75 MHz, CD_3_OD) *δ* 173.5, 166.8, 148.7, 144.7, 132.6, 131.3, 120.8, 118.7, 109.5, 61.9, 52.2, 45.1, 35.5, 30.3, 27.3, 23.8, 16.9; *ѵ*_max_ (cm^-1^): 3331, 2954, 2248, 1738, 1615, 1568, 1536, 1480, 1426, 1400, 1371, 1329, 1266, 1214, 1163, 1132, 1038, 1013, 988, 920, 869, 834, 804, 783, 755, 631, 578; HRMS (ESI, +ive) C_20_H_26_N_4_O_3_ theoretical mass 370.2005, experimental exact mass 370.2000 (mass error 1.2 ppm).

## **methyl (1-(4-cyanobutyl)-1*H*-pyrrolo[2,3-*b*]pyridine-3-carbonyl)-*L*-phenylalaninate (24, MPP-4CN-BUT7AICA).**

Subjecting 1-(4-cyanobutyl)-1*H*-pyrrolo[2,3-*b*]pyridine-3-carboxylic acid (**52**, 243 mg, 1.00 mmol) and methyl (*S*)-2-amino-3-phenylpropanoate hydrochloride (237 mg, 1.10 mmol) to general procedure A furnished, following purification by flash chromatography (hexane:EtOAc 80:20 to 50:50), **24** as a white solid (248 mg, 61%). R*_f_* 0.56 (hexane:EtOAc 1:4); m.p. 102–105 °C; ^1^H NMR (400 MHz, CDCl_3_) *δ* 8.35 (dd, *J* = 4.7, 1.5 Hz, 1H), 8.15 (d, *J* = 8.0 Hz, 1H), 7.75 (s, 1H), 7.34 – 7.27 (m, 3H), 7.21 – 7.13 (m, 3H), 6.34 (d, *J* = 7.5 Hz, 1H), 5.14 (dt, *J* = 7.5, 5.5 Hz, 1H), 4.39 (t, *J* = 6.9 Hz, 2H), 3.79 (s, 3H), 3.33 (dd, *J* = 13.9, 5.8 Hz, 1H), 3.26 (dd, *J* = 13.9, 5.3 Hz, 1H), 2.41 (t, *J* = 7.1 Hz, 2H), 2.15 – 1.99 (m, 2H), 1.75 – 1.58 (m, 2H); ^13^C NMR (101 MHz, CD_3_OD) *δ* 174.1, 166.7, 148.6, 144.7, 138.5, 132.4, 131.3, 130.2, 129.6, 127.9, 120.8, 120.6, 118.6, 109.6, 55.3, 52.8, 45.1, 38.4, 30.3, 23.8, 16.9; *ѵ*_max_ (cm^-1^): 3365, 2949, 2249, 1732, 1656, 1619, 1541, 1451, 1426, 1398, 1378, 1283, 1216, 1162, 1134, 1083, 1033, 991, 919, 872, 841, 803, 780, 752, 702, 612, 579, 535, 525, 504; HRMS (ESI, +ive) C_23_H_24_N_4_O_3_ theoretical mass 404.1848, experimental exact mass 404.1858 (mass error 2.31 ppm).

## **(*S*)-*N*-(1-amino-3-methyl-1-oxobutan-2-yl)-1-(4-fluorobutyl)-1*H*-indole-3-carboxamide (25, AB-4F-BUTICA).**

Subjecting 1-(4-fluorobutyl)-1*H*-indole-3-carboxylic acid (**45**, 94 mg, 0.40 mmol) and (*S*)-2-amino-3-methylbutanamide hydrochloride (65 mg, 0.43 mmol) to general procedure A furnished, following purification by flash chromatography (CH_2_Cl_2_:MeOH 100:0 to 90:10), **25** as a white powder (50 mg, 38%). R*_f_* 0.17 (hexane:EtOAc 1:4); m.p. 225–227 °C; ^1^H NMR (400 MHz, DMSO-*d*_6_) *δ* 8.27 (s, 1H), 8.12 – 8.08 (m, 1H), 7.57 – 7.54 (m, 1H), 7.50 (d, *J* = 8.9 Hz, 1H), 7.47 (brs, 1H), 7.25 – 7.16 (m, 1H), 7.18 – 7.10 (m, 1H), 7.05 (brs, 1H), 4.47 (dt, *J* = 47.4, 6.0 Hz, 3H), 4.34 (dd, *J* = 8.9, 7.0 Hz, 1H), 4.25 (td, *J* = 7.0, 2.7 Hz, 2H), 2.15 – 2.02 (m, 1H), 1.96 – 1.84 (m, 2H), 1.74 – 1.58 (m, 2H), 0.93 (dd, *J* = 6.8, 2.9 Hz, 6H); ^13^C NMR (101 MHz, DMSO-*d*_6_) *δ* 173.6, 164.0, 136.1, 131.3, 126.5, 121.9, 121.1, 120.7, 110.3, 109.5, 83.4 (d, ^1^*J*_CF_ = 161.8 Hz), 57.4, 45.4, 30.4, 27.2 (d, ^2^*J*_CF_ = 19.6 Hz), 25.6 (d, ^3^*J*_CF_ = 5.0 Hz), 19.5, 18.5; ѵ_max_ (cm^-1^): 3367, 3288, 3194, 2957, 1649, 1620, 1530, 1515, 1468, 1433, 1395, 1343, 1302, 1269, 1236, 1172, 1155, 1134, 1054, 1013, 975, 940, 876, 838, 805, 780, 766, 741, 670, 616, 565, 540, 511; HRMS (ESI, +ive) C_18_H_24_FN_3_O_2_ theoretical mass 333.1853 experimental exact mass 333.1848 (mass error 1.3 ppm).

## **(*S*)-*N*-(1-amino-3,3-dimethyl-1-oxobutan-2-yl)-1-(4-fluorobutyl)-1*H*-indole-3-carboxamide (26, ADB-4F-BUTICA).**

Subjecting 1-(4-fluorobutyl)-1*H*-indole-3-carboxylic acid (**45**, 94 mg, 0.42 mmol) and (*S*)-2-amino-3,3-dimethylbutanamide hydrochloride (69 mg, 0.42 mmol) to general procedure A furnished, following purification by flash chromatography (CH_2_Cl_2_:MeOH 100:0 to 90:10), **26** as a white powder (87 mg, 63%). R*_f_* 0.28 (hexane:EtOAc 1:4); m.p. 92–94 °C; ^1^H NMR (400 MHz, DMSO-*d*_6_) *δ* 8.31 (s, 1H), 8.07 (d, *J* = 7.8 Hz, 1H), 7.77 – 7.50 (m, 2H), 7.25 – 7.13 (m, 3H), 7.10 (brs, 1H), 4.47 (d, *J* = 9.5 Hz, 1H), 4.46 (dt, *J* = 47.4, 6.0 Hz, 2H), 4.30 – 4.21 (m, 2H), 1.90 (quin., *J* = 7.2 Hz, 2H), 1.73 – 1.57 (m, 2H), 1.01 (s, 9H); ^13^C NMR (101 MHz, CDCl_3_) *δ* 173.3, 165.1, 136.7, 131.6, 125.6, 122.8, 121.9, 120.5, 110.9, 110.4, 83.6 (d, ^1^*J*_CF_ = 165.7 Hz), 59.9, 46.6, 34.9, 27.8 (d, ^2^*J*_CF_ = 20.1 Hz), 27.0, 26.4 (d, ^3^*J*_CF_ = 4.0 Hz); ѵ_max_ (cm^-1^): 3185, 2960, 1613, 1535, 1464, 1394, 1231, 1180, 1122, 1012, 745, 520; HRMS (ESI, +ive) C_19_H_26_FN_3_O_2_ theoretical mass 347.2009 experimental exact mass 347.2002 (mass error 1.9 ppm).

## **(*S*)-*N*-(1-amino-1-oxo-3-phenylpropan-2-yl)-1-(4-fluorobutyl)-1*H*-indole-3-carboxamide (27, APP-4F-BUTICA).**

Subjecting 1-(4-fluorobutyl)-1*H*-indole-3-carboxylic acid (**45**, 94 mg, 0.40 mmol) and (*S*)-2-amino-3-phenylpropanamide hydrochloride (85 mg, 0.43 mmol) to general procedure A furnished, following purification by flash chromatography (CH_2_Cl_2_:MeOH 100:0 to 90:10), **27** as a white powder (99 mg, 65%). R*_f_* 0.17 (hexane:EtOAc 1:4); m.p. 102–104 °C; ^1^H NMR (400 MHz, CDCl_3_) *δ* 7.78 (d, *J* = 7.9 Hz, 1H), 7.70 (s, 1H), 7.40 – 7.19 (m, 6H), 6.67 (s, 2H), 6.19 (brs, 1H), 5.42 (brs, 1H), 4.98 (q, *J* = 6.8 Hz, 1H), 4.45 (dt, *J* = 47.2, 5.7 Hz, 2H), 4.18 (t, *J* = 7.0 Hz, 2H), 3.35 (dd, *J* = 13.9, 6.2 Hz, 1H), 3.20 (dd, *J* = 13.9, 7.6 Hz, 1H), 2.00 (quin., *J* = 7.2 Hz, 2H), 1.79 – 1.59 (m, 2H); ^13^C NMR (101 MHz, DMSO-*d*_6_) *δ* 173.8, 164.0, 138.6, 136.0, 131.1, 129.2, 128.0, 126.5, 126.1, 121.9, 121.1, 120.6, 110.3, 109.4, 83.4 (d, ^1^*J*_CF_ = 161.8 Hz), 53.8, 45.4, 37.6, 27.2 (d, ^2^*J*_CF_ = 19.6 Hz), 25.6 (d, ^3^*J*_CF_ = 5.1 Hz); ѵ_max_ (cm^-1^): 3310, 1671, 1614, 1536, 1464, 1393, 1273, 1234, 1179, 1123, 1013, 745, 699, 562; HRMS (ESI, +ive) C_22_H_24_FN_3_O_2_ theoretical mass 381.1853 experimental exact mass 381.1845 (mass error 1.8 ppm).

## **(*S*)-*N*-(1-amino-3-methyl-1-oxobutan-2-yl)-1-(4-fluorobutyl)-1*H*-indazole-3-carboxamide (28, AB-4F-BUTINACA).**

Subjecting 1-(4-fluorobutyl)-1*H*-indazole-3-carboxylic acid (**51**, 119 mg, 0.50 mmol) and (*S*)-2-amino-3-methylbutanamide hydrochloride (84 mg, 0.55 mmol) to general procedure A furnished, following purification by flash chromatography (CH_2_Cl_2_:MeOH 100:0 to 97:3), **28** as a white powder (154 mg, 91%). R*_f_* 0.56 (CH_2_Cl_2_:MeOH 9:1); m.p. 188–190 °C; ^1^H NMR (400 MHz, CD_3_OD) *δ* 8.21 (d, *J* = 8.2 Hz, 1H), 7.66 (d, *J* = 8.6 Hz, 1H), 7.47 (t, *J* = 7.7 Hz, 1H), 7.29 (t, *J* = 7.6 Hz, 1H), 4.59 – 4.48 (m, 4H), 4.39 (t, *J* = 5.9 Hz, 1H), 2.21 (h, *J* = 6.8 Hz, 1H), 2.09 (quin., *J* = 7.3 Hz, 2H), 1.81 – 1.62 (m, 2H); ^13^C NMR (101 MHz, CD_3_OD) *δ* 176.2, 164.5, 142.5, 137.7, 128.1, 123.9, 123.9, 123.0, 111.0, 84.4 (d, ^1^*J*_CF_ = 164.1 Hz), 59.0, 49.9, 32.8, 28.8 (d, ^2^*J*_CF_ = 20.1 Hz), 26.9 (d, ^3^*J*_CF_ = 4.8 Hz), 19.8, 18.5 ѵ_max_ (cm^-1^): 3435, 3284, 2952, 1664, 1632, 1539, 1478, 1447, 1407, 1324, 1295, 1235, 1176, 1053, 1020, 935, 891, 807, 771, 744, 597, 518; HRMS (ESI, +ive) C_17_H_23_FN_4_O_2_ theoretical mass 334.1805 experimental exact mass 334.1812 (mass error 2.01 ppm).

## **(*S*)-*N*-(1-amino-3,3-dimethyl-1-oxobutan-2-yl)-1-(4-fluorobutyl)-1*H*-indazole-3-carboxamide (29, ADB-4F-BUTINACA).**

Subjecting 1-(4-fluorobutyl)-1*H*-indazole-3-carboxylic acid (**51**, 116 mg, 0.50 mmol) and (*S*)-2-amino-3,3-dimethylbutanamide hydrochloride (88 mg, 0.53 mmol) to general procedure A furnished, following purification by flash chromatography (CH_2_Cl_2_:MeOH 100:0 to 90:10), **29** as a white powder (156 mg, 91%). R*_f_* 0.61 (CH_2_Cl_2_:MeOH 9:1); m.p. 77–79 °C; ^1^H NMR (400 MHz, CD_3_OD) *δ* 8.19 (d, *J* = 8.4 Hz, 1H), 7.64 (d, *J* = 8.5 Hz, 1H), 7.45 (t, *J* = 8.2, 7.1 Hz, 1H), 7.27 (t, *J* = 7.5 Hz, 1H), 4.58 – 4.49 (m, 3H), 4.42 (dt, *J* = 47.4, 5.8 Hz, 2H), 2.07 (quin., *J* = 7.3 Hz, 2H), 1.79 – 1.57 (m, 2H), 1.08 (s, 9H); ^13^C NMR (101 MHz, CDCl_3_) *δ* 173.0, 162.7, 141.0, 136.9, 127.0, 123.0, 122.9, 122.7, 109.4, 83.6 (d, ^1^*J*_CF_ = 165.4 Hz), 59.8, 49.1, 34.8, 27.8 (d, ^2^*J*_CF_ = 20.1 Hz), 26.9, 26.0 (d, ^3^*J*_CF_ = 4.4 Hz); ѵ_max_ (cm^-1^): 3347, 2962, 1650, 1526, 1490, 1366, 1316, 1214, 1174, 1132, 1000, 776, 749, 595; HRMS (ESI, +ive) C_18_H_25_FN_4_O_2_ theoretical mass 348.1962 experimental exact mass 348.1970 (mass error 2.42 ppm).

## **(*S*)-*N*-(1-amino-1-oxo-3-phenylpropan-2-yl)-1-(4-fluorobutyl)-1*H*-indazole-3-carboxamide (30, APP-4F-BUTINACA).**

Subjecting 1-(4-fluorobutyl)-1*H*-indazole-3-carboxylic acid (**51**, 80 mg, 0.34 mmol) and (*S*)-2-amino-3-phenylpropanamide hydrochloride (71 mg, 0.36 mmol) to general procedure A furnished, following purification by flash chromatography (CH_2_Cl_2_:MeOH 100:0 to 93:7), **30** as a white powder (128 mg, 99%). R*_f_* 0.26 (hexane:EtOAc 1:1); m.p. 100–103 °C; ^1^H NMR (400 MHz, DMSO-*d*_6_) *δ* 8.11 (dt, *J* = 8.2, 1.0 Hz, 1H), 7.93 (d, *J* = 8.3 Hz, 1H), 7.78 (dt, *J* = 8.6, 0.9 Hz, 1H), 7.68 – 7.59 (brs, 1H), 7.50 – 7.37 (m, 1H), 7.32 – 7.11 (m, 6H), 4.75 (td, *J* = 8.2, 5.0 Hz, 1H), 4.62 – 4.46 (m, 2H), 4.45 (dt, *J* = 47.4, 5.9 Hz, 2H), 3.16 (dd, *J* = 13.8, 5.1 Hz, 1H), 3.08 (dd, *J* = 13.8, 8.1 Hz, 1H), 2.00 – 1.87 (m, 2H), 1.74 – 1.54 (m, 2H); ^13^C NMR (101 MHz, DMSO-*d*_6_) *δ* 172.6, 161.2, 140.5, 137.6, 136.5, 129.3, 128.0, 126.6, 126.3, 122.47, 122.0, 121.7, 110.4, 83.4 (d, ^1^*J*_CF_ = 161.8 Hz), 53.2, 48.2, 37.7, 27.1 (d, ^2^*J*_CF_ = 19.6 Hz), 25.4 (d, ^3^*J*_CF_ = 5.2 Hz); ѵ_max_ (cm^-1^): 3346, 1691, 1645, 1529, 1490, 1393, 1175, 1030, 775, 745, 699, 566; HRMS (ESI, +ive) C_21_H_23_FN_4_O_2_ theoretical mass 382.1805 experimental exact mass 382.1812 (mass error 1.83 ppm).

## **(*S*)-*N*-(1-amino-3-methyl-1-oxobutan-2-yl)-1-(4-fluorobutyl)-1*H*-pyrrolo[2,3-*b*]pyridine-3-carboxamide (31, AB-4F-BUT7AICA).**

Subjecting 1-(4-fluorobutyl)-1*H*-pyrrolo[2,3-*b*]pyridine-3-carboxylic acid (**53**, 71 mg, 0.30 mmol) and (*S*)-2-amino-3-methylbutanamide hydrochloride (50 mg, 0.32 mmol) to general procedure A furnished, following purification by flash chromatography (CH_2_Cl_2_:MeOH 100:0 to 97:3), **31** as a white powder (80 mg, 80%). R*_f_* 0.29 (hexane:EtOAc 1:1); low melting solid; ^1^H NMR (400 MHz, CD_3_OD) *δ* 8.45 (dd, *J* = 8.0, 1.6 Hz, 1H), 8.31 (dd, *J* = 4.8, 1.6 Hz, 1H), 8.22 (s, 1H), 7.23 (dd, *J* = 7.9, 4.8 Hz, 1H), 4.45 (d, *J* = 7.4 Hz, 1H), 4.43 (dt, *J* = 47.5, 6.0 Hz, 4H), 2.26 – 2.11 (m, 1H), 2.08 – 1.96 (m, 2H), 1.79 – 1.61 (m, 2H), 1.06 (dd, *J* = 6.8, 1.8 Hz, 6H); ^13^C NMR (101 MHz, CD_3_OD) *δ* 176.8, 166.8, 148.6, 144.5, 132.6, 131.2, 120.8, 118.6, 109.5, 84.4 (d, ^1^*J*_CF_ = 164.2 Hz), 59.8, 45.7, 32.0, 28.8 (d, ^2^*J*_CF_ =20.1 Hz), 27.4 (d, ^3^*J*_CF_ = 4.8 Hz), 19.9, 19.0; ѵ_max_ (cm^-1^): 3286, 1650, 1622, 1532, 1426, 1313,1268, 1233, 1175, 1137, 1058, 1023, 800, 777, 752, 674, 570; HRMS (ESI, +ive) C_17_H_23_FN_4_O_2_ theoretical mass 334.1805 experimental exact mass 334.1810 (mass error 1.51 ppm).

## **(*S*)-*N*-(1-amino-3,3-dimethyl-1-oxobutan-2-yl)-1-(4-fluorobutyl)-1*H*-pyrrolo[2,3-*b*]pyridine-3-carboxamide (32, ADB-4F-BUT7AICA).**

Subjecting 1-(4-fluorobutyl)-1*H*-pyrrolo[2,3-*b*]pyridine-3-carboxylic acid (**53**, 71 mg, 0.30 mmol) and (*S*)-2-amino-3,3-dimethylbutanamide hydrochloride (54 mg, 0.32 mmol) to general procedure A furnished, following purification by flash chromatography (CH_2_Cl_2_:MeOH 99:1 to 96:4), **32** as a white powder (79 mg, 75%). R*_f_* 0.40 (hexane:EtOAc 1:1); m.p. 95–96 °C; ^1^H NMR (400 MHz, CDCl_3_) *δ* 8.43 – 8.31 (m, 2H), 7.84 (s, 1H), 7.23 (dd, *J* = 7.9, 4.8 Hz, 1H), 6.70 (d, *J* = 9.1 Hz, 1H), 6.21 (brs, 1H), 5.68 (brs, 1H), 4.64 (d, *J* = 9.2 Hz, 1H), 4.44 (dt, *J* = 47.3, 5.8 Hz, 2H), 4.44 – 4.36 (m, 2H), 2.05 (quin., *J* = 7.3 Hz, 2H), 1.88 – 1.61 (m, 2H), 1.14 (s, 9H); ^13^C NMR (101 MHz, CD_3_OD) *δ* 175.6, 166.4, 148.7, 144.6, 132.7, 131.0, 120.6, 118.6, 109.6, 84.4 (d, ^1^*J*_CF_ = 164.2 Hz), 61.5, 35.4, 28.8 (d, ^2^*J*_CF_ = 20.1 Hz), 27.4 (d, ^3^*J*_CF_ = 4.8 Hz), 27.3; ѵ_max_ (cm^-1^): 3194, 2960, 1674, 1619, 1535, 1427, 1398, 1260, 1179, 1135, 801, 775, 749, 615; HRMS (ESI, +ive) C_18_H_25_FN_4_O_2_ theoretical mass 348.1962 experimental exact mass 348.1968 (mass error 1.85 ppm).

## **(*S*)-*N*-(1-amino-1-oxo-3-phenylpropan-2-yl)-1-(4-fluorobutyl)-1H-pyrrolo[2,3-*b*]pyridine-3-carboxamide (33, APP-4F-BUT7AICA).**

Subjecting 1-(4-fluorobutyl)-1*H*-pyrrolo[2,3-*b*]pyridine-3-carboxylic acid (**53**, 81 mg, 0.34 mmol) and (*S*)-2-amino-3-phenylpropanamide hydrochloride (73 mg, 0.36 mmol) to general procedure A furnished, following purification by flash chromatography (CH_2_Cl_2_:MeOH 100:0 to 93:7), **33** as a white powder (116 mg, 89%). R*_f_* 0.62 (hexane:EtOAc 1:1); m.p. 153–154 °C; ^1^H NMR (300 MHz, DMSO-*d*_6_) *δ* 8.36 – 8.26 (m, 3H), 8.07 (d, *J* = 8.5 Hz, 1H), 7.54 (brs, 1H), 7.37 – 7.31 (m, 2H), 7.24 (t, *J* = 7.4 Hz, 2H), 7.21 – 7.12 (m, 2H), 7.09 (brs, 1H), 4.71 – 4.62 (m, 1H), 4.47 (dt, *J* = 47.7, 6.1 Hz, 2H), 4.35 – 4.24 (m, 2H), 3.16 – 2.89 (m, 2H), 1.99 – 1.85 (m, 2H), 1.74 – 1.55 (m, 2H); ^13^C NMR (101 MHz, DMSO-*d*_6_) *δ* 173.6, 163.4, 147.1, 143.3, 138.6, 131.1, 129.4, 129.2, 128.0, 126.2, 118.7, 117.1, 108.1, 84.2, 82.6, 53.8, 43.7, 37.5, 27.3, 27.1, 25.6, 25.6; ѵ_max_ (cm^-1^): 3205, 1677, 1606, 1572, 1538, 1424, 1397, 1304, 1272, 1177, 1139, 1030, 908, 845, 807, 784, 750, 700, 580; HRMS (ESI, +ive) C_21_H_23_FN_4_O_2_ theoretical mass 382.1805 experimental exact mass 382.1799 (mass error 1.6 ppm).

## **methyl (1-(4-fluorobutyl)-1*H*-indole-3-carbonyl)-*L*-valinate (34, MMB-4F-BUTICA).**

Subjecting 1-(4-fluorobutyl)-1*H*-indole-3-carboxylic acid (**45**, 235 mg, 1.00 mmol) and methyl (*S*)-2-amino-3-methylbutanoate hydrochloride (168 mg, 1.05 mmol) to general procedure A furnished, following purification by flash chromatography (hexane:EtOAc 90:10 to 60:40), **34** as a white powder (241 mg, 69%). R*_f_* 0.41 (hexane:EtOAc 1:1); m.p. 111–114 °C; ^1^H NMR (400 MHz, CDCl_3_) *δ* 8.05 – 7.95 (m, 1H), 7.76 (s, 1H), 7.43 – 7.35 (m, 1H), 7.33 – 7.27 (m, 2H), 6.48 (d, *J* = 8.7 Hz, 1H), 4.87 (dd, *J* = 8.7, 4.8 Hz, 1H), 4.46 (dt, *J* = 47.2, 5.7 Hz, 2H), 4.22 (t, *J* = 7.1 Hz, 2H), 3.79 (s, 3H), 2.38 – 2.24 (m, 1H), 2.03 (quin., *J* = 7.2 Hz, 2H), 1.80 – 1.64 (m, 2H), 1.04 (dd, *J* = 9.7, 6.8 Hz, 6H); ^13^C NMR (101 MHz, CD_3_OD) *δ* 174.4, 168.0, 138.0, 132.5, 128.0, 123.7, 122.4, 122.1, 111.2, 110.5, 84.5 (d, ^1^*J*_CF_ = 164.1 Hz), 59.34, 52.5, 47.3, 32.0, 28.8 (d, ^2^*J*_CF_ = 20.1 Hz), 27.3 (d, ^3^*J*_CF_ = 4.6 Hz),19.6, 19.1; ѵ_max_ (cm^-1^): 3327, 2965, 1737, 1626, 1530, 1510, 1467, 1391, 1343, 1323, 1280, 1230, 1198, 1175, 1146, 1123, 1055, 1011, 983, 940, 898, 840, 812, 780, 768, 751, 635, 612, 567, 541, 510; HRMS (ESI, +ive) C_19_H_25_FN_2_O_3_ theoretical mass 348.1849 experimental exact mass 348.1857 (mass error 2.29 ppm).

## **methyl (1-(4-fluorobutyl)-1*H*-indole-3-carbonyl)-*L*-phenylalaninate (35, MPP-4F-BUTICA).**

Subjecting 1-(4-fluorobutyl)-1*H*-indole-3-carboxylic acid (**45**, 235 mg, 1.00 mmol) and methyl (*S*)-2-amino-3-phenylpropanoate hydrochloride (216 mg, 1.05 mmol) to general procedure A furnished, following purification by flash chromatography (hexane:EtOAc 95:5 to 50:50), **35** as a white powder (381 mg, 96%). R*_f_* 0.38 (hexane:EtOAc 1:1); m.p. 124–126 °C; ^1^H NMR (400 MHz, CDCl_3_) *δ* 7.77 (d, *J* = 7.8 Hz, 1H), 7.71 (s, 1H), 7.44 – 7.36 (m, 1H), 7.34 – 7.25 (m, 4H), 7.22 – 7.15 (m, 3H), 6.41 (d, *J* = 7.5 Hz, 1H), 5.21 – 5.12 (m, 1H), 4.46 (dt, *J* = 47.2, 5.7 Hz, 2H), 4.20 (t, *J* = 7.0 Hz, 2H), 3.78 (s, 3H), 3.34 (dd, *J* = 13.8, 5.8 Hz, 1H), 3.27 (dd, *J* = 13.8, 5.3 Hz, 1H), 2.02 (quin., *J* = 7.2 Hz, 2H), 1.79 – 1.62 (m, 2H); ^13^C NMR (101 MHz, CD_3_OD) *δ* 174.2, 167.7, 138.5, 137.9, 132.5, 130.3, 129.5, 127.9, 127.7, 123.7, 122.3, 122.0, 111.2, 110.5, 84.5 (d, ^1^*J*_CF_ = 164.1 Hz), 55.3, 52.7, 47.2, 38.4, 28.8 (d, ^2^*J*_CF_ = 20.0 Hz), 27.2 (d, ^3^*J*_CF_ = 4.7 Hz); ѵ_max_ (cm^-1^): 3306, 3027, 2951, 1737, 1624, 1542, 1524, 1496, 1467, 1453, 1389, 1341, 1281, 1244, 1217, 1183, 1126, 1081, 1056, 1035, 1001, 938, 902, 886, 871, 849, 827, 747, 703, 682, 619, 570, 540, 509; HRMS (ESI, +ive) theoretical mass C_23_H_25_FN_2_O_3_ theoretical mass 396.1849 experimental exact mass 396.1856 (mass error 1.65 ppm).

## **methyl (1-(4-fluorobutyl)-1*H*-indazole-3-carbonyl)-*L*-valinate (36, MMB-4F-BUTINACA).**

Subjecting 1-(4-fluorobutyl)-1*H*-indazole-3-carboxylic acid (**51**, 237 mg, 1.00 mmol) and methyl (*S*)-2-amino-3-methylbutanoate hydrochloride (179 mg, 1.07 mmol) to general procedure A furnished, following purification by flash chromatography (hexane:EtOAc 85:15 to 80:20), **36** as a colorless oil (273 mg, 68%). R*_f_* 0.57 (hexane:EtOAc 1:1); ^1^H NMR (400 MHz, DMSO-*d*_6_) *δ* 8.13 (dt, *J* = 8.2, 1.0 Hz, 1H), 8.09 (d, *J* = 8.3 Hz, 1H), 7.81 (d, *J* = 8.6, 0.9 Hz, 1H), 7.47 (ddd, *J* = 8.5, 6.9, 1.1 Hz, 1H), 7.29 (ddd, *J* = 8.2, 6.9, 0.9 Hz, 1H), 4.56 (t, *J* = 7.2 Hz, 2H), 4.47 (dt, *J* = 47.3, 6.1 Hz, 3H), 4.45 – 4.41 (m, 1H), 3.69 (s, 3H), 2.26 (h, *J* = 6.8 Hz, 1H), 1.98 (quin., *J* = 7.3 Hz, 2H), 1.76 – 1.54 (m, 2H), 0.96 (t, *J* = 6.7 Hz, 6H); ^13^C NMR (101 MHz, CD_3_OD) *δ* 173.7, 164.6, 142.4, 137.6, 128.1, 124.0, 123.9, 123.0, 111.1, 84.4 (d, ^1^*J*_CF_ = 164.1 Hz), 58.7, 52.7, 50.0, 32.3, 28.8 (d, ^2^*J*_CF_ = 20.1 Hz), 26.9 (d, ^3^*J*_CF_ = 4.8 Hz), 19.6, 18.6; ѵ_max_ (cm^-1^): 3415, 2963, 1738, 1665, 1574, 1523, 1490, 1469, 1437, 1391, 1309, 1264, 1205, 1173, 1004, 940, 903, 814, 788, 776, 750, 669, 547; HRMS (ESI, +ive) C_18_H_24_FN_3_O_3_ theoretical mass 349.1802 experimental exact mass 349.1796 (mass error 1.6 ppm).

## **methyl (1-(4-fluorobutyl)-1*H*-indazole-3-carbonyl)-*L*-phenylalaninate (37, MPP-4F-BUTINACA).**

Subjecting 1-(4-fluorobutyl)-1*H*-indazole-3-carboxylic acid (**51**, 118 mg, 0.50 mmol) and methyl (*S*)-2-amino-3-phenylpropanoate hydrochloride (119 mg, 0.55 mmol) to general procedure A furnished, following purification by flash chromatography (hexane:EtOAc 90:10 to 80:20), **37** as a colorless oil (133 mg, 68%). R*_f_* 0.74 (hexane:EtOAc 1:1); ^1^H NMR (400 MHz, CDCl_3_) *δ* 8.34 (dt, *J* = 8.2, 1.0 Hz, 1H), 7.50 – 7.37 (m, 3H), 7.32 – 7.18 (m, 6H), 5.13 (dt, *J* = 8.2, 6.0 Hz, 1H), 4.46 (dt, *J* = 47.3, 5.9 Hz, 2H), 4.43 (t, *J* = 7.0 Hz, 2H), 3.75 (s, 3H), 3.35 – 3.18 (m, 2H), 2.15 – 2.04 (m, 2H), 1.79 – 1.62 (m, 2H); ^13^C NMR (101 MHz, CD_3_OD) *δ* 173.5, 164.4, 142.4, 138.0, 137.5, 130.4, 129.6, 128.0, 123.9, 123.9, 122.9, 111.0, 84.4 (d, ^1^*J*_CF_ = 164.0 Hz), 54.7, 52.9, 49.8, 38.5, 28.7 (d, ^2^*J*_CF_ = 20.0 Hz), 26.8 (d, ^3^*J*_CF_ = 4.8 Hz); ѵ_max_ (cm^-1^): 3409, 2953, 1739, 1660, 1574, 1524, 1490, 1444, 1405, 1349, 1320, 1207, 1172, 1134, 1077, 1004, 940, 903, 840, 788, 774, 749, 700, 564; HRMS (ESI, +ive) C_22_H_24_FN_3_O_3_ theoretical mass 397.1802 experimental exact mass 397.1810 (mass error 2.04 ppm).

## **methyl (1-(4-fluorobutyl)-1*H*-pyrrolo[2,3-*b*]pyridine-3-carbonyl)-*L*-valinate (38, MMB-4F-BUT7AICA).**

Subjecting 1-(4-fluorobutyl)-1*H*-pyrrolo[2,3-*b*]pyridine-3-carboxylic acid (**53**, 119 mg, 0.50 mmol) and methyl (*S*)-2-amino-3-methylbutanoate hydrochloride (88 mg, 0.53 mmol) to general procedure A furnished, following purification by flash chromatography (hexane:EtOAc 88:12 to 75:25), **38** as a white powder (145 mg, 83%). R*_f_* 0.36 (hexane:EtOAc 1:1); m.p. 81–82 °C; ^1^H NMR (400 MHz, DMSO-*d*_6_) *δ* 8.49 (s, 1H), 8.41 (dd, *J* = 7.9, 1.7 Hz, 1H), 8.32 (dd, *J* = 4.7, 1.6 Hz, 1H), 8.13 (d, *J* = 8.0 Hz, 1H), 7.21 (dd, *J* = 7.9, 4.7 Hz, 1H), 4.48 (dt, *J* = 47.4, 6.0 Hz, 2H), 4.39 – 4.27 (m, 3H), 3.66 (s, 3H), 2.15 (h, *J* = 6.8 Hz, 1H), 2.00 – 1.89 (m, 2H), 1.75 – 1.57 (m, 2H), 0.97 (dd, *J* = 20.1, 6.8 Hz, 6H); ^13^C NMR (101 MHz, CD_3_OD) *δ* 174.2, 167.1, 148.6, 144.6, 132.6, 131.4, 121.0, 118.6, 109.4, 84.4 (d, ^1^*J*_CF_ = 164.2 Hz), 59.5, 52.5, 45.7, 31.9, 28.8 (d, ^2^*J*_CF_ = 20.1 Hz), 27.4 (d, ^3^*J*_CF_ = 4.7 Hz), 19.6, 19.2; ѵ_max_ (cm^-1^): 3331, 2967, 1728, 1619, 1538, 1519, 1423, 1385, 1313, 1256, 1200, 1147, 1049, 984, 903, 857, 809, 785, 750, 637; HRMS (ESI, +ive) C_18_H_24_FN_3_O_3_ theoretical mass 349.1802 experimental exact mass 349.1807 (mass error 1.40 ppm).

## **methyl (*S*)-2-(1-(4-fluorobutyl)-1*H*-pyrrolo[2,3-*b*]pyridine-3-carboxamido)-3,3-dimethylbutanoate (39, MDMB-4F-BUT7AICA).**

Subjecting 1-(4-fluorobutyl)-1*H*-pyrrolo[2,3-*b*]pyridine-3-carboxylic acid (**53**, 118 mg, 0.50 mmol) and methyl (*S*)-2-amino-3,3-dimethylbutanoate hydrochloride (96 mg, 0.53 mmol) to general procedure A furnished, following purification by flash chromatography (hexane:EtOAc 85:15 to 60:40), **39** as a white powder (143 mg, 79%). R*_f_* 0.49 (hexane:EtOAc 1:1); m.p. 56–58 °C; ^1^H NMR (400 MHz, DMSO-*d*_6_) *δ* 8.62 (s, 1H), 8.40 (dd, *J* = 7.9, 1.7 Hz, 1H), 8.32 (dd, *J* = 4.7, 1.6 Hz, 1H), 7.84 (d, *J* = 8.7 Hz, 1H), 7.21 (dd, *J* = 7.9, 4.7 Hz, 1H), 4.49 (dt, *J* = 47.4, 6.0 Hz, 2H), 4.49 (d, *J* = 8.7 Hz, 1H), 4.41 – 4.27 (m, 2H), 3.66 (s, 3H), 2.03 – 1.91 (m, 2H), 1.76 – 1.59 (m, 2H), 1.04 (s, 9H); ^13^C NMR (101 MHz, CD_3_OD) *δ* 173.5, 166.8, 148.6, 144.6, 132.7, 131.3, 120.9, 118.6, 109.3, 84.4 (d, ^1^*J*_CF_ = 164.2 Hz), 61.8, 52.3, 45.7, 35.5, 28.8 (d, ^2^*J*_CF_ = 20.0 Hz), 27.4 (d, ^3^*J*_CF_ = 4.8 Hz), 27.3; ѵ_max_ (cm^-1^): 3309, 2962, 1736, 1612, 1568, 1536, 1512, 1427, 1401, 1369, 1303, 1268, 1212, 1161, 1134, 1030, 900, 809, 777, 752, 634, 566; HRMS (ESI, +ive) C_19_H_26_FN_3_O_3_ theoretical mass 363.1958 experimental exact mass 363.1952 (mass error 1.6 ppm).

## **methyl (1-(4-fluorobutyl)-1*H*-pyrrolo[2,3-*b*]pyridine-3-carbonyl)-*L*-phenylalaninate (40, MPP-4F-BUT7AICA).**

Subjecting 1-(4-fluorobutyl)-1*H*-pyrrolo[2,3-*b*]pyridine-3-carboxylic acid (**53**, 118 mg, 0.50 mmol) and methyl (*S*)-2-amino-3-phenylpropanoate hydrochloride (129 mg, 0.60 mmol) to general procedure A furnished, following purification by flash chromatography (hexane:EtOAc 90:10 to 75:25), **40** as a white powder (175 mg, 88%). R*_f_* 0.67 (hexane:EtOAc 1:4); m.p. 109–112 °C; ^1^H NMR (400 MHz, CDCl_3_) *δ* 8.37 (dd, *J* = 4.8, 1.5 Hz, 1H), 8.15 (dd, *J* = 8.0, 1.6 Hz, 1H), 7.77 (s, 1H), 7.34 – 7.26 (m, 4H), 7.21 – 7.14 (m, 3H), 6.32 (d, *J* = 7.5 Hz, 1H), 5.19 – 5.11 (m, 1H), , 4.45 (dt, *J* = 47.3, 5.9 Hz, 2H), 4.38 (d, *J* = 7.1 Hz, 2H), 3.79 (s, 3H), 3.33 (dd, *J* = 13.8, 5.8 Hz, 1H), 3.26 (dd, *J* = 13.8, 5.3 Hz, 1H), 2.10 – 1.98 (m, 2H), 1.80 – 1.63 (m, 2H); ^13^C NMR (101 MHz, CD_3_OD) *δ* 173.5, 164.4, 142.4, 138.0, 137.5, 130.4, 129.6, 128.0, 123.9, 123.9, 122.9, 111.0, 84.4 (d, ^1^*J*_CF_ = 164.0 Hz), 54.7, 52.9, 49.8, 38.5, 28.7 (d, ^2^*J*_CF_ = 20.0 Hz), 26.8 (d, ^3^*J*_CF_ = 4.8 Hz); ѵ_max_ (cm^-1^): 3291, 3029, 2949, 1737, 1621, 1548, 1523, 1496, 1450, 1427, 1400, 1378, 1285, 1225, 1181, 1139, 1082, 1033, 998, 899, 872, 839, 802, 780, 754, 702, 620, 580, 558, 541; HRMS (ESI, +ive) C_22_H_24_FN_3_O_3_ theoretical mass 397.1802 experimental exact mass 397.1807 (mass error 1.27 ppm).

## **methyl 1-(4-fluorobutyl)-1*H*-pyrrolo[2,3-*b*]pyridine-3-carboxylate (49, 4F-BUT7AICA COOMe).**

A solution of methyl 1*H*-pyrrolo[2,3-*b*]pyridine-3-carboxylate (1.76 g, 10.0 mmol) in DMF (30 mL) was cooled to 0 °C, following which NaH (60% dispersion in mineral oil, 440 mg, 11.0 mmol) was added portion wise. The mixture was allowed to warm to room temperature over 1 h, after which it was cooled to 0 °C, before 1-bromo-4-fluorobutane (1.18 mL, 11.0 mmol) was added dropwise. The solution was then warmed to room temperature and left to stir for 18 h, following which in was poured onto H_2_O (600 mL), extracted with EtOAc (3 × 100 mL), with the organic layers combined, washed with H_2_O (2 × 200 mL), brine (200 mL) and dried over Na_2_SO_4_. The solution was then concentrated *in vacuo* and subjected to flash chromatography (hexane:EtOAc 90:10 to 75:25), with the product concentrated and dried *in vacuo*, furnishing **49** as a white solid (2.02 g, 81%). R*_f_* 0.74 (hexane:EtOAc 1:1), m.p. 66–67 °C, ^1^H NMR (400 MHz, CDCl_3_) *δ* 8.46 (dd, *J* = 7.9, 1.6 Hz, 1H), 8.38 (dd, *J* = 4.8, 1.6 Hz, 1H), 7.96 (s, 1H), 7.31 – 7.22 (m, 1H), 4.46 (dt, *J* = 47.1, 5.7 Hz, 2H), 4.45 – 4.43 (m, 2H), 3.92 (s, 3H), 2.06 (quin., *J* = 7.3 Hz, 2H), 1.81 – 1.64 (m, 2H); ^13^C NMR (101 MHz, CDCl_3_) *δ* 164.9, 147.3, 143.5, 134.2, 130.8, 119.6, 118.1, 106.1, 83.6 (d, ^1^*J*_CF_ = 165.5 Hz), 51.4, 45.1, 27.7 (d, ^2^*J*_CF_ = 20.1 Hz), 26.5 (d, ^3^*J*_CF_ = 4.5 Hz); HRMS (ESI +ive) C_13_H_15_FN_2_O_2_ theoretical mass 250.1118 experimental exact mass 250.1113 (mass error 0.3 ppm).

## **1-(4-fluorobutyl)-1*H*-pyrrolo[2,3-*b*]pyridine-3-carboxylic acid (53, 4F-BUT7AICA COOH).**

To a solution of methyl 1-(4-fluorobutyl)-1*H*-pyrrolo[2,3-*b*]pyridine-3-carboxylate (684 mg, 2.73 mmol) in MeOH (50 mL) was added aqueous sodium hydroxide (1 M, 37.3 mL, 37.3 mmol), which was stirred for 48 h. Solvent was then removed *in vacuo*, H_2_O (50 mL) was added, acidified with hydrochloric acid (1 M, pH 2), extracted with EtOAc (3 × 50 mL), with the organic layers combined and dried over MgSO_4_. Solvent was then removed *in vacuo*, furnishing **53** as a white powder (633 mg, 98%). R*_f_* 0.30 (hexane:EtOAc 1:1); m.p. 213–215 °C (decomp.); ^1^H NMR (400 MHz, DMSO-*d*_6_) *δ* 12.26 (brs, 1H), 8.36 – 8.27 (m, 3H), 7.26 (dd, *J* = 7.8, 4.7 Hz, 1H), 4.41 (dt, *J* = 47.4, 6.1 Hz, 2H), 4.41 – 4.33 (m, 2H), 1.99 – 1.84 (m, 2H), 1.70 – 1.50 (m, 2H); ^13^C NMR (75 MHz, DMSO-*d*_6_) *δ* 165.2, 147.4, 143.5, 135.3, 129.2, 118.8, 117.8, 105.2, 83.3 (d, ^1^*J*_CF_ = 161.9 Hz), 43.95, 27.12 (d, ^2^*J*_CF_ = 19.6 Hz), 25.51 (d, ^3^*J*_CF_ = 5.1 Hz); HRMS (ESI +ive) C_12_H_13_FN_2_O_2_ theoretical mass 236.0961 experimental exact mass 236.0956 (mass error 0.3 ppm).

# ^1^H NMR, ^13^C NMR, LC-UV and UV spectra for newly described compounds.

## **Figure S1.** ^1^H (400 MHz, DMSO-*d*_6_) and ^13^C (101 MHz, DMSO-*d*_6_) NMR spectra for (*S*)-*N*-(1-amino-3-methyl-1-oxobutan-2-yl)-1-(4-cyanobutyl)-1*H*-indole-3-carboxamide (AB-4CN-BUTICA, **5**).


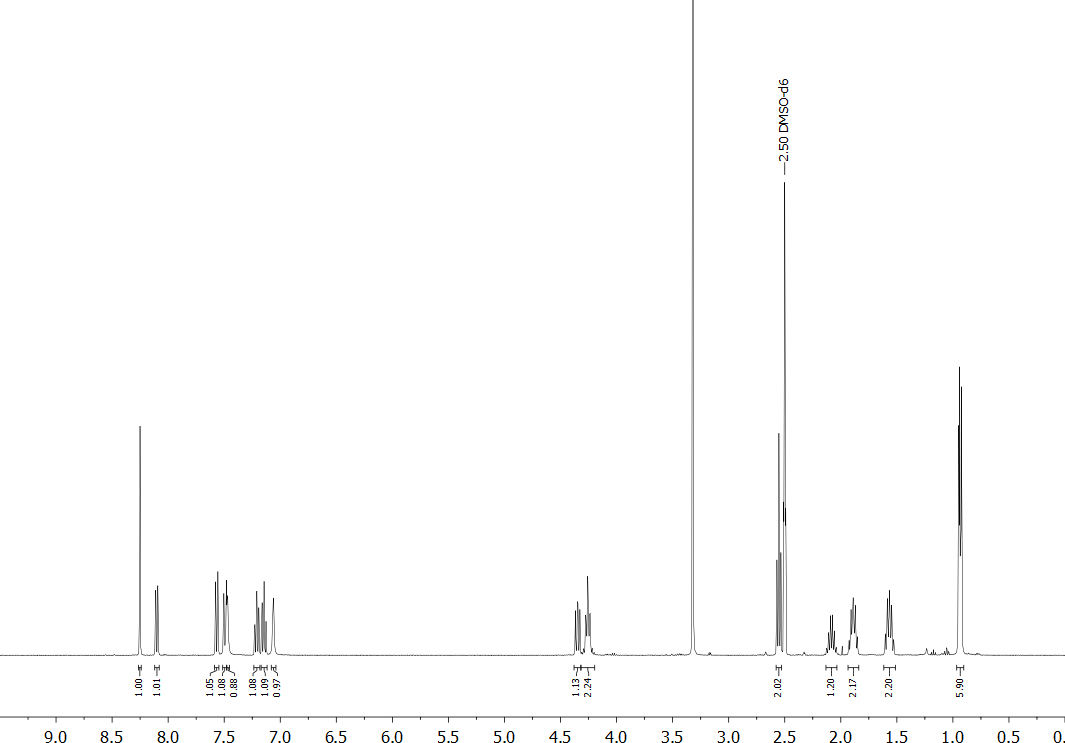


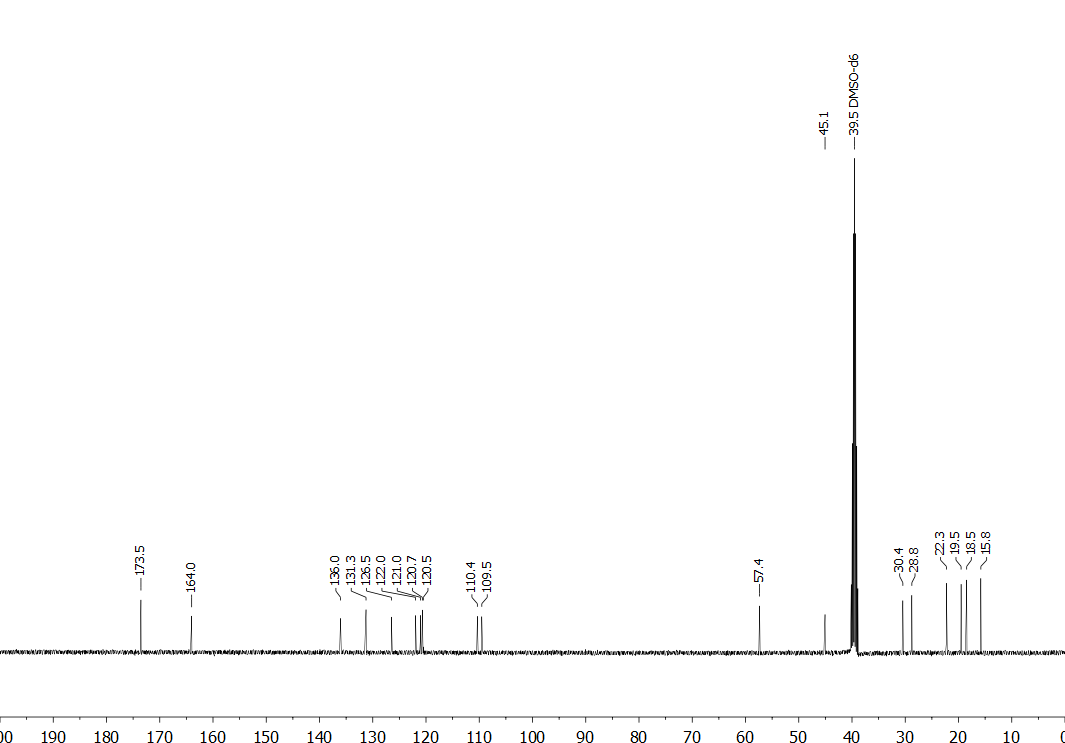


## **Figure S2.** ^1^H (400 MHz, DMSO-*d*_6_) and ^13^C (101 MHz, DMSO-*d*_6_) NMR spectra for (*S*)-*N*-(1-amino-3,3-dimethyl-1-oxobutan-2-yl)-1-(4-cyanobutyl)-1*H*-indole-3-carboxamide (ADB-4CN-BUTICA, **9**).


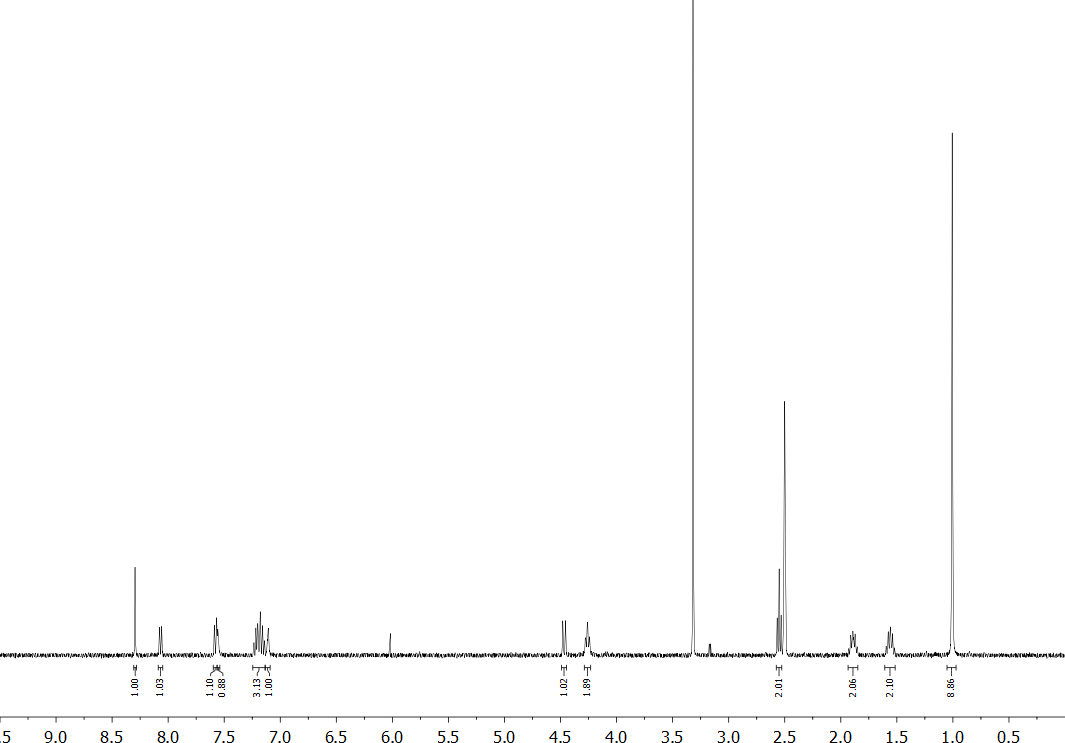


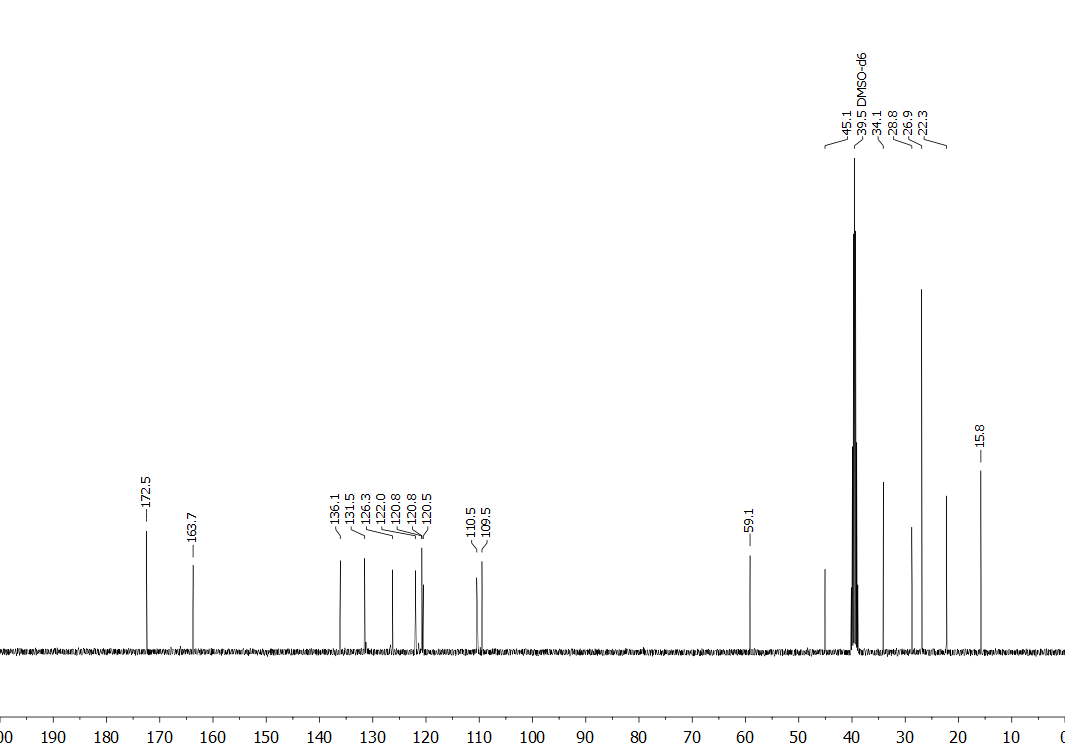


## **Figure S3.** ^1^H (400 MHz, DMSO-*d*_6_) and ^13^C (101 MHz, DMSO-*d*_6_) NMR spectra for (*S*)-*N*-(1-amino-1-oxo-3-phenylpropan-2-yl)-1-(4-cyanobutyl)-1*H*-indole-3-carboxamide (APP-4CN-BUTICA, **10**).


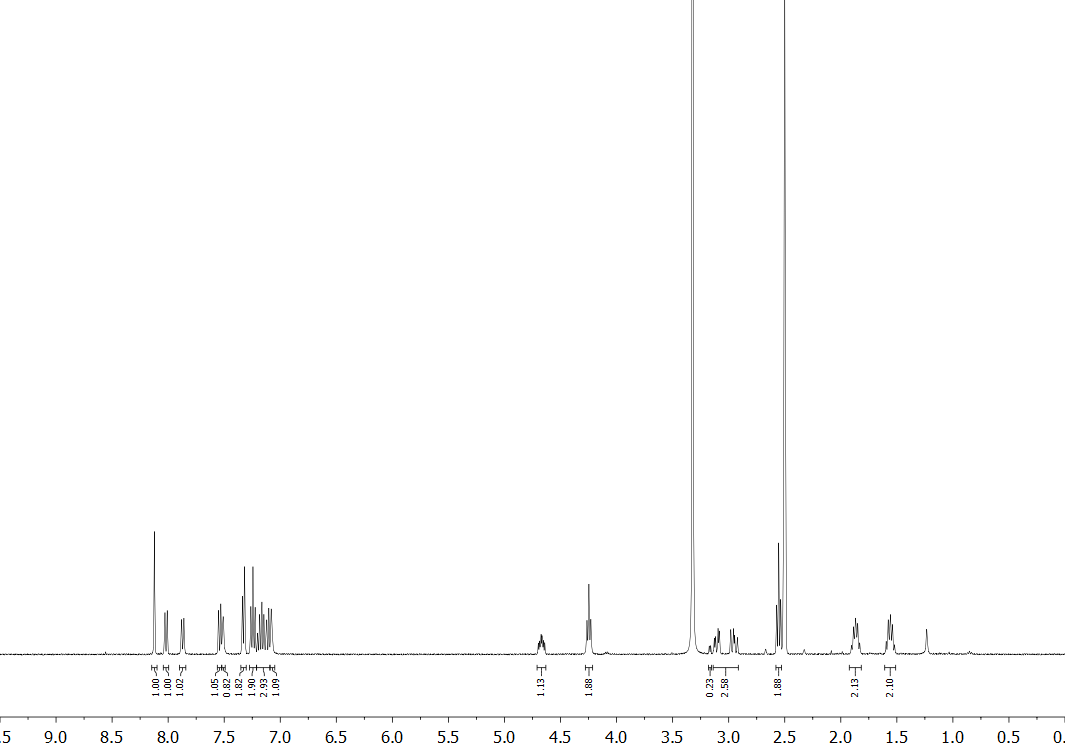


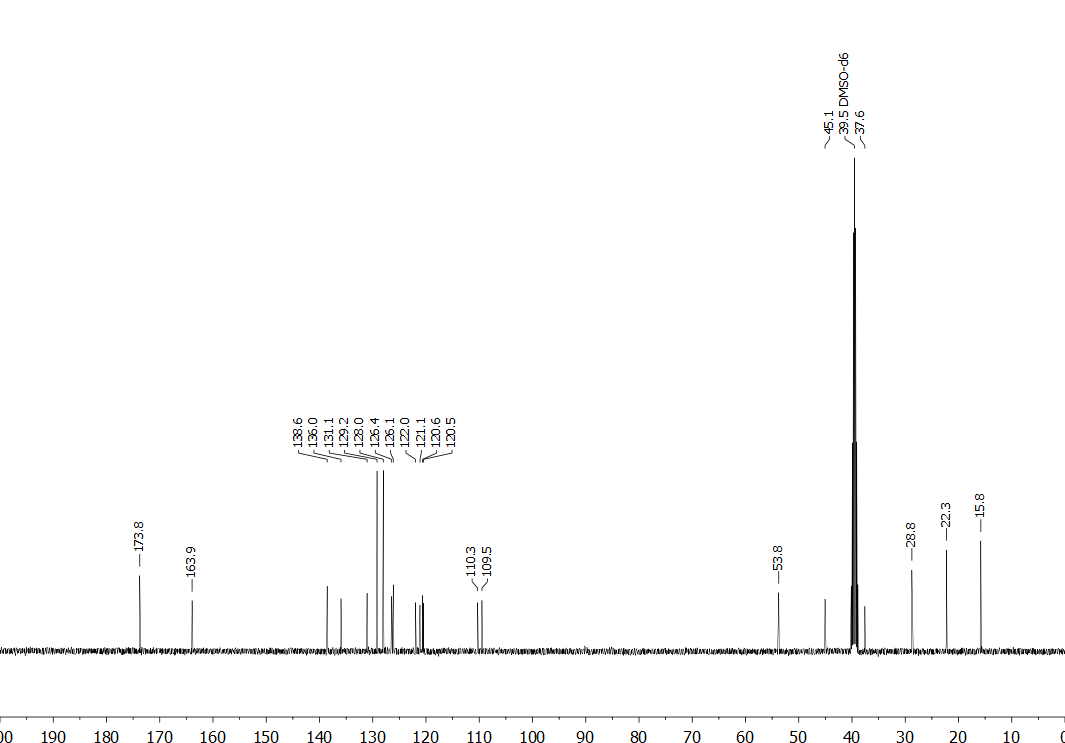


## **Figure S4.** ^1^H (400 MHz, CD_3_OD) and ^13^C (101 MHz, CD_3_OD) NMR spectra for (*S*)-*N*-(1-amino-3-methyl-1-oxobutan-2-yl)-1-(4-cyanobutyl)-1*H*-indazole-3-carboxamide (AB-4CN-BUTINACA, **11**).


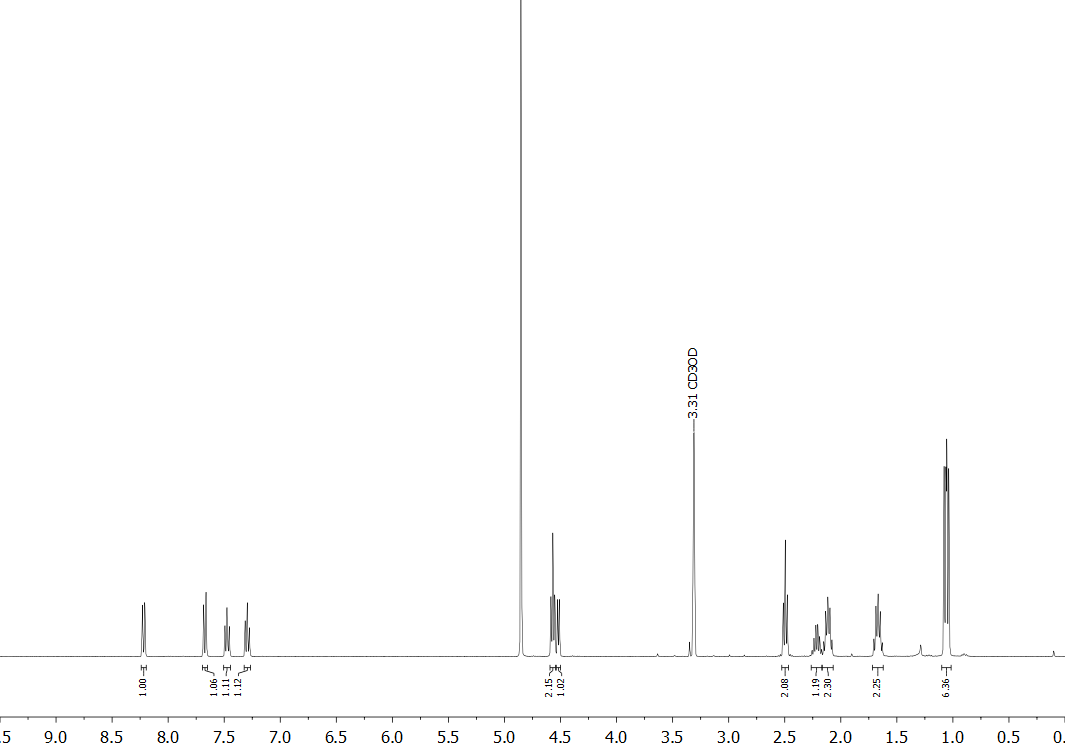


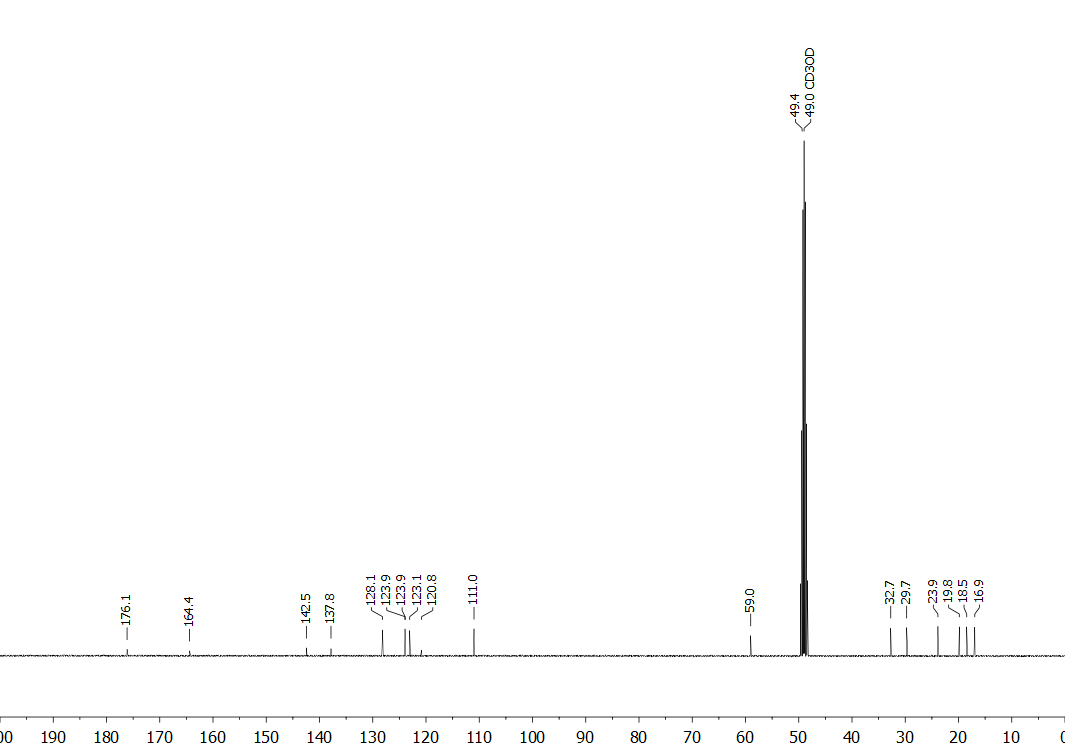


## **Figure S5.** ^1^H (400 MHz, CD_3_OD) and ^13^C (101 MHz, CD_3_OD) NMR spectra for (*S*)-*N*-(1-amino-3,3-dimethyl-1-oxobutan-2-yl)-1-(4-cyanobutyl)-1*H*-indazole-3-carboxamide (ADB-4CN-BUTINACA, **12**).


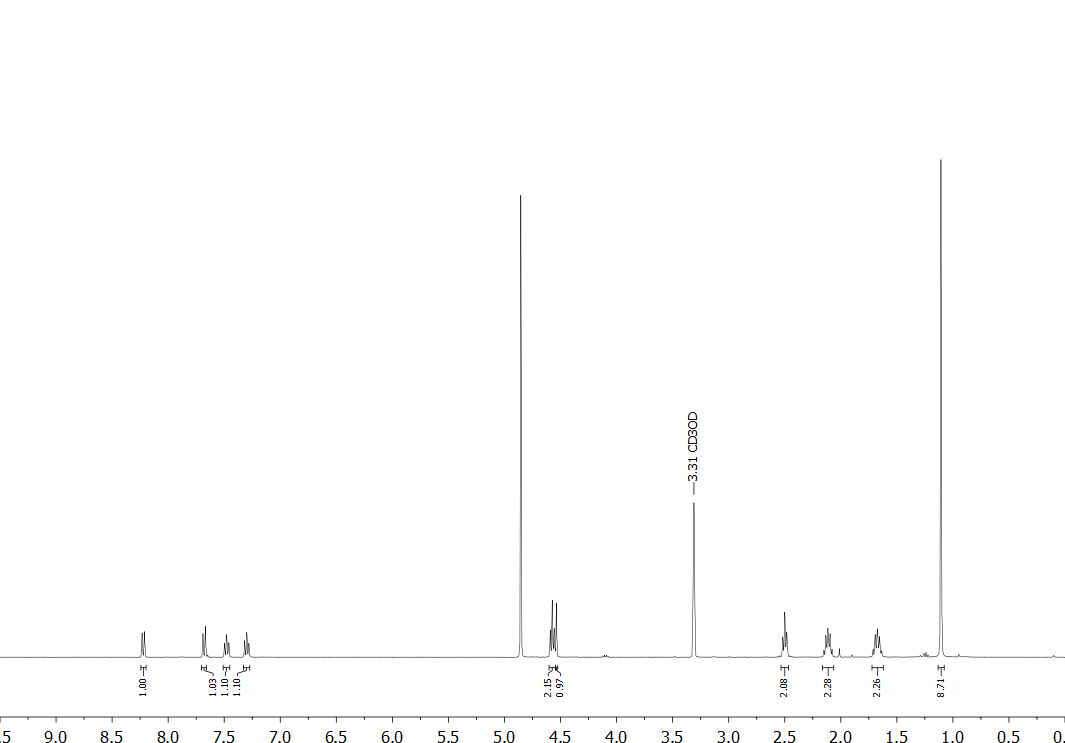


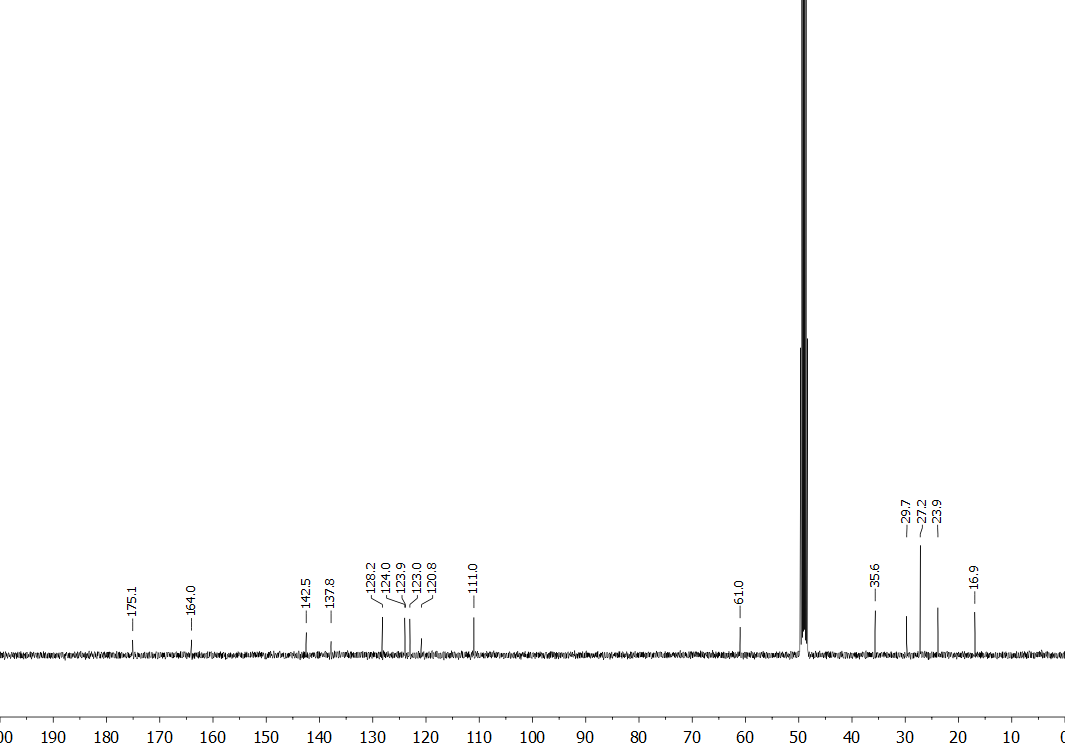


## **Figure S6.** ^1^H (400 MHz, CDCl_3_) and ^13^C (101 MHz, DMSO-*d*_6_) NMR spectra for (*S*)-*N*-(1-amino-1-oxo-3-phenylpropan-2-yl)-1-(4-cyanobutyl)-1*H*-indazole-3-carboxamide (APP-4CN-BUTINACA, **13**).


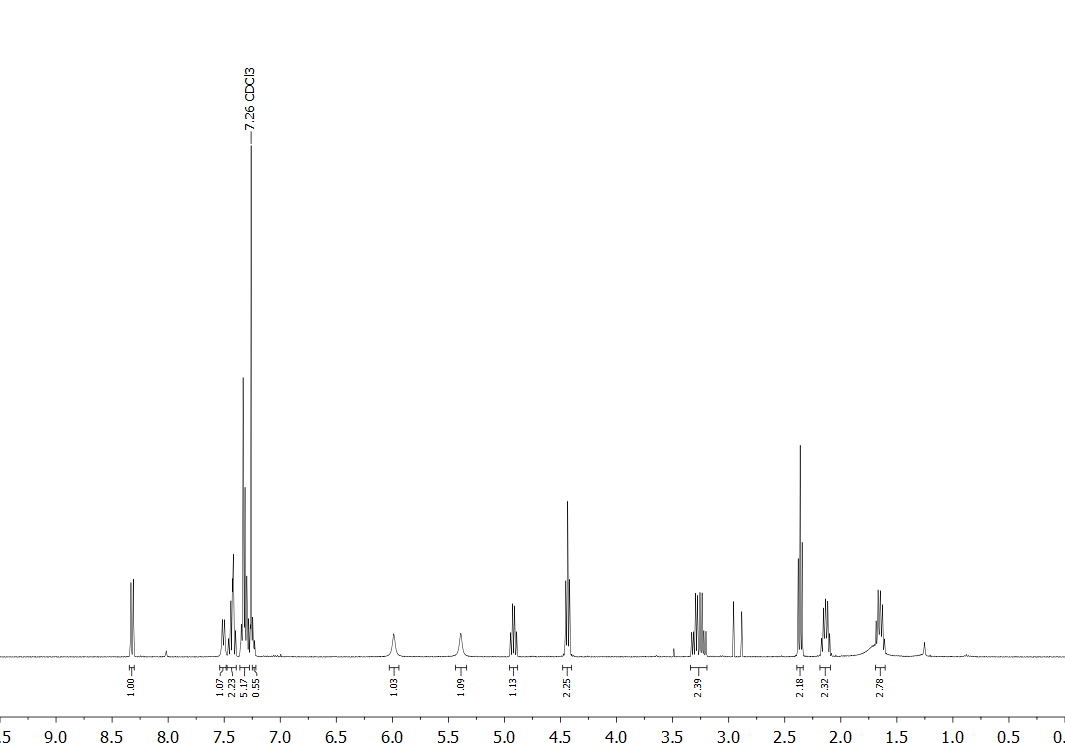


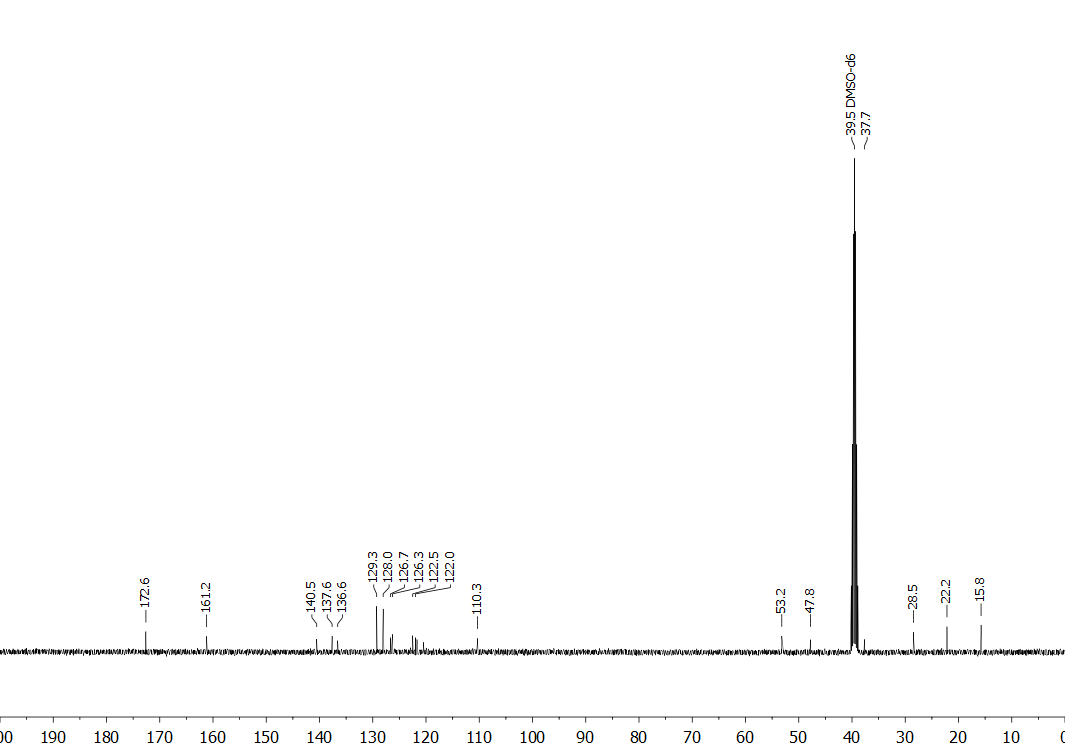


## **Figure S7.** ^1^H (400 MHz, DMSO-*d*_6_) and ^13^C (101 MHz, DMSO-*d*_6_) NMR spectra for (*S*)-*N*-(1-amino-3-methyl-1-oxobutan-2-yl)-1-(4-cyanobutyl)-1*H*-pyrrolo[2,3-*b*]pyridine-3-carboxamide (AB-4CN-BUT7AICA, **14**).


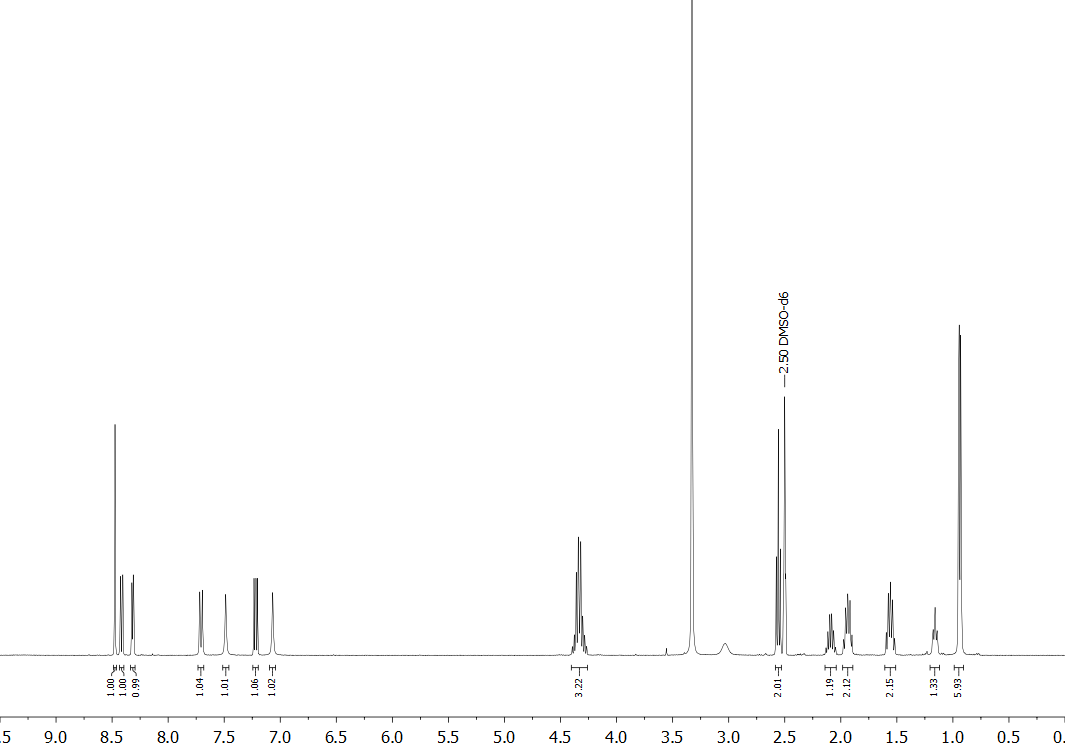


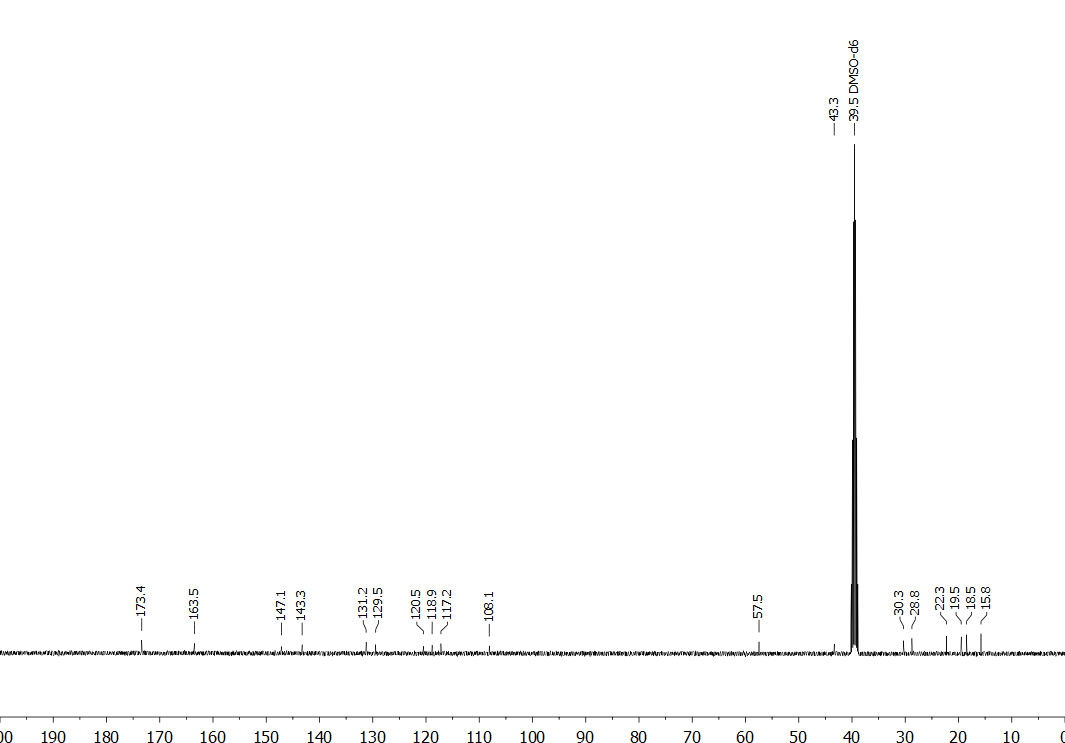


## **Figure S8.** ^1^H (400 MHz, DMSO-*d*_6_) and ^13^C (101 MHz, DMSO-*d*_6_) NMR spectra for (*S*)-*N*-(1-amino-3,3-dimethyl-1-oxobutan-2-yl)-1-(4-cyanobutyl)-1*H*-pyrrolo[2,3-*b*]pyridine-3-carboxamide (ADB-4CN-BUT7AICA, **15**).


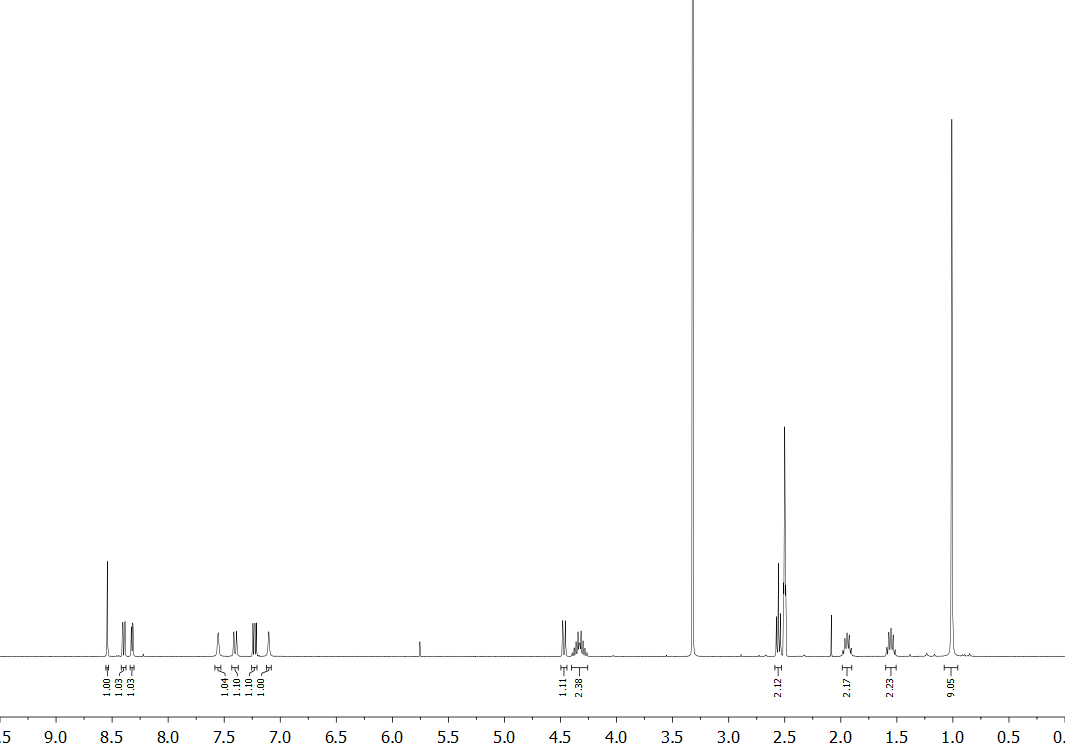


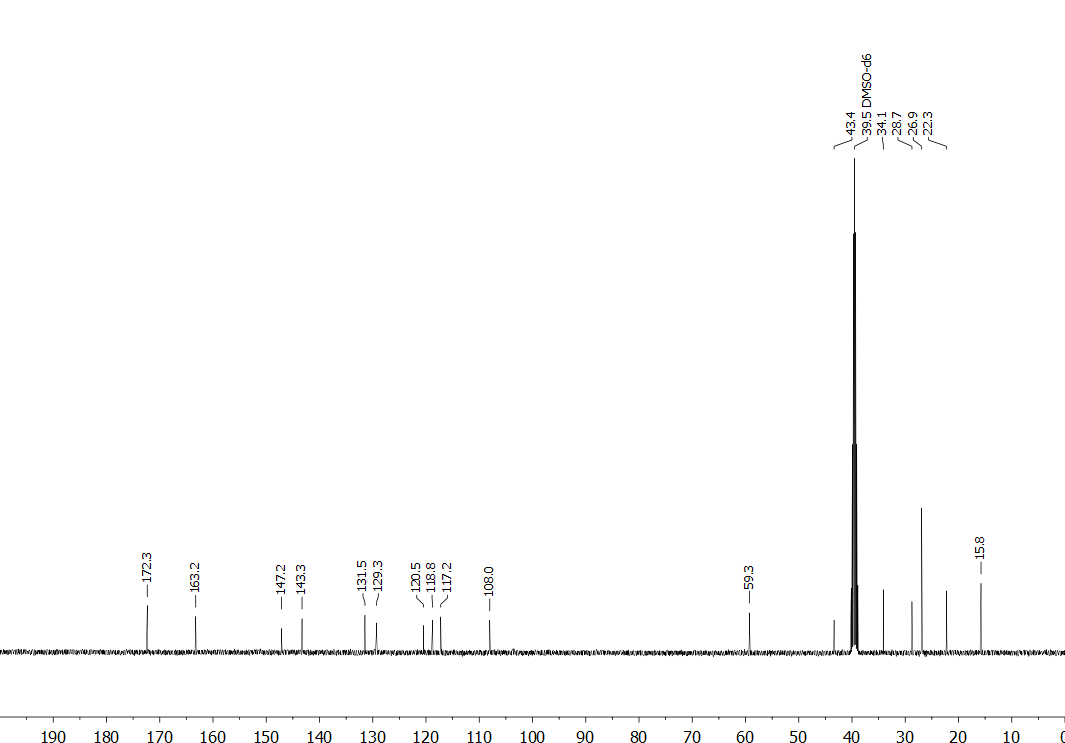


## **Figure S9.** ^1^H (400 MHz, CD_3_OD) and ^13^C (101 MHz, DMSO-*d*_6_) NMR spectra for (*S*)-*N*-(1-amino-1-oxo-3-phenylpropan-2-yl)-1-(4-cyanobutyl)-1*H*-pyrrolo[2,3-*b*]pyridine-3-carboxamide (APP-4CN-BUT7AICA, **16**).


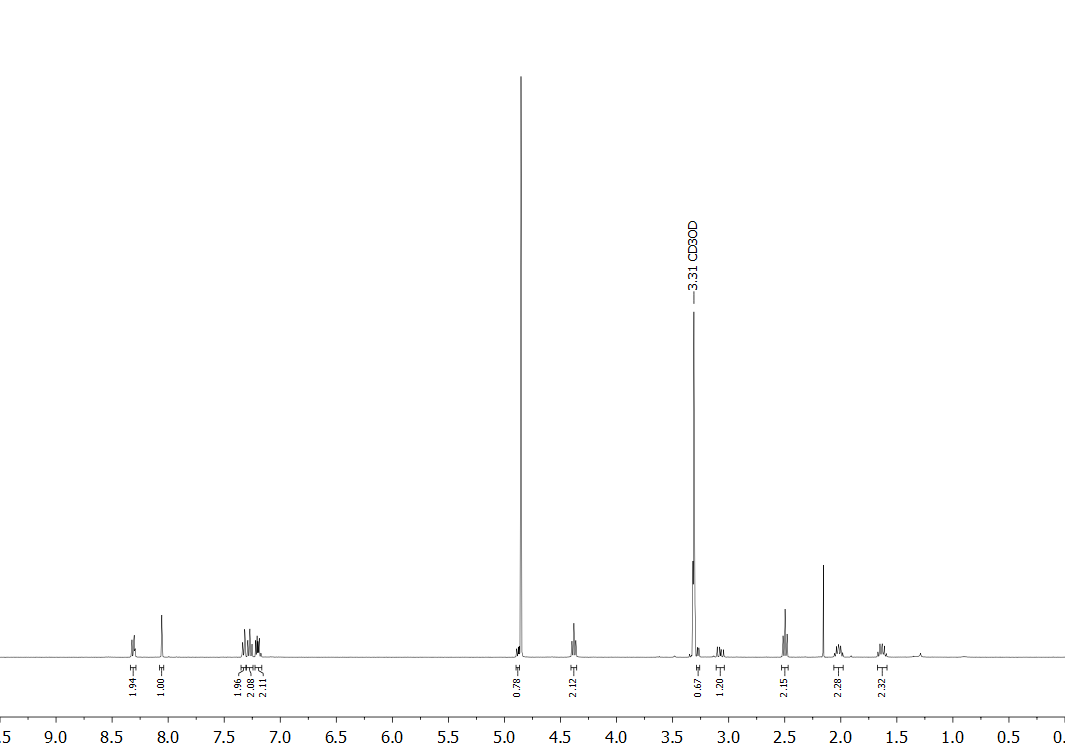


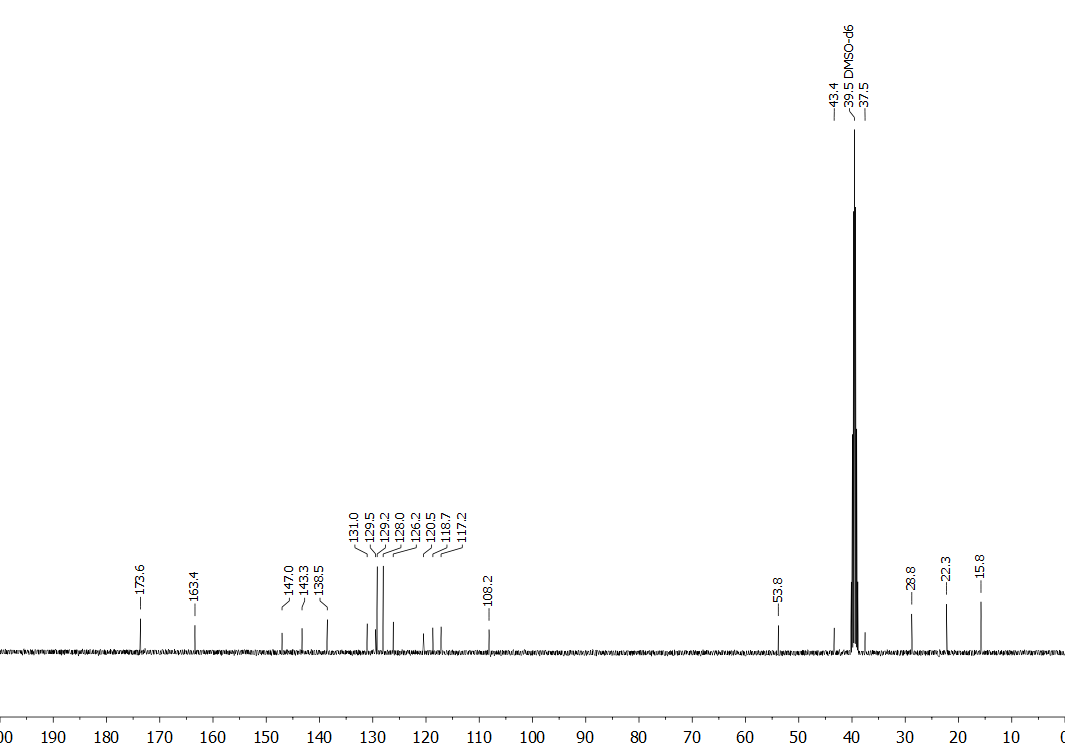


## **Figure S10.** ^1^H (400 MHz, CDCl_3_) and ^13^C (101 MHz, CD_3_OD) NMR spectra for methyl (1-(4-cyanobutyl)-1*H*-indole-3-carbonyl)-*L*-valinate (MMB-4CN-BUTICA, **17**).


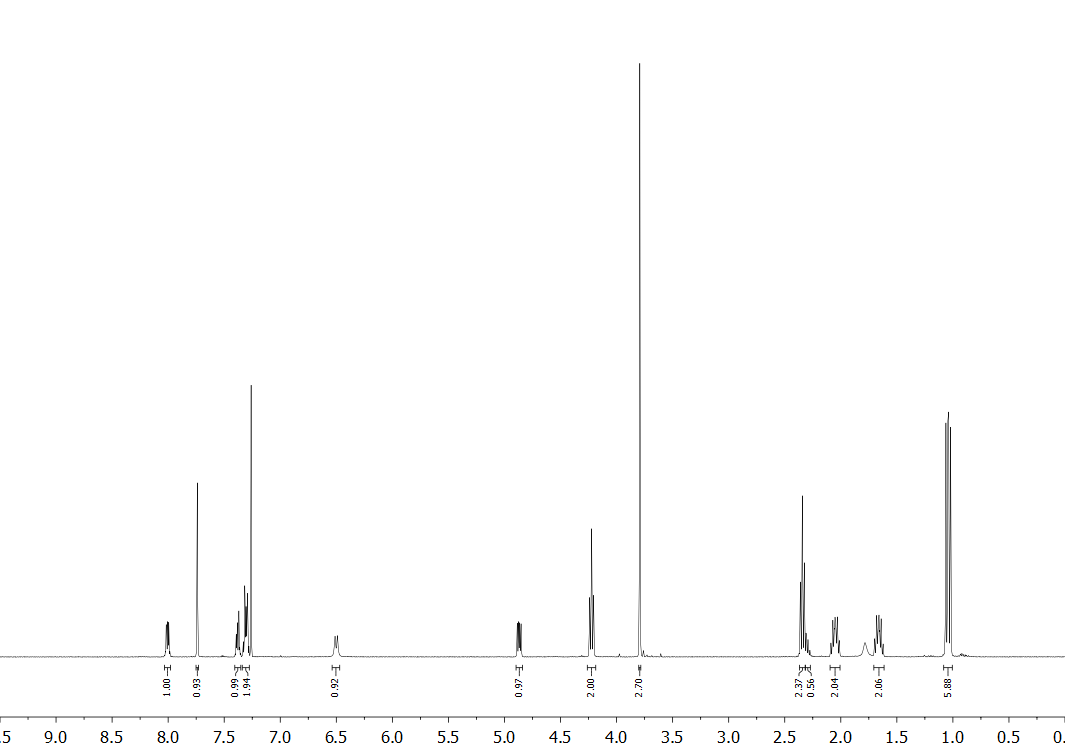


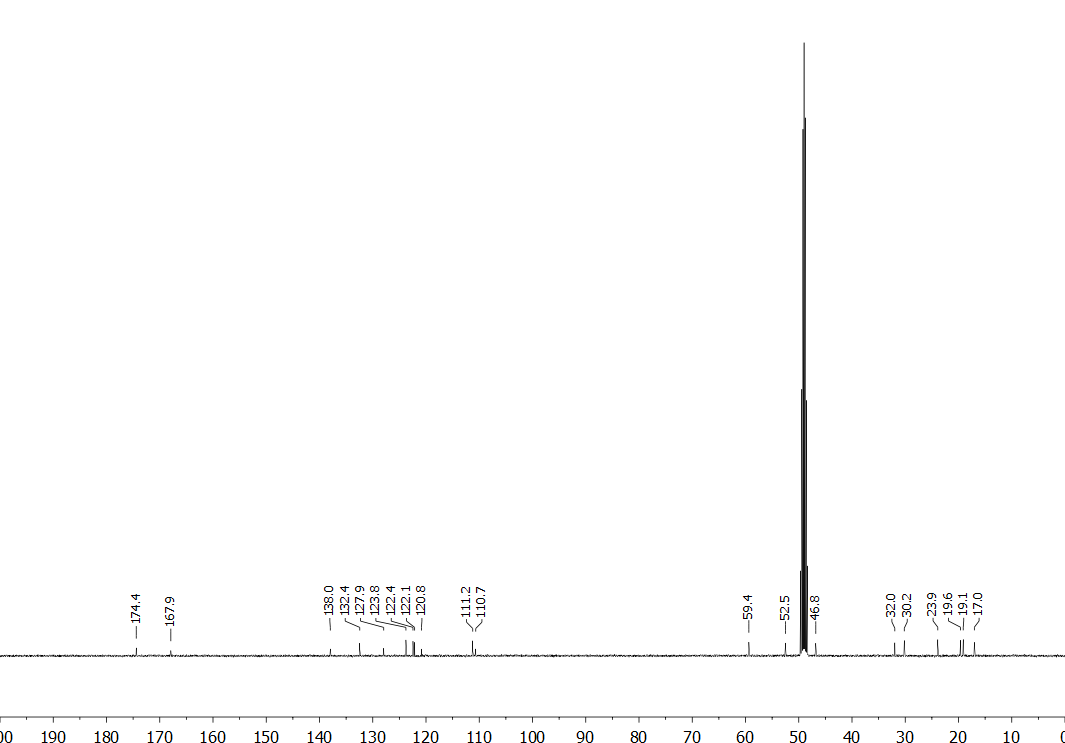


## **Figure S11.** ^1^H (400 MHz, DMSO-*d*_6_) and ^13^C (101 MHz, CD_3_OD) NMR spectra for methyl (*S*)-2-(1-(4-cyanobutyl)-1*H*-indole-3-carboxamido)-3,3-dimethylbutanoate (MDMB-4CN-BUTICA, **18**).


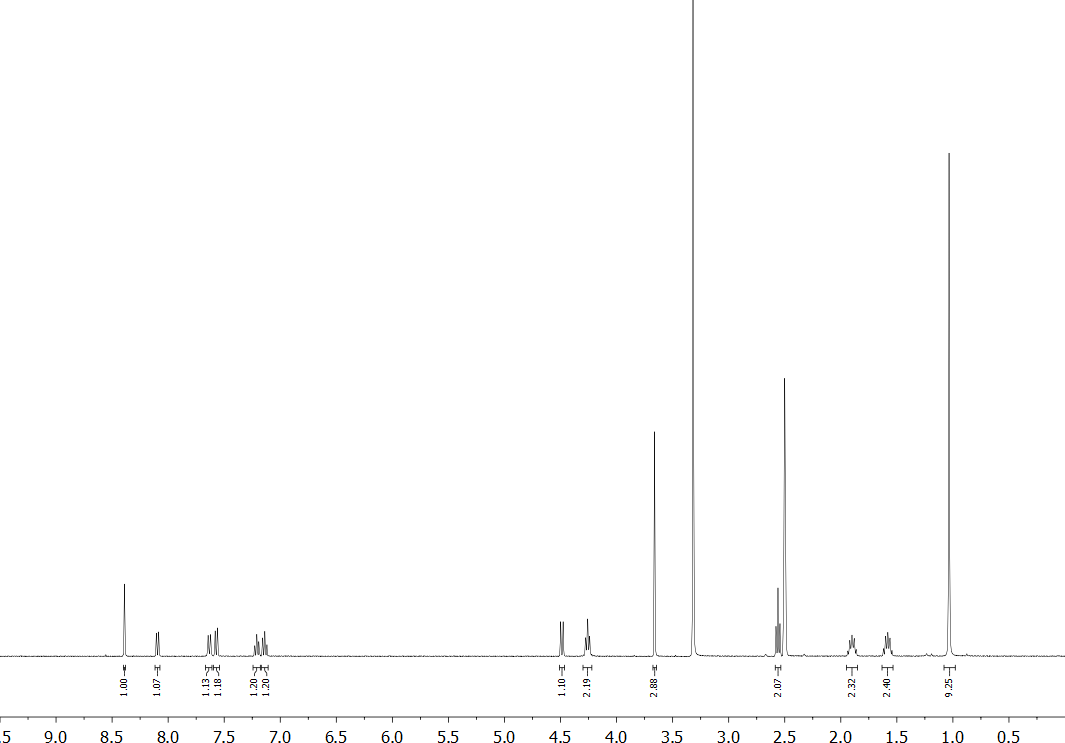


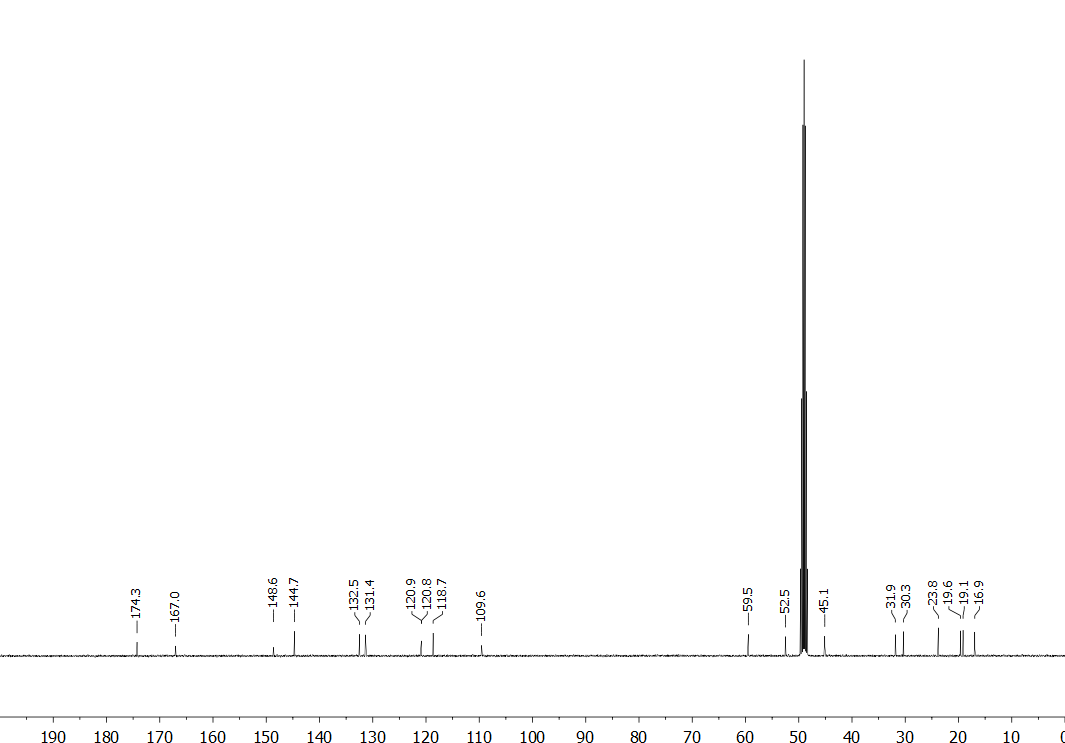


## **Figure S12.** ^1^H (400 MHz, CDCl_3_) and ^13^C (101 MHz, DMSO-*d*_6_) NMR spectra for methyl (1-(4-cyanobutyl)-1*H*-indole-3-carbonyl)-*L*-phenylalaninate (MPP-4CN-BUTICA, **19**).


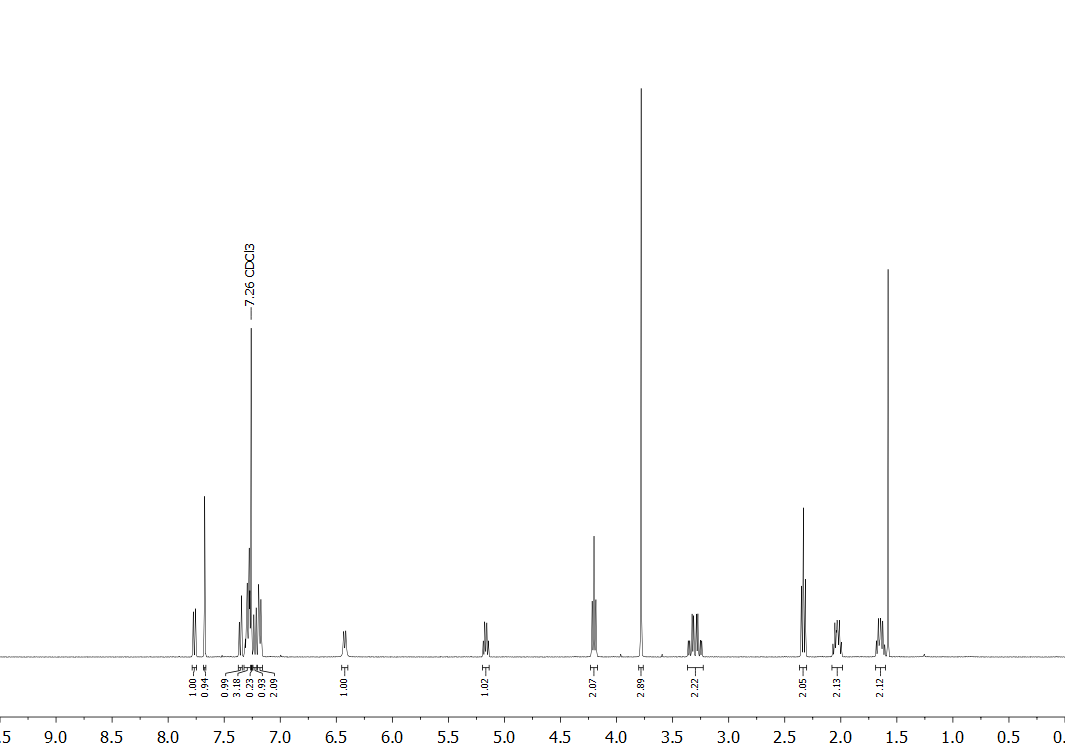


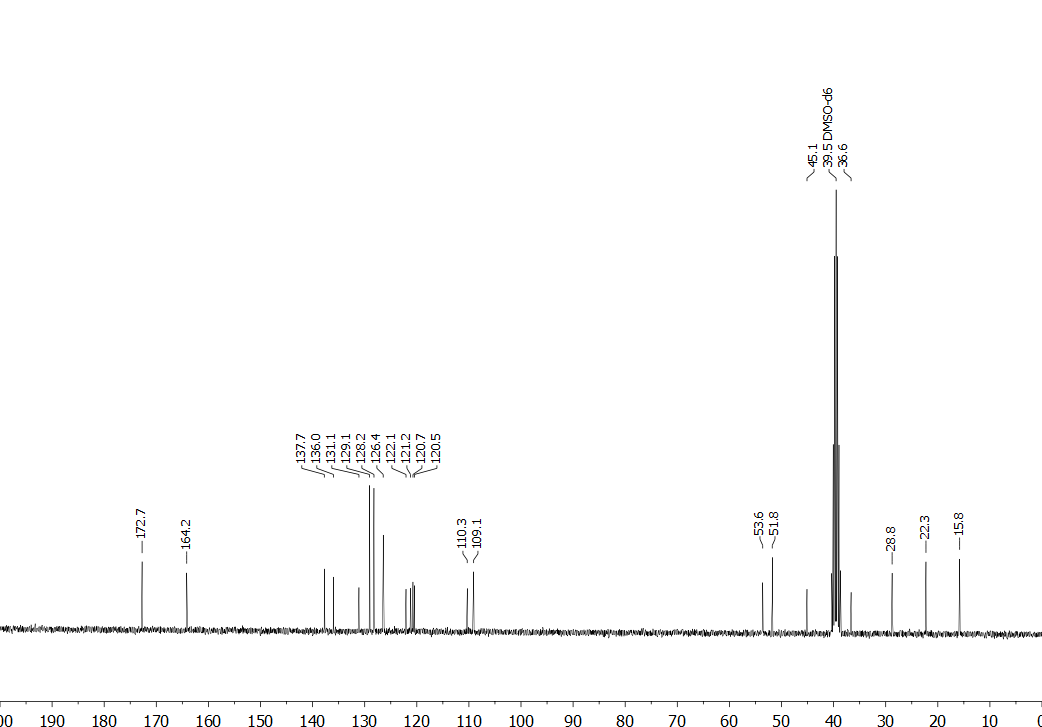


## **Figure S13.** ^1^H (400 MHz, DMSO-*d*_6_) and ^13^C (101 MHz, CD_3_OD) NMR spectra for methyl (1-(4-cyanobutyl)-1*H*-indazole-3-carbonyl)-*L*-valinate (MMB-4CN-BUTINACA, **6**).


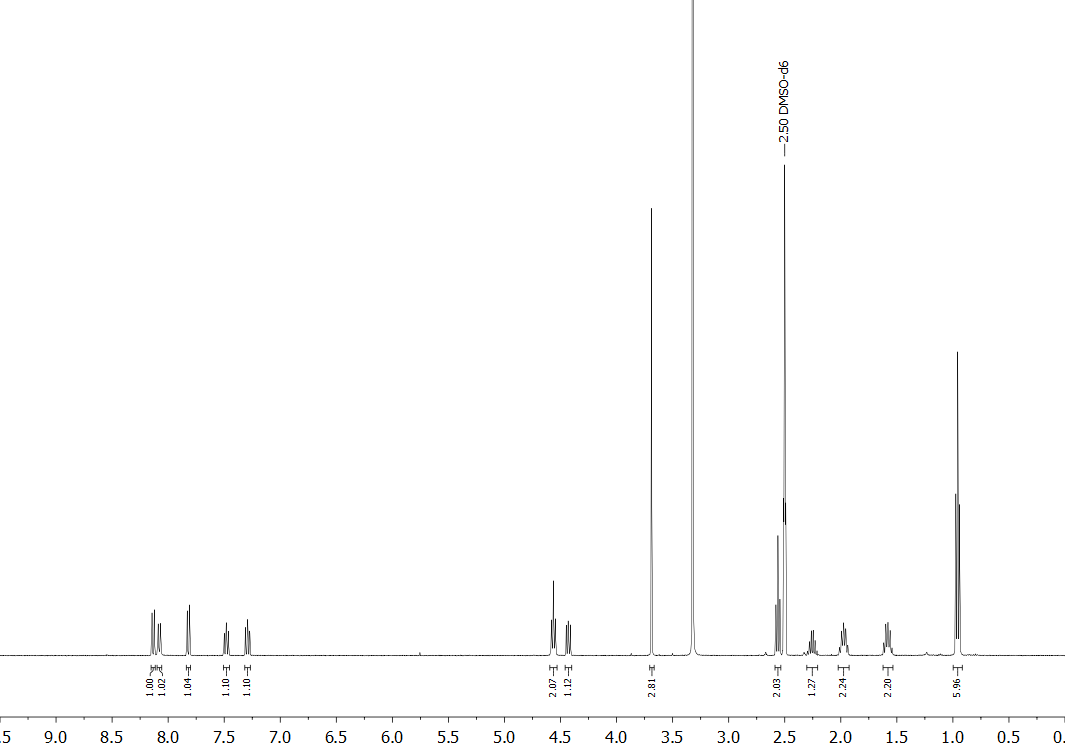


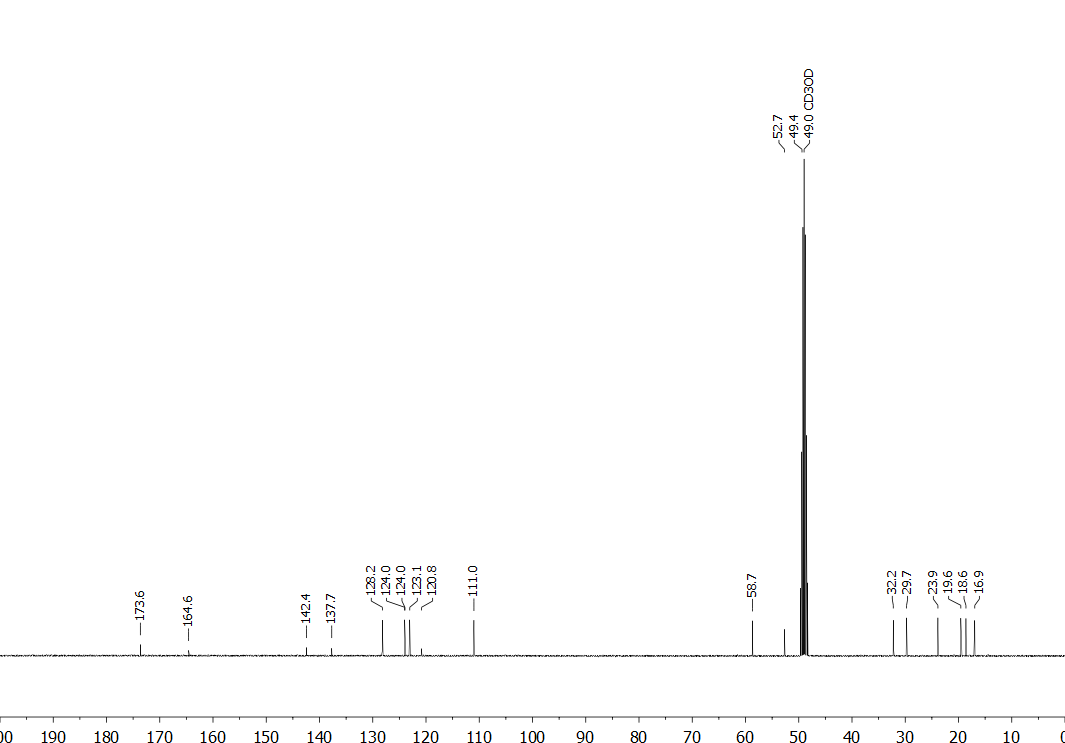


## **Figure S14.** ^1^H (400 MHz, DMSO-*d*_6_) and ^13^C (101 MHz, DMSO-*d*_6_) NMR spectra for methyl (*S*)-2-(1-(4-cyanobutyl)-1*H*-indazole-3-carboxamido)-3,3-dimethylbutanoate (MDMB-4CN-BUTINACA, **20**).


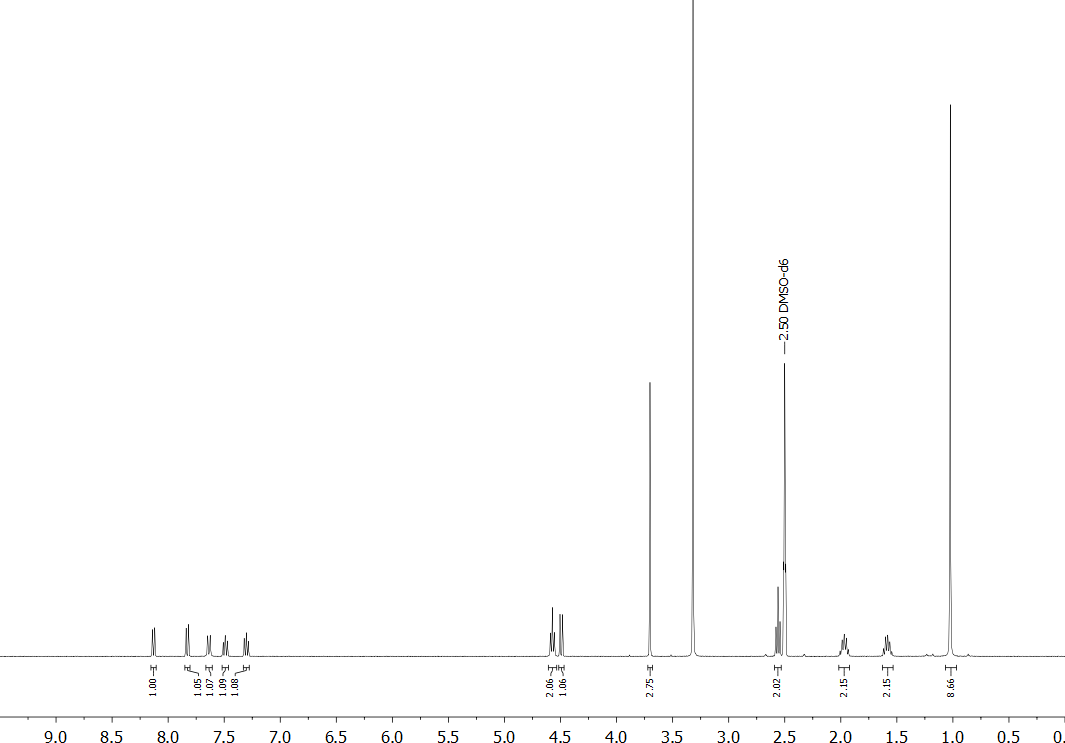


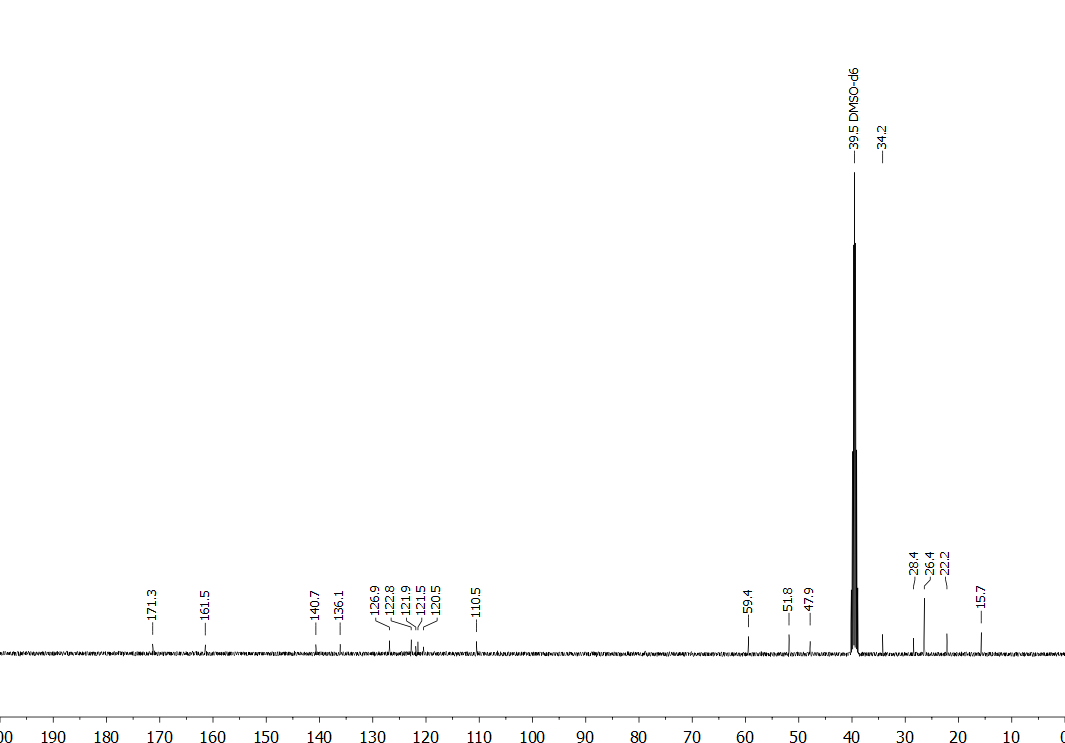


## **Figure S15.** ^1^H (400 MHz, CDCl_3_) and ^13^C (101 MHz, CD_3_OD) NMR spectra for methyl (1-(4-cyanobutyl)-1*H*-indazole-3-carbonyl)-*L*-phenylalaninate (MPP-4CN-BUTINACA, **21**).


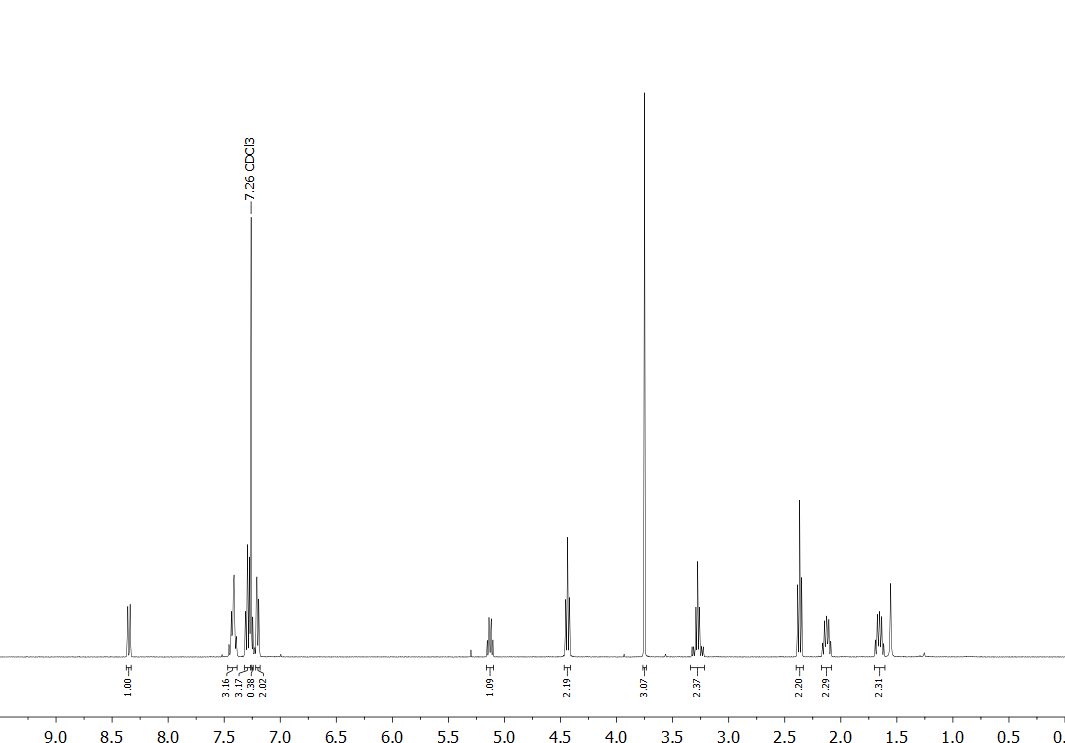


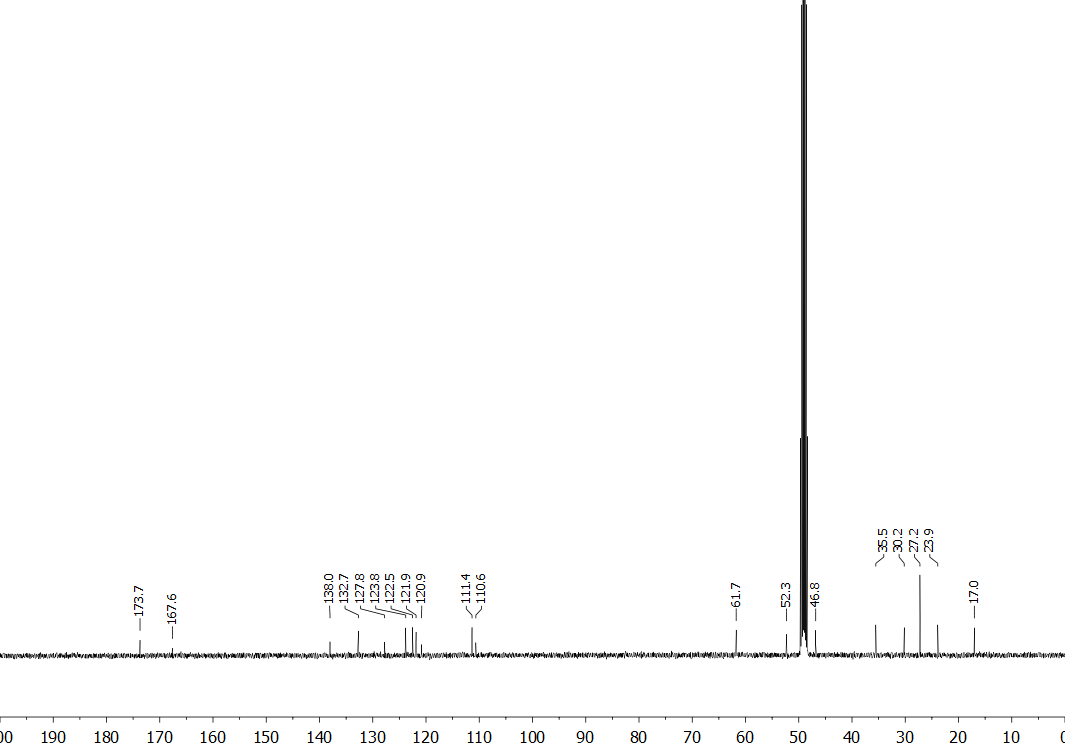


## **Figure S16.** ^1^H (400 MHz, CDCl_3_) and ^13^C (101 MHz, DMSO-*d*_6_) NMR spectra for methyl (1-(4-cyanobutyl)-1*H*-pyrrolo[2,3-*b*]pyridine-3-carbonyl)-*L*-valinate (MMB-4CN-BUT7AICA, **22**).


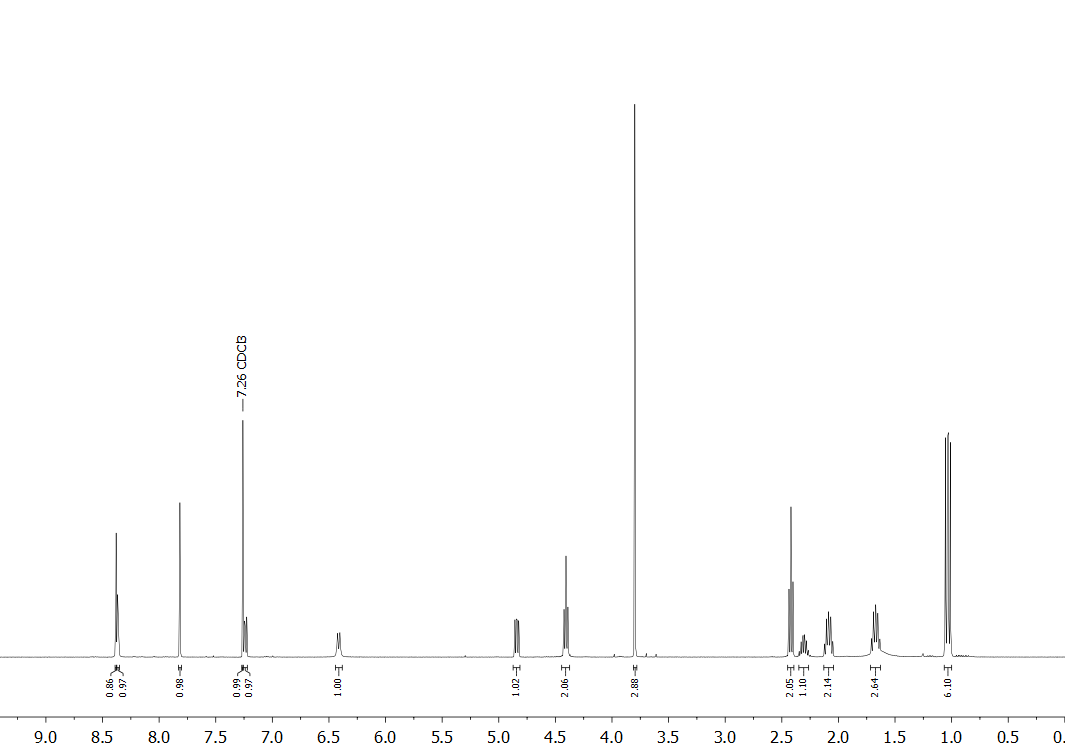


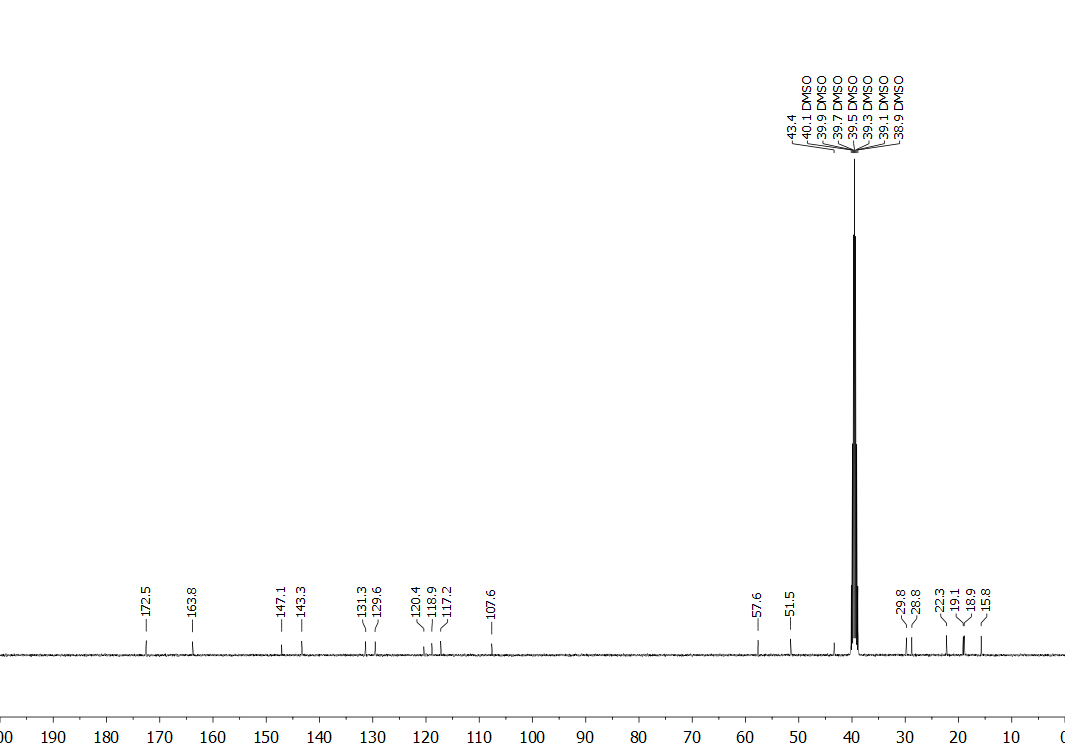


## **Figure S17.** ^1^H (400 MHz, CD_3_OD) and ^13^C (101 MHz, CD_3_OD) NMR spectra for methyl (*S*)-2-(1-(4-cyanobutyl)-1*H*-pyrrolo[2,3-*b*]pyridine-3-carboxamido)-3,3-dimethylbutanoate (MDMB-4CN-BUT7AICA, **23**).


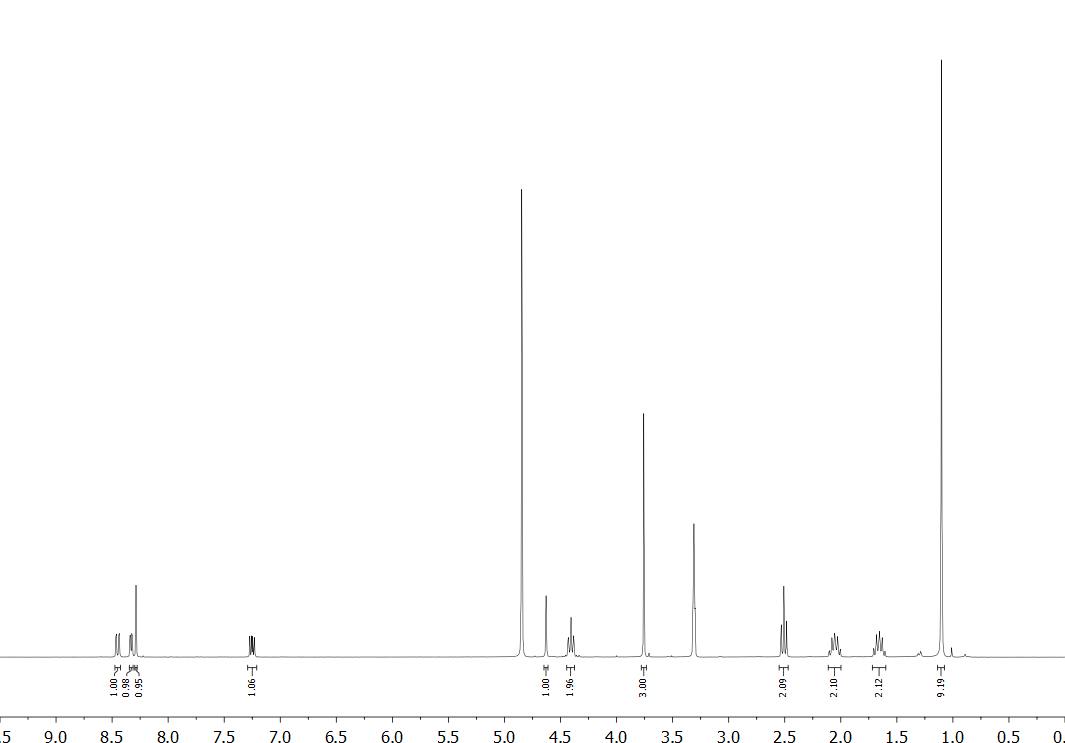


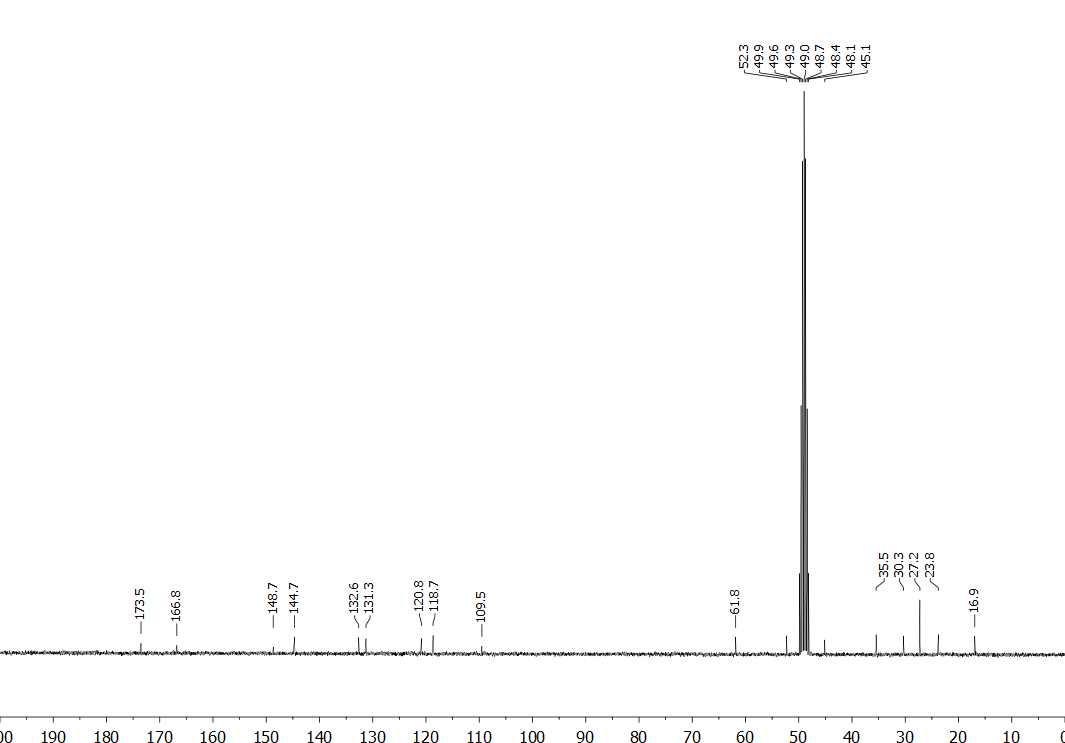


## **Figure S18.** ^1^H (400 MHz, CDCl_3_) and ^13^C (101 MHz, CD_3_OD) NMR spectra for methyl (1-(4-cyanobutyl)-1*H*-pyrrolo[2,3-*b*]pyridine-3-carbonyl)-*L*-phenylalaninate (MPP-4CN-BUT7AICA, **24**).


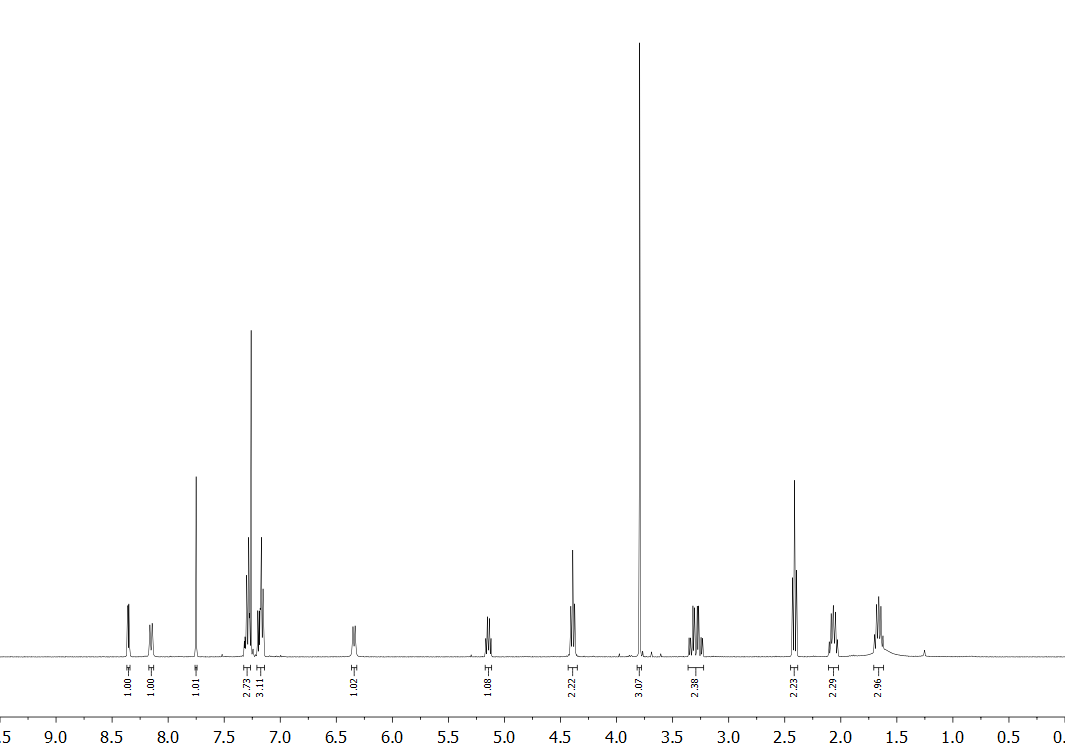


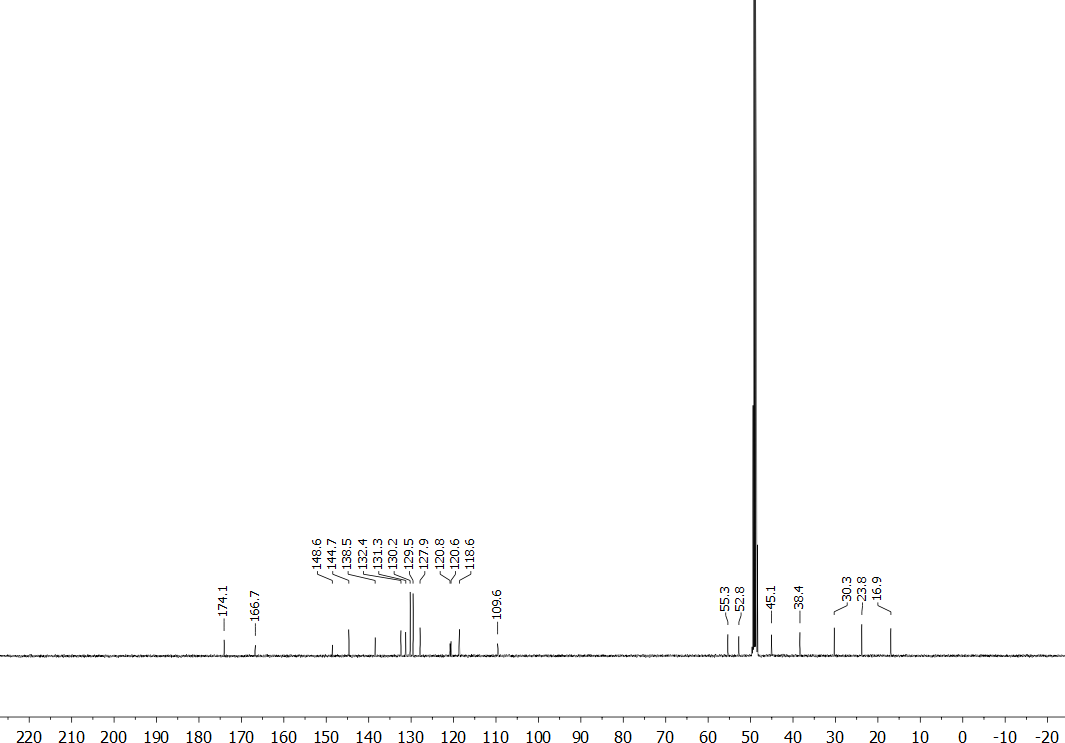


## **Figure S19.** ^1^H (400 MHz, DMSO-*d*_6_) and ^13^C (101 MHz, DMSO-*d*_6_) NMR spectra for (*S*)-*N*-(1-amino-3-methyl-1-oxobutan-2-yl)-1-(4-fluorobutyl)-1*H*-indole-3-carboxamide (AB-4F-BUTICA, **25**).


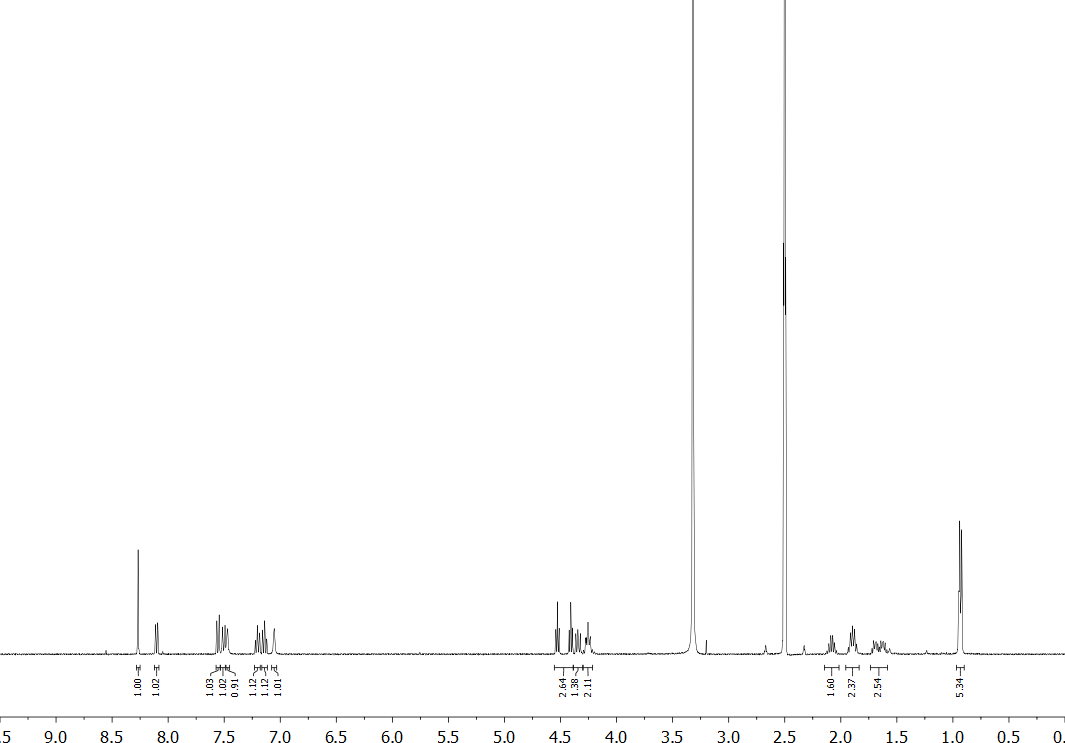


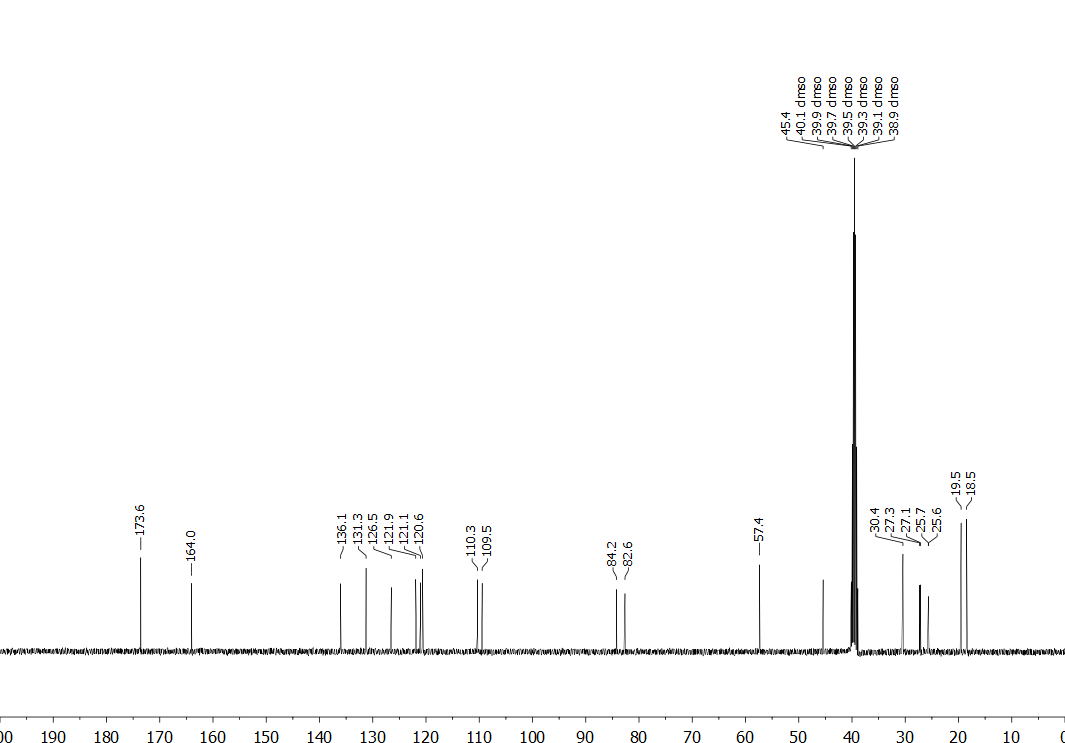


## **Figure S20.** ^1^H (400 MHz, DMSO-*d*_6_) and ^13^C (101 MHz, CDCl_3_) NMR spectra for (*S*)-*N*-(1-amino-3,3-dimethyl-1-oxobutan-2-yl)-1-(4-fluorobutyl)-1*H*-indole-3-carboxamide (ADB-4F-BUTICA, **26**).


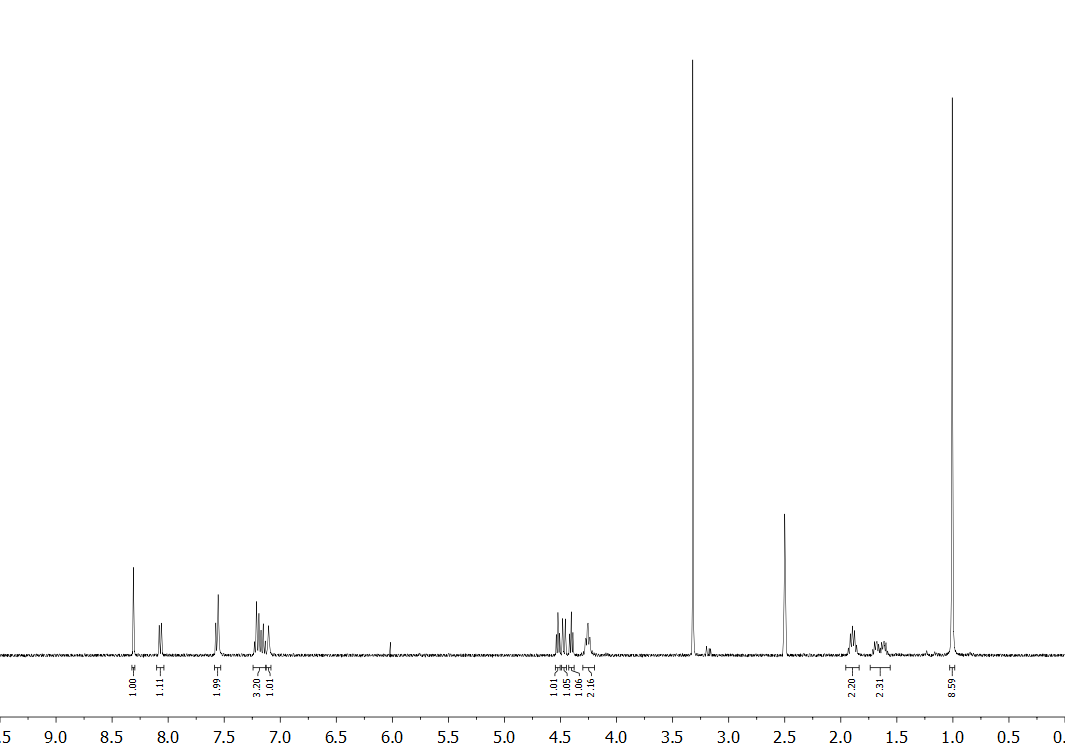


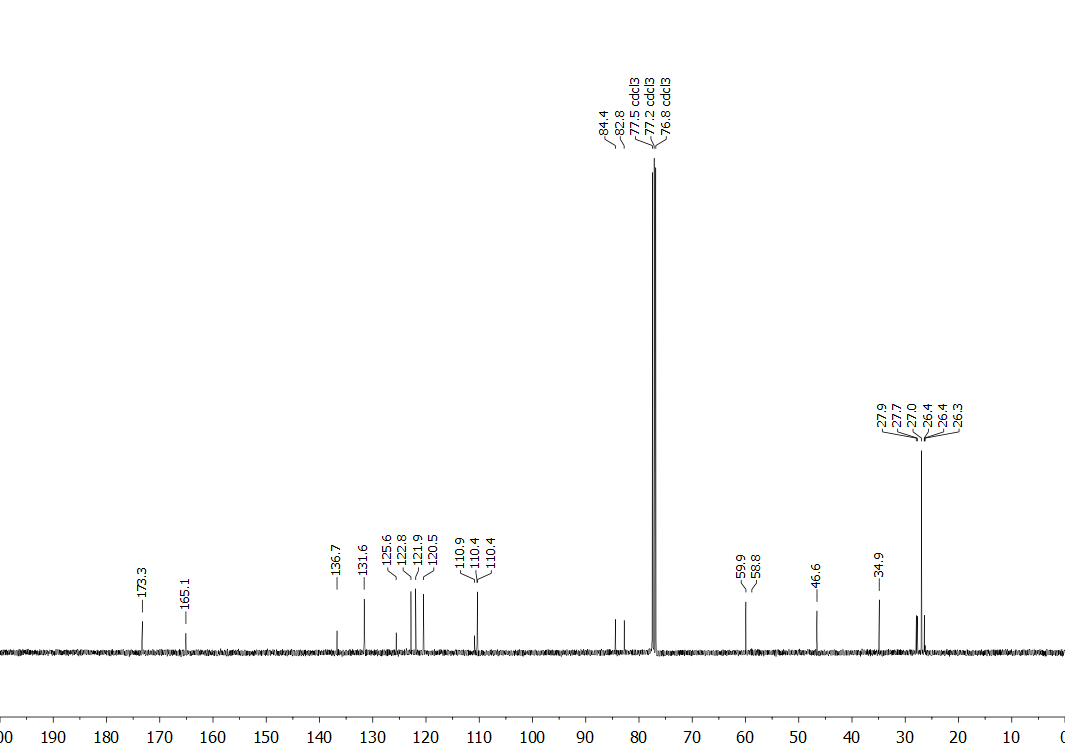


## **Figure S21.** ^1^H (400 MHz, CDCl_3_) and ^13^C (101 MHz, DMSO-*d*_6_) NMR spectra for (*S*)-*N*-(1-amino-1-oxo-3-phenylpropan-2-yl)-1-(4-fluorobutyl)-1*H*-indole-3-carboxamide (APP-4F-BUTICA, **27**).


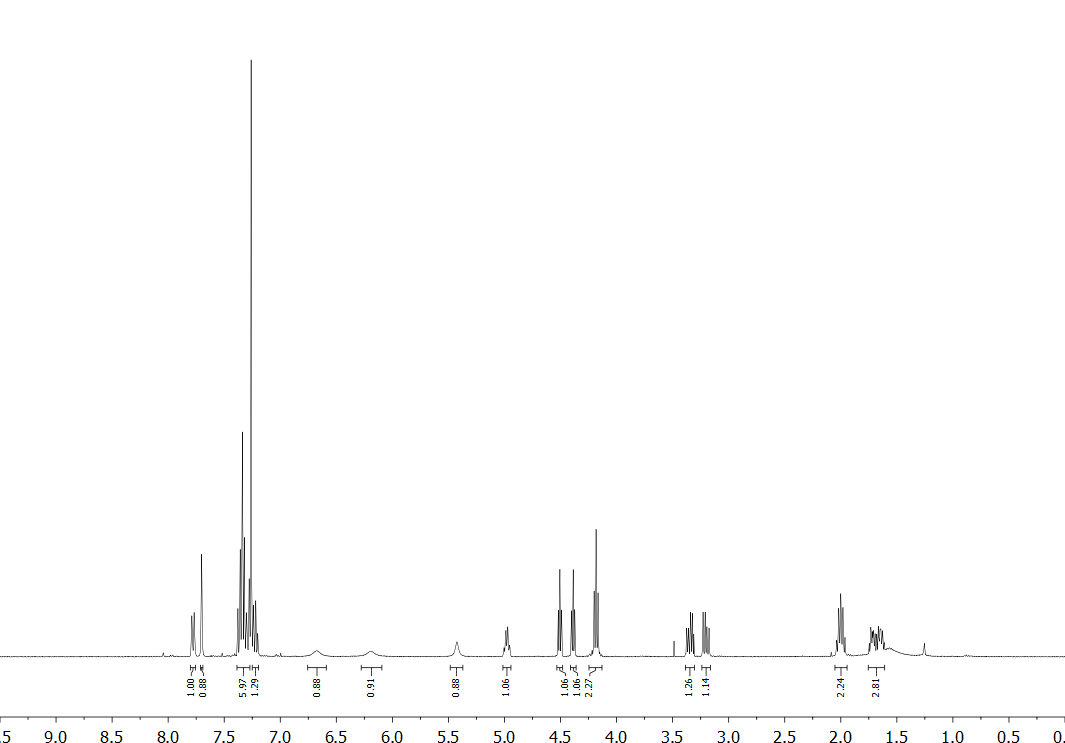


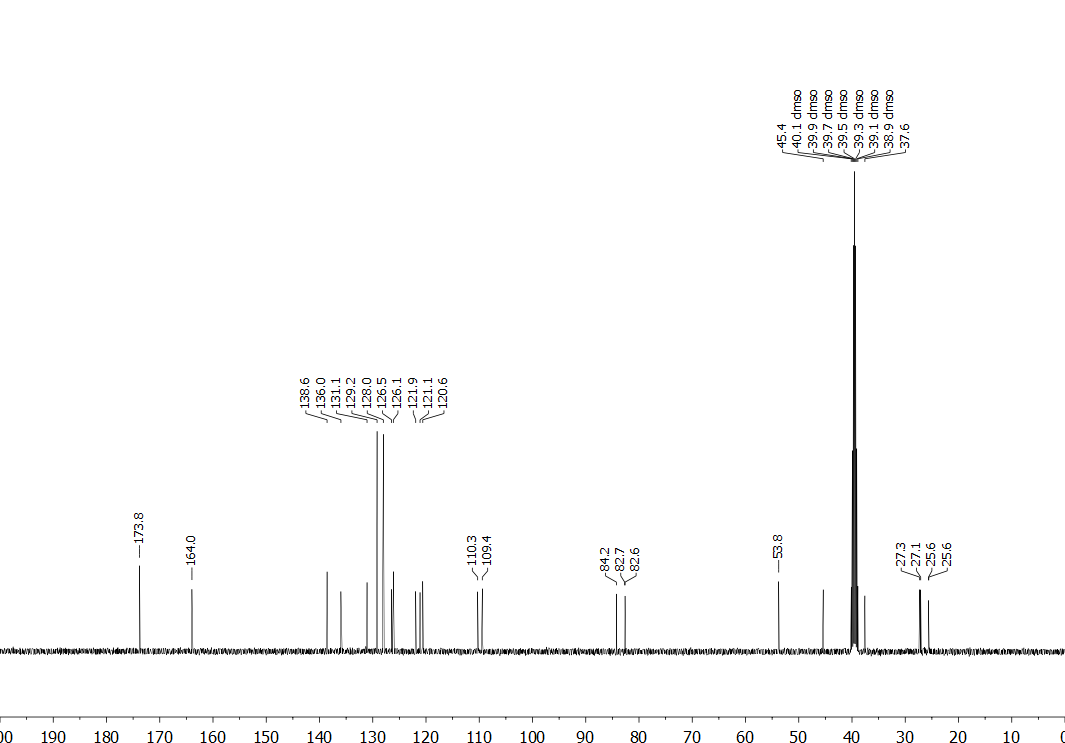


## **Figure S22.** ^1^H (400 MHz, CD_3_OD) and ^13^C (101 MHz, CD_3_OD) NMR spectra for (*S*)-*N*-(1-amino-3-methyl-1-oxobutan-2-yl)-1-(4-fluorobutyl)-1*H*-indazole-3-carboxamide (AB-4F-BUTINACA, **28**).


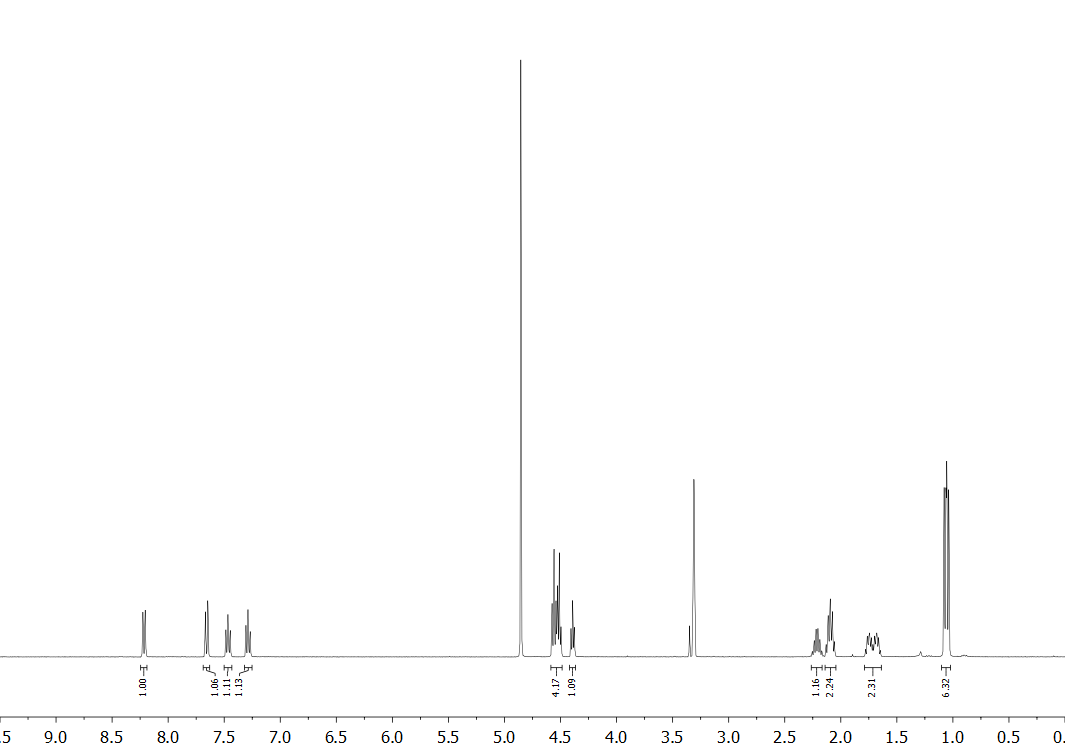


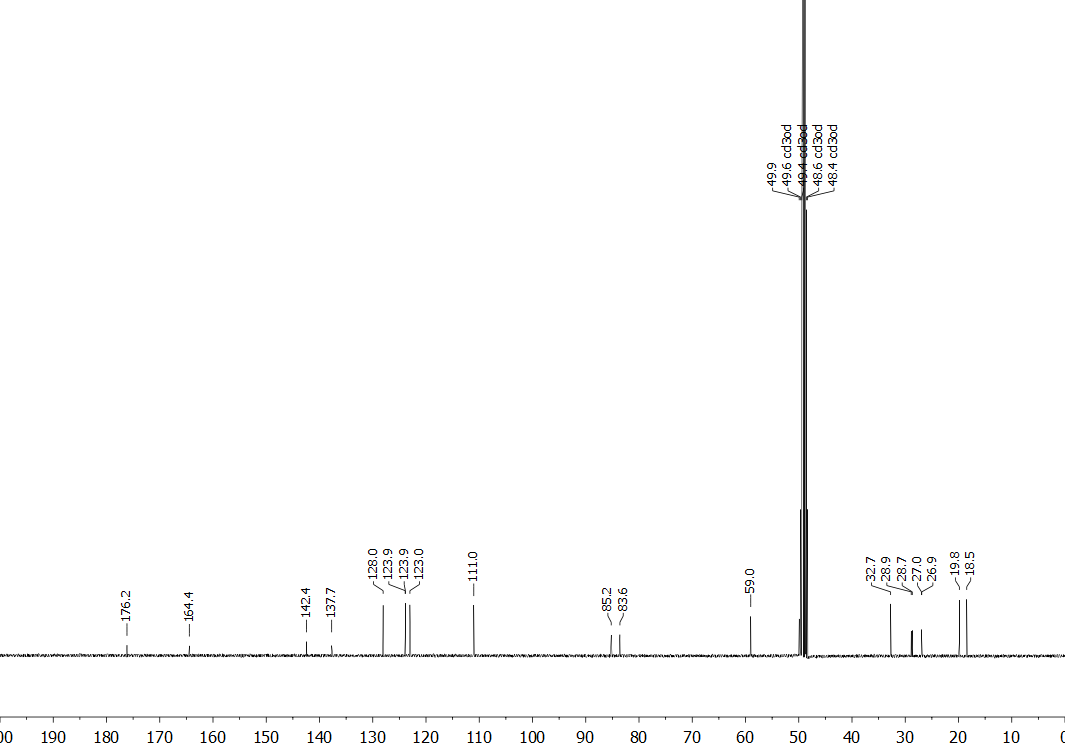


## **Figure S23.** ^1^H (400 MHz, CD_3_OD) and ^13^C (101 MHz, CDCl_3_) NMR spectra for (*S*)-*N*-(1-amino-3,3-dimethyl-1-oxobutan-2-yl)-1-(4-fluorobutyl)-1*H*-indazole-3-carboxamide (ADB-4F-BUTINACA, **29**).


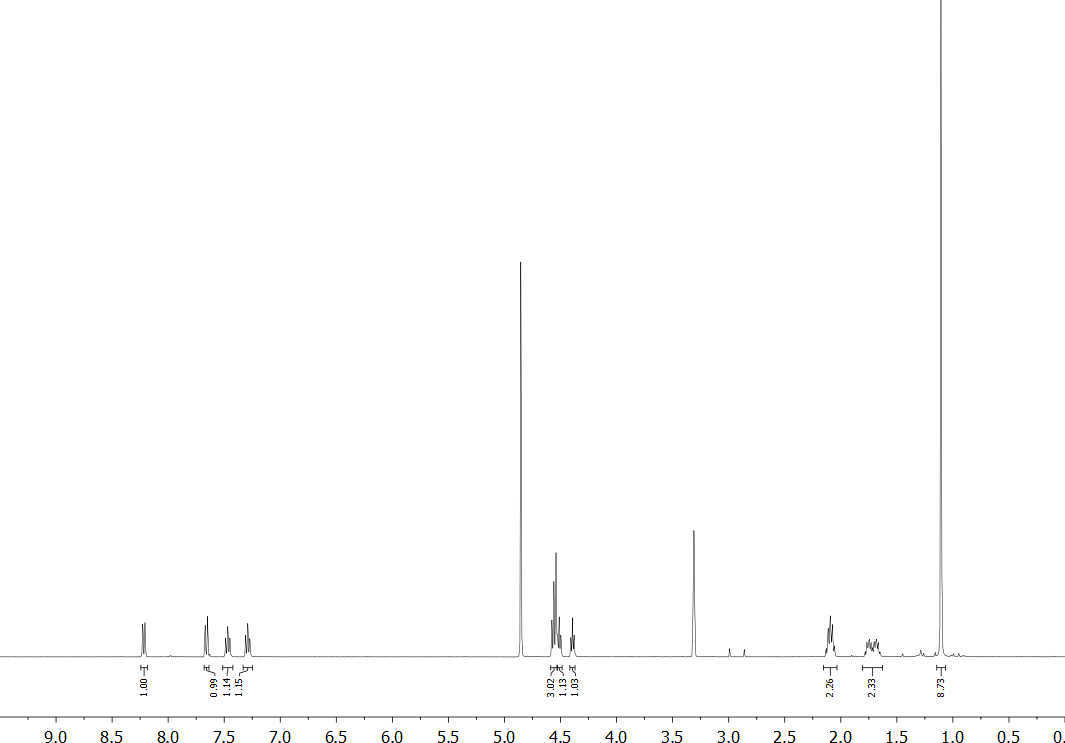


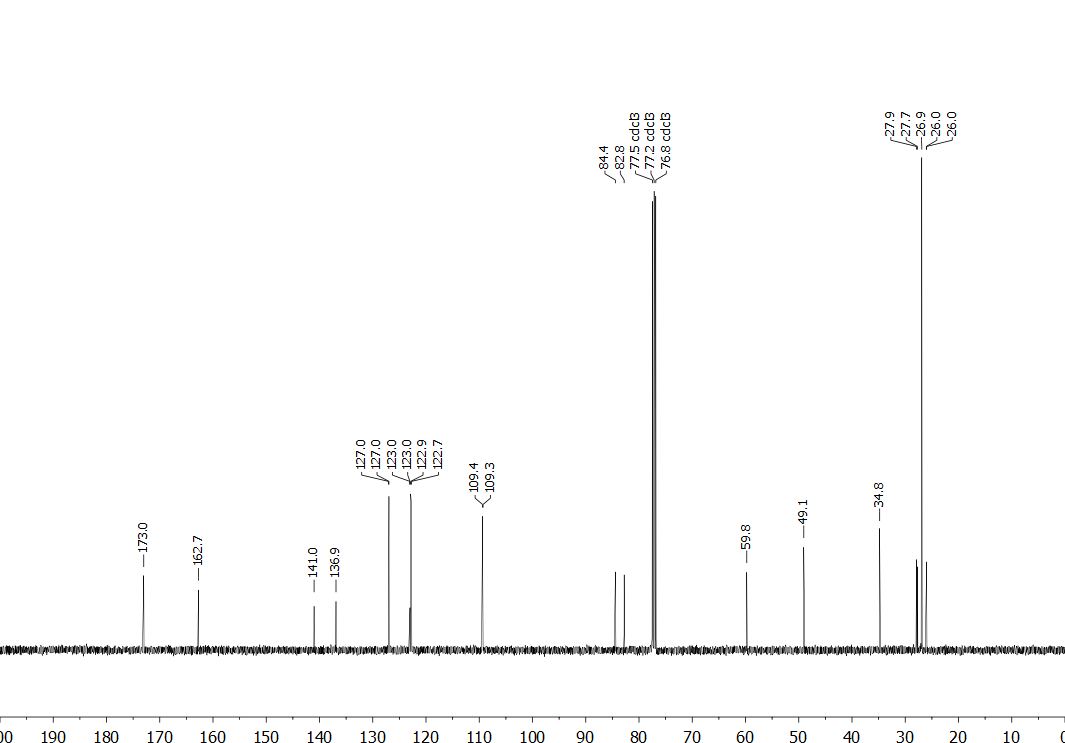


## **Figure S24.** ^1^H (400 MHz, DMSO-*d*_6_) and ^13^C (101 MHz, DMSO-*d*_6_) NMR spectra for (*S*)-*N*-(1-amino-1-oxo-3-phenylpropan-2-yl)-1-(4-fluorobutyl)-1*H*-indazole-3-carboxamide (APP-4F-BUTINACA, **30**).


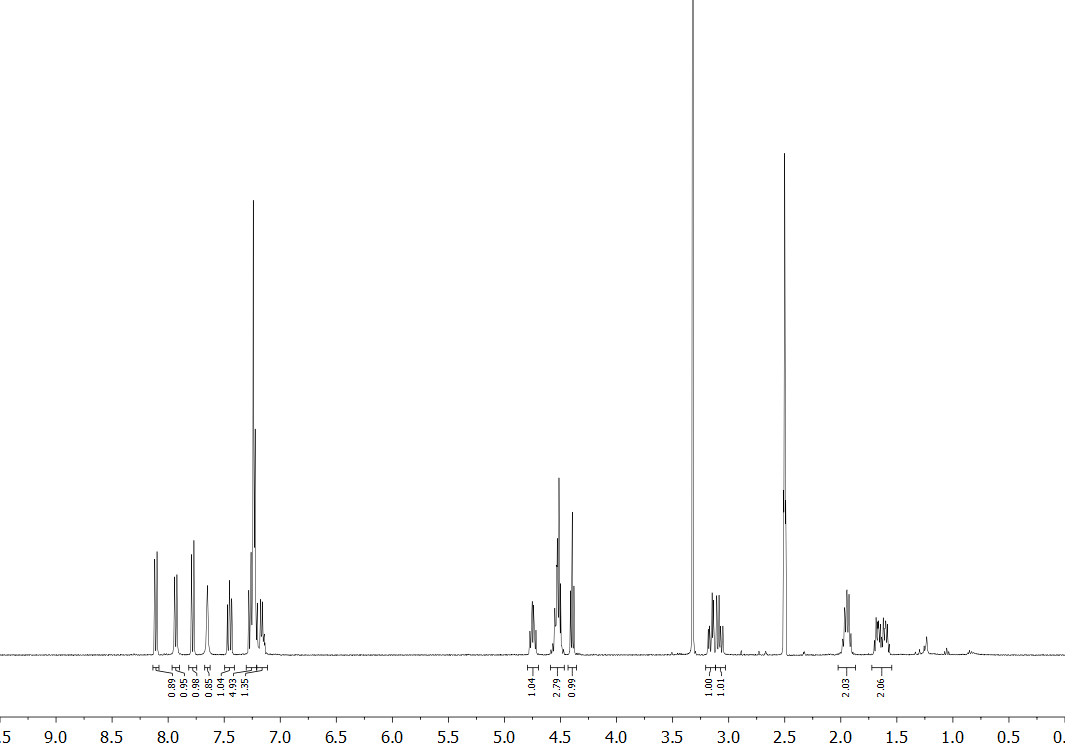


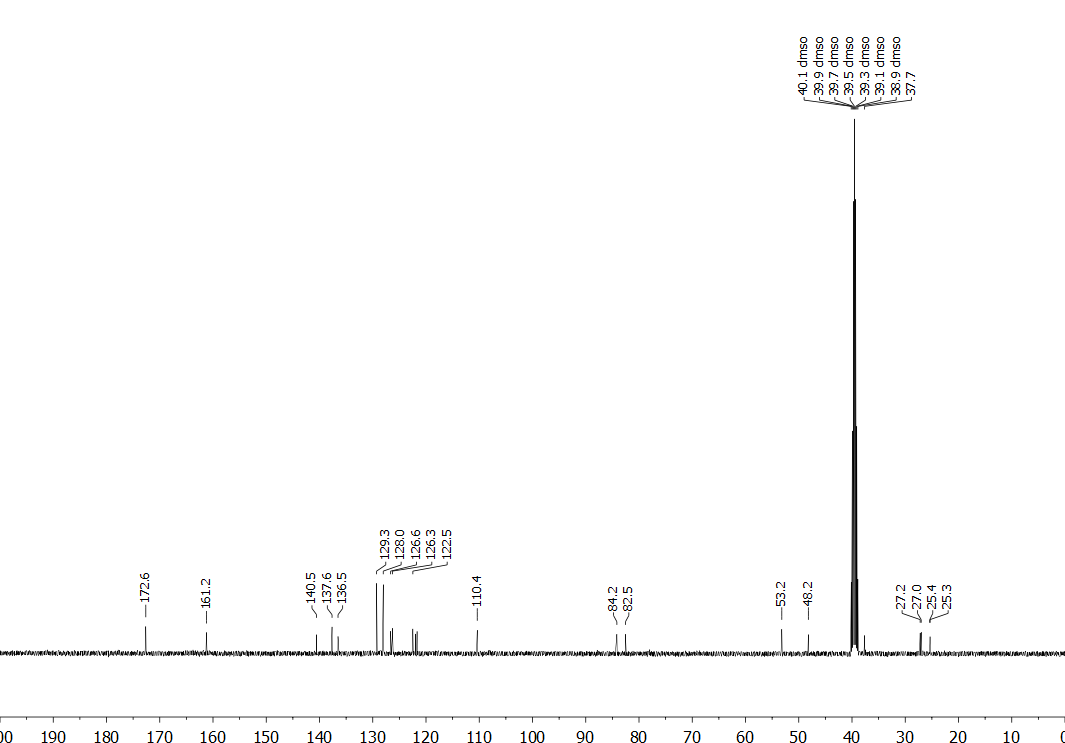


## **Figure S25.** ^1^H (400 MHz, CD_3_OD) and ^13^C (101 MHz, CD_3_OD) NMR spectra for (*S*)-*N*-(1-amino-3-methyl-1-oxobutan-2-yl)-1-(4-fluorobutyl)-1*H*-pyrrolo[2,3-*b*]pyridine-3-carboxamide (AB-4F-BUT7AICA, **31**).


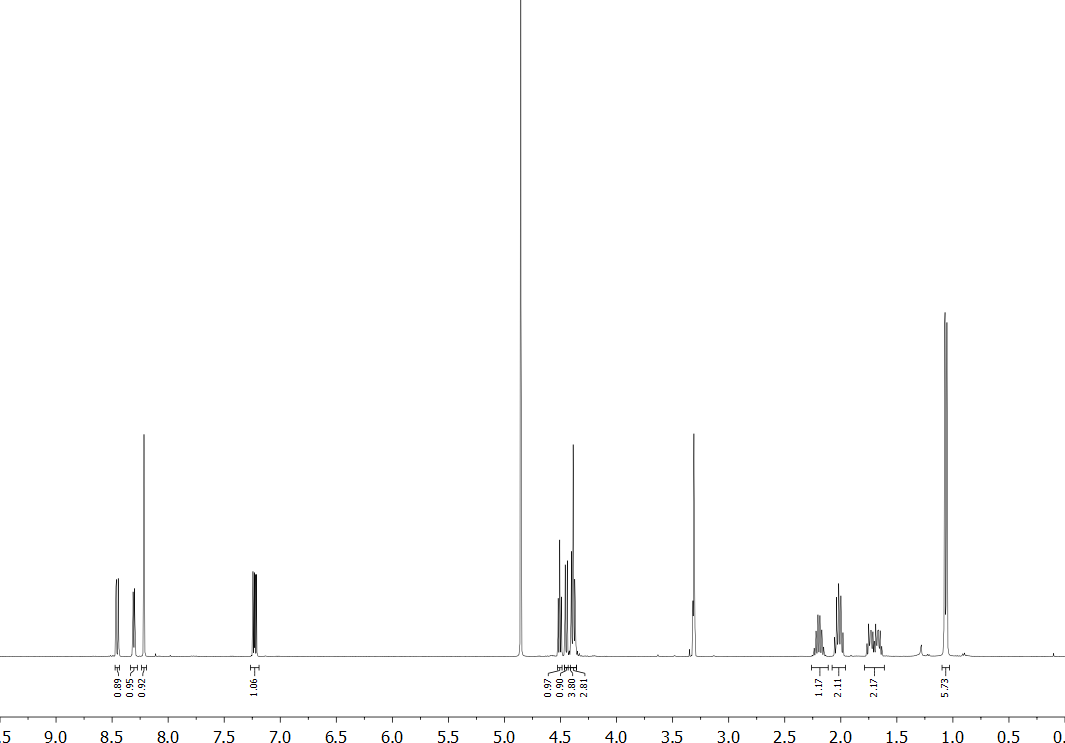


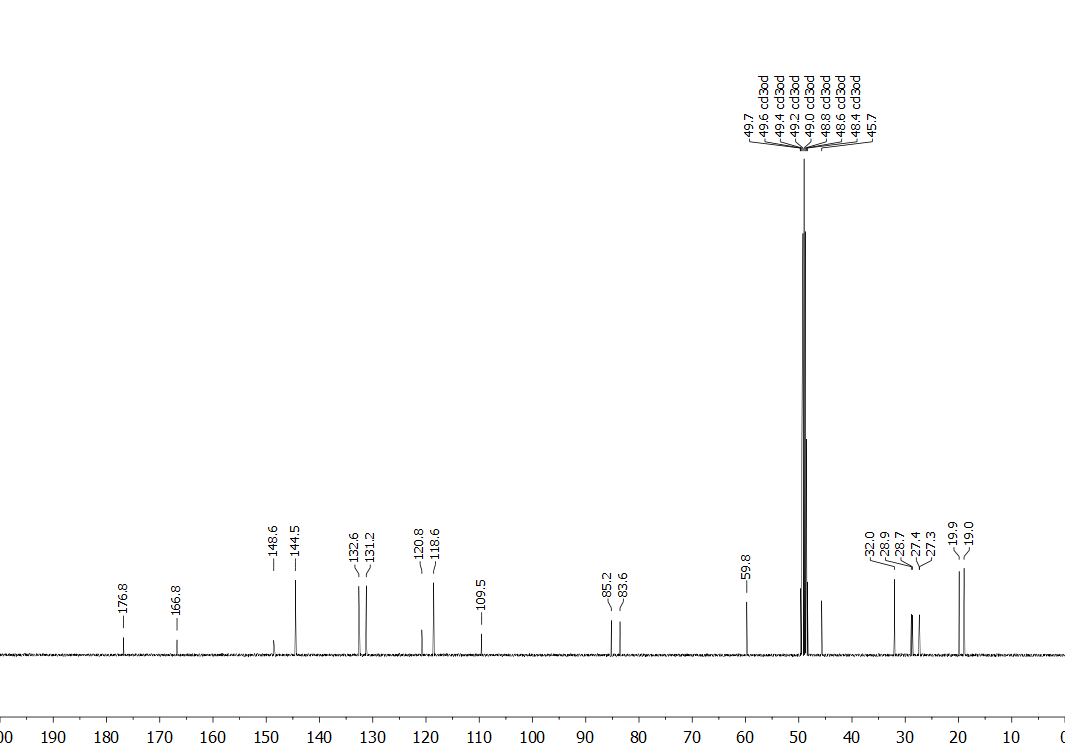


## **Figure S26.** ^1^H (400 MHz, CDCl_3_) and ^13^C (101 MHz, CD_3_OD) NMR spectra for (*S*)-*N*-(1-amino-3,3-dimethyl-1-oxobutan-2-yl)-1-(4-fluorobutyl)-1*H*-pyrrolo[2,3-*b*]pyridine-3-carboxamide (ADB-4F-BUT7AICA, **32**).


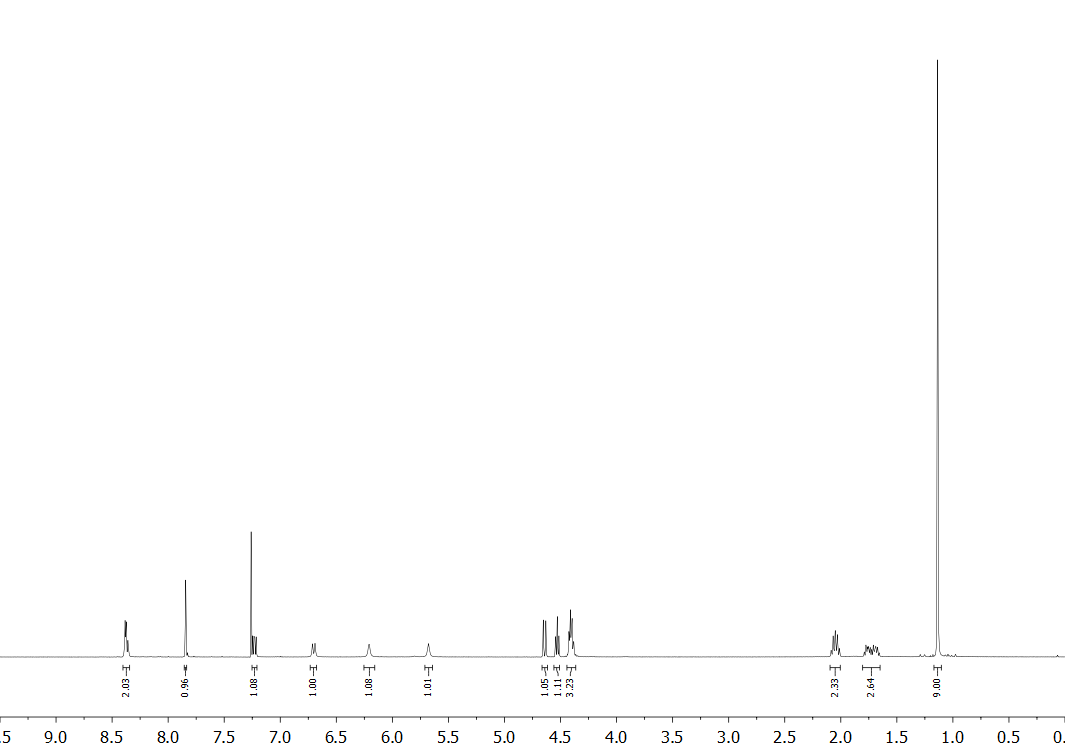


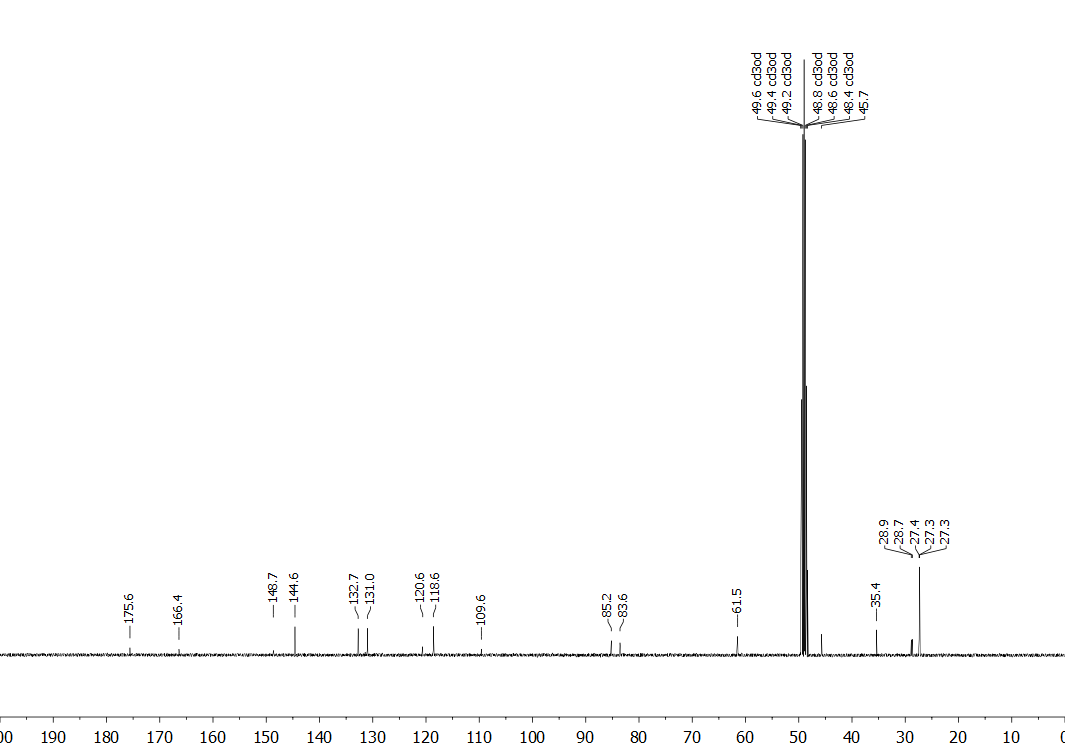


## **Figure S27.** ^1^H (400 MHz, DMSO-*d*_6_) and ^13^C (101 MHz, DMSO-*d*_6_) NMR spectra for (*S*)-*N*-(1-amino-1-oxo-3-phenylpropan-2-yl)-1-(4-fluorobutyl)-1*H*-pyrrolo[2,3-*b*]pyridine-3-carboxamide (APP-4F-BUT7AICA, **33**).


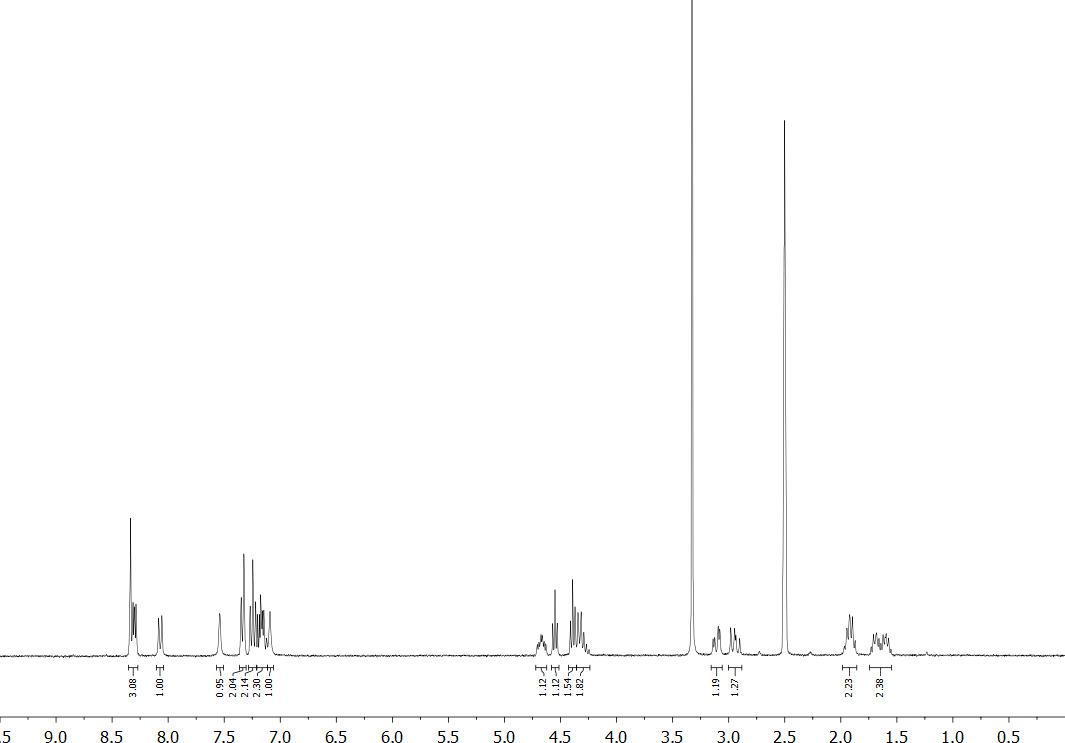


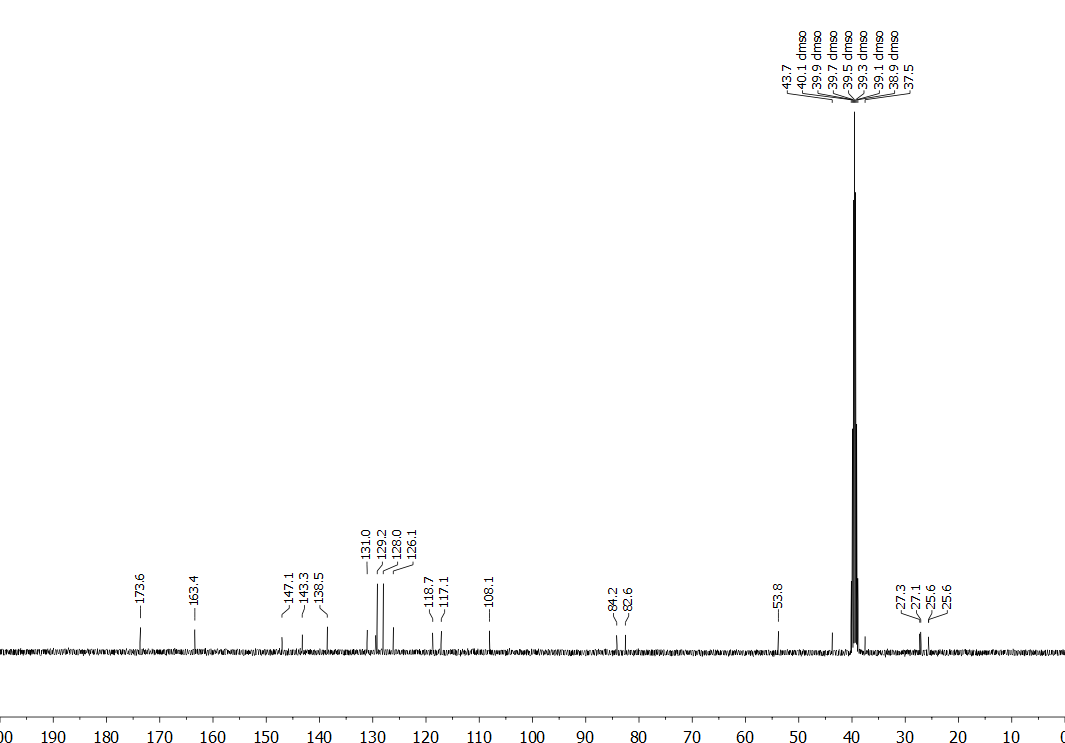


## **Figure S28.** ^1^H (400 MHz, CDCl_3_) and ^13^C (101 MHz, CD_3_OD) NMR spectra for methyl (1-(4-fluorobutyl)-1*H*-indole-3-carbonyl)-*L*-valinate (MMB-4F-BUTICA, **34**).


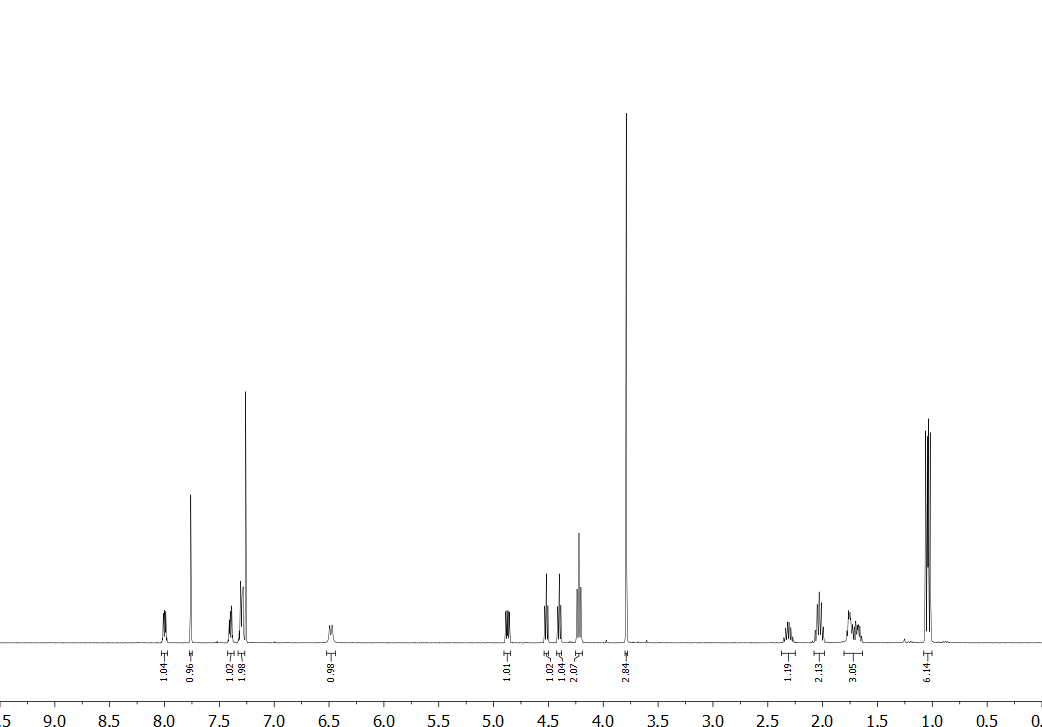


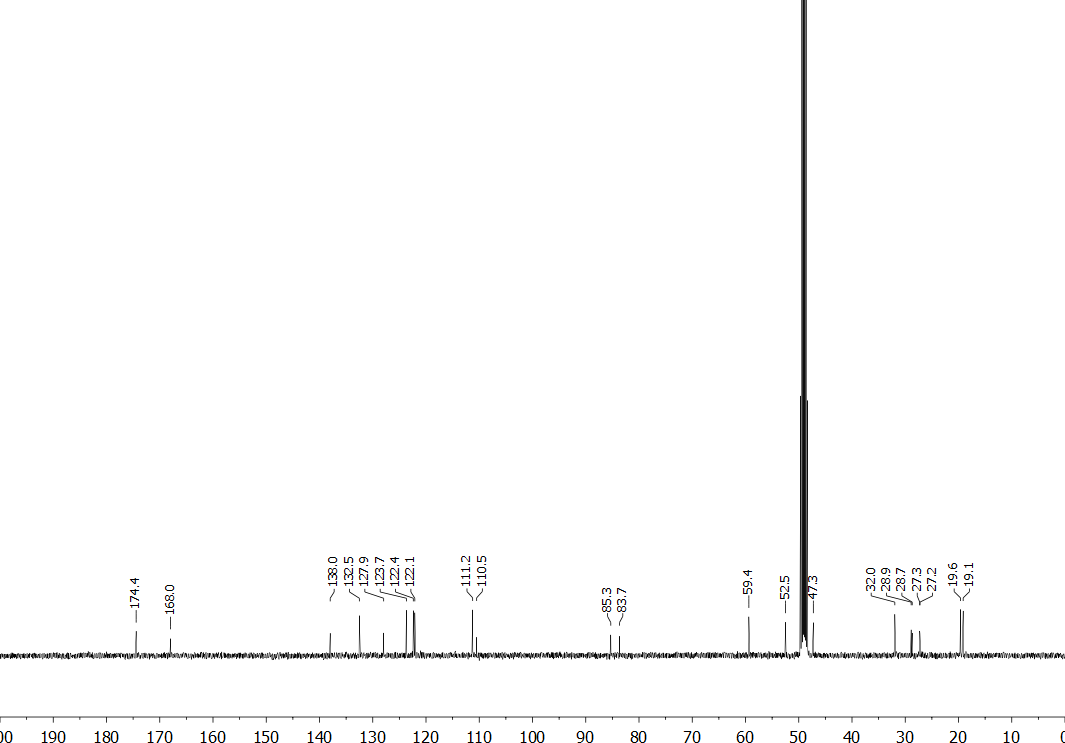


## **Figure S29.** ^1^H (400 MHz, DMSO-*d*_6_) and ^13^C (101 MHz, DMSO-*d*_6_) NMR spectra for methyl (*S*)-2-(1-(4-fluorobutyl)-1*H*-indole-3-carboxamido)-3,3-dimethylbutanoate (MDMB-4F-BUTICA, **7**).


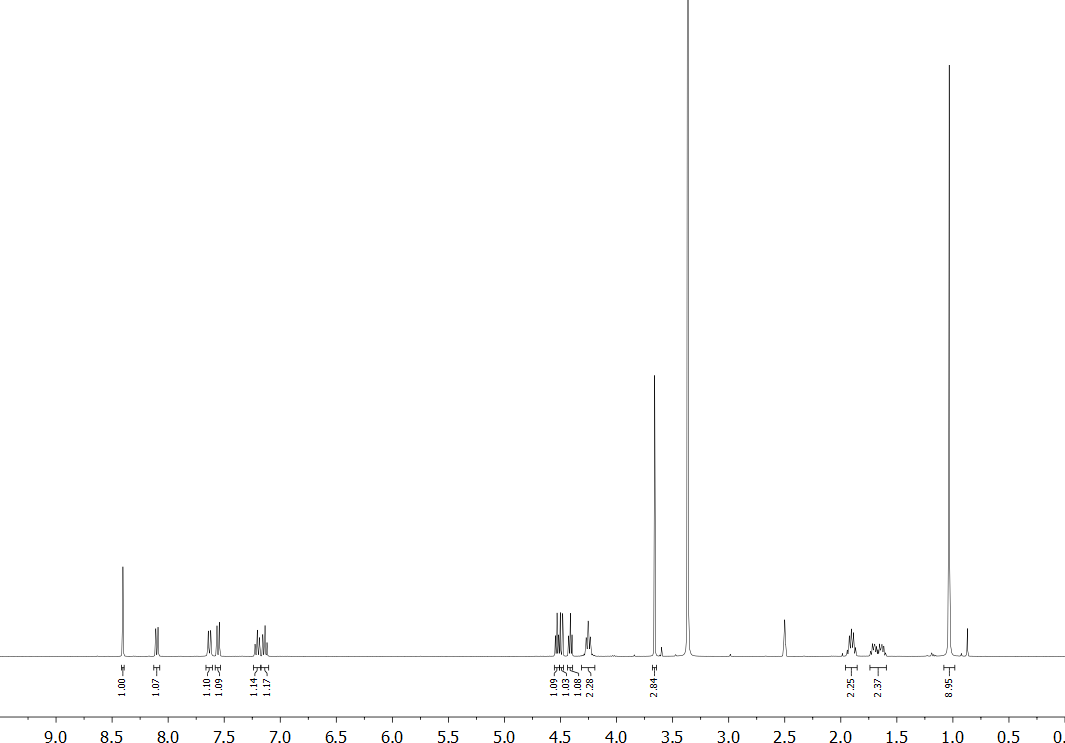


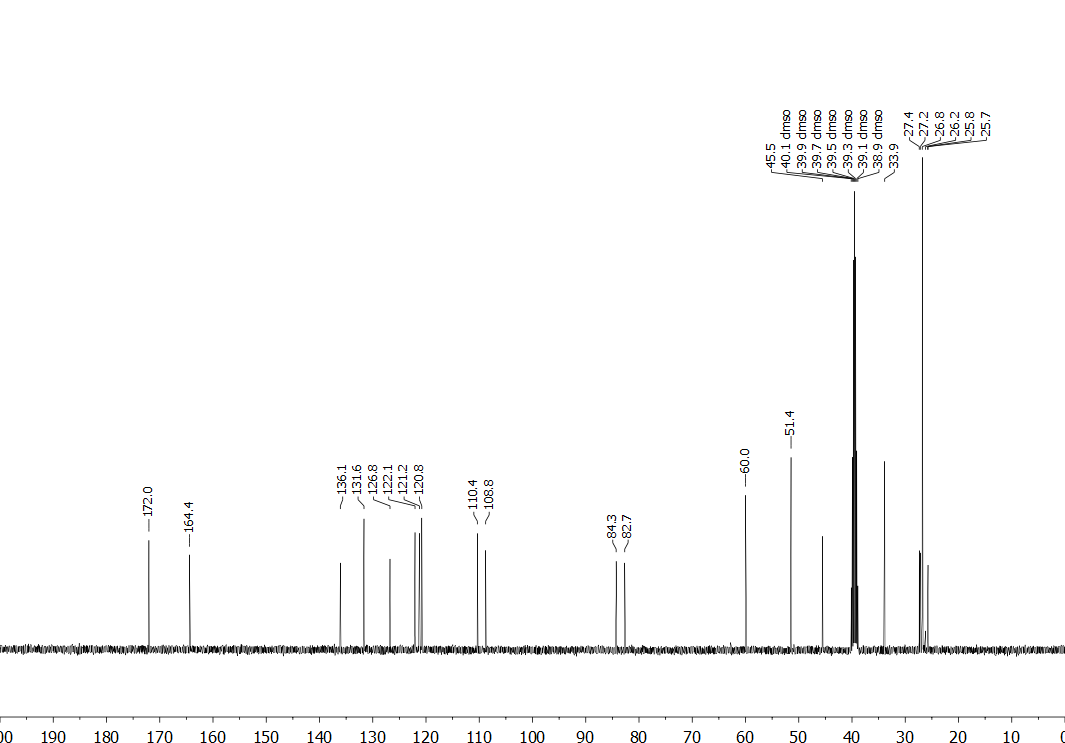


## **Figure S30.** ^1^H (400 MHz, CDCl_3_) and ^13^C (101 MHz, CD_3_OD) NMR spectra for methyl (1-(4-fluorobutyl)-1*H*-indole-3-carbonyl)-*L*-phenylalaninate (MPP-4F-BUTICA, **35**).


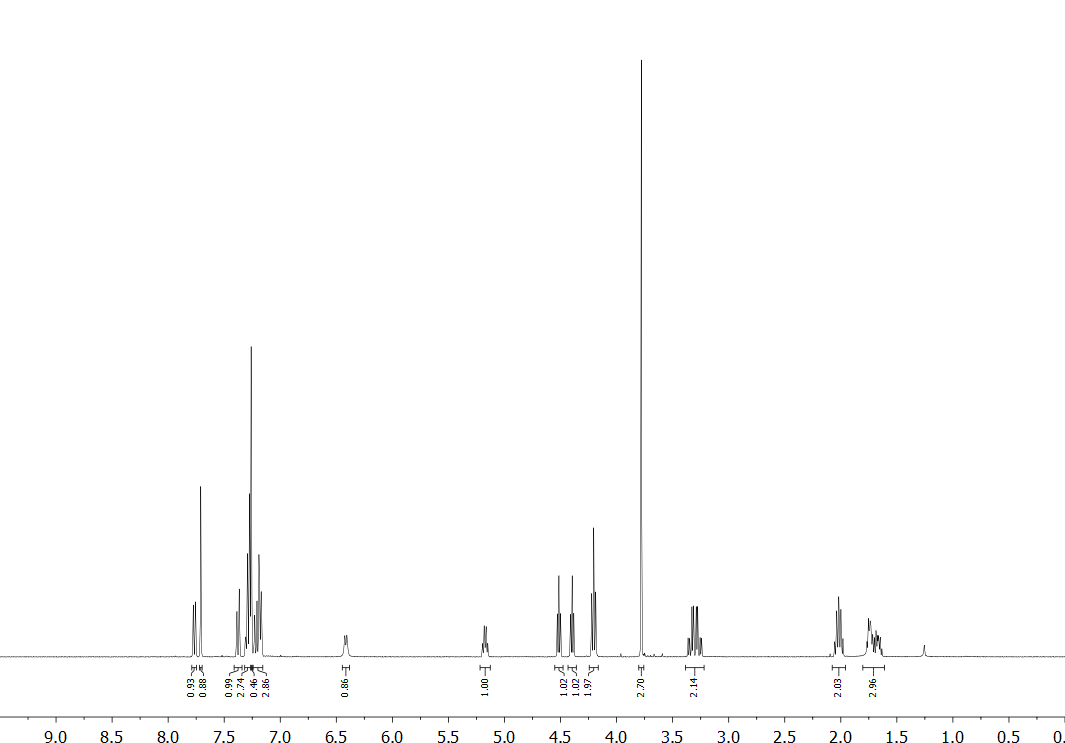


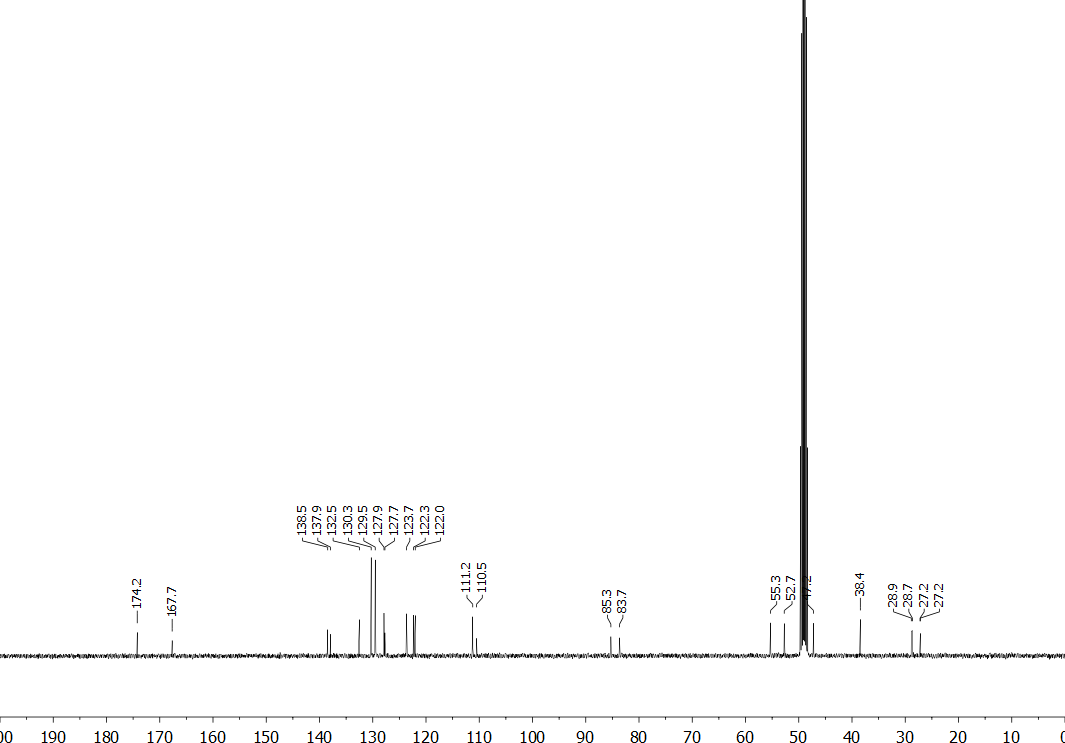


## **Figure S31.** ^1^H (400 MHz, DMSO-*d*_6_) and ^13^C (101 MHz, CD_3_OD) NMR spectra for methyl (1-(4-fluorobutyl)-1*H*-indazole-3-carbonyl)-*L*-valinate (MMB-4F-BUTINACA, **36**).


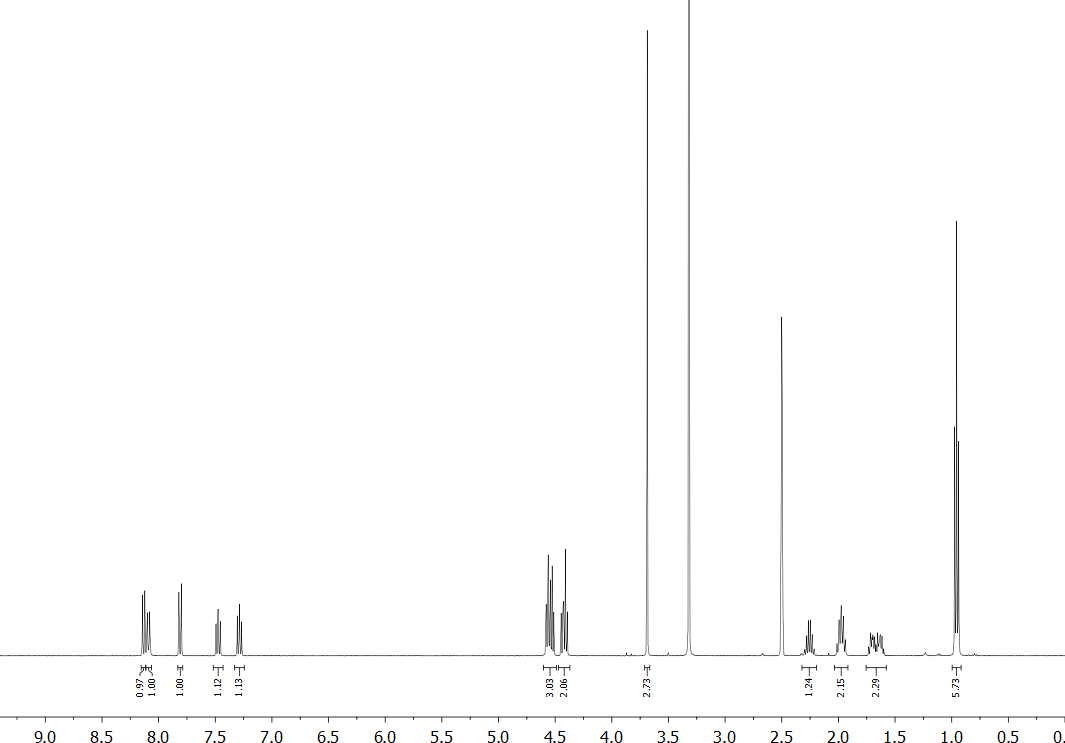


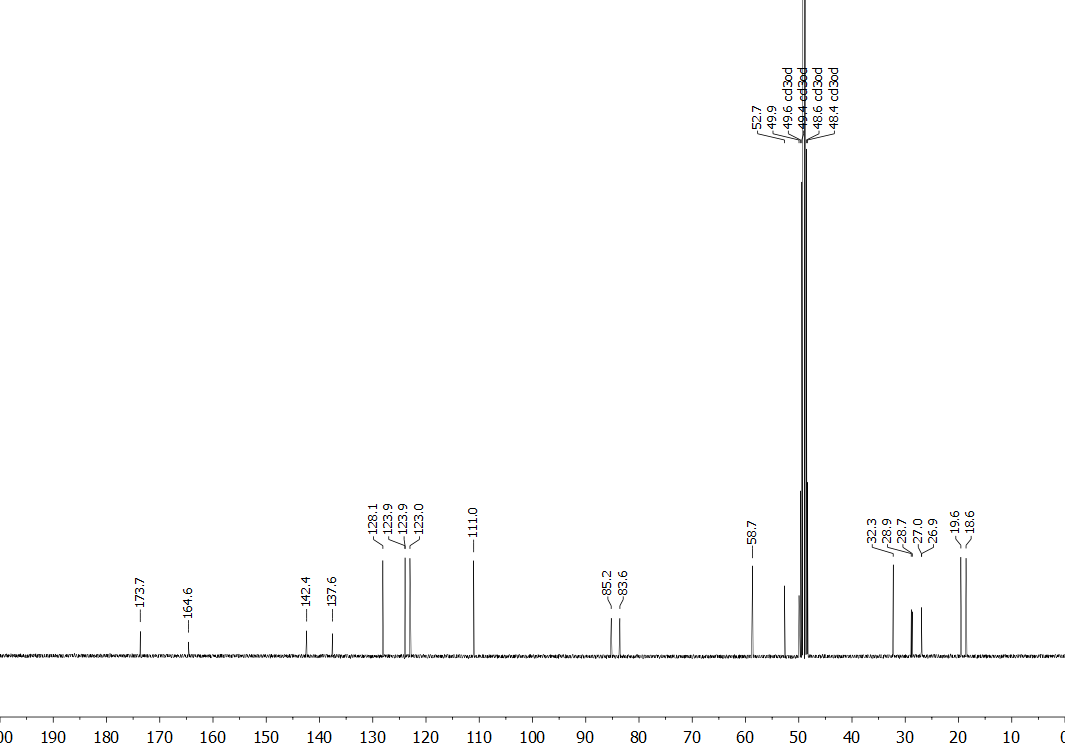


## **Figure S32.** ^1^H (400 MHz, DMSO-*d*_6_) and ^13^C (101 MHz, DMSO-*d*_6_) NMR spectra for methyl (*S*)-2-(1-(4-fluorobutyl)-1*H*-indazole-3-carboxamido)-3,3-dimethylbutanoate (MDMB-4F-BUTINACA, **8**).


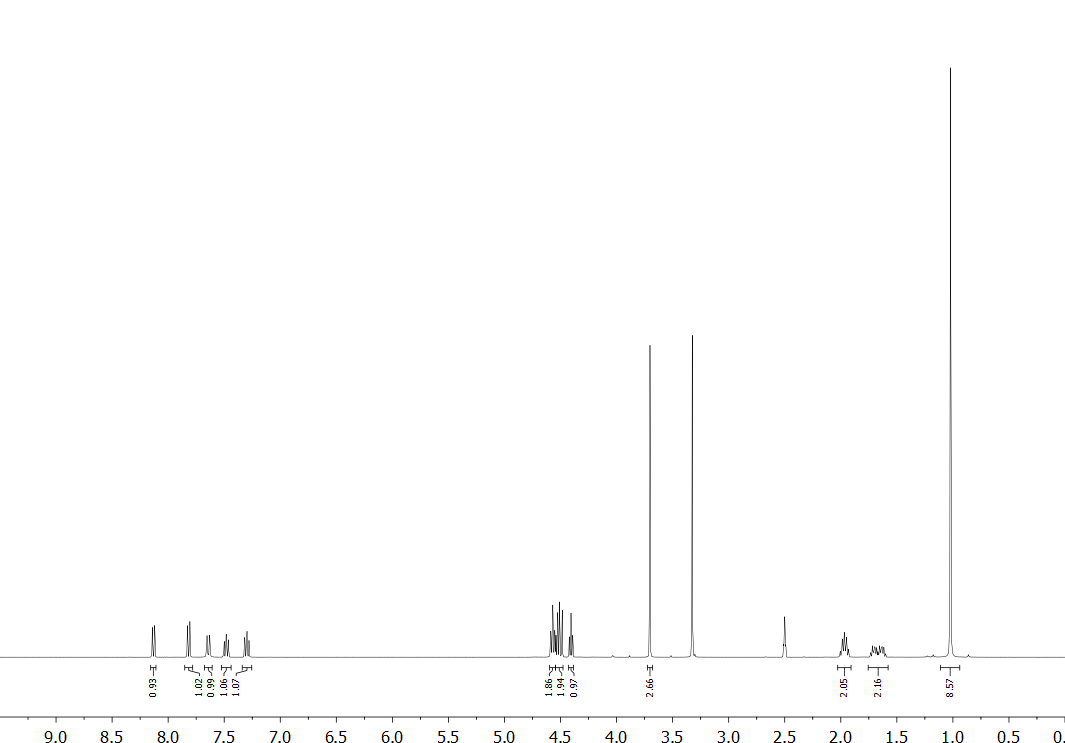


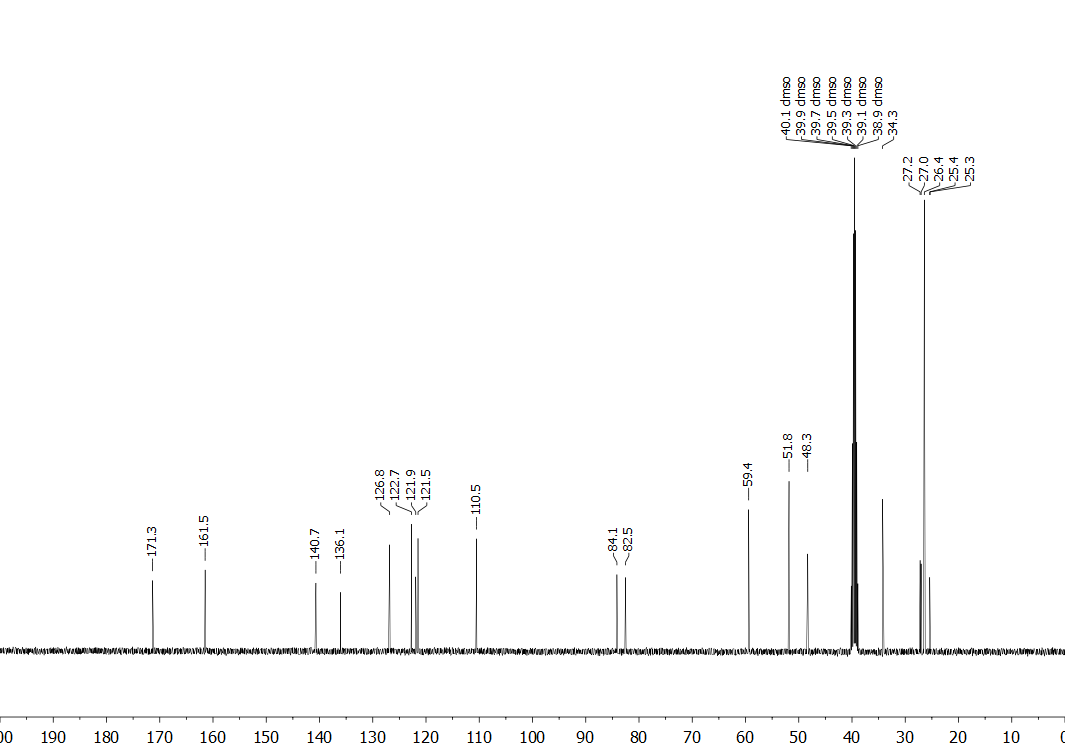


## **Figure S33.** ^1^H (400 MHz, CDCl_3_) and ^13^C (101 MHz, CD_3_OD) NMR spectra for methyl (1-(4-fluorobutyl)-1*H*-indazole-3-carbonyl)-*L*-phenylalaninate (MPP-4F-BUTINACA, **37**).


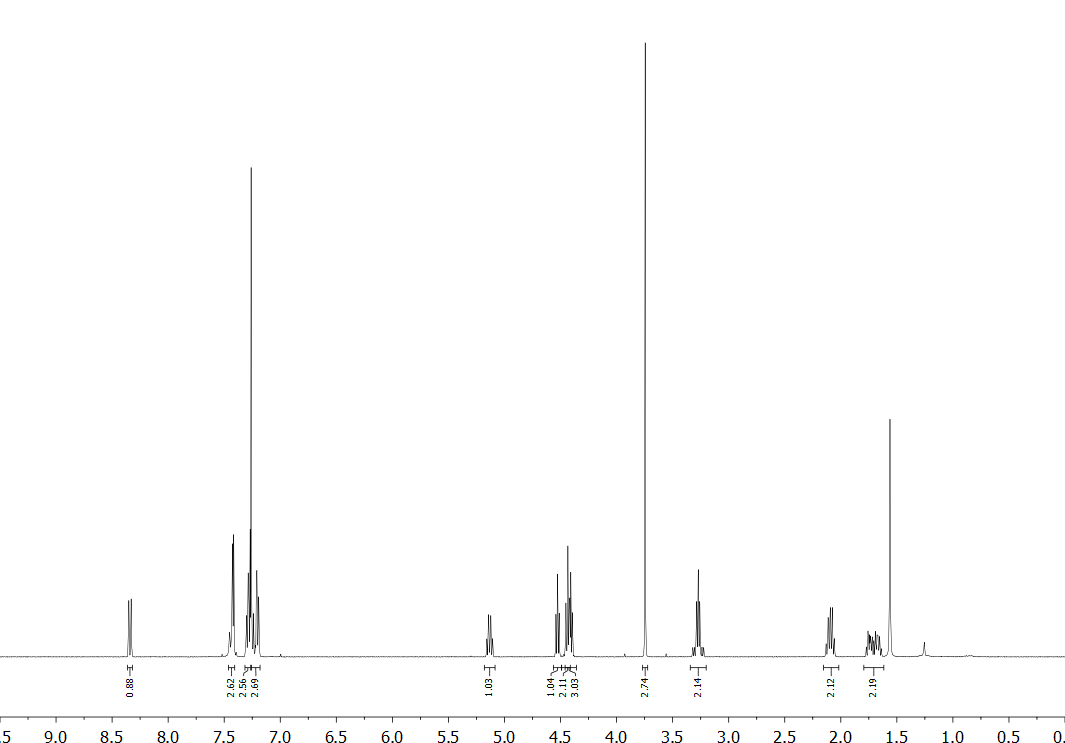


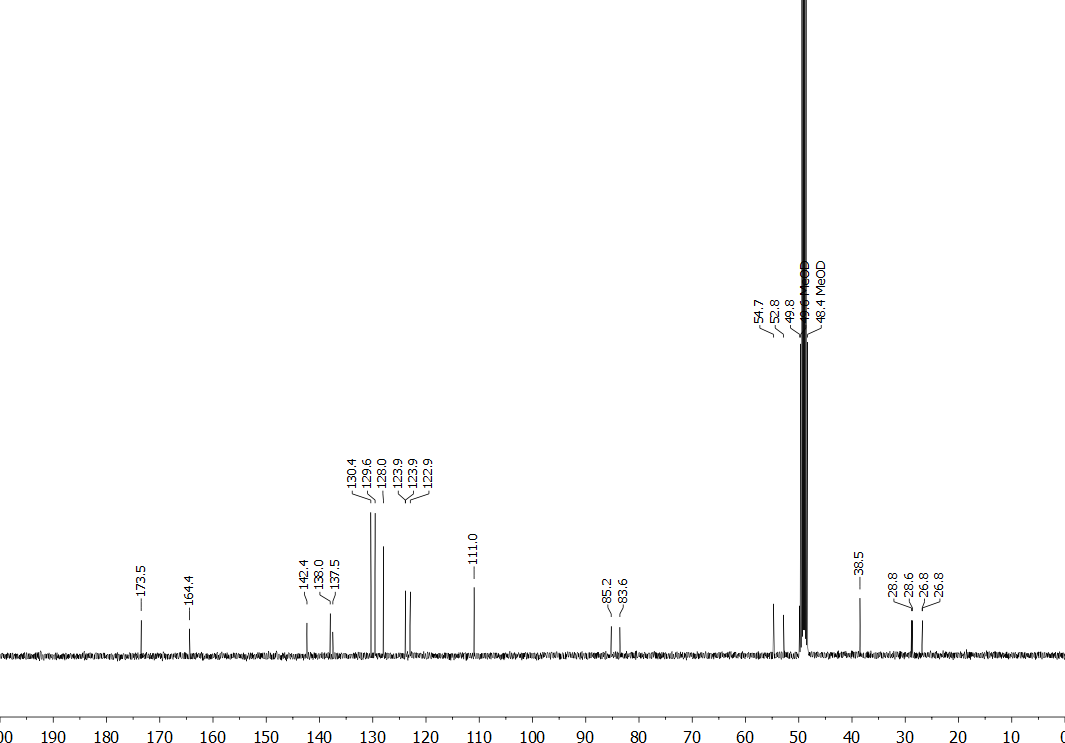


## **Figure S34.** ^1^H (400 MHz, DMSO-*d*_6_) and ^13^C (101 MHz, CD_3_OD) NMR spectra for methyl (1-(4-fluorobutyl)-1*H*-pyrrolo[2,3-*b*]pyridine-3-carbonyl)-*L*-valinate (MMB-4F-BUT7AICA¸**38**).


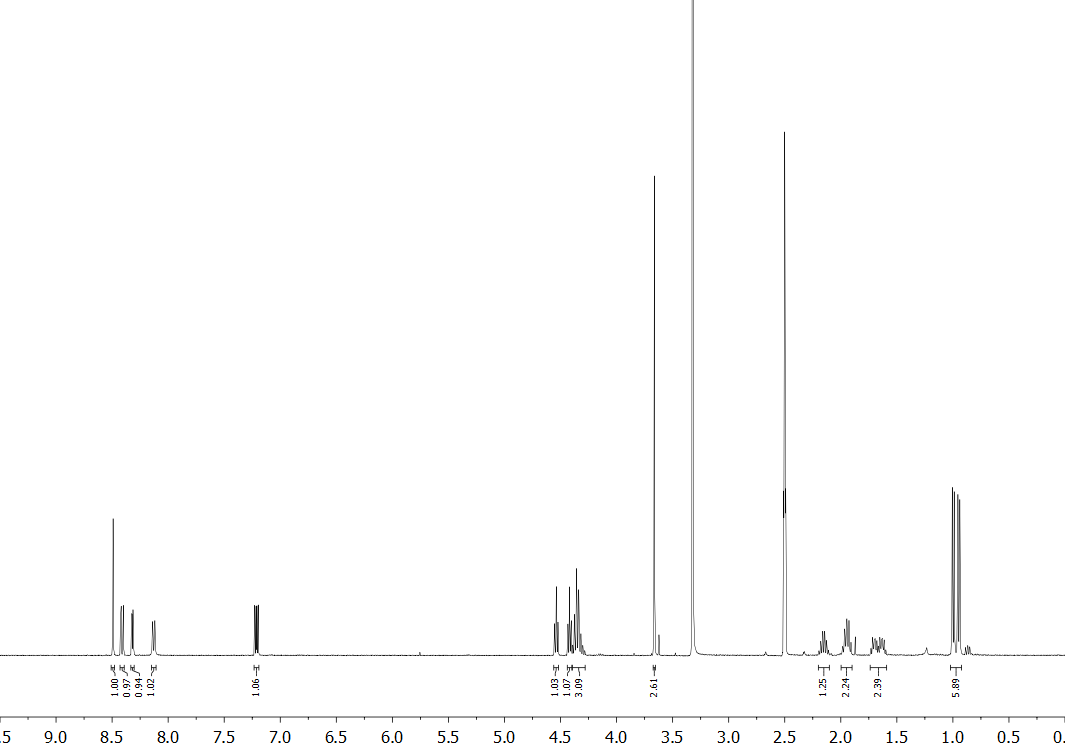


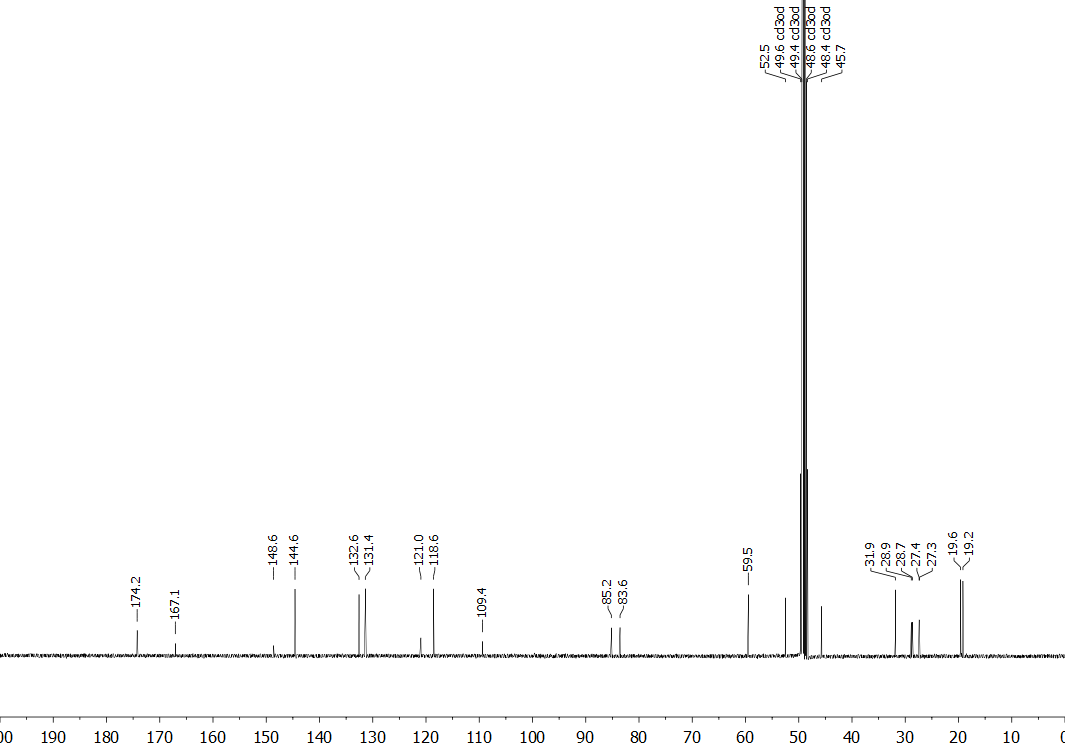


## **Figure S35.** ^1^H (400 MHz, DMSO-*d*_6_) and ^13^C (101 MHz, CD_3_OD) NMR spectra for methyl (*S*)-2-(1-(4-fluorobutyl)-1*H*-pyrrolo[2,3-*b*]pyridine-3-carboxamido)-3,3-dimethylbutanoate (MDMB-4F-BUT7AICA, **39**).


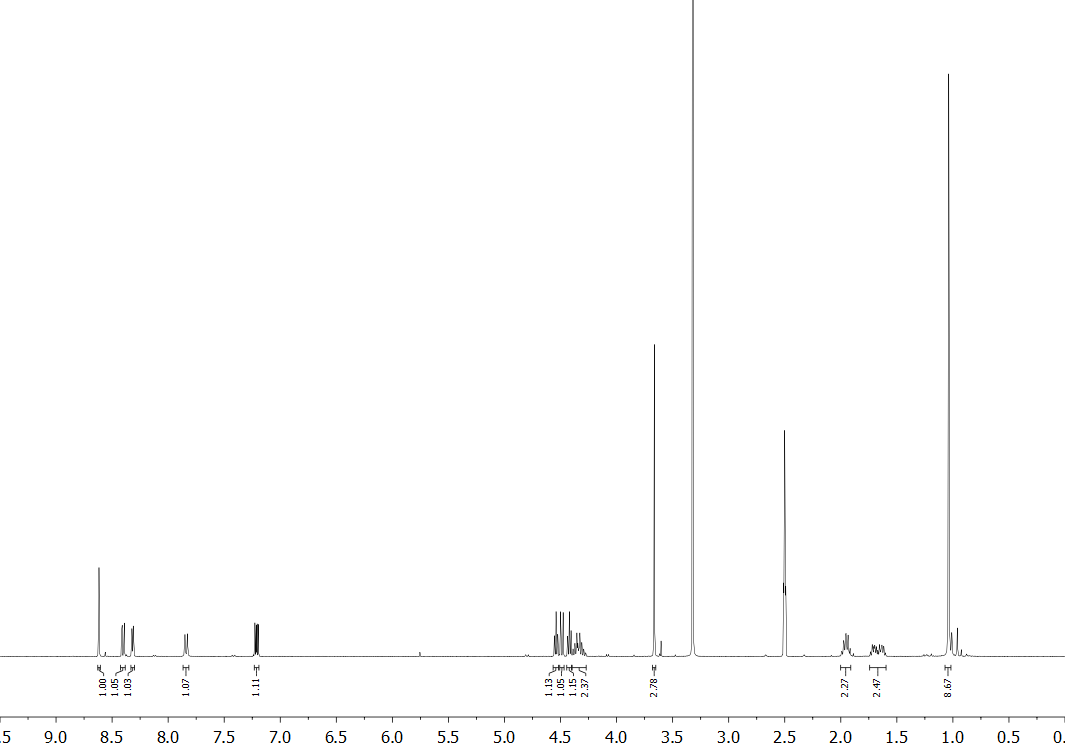


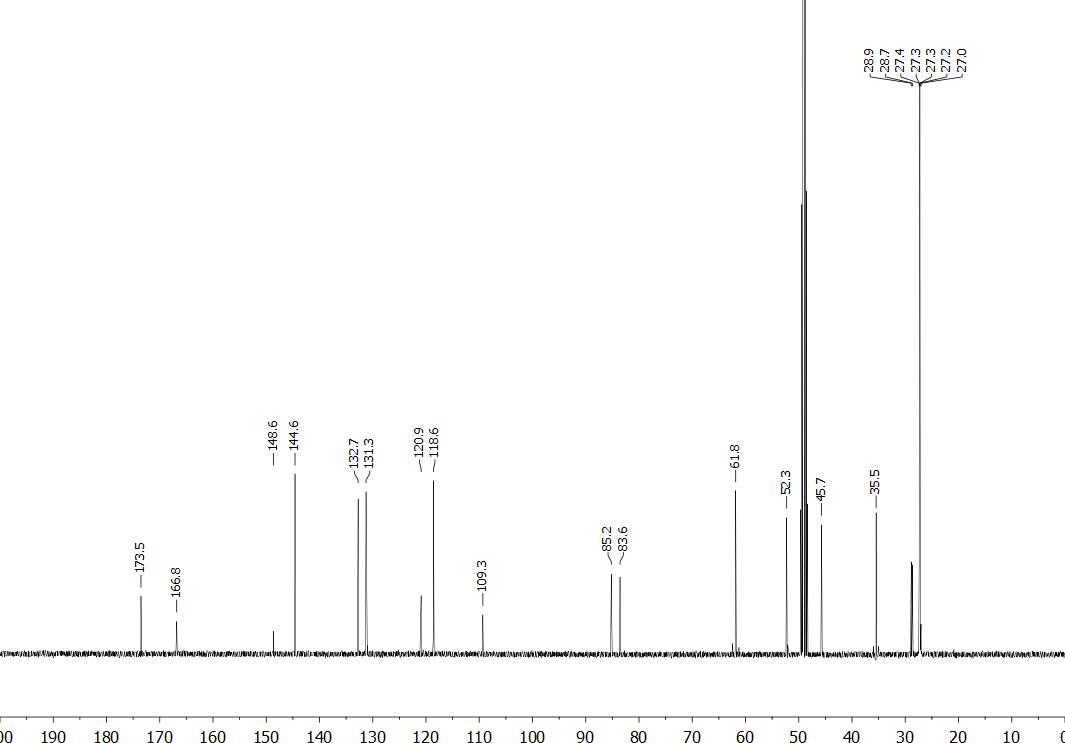


## **Figure S36.** ^1^H (400 MHz, CDCl_3_) and ^13^C (101 MHz, CD_3_OD) NMR spectra for methyl (1-(4-fluorobutyl)-1*H*-pyrrolo[2,3-*b*]pyridine-3-carbonyl)-*L*-phenylalaninate (MPP-4F-BUT7AICA, **40**).


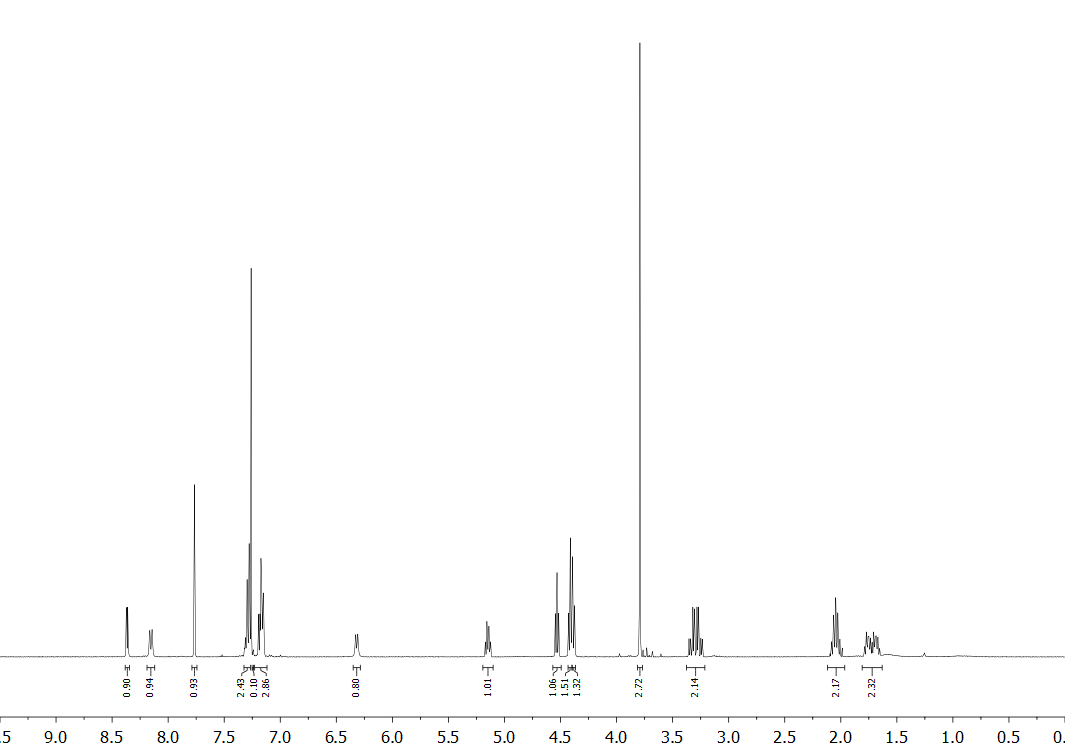


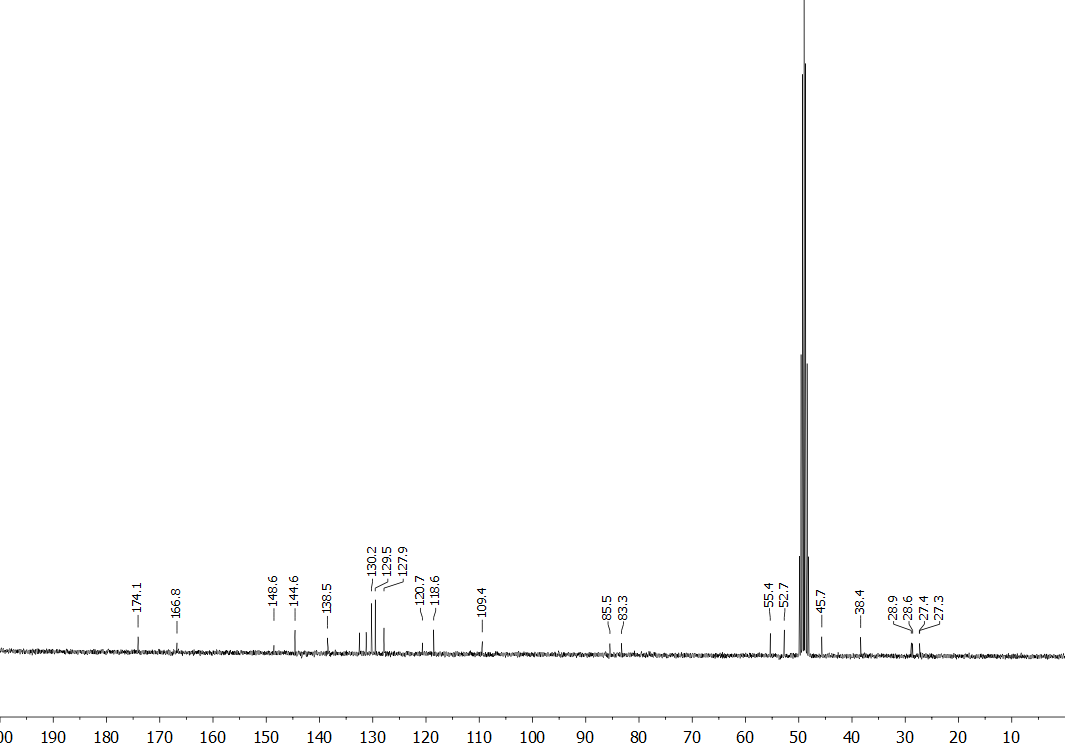


## **Figure S37.** ^1^H (400 MHz, CDCl_3_) and ^13^C (101 MHz, CDCl_3_) NMR spectra for methyl 1-(4-fluorobutyl)-1*H*-pyrrolo[2,3-*b*]pyridine-3-carboxylate (4F-BUT7AICA COOMe, **49**).


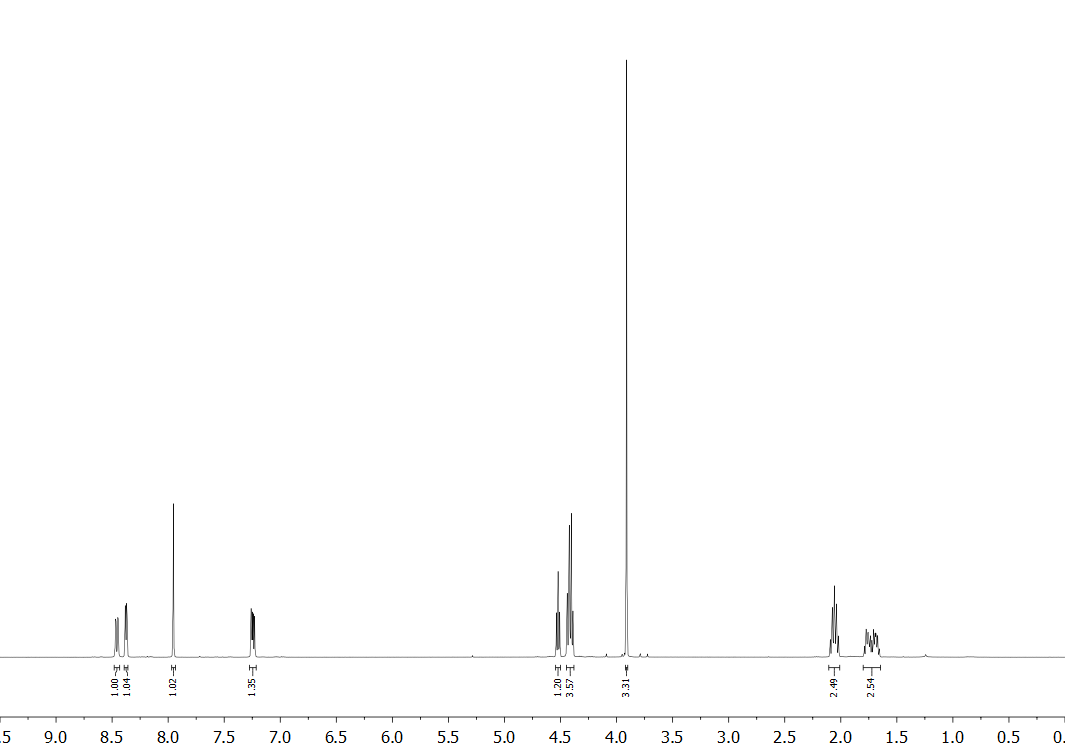


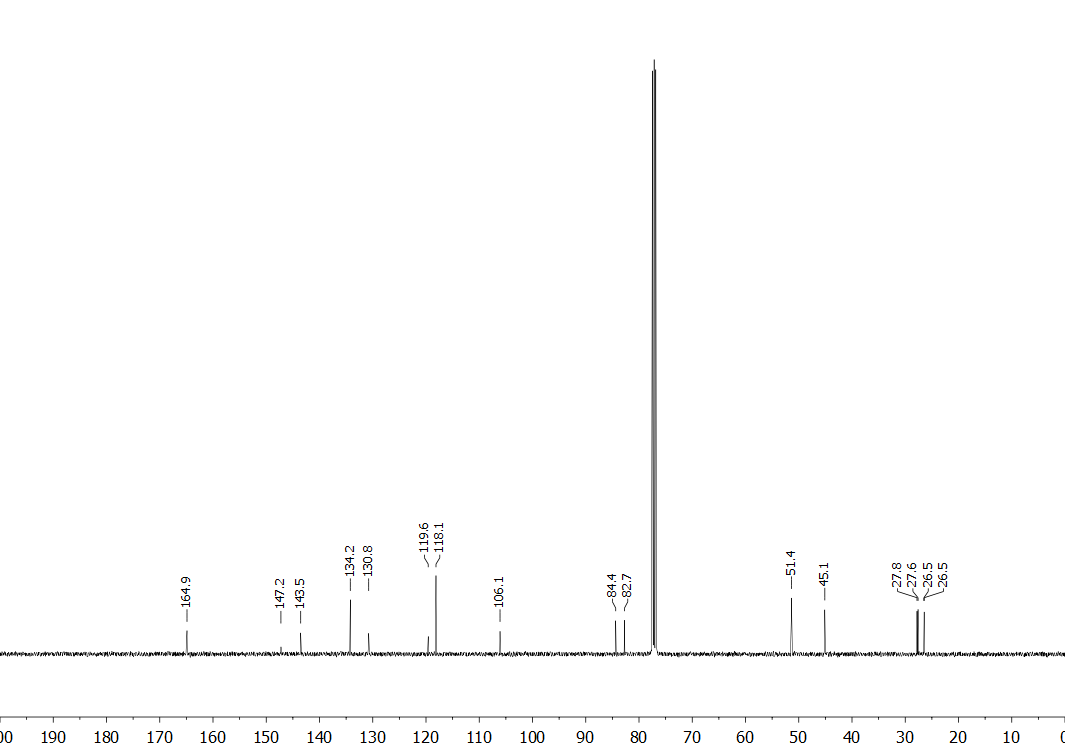


## **Figure S38.** ^1^H (400 MHz, DMSO-*d*_6_) and ^13^C (75 MHz, DMSO-*d*_6_) NMR spectra for 1-(4-fluorobutyl)-1*H*-pyrrolo[2,3-*b*]pyridine-3-carboxylic acid (4F-BUT7AICA COOH, **53**).


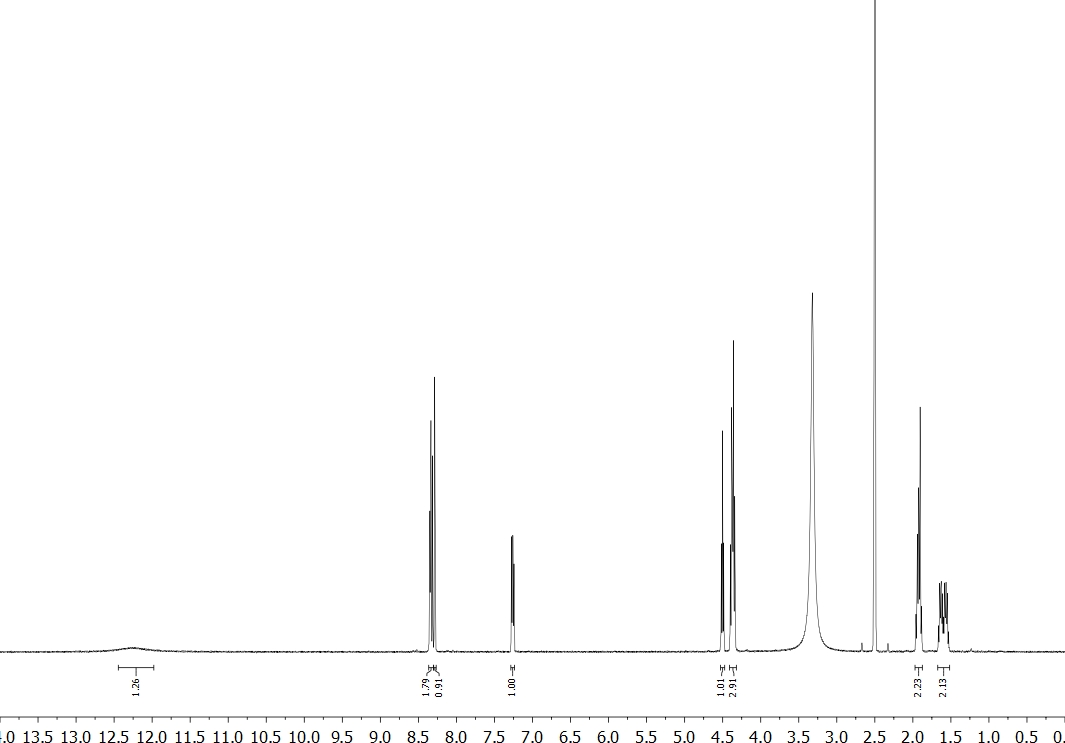


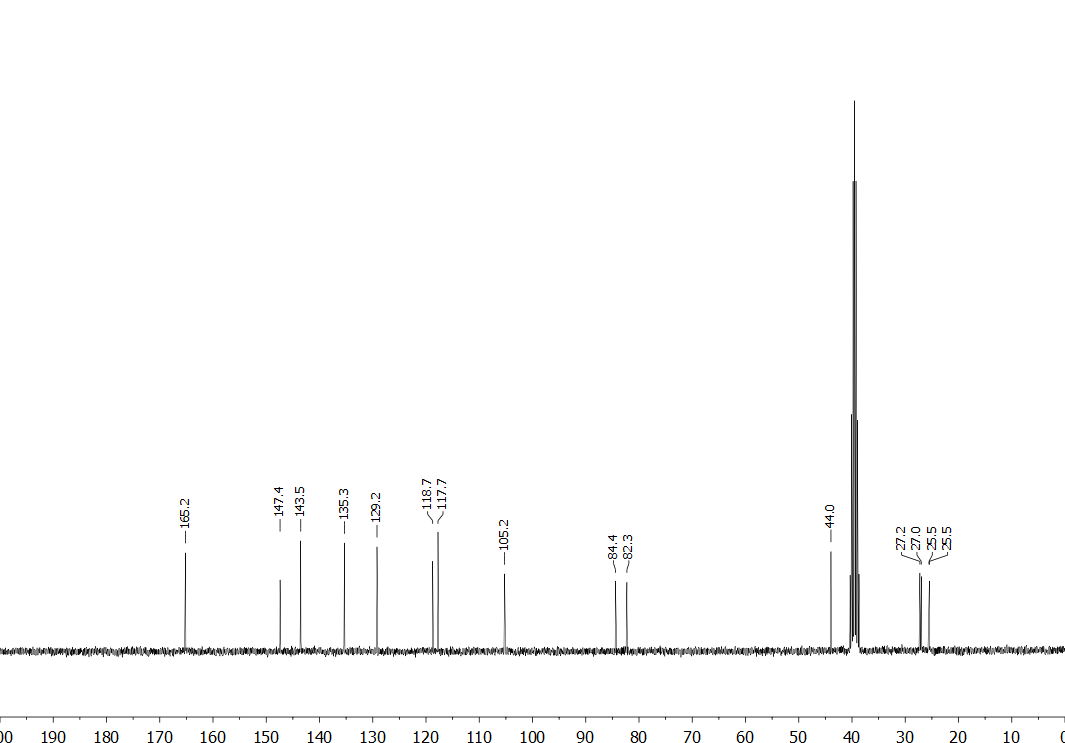


## **Figure S39.** LC-UV chromatogram for (*S*)-*N*-(1-amino-3-methyl-1-oxobutan-2-yl)-1-(4-cyanobutyl)-1*H*-indole-3-carboxamide (AB-4CN-BUTICA, **5**).


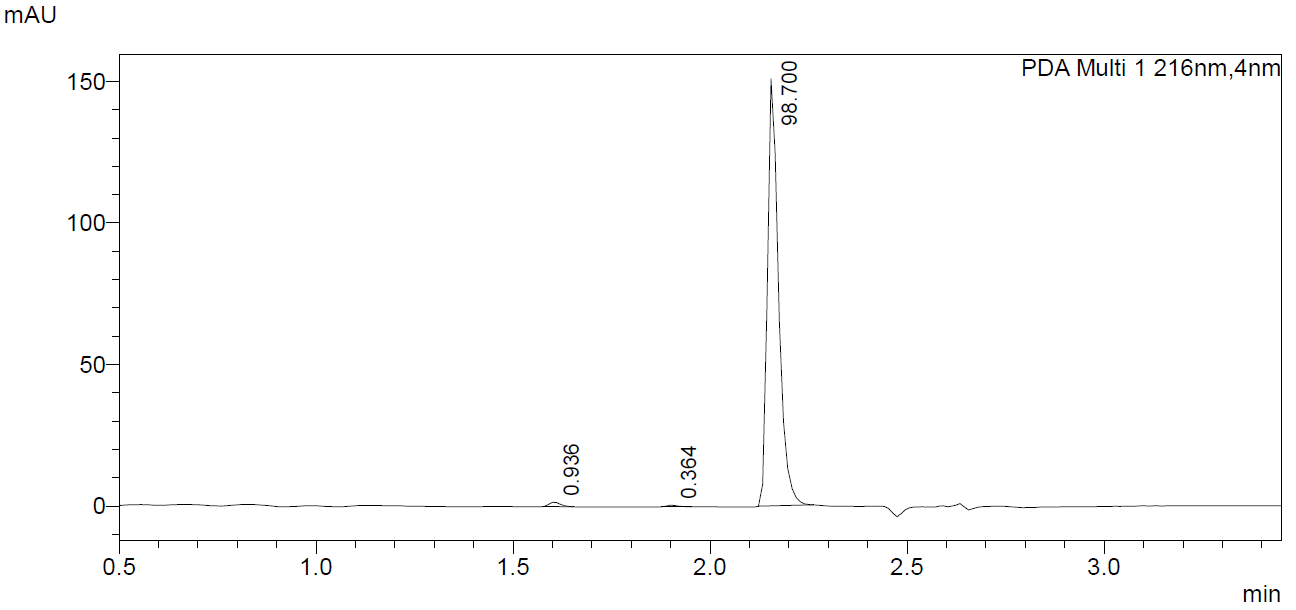


## **Figure S40.** LC-UV chromatogram for (*S*)-*N*-(1-amino-3,3-dimethyl-1-oxobutan-2-yl)-1-(4-cyanobutyl)-1*H*-indole-3-carboxamide (ADB-4CN-BUTICA, **9**).


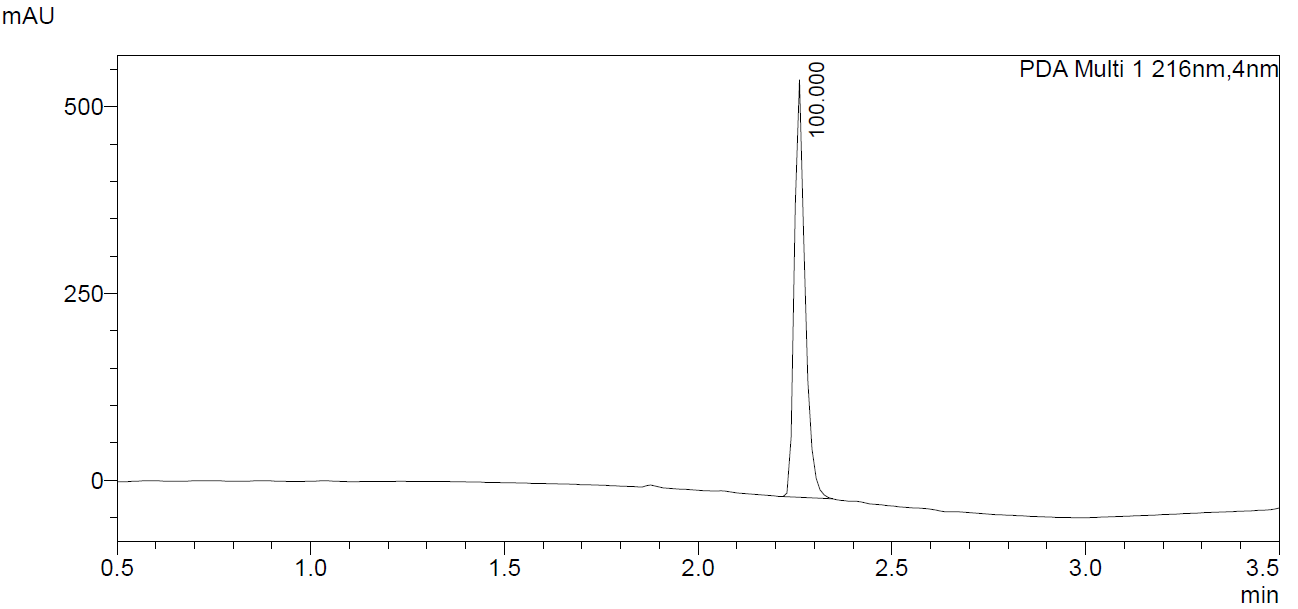


## **Figure S41.** LC-UV chromatogram for (*S*)-*N*-(1-amino-1-oxo-3-phenylpropan-2-yl)-1-(4-cyanobutyl)-1*H*-indole-3-carboxamide (APP-4CN-BUTICA, **10**).


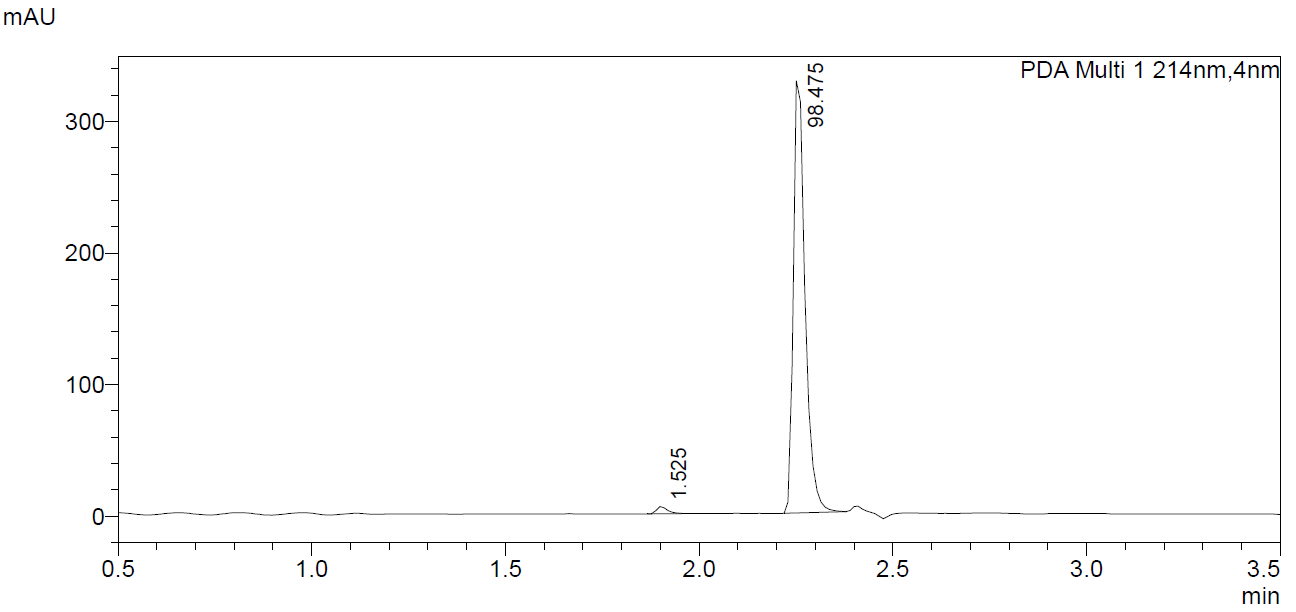


## **Figure S42.** LC-UV chromatogram for (*S*)-*N*-(1-amino-3-methyl-1-oxobutan-2-yl)-1-(4-cyanobutyl)-1*H*-indazole-3-carboxamide (AB-4CN-BUTINACA, **11**).


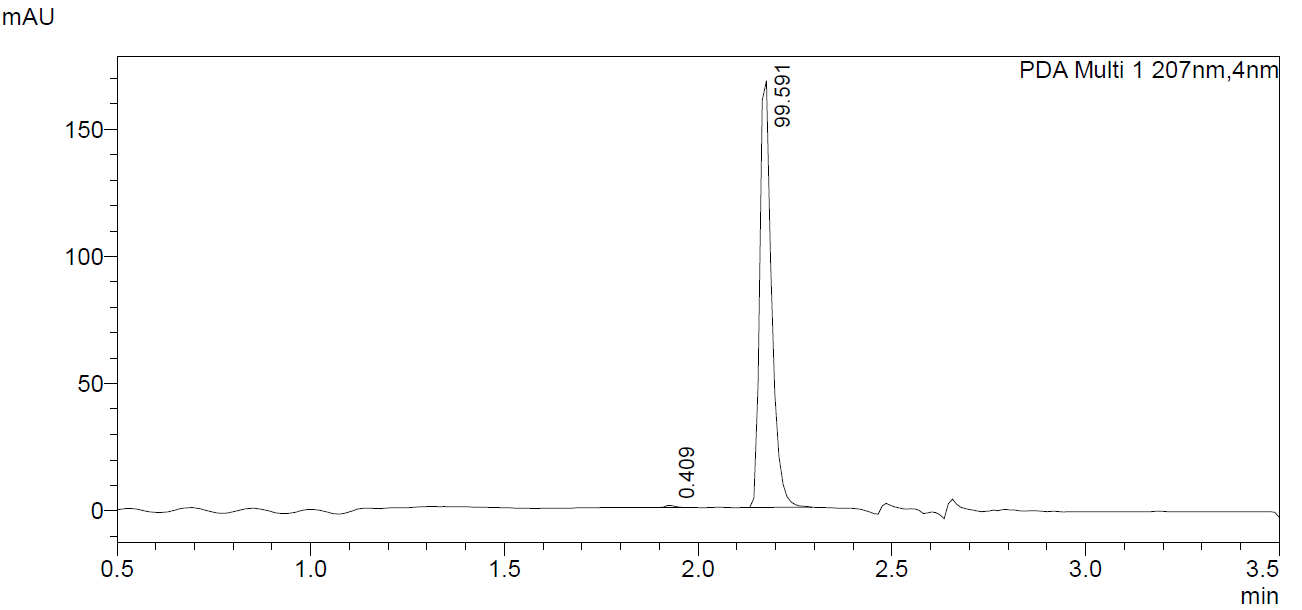


## **Figure S43.** LC-UV chromatogram for (*S*)-*N*-(1-amino-3,3-dimethyl-1-oxobutan-2-yl)-1-(4-cyanobutyl)-1*H*-indazole-3-carboxamide (ADB-4CN-BUTINACA, **12**).


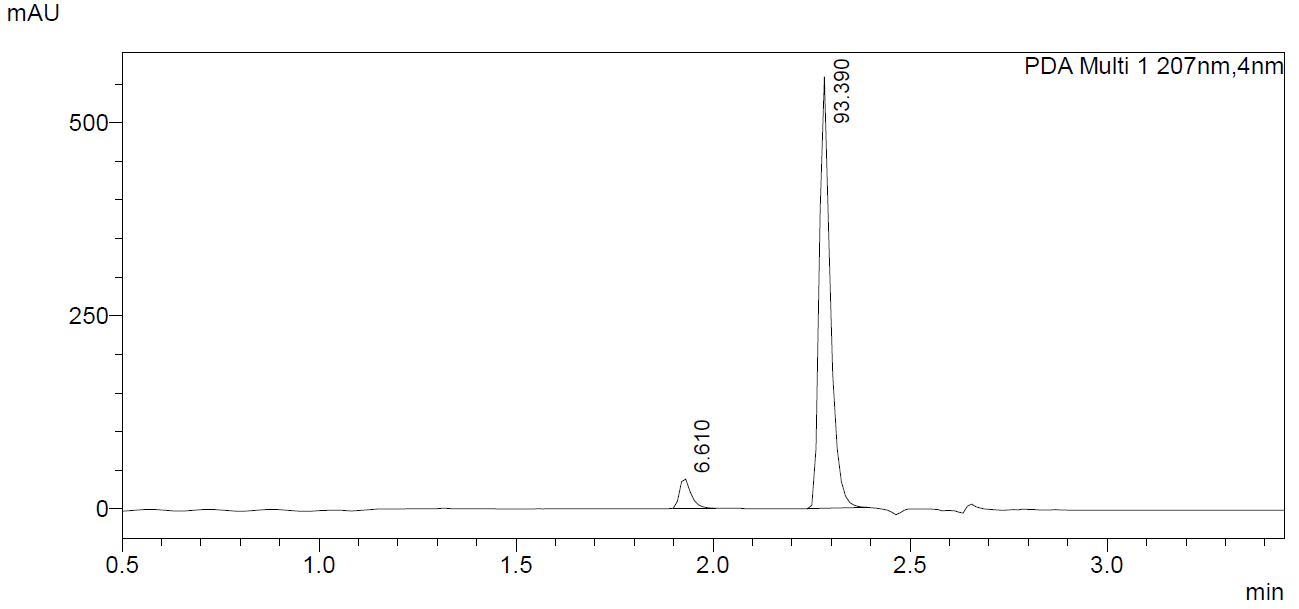


## **Figure S44.** LC-UV chromatogram for (*S*)-*N*-(1-amino-1-oxo-3-phenylpropan-2-yl)-1-(4-cyanobutyl)-1*H*-indazole-3-carboxamide (APP-4CN-BUTINACA, **13**).


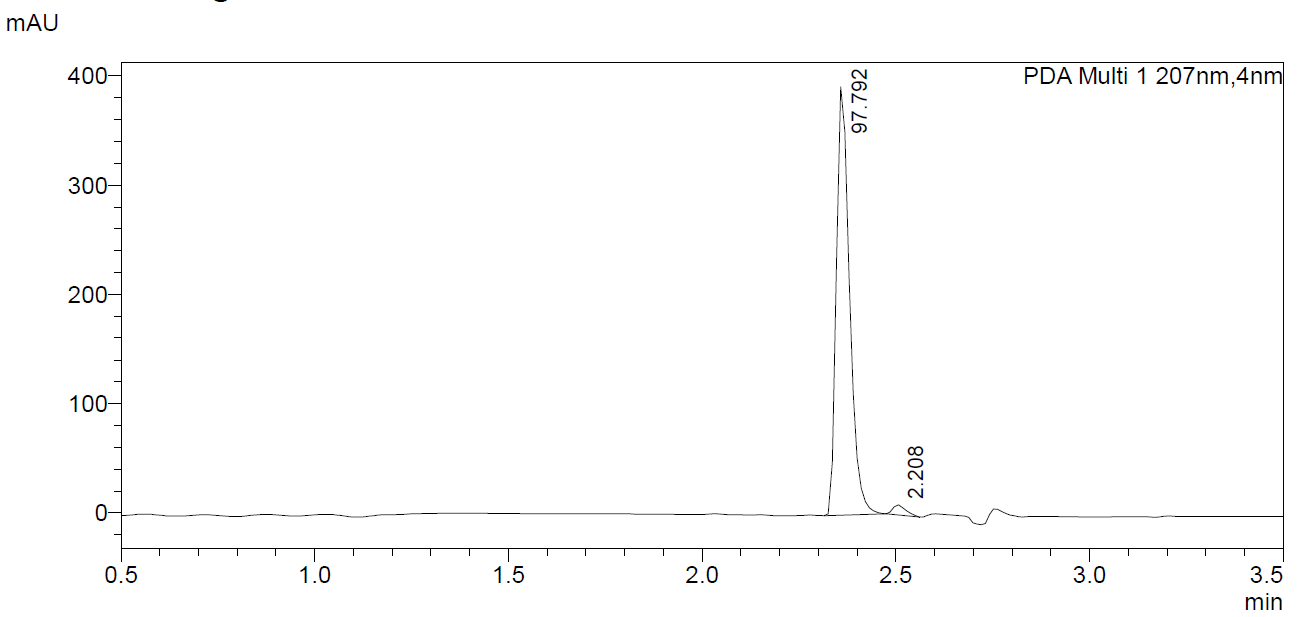


## **Figure S45.** LC-UV chromatogram for (*S*)-*N*-(1-amino-3-methyl-1-oxobutan-2-yl)-1-(4-cyanobutyl)-1*H*-pyrrolo[2,3-*b*]pyridine-3-carboxamide (AB-4CN-BUT7AICA, **14**).


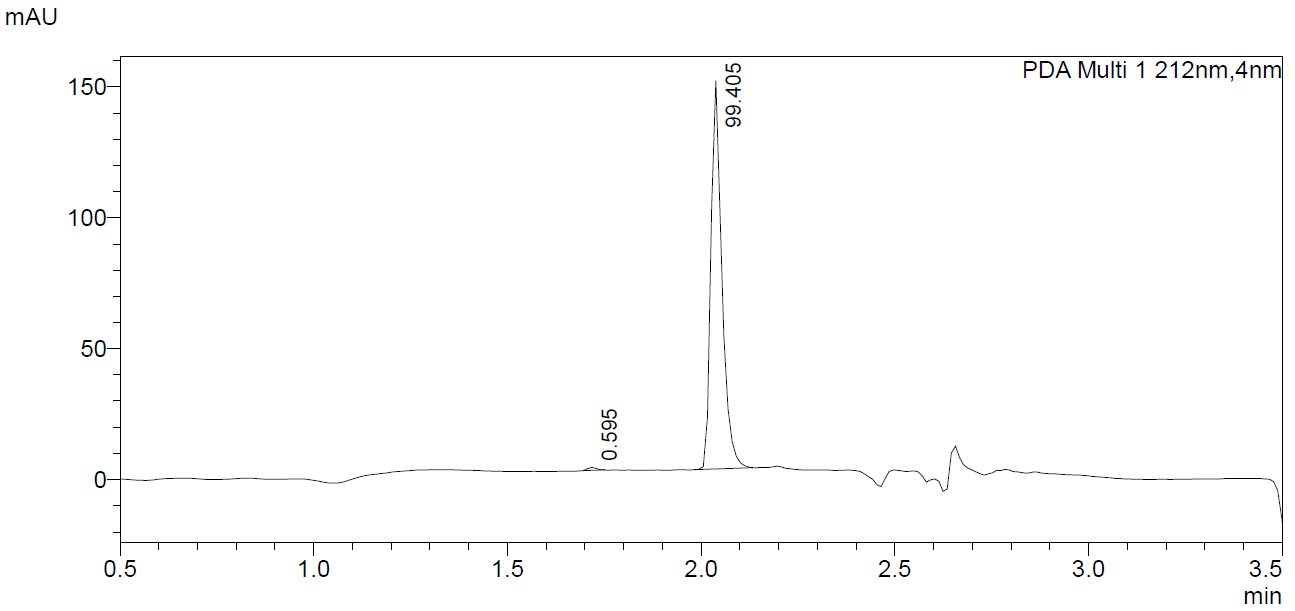


## **Figure S46.** LC-UV chromatogram for (*S*)-*N*-(1-amino-3,3-dimethyl-1-oxobutan-2-yl)-1-(4-cyanobutyl)-1*H*-pyrrolo[2,3-*b*]pyridine-3-carboxamide (ADB-4CN-BUT7AICA, **15**).


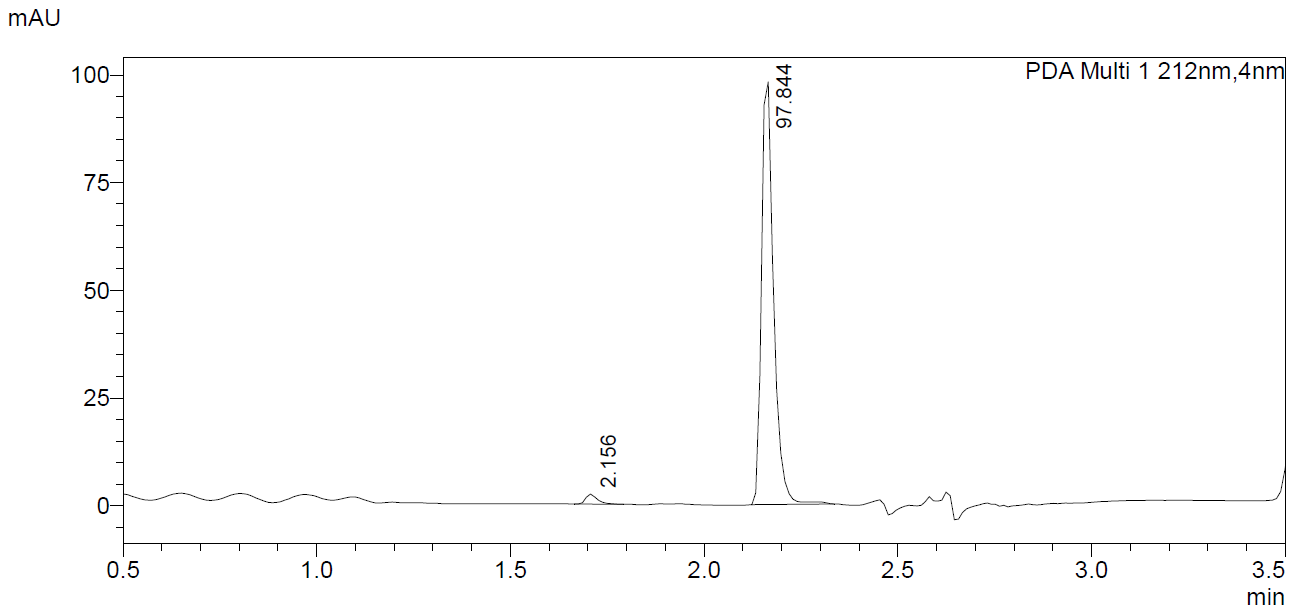


## **Figure S47.** LC-UV chromatogram for (*S*)-*N*-(1-amino-1-oxo-3-phenylpropan-2-yl)-1-(4-cyanobutyl)-1*H*-pyrrolo[2,3-*b*]pyridine-3-carboxamide (APP-4CN-BUT7AICA, **16**).


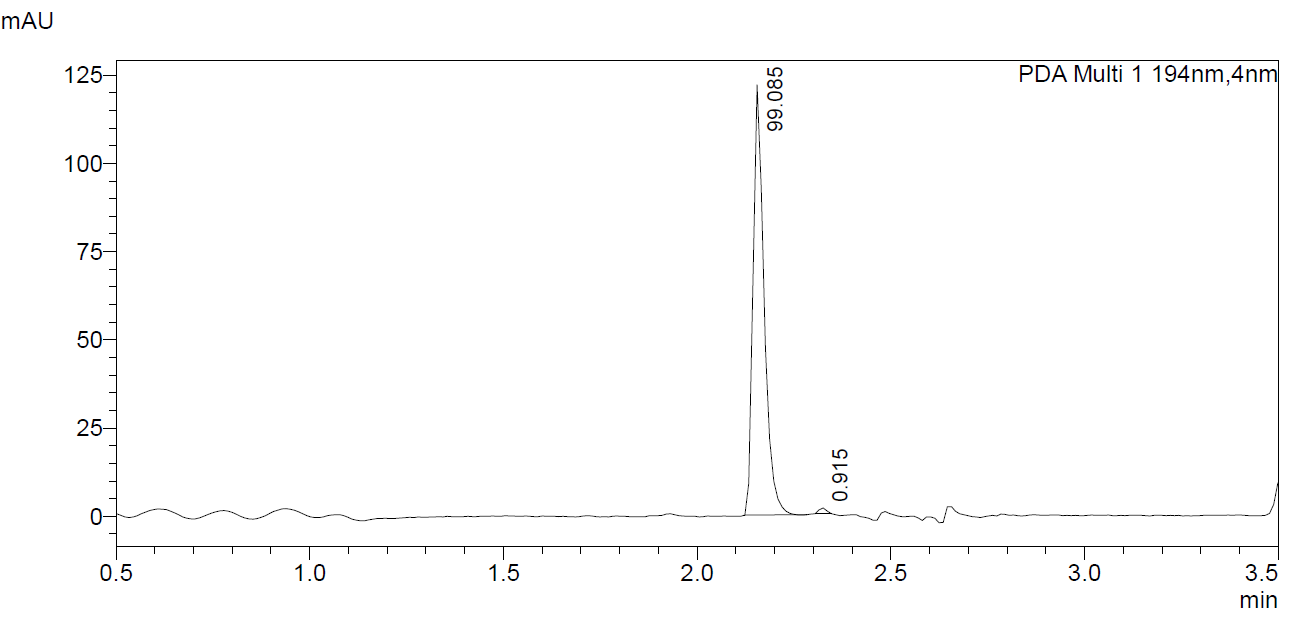


## **Figure S48.** LC-UV chromatogram for methyl (1-(4-cyanobutyl)-1*H*-indole-3-carbonyl)-*L*-valinate (MMB-4CN-BUTICA, **17**).


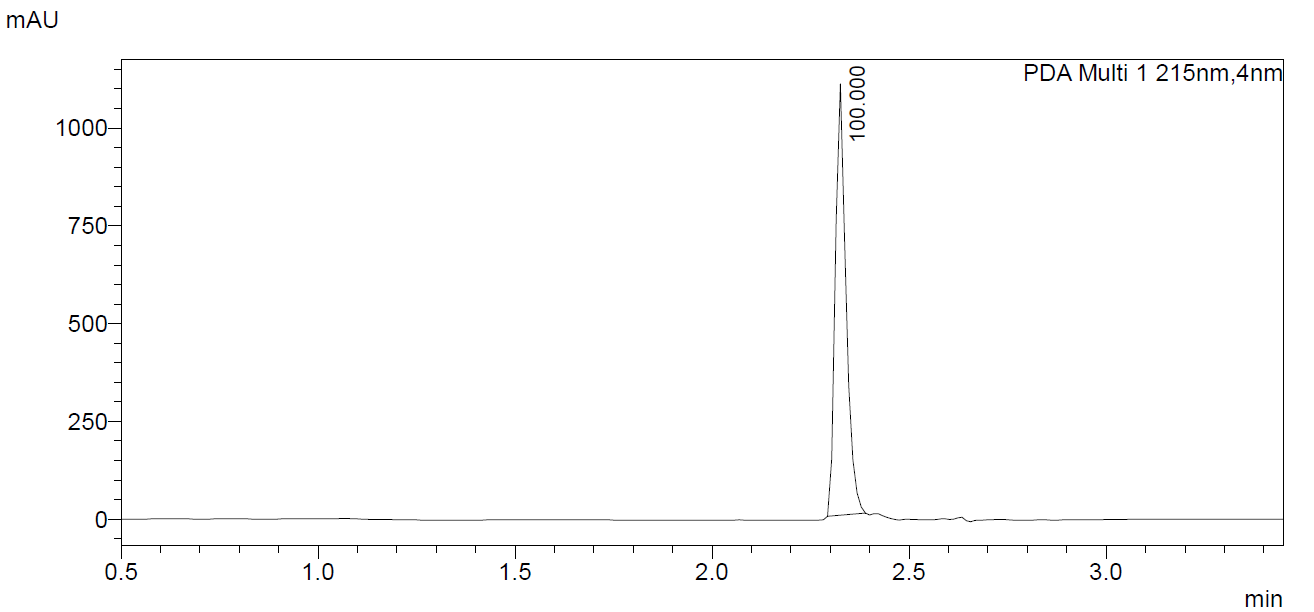


## **Figure S49.** LC-UV chromatogram for methyl (*S*)-2-(1-(4-cyanobutyl)-1*H*-indole-3-carboxamido)-3,3-dimethylbutanoate (MDMB-4CN-BUTICA, **18**).


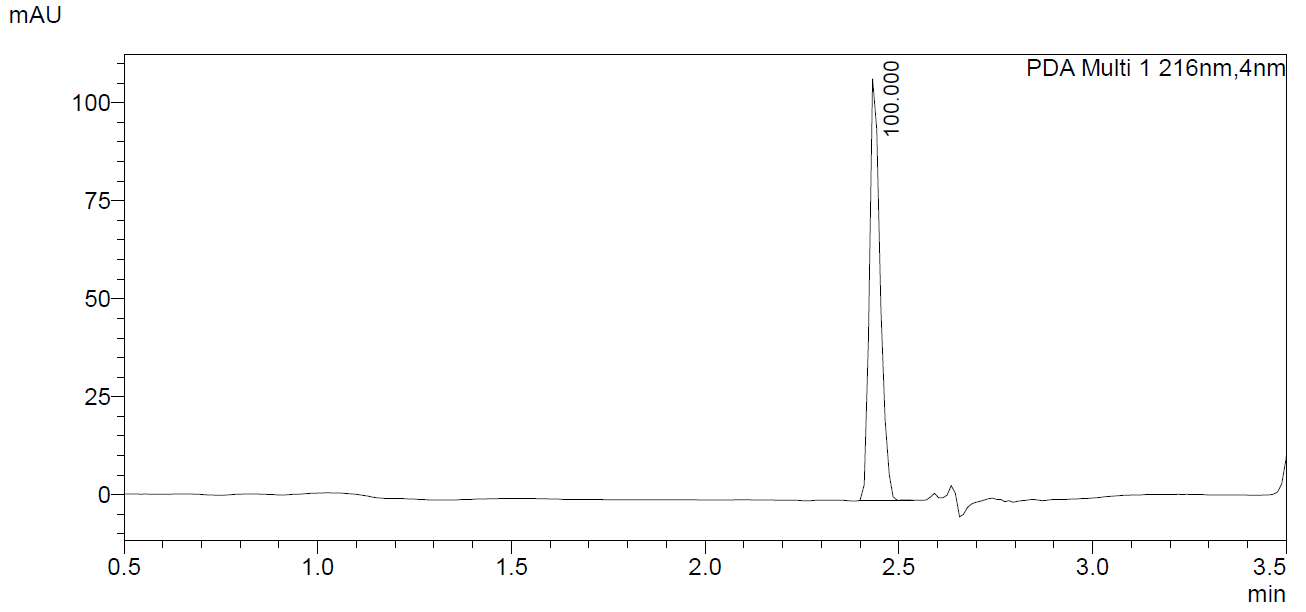


## **Figure S50.** LC-UV chromatogram for methyl (1-(4-cyanobutyl)-1*H*-indole-3-carbonyl)-*L*-phenylalaninate (MPP-4CN-BUTICA, **19**).


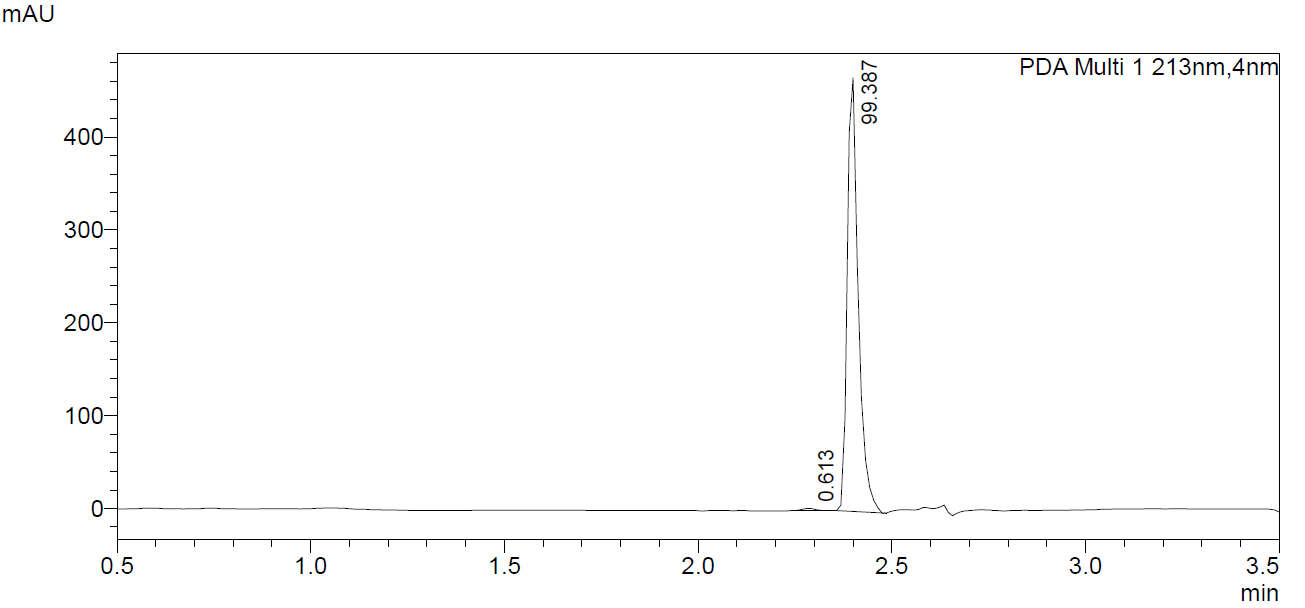


## **Figure S51.** LC-UV chromatogram for methyl (1-(4-cyanobutyl)-1*H*-indazole-3-carbonyl)-*L*-valinate (MMB-4CN-BUTINACA, **6**).


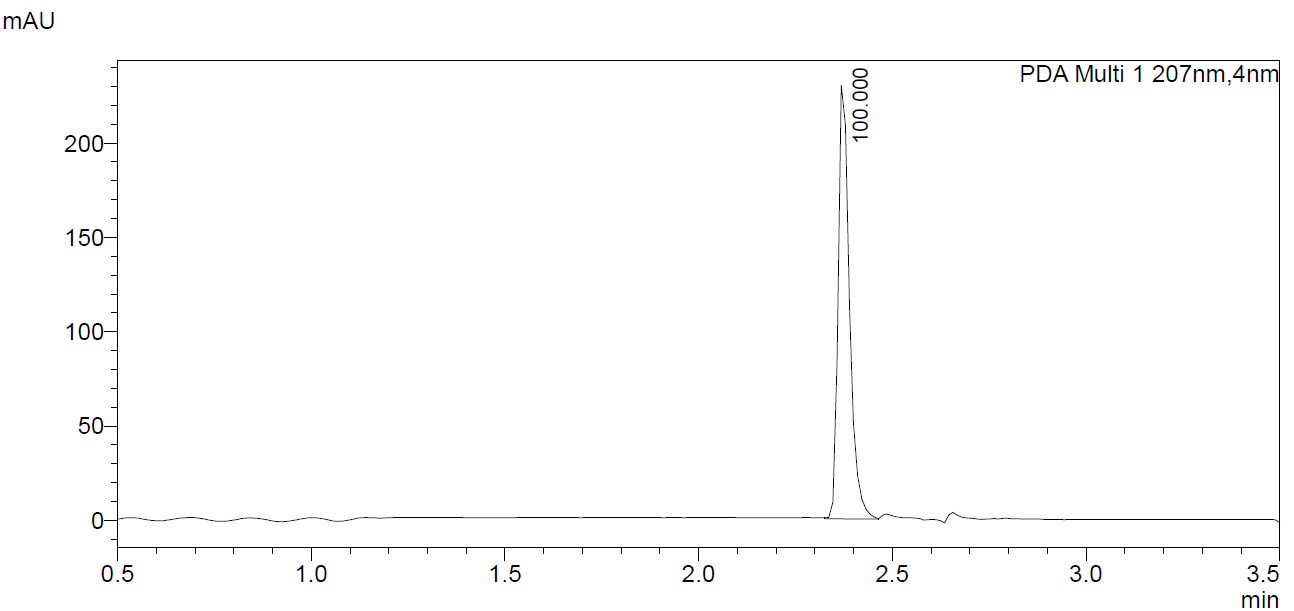


## **Figure S52.** LC-UV chromatogram for methyl (*S*)-2-(1-(4-cyanobutyl)-1*H*-indazole-3-carboxamido)-3,3-dimethylbutanoate (MDMB-4CN-BUTINACA, **20**).


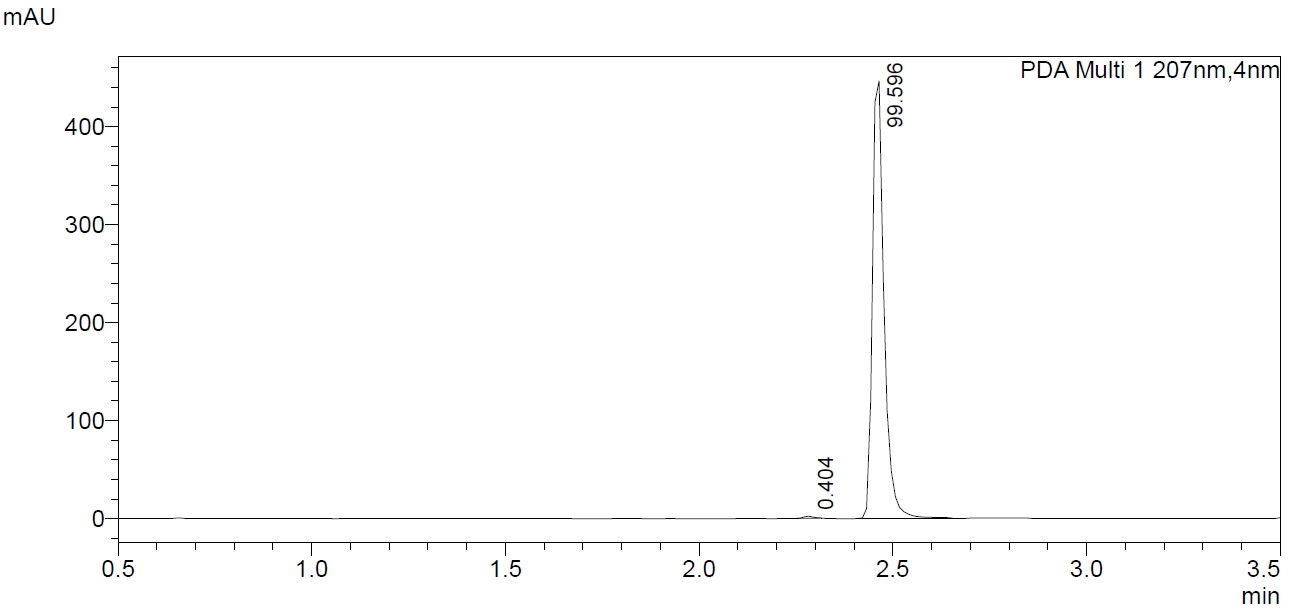


## **Figure S53.** LC-UV chromatogram for methyl (1-(4-cyanobutyl)-1*H*-indazole-3-carbonyl)-*L*-phenylalaninate (MPP-4CN-BUTINACA, **21**).


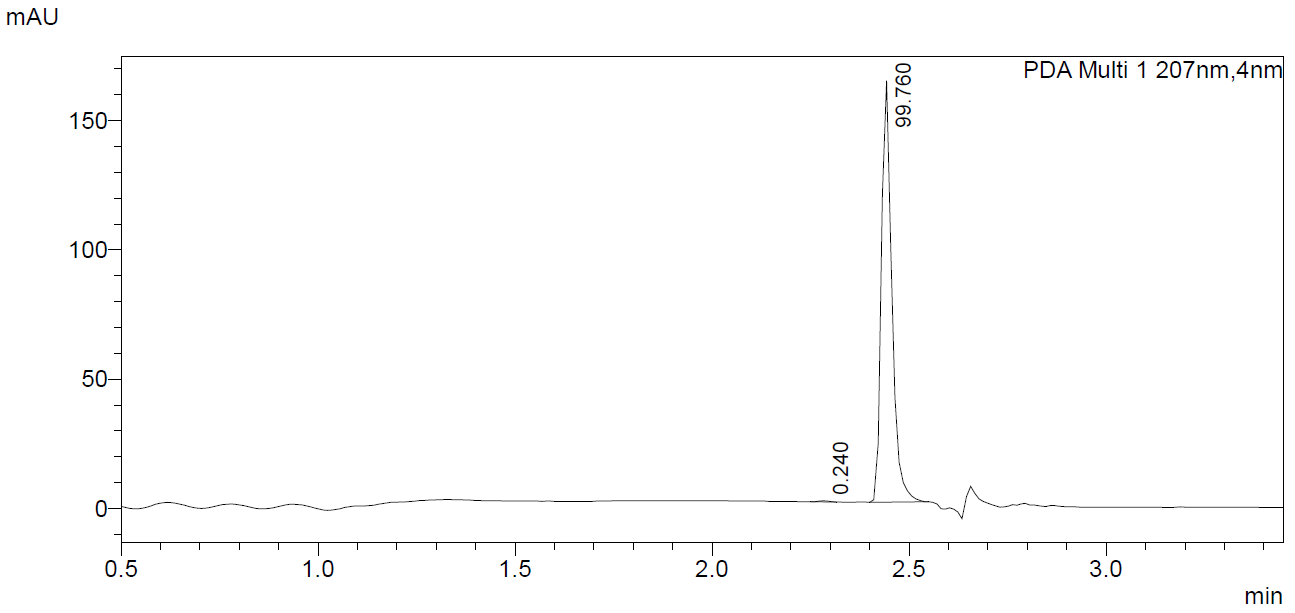


## **Figure S54.** LC-UV chromatogram for methyl (1-(4-cyanobutyl)-1*H*-pyrrolo[2,3-*b*]pyridine-3-carbonyl)-*L*-valinate (MMB-4CN-BUT7AICA, **22**).


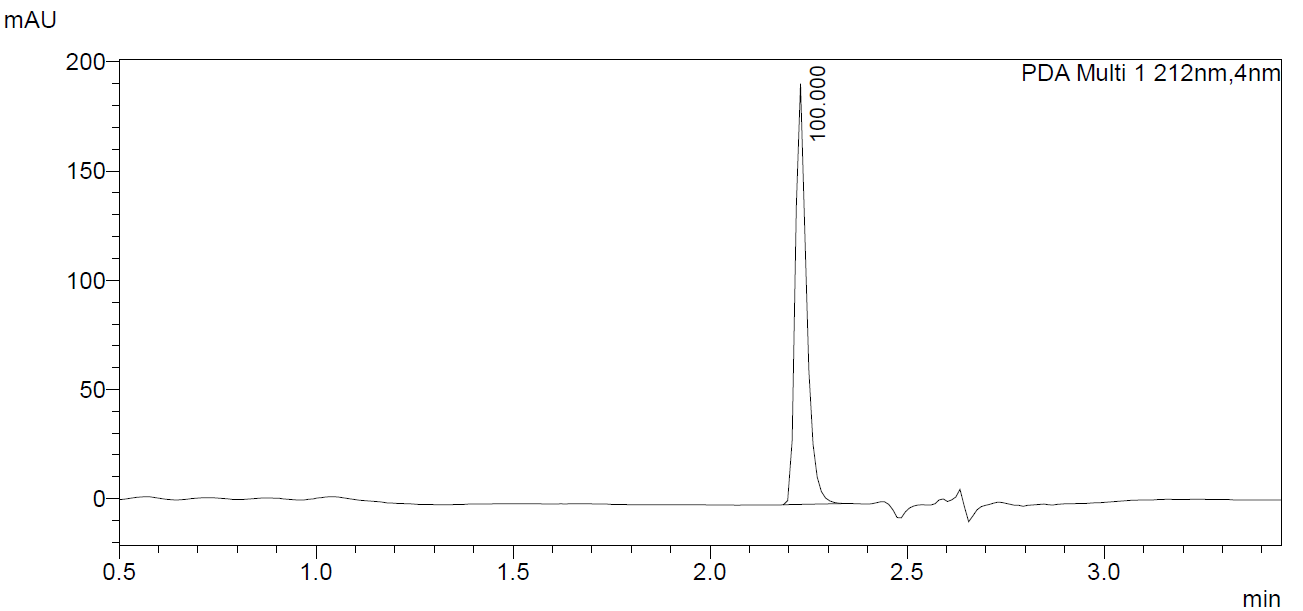


## **Figure S55.** LC-UV chromatogram for methyl (*S*)-2-(1-(4-cyanobutyl)-1*H*-pyrrolo[2,3-*b*]pyridine-3-carboxamido)-3,3-dimethylbutanoate (MDMB-4CN-BUT7AICA, **23**).


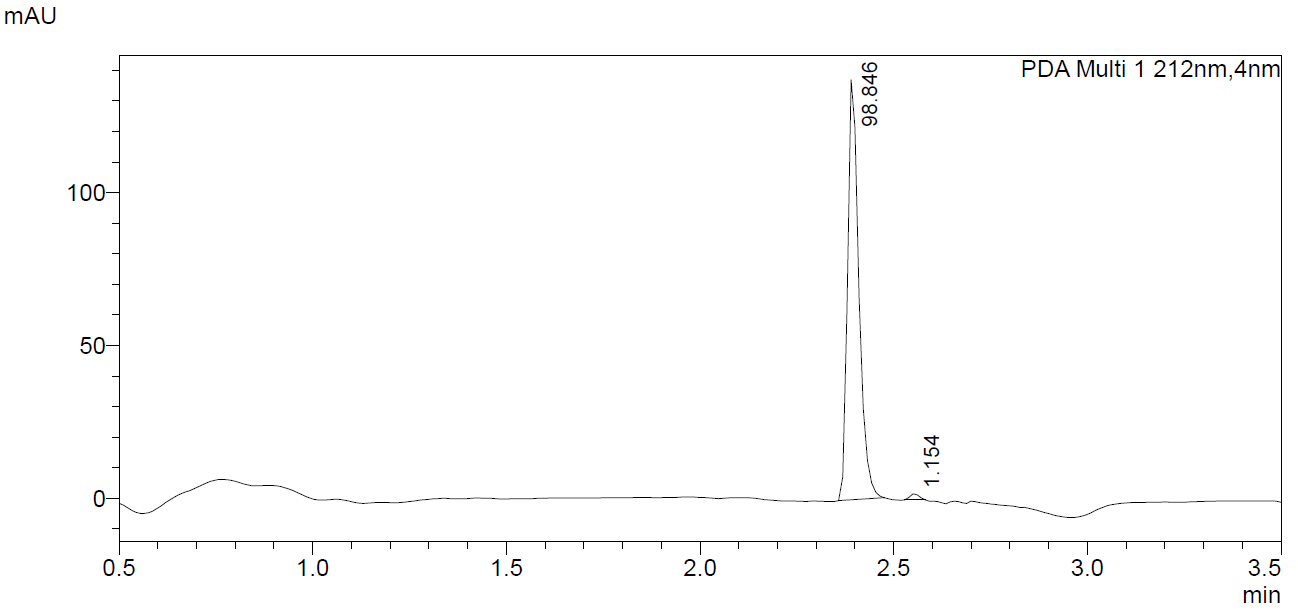


## **Figure S56.** LC-UV chromatogram for methyl (1-(4-cyanobutyl)-1*H*-pyrrolo[2,3-*b*]pyridine-3-carbonyl)-*L*-phenylalaninate (MPP-4CN-BUT7AICA, **24**).


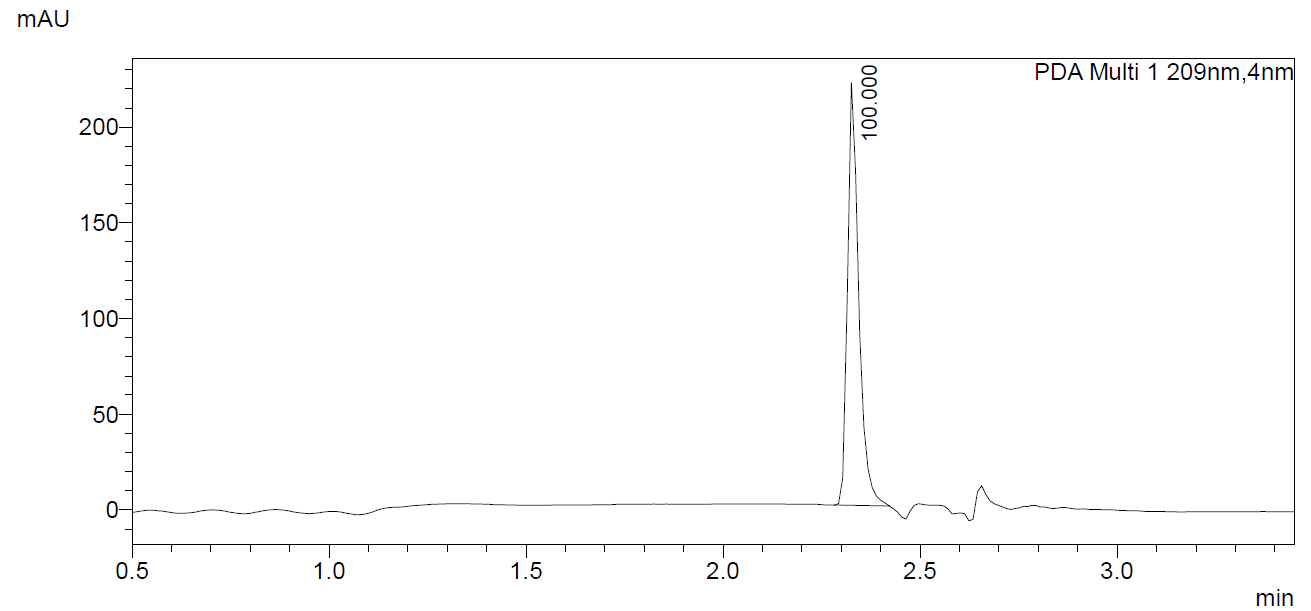


## **Figure S57.** LC-UV chromatogram for (*S*)-*N*-(1-amino-3-methyl-1-oxobutan-2-yl)-1-(4-fluorobutyl)-1*H*-indole-3-carboxamide (AB-4F-BUTICA, **25**).


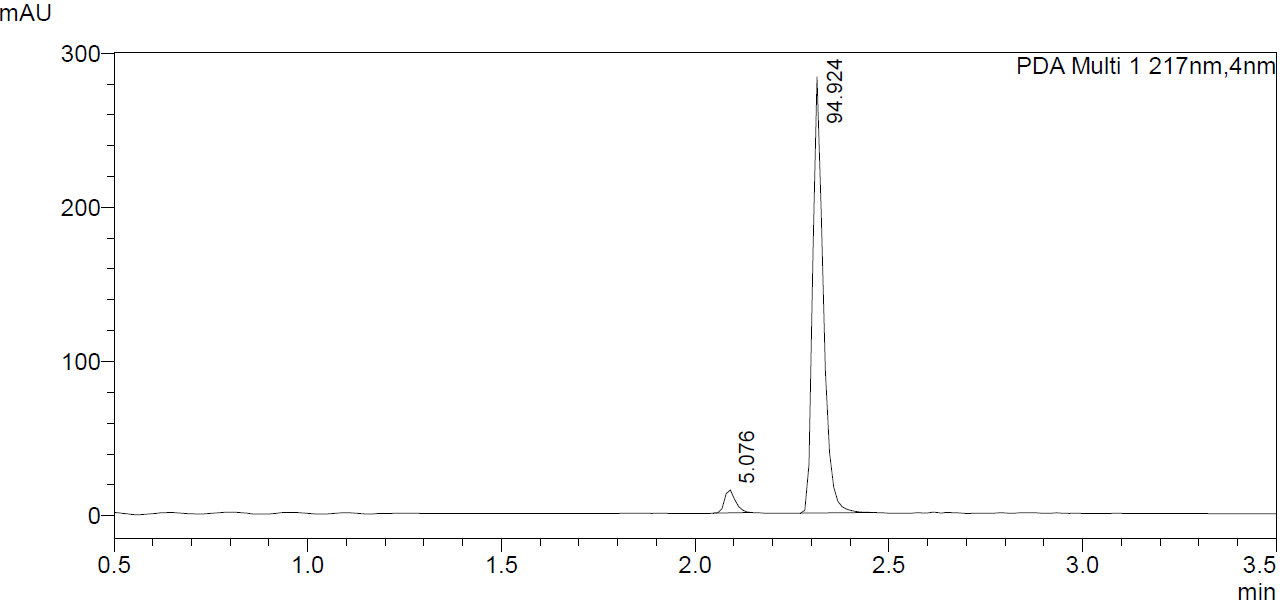


## **Figure S58.** LC-UV chromatogram for (*S*)-*N*-(1-amino-3,3-dimethyl-1-oxobutan-2-yl)-1-(4-fluorobutyl)-1*H*-indole-3-carboxamide (ADB-4F-BUTICA, **26**).


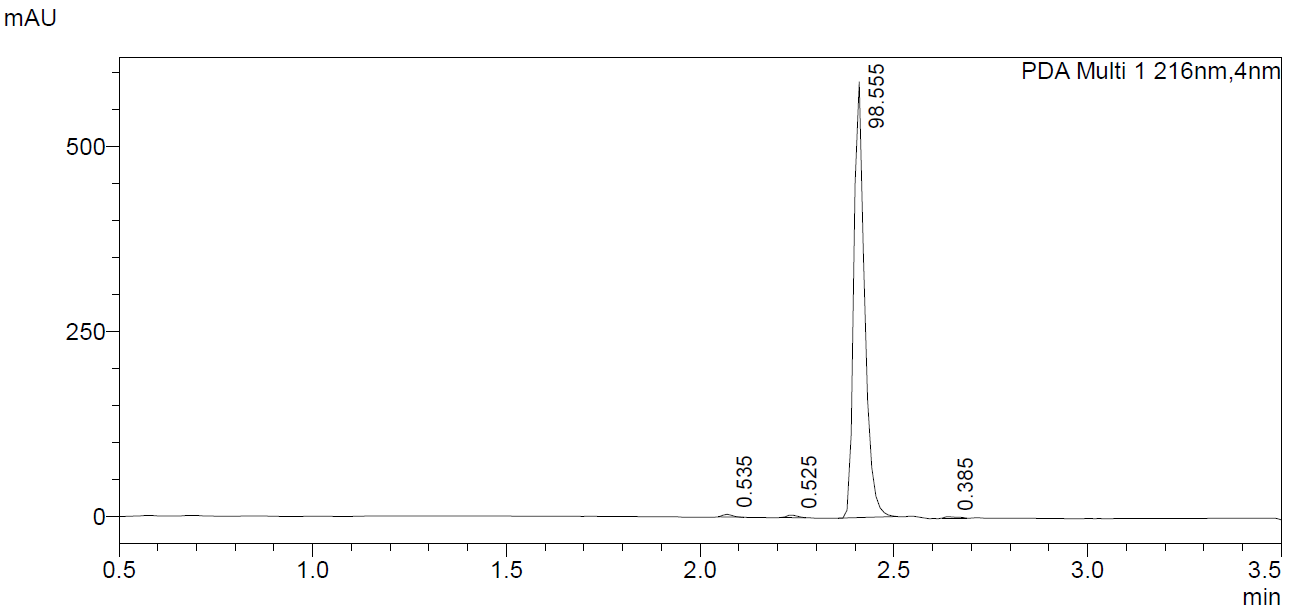


## **Figure S59.** LC-UV chromatogram for (*S*)-*N*-(1-amino-1-oxo-3-phenylpropan-2-yl)-1-(4-fluorobutyl)-1*H*-indole-3-carboxamide (APP-4F-BUTICA, **27**).


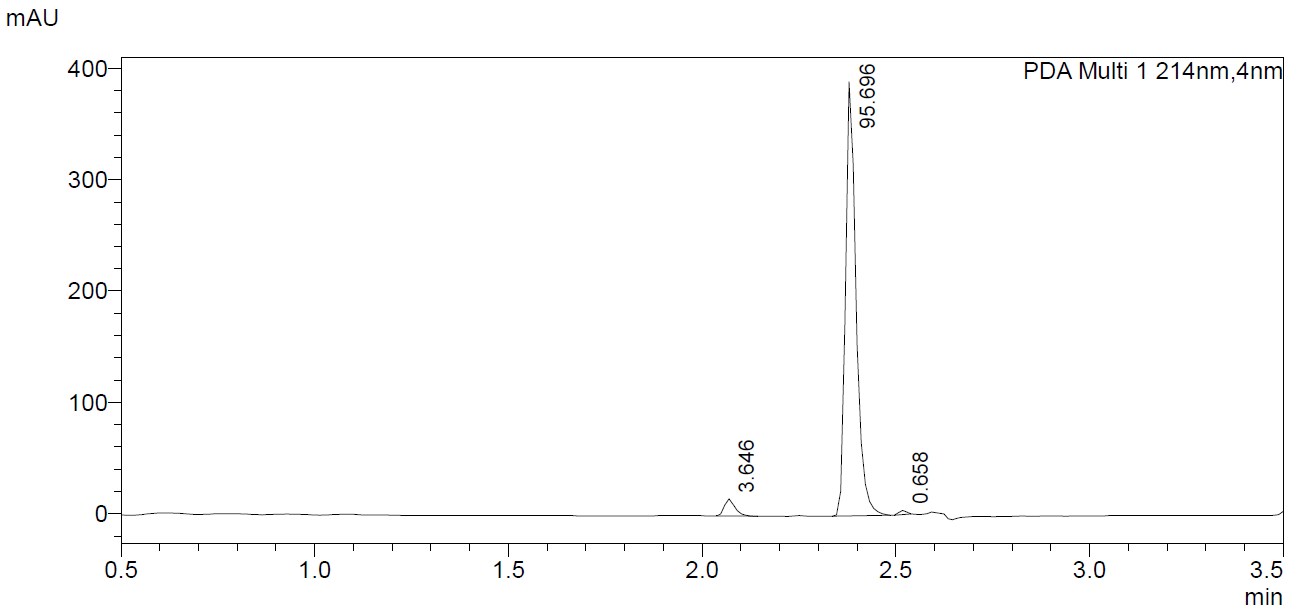


## **Figure S60.** LC-UV chromatogram for (*S*)-*N*-(1-amino-3-methyl-1-oxobutan-2-yl)-1-(4-fluorobutyl)-1*H*-indazole-3-carboxamide (AB-4F-BUTINACA, **28**).


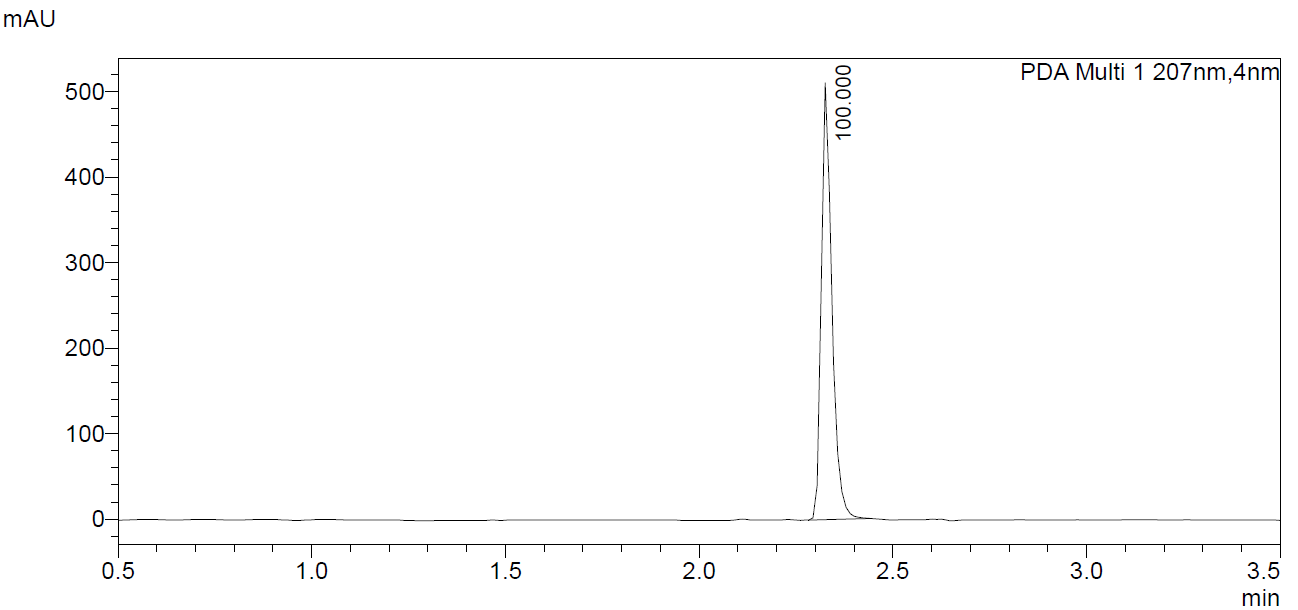


## **Figure S61.** LC-UV chromatogram for (*S*)-*N*-(1-amino-3,3-dimethyl-1-oxobutan-2-yl)-1-(4-fluorobutyl)-1*H*-indazole-3-carboxamide (ADB-4F-BUTINACA, **29**).


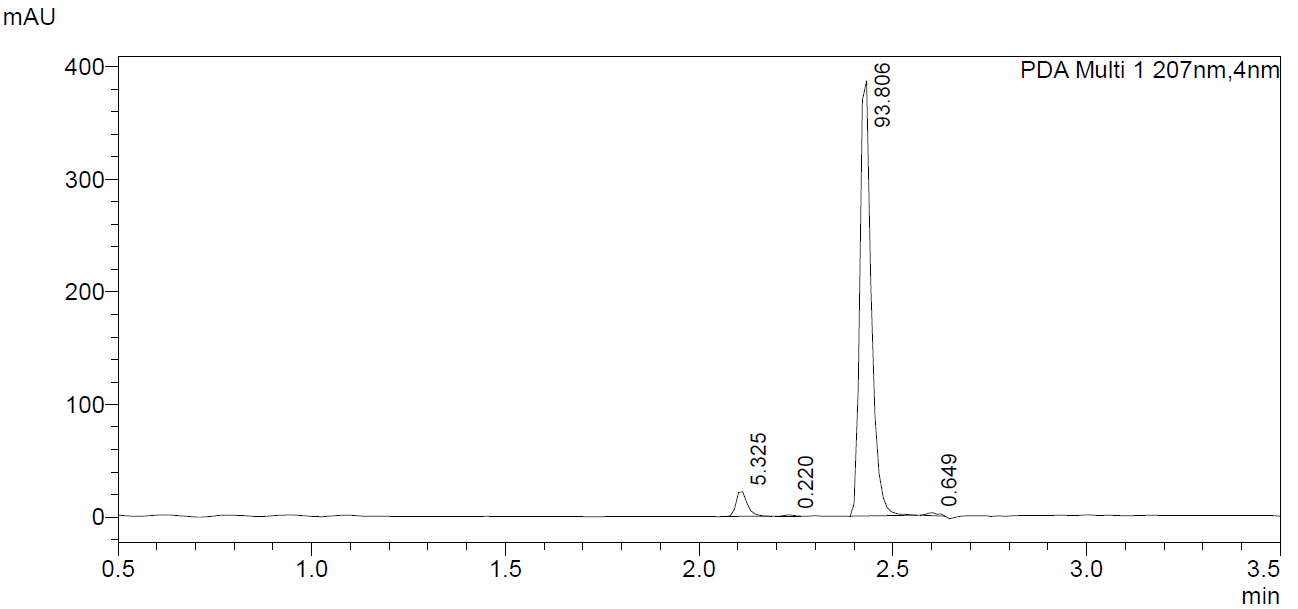


## **Figure S62.** LC-UV chromatogram for (*S*)-*N*-(1-amino-1-oxo-3-phenylpropan-2-yl)-1-(4-fluorobutyl)-1*H*-indazole-3-carboxamide (APP-4F-BUTINACA, **30**).


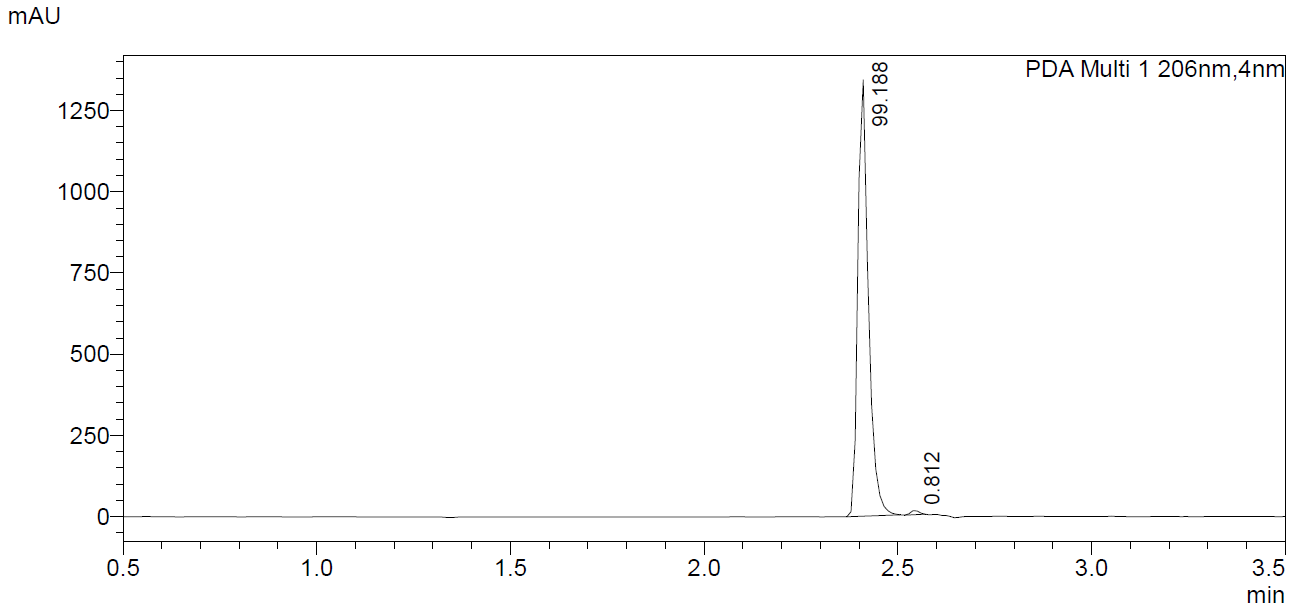


## **Figure S63.** LC-UV chromatogram for (*S*)-*N*-(1-amino-3-methyl-1-oxobutan-2-yl)-1-(4-fluorobutyl)-1*H*-pyrrolo[2,3-*b*]pyridine-3-carboxamide (AB-4F-BUT7AICA, **31**).

## **Figure S64.** LC-UV chromatogram for (*S*)-*N*-(1-amino-3,3-dimethyl-1-oxobutan-2-yl)-1-(4-fluorobutyl)-1*H*-pyrrolo[2,3-*b*]pyridine-3-carboxamide (ADB-4F-BUT7AICA, **32**).

## **Figure S65.** LC-UV chromatogram for (*S*)-*N*-(1-amino-1-oxo-3-phenylpropan-2-yl)-1-(4-fluorobutyl)-1*H*-pyrrolo[2,3-*b*]pyridine-3-carboxamide (APP-4F-BUT7AICA, **33**).

## **Figure S66.** LC-UV chromatogram for methyl (1-(4-fluorobutyl)-1*H*-indole-3-carbonyl)-*L*-valinate (MMB-4F-BUTICA, **34**).

## **Figure S67.** LC-UV chromatogram for methyl (*S*)-2-(1-(4-fluorobutyl)-1*H*-indole-3-carboxamido)-3,3-dimethylbutanoate (MDMB-4F-BUTICA, **7**).

## **Figure S68.** LC-UV chromatogram for methyl (1-(4-fluorobutyl)-1*H*-indole-3-carbonyl)-*L*-phenylalaninate (MPP-4F-BUTICA, **35**).

## **Figure S69.** LC-UV chromatogram for methyl (1-(4-fluorobutyl)-1*H*-indazole-3-carbonyl)-*L*-valinate (MMB-4F-BUTINACA, **36**).

## **Figure S70.** LC-UV chromatogram for methyl (*S*)-2-(1-(4-fluorobutyl)-1*H*-indazole-3-carboxamido)-3,3-dimethylbutanoate (MDMB-4F-BUTINACA, **8**).

## **Figure S71.** LC-UV chromatogram for methyl (1-(4-fluorobutyl)-1*H*-indazole-3-carbonyl)-*L*-phenylalaninate (MPP-4F-BUTINACA, **37**).

## **Figure S72.** LC-UV chromatogram for methyl (1-(4-fluorobutyl)-1*H*-pyrrolo[2,3-*b*]pyridine-3-carbonyl)-*L*-valinate (MMB-4F-BUT7AICA, **38**).

## **Figure S73.** LC-UV chromatogram for methyl (*S*)-2-(1-(4-fluorobutyl)-1*H*-pyrrolo[2,3-*b*]pyridine-3-carboxamido)-3,3-dimethylbutanoate (MDMB-4F-BUT7AICA, **39**).

## **Figure S74.** LC-UV chromatogram for methyl (1-(4-fluorobutyl)-1*H*-pyrrolo[2,3-*b*]pyridine-3-carbonyl)-*L*-phenylalaninate (MPP-4F-BUT7AICA, **40**).

## **Figure S75.** UV spectrum for (*S*)-*N*-(1-amino-3-methyl-1-oxobutan-2-yl)-1-(4-cyanobutyl)-1*H*-indole-3-carboxamide (AB-4CN-BUTICA, **5**).

## **Figure S76.** UV spectrum for (*S*)-*N*-(1-amino-3,3-dimethyl-1-oxobutan-2-yl)-1-(4-cyanobutyl)-1*H*-indole-3-carboxamide (ADB-4CN-BUTICA, **9**).

## **Figure S77.** UV spectrum for (*S*)-*N*-(1-amino-1-oxo-3-phenylpropan-2-yl)-1-(4-cyanobutyl)-1*H*-indole-3-carboxamide (APP-4CN-BUTICA, **10**).

## **Figure S78.** UV spectrum for (*S*)-*N*-(1-amino-3,3-dimethyl-1-oxobutan-2-yl)-1-(4-cyanobutyl)-1*H*-indazole-3-carboxamide (ADB-4CN-BUTINACA, **12**).

## **Figure S79.** UV spectrum for (*S*)-*N*-(1-amino-1-oxo-3-phenylpropan-2-yl)-1-(4-cyanobutyl)-1*H*-indazole-3-carboxamide (APP-4CN-BUTINACA, **13**).

## **Figure S80.** UV spectrum for (*S*)-*N*-(1-amino-3-methyl-1-oxobutan-2-yl)-1-(4-cyanobutyl)-1*H*-pyrrolo[2,3-*b*]pyridine-3-carboxamide (AB-4CN-BUT7AICA, **14**).

## **Figure S81.** UV spectrum for (*S*)-*N*-(1-amino-3,3-dimethyl-1-oxobutan-2-yl)-1-(4-cyanobutyl)-1*H*-pyrrolo[2,3-*b*]pyridine-3-carboxamide (ADB-4CN-BUT7AICA, **15**).

## **Figure S82.** UV spectrum for (*S*)-*N*-(1-amino-1-oxo-3-phenylpropan-2-yl)-1-(4-cyanobutyl)-1*H*-pyrrolo[2,3-*b*]pyridine-3-carboxamide (APP-4CN-BUT7AICA, **16**).

## **Figure S83.** UV spectrum for methyl (*S*)-2-(1-(4-cyanobutyl)-1*H*-indole-3-carboxamido)-3,3-dimethylbutanoate (MDMB-4CN-BUTICA, **18**).

## **Figure S84.** UV spectrum for methyl (1-(4-cyanobutyl)-1*H*-indole-3-carbonyl)-*L*-phenylalaninate (MPP-4CN-BUTICA, **19**).

## **Figure S85.** UV spectrum for methyl (*S*)-2-(1-(4-cyanobutyl)-1*H*-indazole-3-carboxamido)-3,3-dimethylbutanoate (MDMB-4CN-BUTINACA, **20**).

## **Figure S86.** UV spectrum for methyl (1-(4-cyanobutyl)-1*H*-indazole-3-carbonyl)-*L*-phenylalaninate (MPP-4CN-BUTINACA, **21**).

## **Figure S87.** UV spectrum for methyl (*S*)-2-(1-(4-cyanobutyl)-1*H*-pyrrolo[2,3-*b*]pyridine-3-carboxamido)-3,3-dimethylbutanoate (MDMB-4CN-BUT7AICA, **23**).

## **Figure S88.** UV spectrum for methyl (1-(4-cyanobutyl)-1*H*-pyrrolo[2,3-*b*]pyridine-3-carbonyl)-*L*-phenylalaninate (MPP-4CN-BUT7AICA, **24**).

## **Figure S89.** UV spectrum for (*S*)-*N*-(1-amino-3-methyl-1-oxobutan-2-yl)-1-(4-fluorobutyl)-1*H*-indole-3-carboxamide (AB-4F-BUTICA, **25**).

## **Figure S90.** UV spectrum for (*S*)-*N*-(1-amino-3,3-dimethyl-1-oxobutan-2-yl)-1-(4-fluorobutyl)-1*H*-indole-3-carboxamide (ADB-4F-BUTICA, **26**).

## **Figure S91.** UV spectrum for (*S*)-*N*-(1-amino-1-oxo-3-phenylpropan-2-yl)-1-(4-fluorobutyl)-1*H*-indole-3-carboxamide (APP-4F-BUTICA, **27**).

## **Figure S92.** UV spectrum for (*S*)-*N*-(1-amino-3-methyl-1-oxobutan-2-yl)-1-(4-fluorobutyl)-1*H*-indazole-3-carboxamide (AB-4F-BUTINACA, **28**).

## **Figure S93.** UV spectrum for (*S*)-*N*-(1-amino-3,3-dimethyl-1-oxobutan-2-yl)-1-(4-fluorobutyl)-1*H*-indazole-3-carboxamide (ADB-4F-BUTINACA, **29**).

## **Figure S94.** UV spectrum for (*S*)-*N*-(1-amino-1-oxo-3-phenylpropan-2-yl)-1-(4-fluorobutyl)-1*H*-indazole-3-carboxamide (APP-4F-BUTINACA, **30**).

## **Figure S95.** UV spectrum for (*S*)-*N*-(1-amino-3-methyl-1-oxobutan-2-yl)-1-(4-fluorobutyl)-1*H*-pyrrolo[2,3-*b*]pyridine-3-carboxamide (AB-4F-BUT7AICA, **31**).

## **Figure S96.** UV spectrum for (*S*)-*N*-(1-amino-3,3-dimethyl-1-oxobutan-2-yl)-1-(4-fluorobutyl)-1*H*-pyrrolo[2,3-*b*]pyridine-3-carboxamide (ADB-4F-BUT7AICA, **32**).

## **Figure S97.** UV spectrum for (*S*)-*N*-(1-amino-1-oxo-3-phenylpropan-2-yl)-1-(4-fluorobutyl)-1*H*-pyrrolo[2,3-*b*]pyridine-3-carboxamide (APP-4F-BUT7AICA, **33**).

## **Figure S98.** UV spectrum for methyl (1-(4-fluorobutyl)-1*H*-indole-3-carbonyl)-*L*-valinate (MMB-4F-BUTICA, **34**).

## **Figure S99.** UV spectrum for methyl (1-(4-fluorobutyl)-1*H*-indole-3-carbonyl)-*L*-phenylalaninate (MPP-4F-BUTICA, **35**).

## **Figure S100.** UV spectrum for methyl (1-(4-fluorobutyl)-1*H*-indazole-3-carbonyl)-*L*-valinate (MMB-4F-BUTINACA, **36**).

## **Figure S101.** UV spectrum for methyl (1-(4-fluorobutyl)-1*H*-indazole-3-carbonyl)-*L*-phenylalaninate (MPP-4F-BUTINACA, **37**).

## **Figure S102.** UV spectrum for methyl (1-(4-fluorobutyl)-1*H*-pyrrolo[2,3-*b*]pyridine-3-carbonyl)-*L*-valinate (MMB-4F-BUT7AICA, **38**).

## **Figure S103.** UV spectrum for methyl (*S*)-2-(1-(4-fluorobutyl)-1*H*-pyrrolo[2,3-*b*]pyridine-3-carboxamido)-3,3-dimethylbutanoate (MDMB-4F-BUT7AICA, **39**).

## **Figure S104.** UV spectrum for methyl (1-(4-fluorobutyl)-1*H*-pyrrolo[2,3-*b*]pyridine-3-carbonyl)-*L*-phenylalaninate (MPP-4F-BUT7AICA, **40**).

# Table T Strain energy calculations of compounds 5-40

| **Compound** | **Core** | **Head** | **Tail** | **CB_1_ Strain (kcal/mol)** | **∆_strain_** | **CB_2_ Strain (kcal/mol)** | **∆_strain_** |
| --- | --- | --- | --- | --- | --- | --- | --- |
| **14** | 7aza | AB | 4CN | 1.608 | 0.108 | 2.513 | 1.273 |
| **31** | 7aza | AB | 4F | 1.500 |  | 1.240 |  |
| **15** | 7aza | ADB | 4CN | 6.131 | -1.282 | 4.039 | 1.422 |
| **32** | 7aza | ADB | 4F | 7.413 |  | 2.617 |  |
| **22** | 7aza | MMB | 4CN | 1.275 | -0.217 | 1.334 | 1.319 |
| **38** | 7aza | MMB | 4F | 1.492 |  | 0.015 |  |
| **16** | 7aza | APP | 4CN | 3.767 | -0.455 | 6.604 | 0.965 |
| **33** | 7aza | APP | 4F | 4.222 |  | 5.639 |  |
| **23** | 7aza | MDMB | 4CN | 1.360 | 0.136 | 2.678 | 1.201 |
| **39** | 7aza | MDMB | 4F | 1.224 |  | 1.477 |  |
| **24** | 7aza | MPP | 4CN | 12.845 | 5.784 | 12.152 | 3.319 |
| **40** | 7aza | MPP | 4F | 7.061 |  | 8.833 |  |
| **11** | Indazole | AB | 4CN | 1.943 | -1.382 | 0.334 | 0.165 |
| **28** | Indazole | AB | 4F | 3.325 |  | 0.169 |  |
| **12** | Indazole | ADB | 4CN | 8.485 | -0.208 | 6.454 | 3.427 |
| **29** | Indazole | ADB | 4F | 8.693 |  | 3.027 |  |
| **6** | Indazole | MMB | 4CN | 0.012 | -1.283 | 3.975 | 3.800 |
| **36** | Indazole | MMB | 4F | 1.295 |  | 0.175 |  |
| **13** | Indazole | APP | 4CN | 1.711 | 0.073 | 8.010 | 1.275 |
| **30** | Indazole | APP | 4F | 1.638 |  | 6.735 |  |
| **20** | Indazole | MDMB | 4CN | 0.571 | -0.252 | 1.777 | 0.858 |
| **8** | Indazole | MDMB | 4F | 0.823 |  | 0.919 |  |
| **21** | Indazole | MPP | 4CN | 1.145 | -0.641 | 15.514 | 7.820 |
| **37** | Indazole | MPP | 4F | 1.786 |  | 7.694 |  |
| **5** | Indole | AB | 4CN | 1.986 | 0.254 | 3.960 | 1.656 |
| **25** | Indole | AB | 4F | 1.732 |  | 2.304 |  |
| **9** | Indole | ADB | 4CN | 5.880 | 0.220 | 5.667 | 5.657 |
| **26** | Indole | ADB | 4F | 5.660 |  | 0.010 |  |
| **17** | Indole | MMB | 4CN | 0.571 | -0.358 | 1.627 | 1.595 |
| **34** | Indole | MMB | 4F | 0.929 |  | 0.032 |  |
| **10** | Indole | APP | 4CN | 3.531 | 0.207 | 13.244 | 2.927 |
| **27** | Indole | APP | 4F | 3.324 |  | 10.317 |  |
| **18** | Indole | MDMB | 4CN | 1.684 | 0.102 | 3.808 | 2.303 |
| **7** | Indole | MDMB | 4F | 1.582 |  | 1.505 |  |
| **19** | Indole | MPP | 4CN | 4.042 | 3.157 | 12.615 | 4.952 |
| **35** | Indole | MPP | 4F | 0.885 |  | 7.663 |  |

# Calculated binding poses

Representational computationally predicted binding modes of 4CN (orange) and 4F (green) overlayed at the CB_1_ (PDB:6N4B) and CB_2_ (PDB:6PT0) receptors. The hydrophobic regions of the binding site (grey) were evaluated with SiteMap. Glide docking performed using the Schrodinger computational chemistry suite.

## **Figure S105.** Computationally predicted binding modes of **11** (orange) and **28** (green) overlayed at the CB_1_ (PDB:6N4B) receptor.

## **Figure S106.** Computationally predicted binding modes of **13** (orange) and **30** (green) overlayed at the CB_1_ (PDB:6N4B) receptor.

## **Figure S107**. Computationally predicted binding modes of **9** (orange) and **26** (green) overlayed at the CB_1_ (PDB:6N4B) receptor.

## **Figure S108.** Computationally predicted binding modes of **15** (orange) and **32** (green) overlayed at the CB_1_ (PDB:6N4B) receptor.

## **Figure S109.** Computationally predicted binding modes of **23** (orange) and **39** (green) overlayed at the CB_1_ (PDB:6N4B) receptor.

## **Figure S110.** Computationally predicted binding modes of **18** (orange) and **7** (green) overlayed at the CB_1_ (PDB:6N4B) receptor.

## **Figure S111.** Computationally predicted binding modes of **11** (orange) and **28** (green) overlayed at the CB_2_ (PDB:6PT0) receptor.

## **Figure S112.** Computationally predicted binding modes of **13** (orange) and **30** (green) overlayed at the CB_2_ (PDB:6PT0) receptor.

## **Figure S113.** Computationally predicted binding modes of **9** (orange) and **26** (green) overlayed at the CB_2_ (PDB:6PT0) receptor.

## **Figure S114.** Computationally predicted binding modes of **15** (orange) and **32** (green) overlayed at the CB_2_ (PDB:6PT0) receptor.

## **Figure S115.** Computationally predicted binding modes of **23** (orange) and **39** (green) overlayed at the CB_2_ (PDB:6PT0) receptor.

## **Figure S116.** Computationally predicted binding modes of **18** (orange) and **7** (green) overlayed at the CB_2_ (PDB:6PT0) receptor.
